# The relevance of sustainable laboratory practices†

**DOI:** 10.1039/d4su00056k

**Published:** 2024-03-18

**Authors:** Thomas Freese, Nils Elzinga, Matthias Heinemann, Michael M. Lerch, Ben L. Feringa

**Affiliations:** a Stratingh Institute for Chemistry, University of Groningen Nijenborgh 4 9747 AG Groningen The Netherlands m.m.lerch@rug.nl b.l.feringa@rug.nl; b Green Office, University of Groningen Broerstraat 5 9712 CP Groningen The Netherlands; c Groningen Biomolecular Sciences and Biotechnology Institute, University of Groningen Nijenborgh 4 9747 AG Groningen The Netherlands

## Abstract

Scientists are of key importance to the society to advocate awareness of the climate crisis and its underlying scientific evidence and provide solutions for a sustainable future. As much as scientific research has led to great achievements and benefits, traditional laboratory practices come with unintended environmental consequences. Scientists, while providing solutions to climate problems and educating the young innovators of the future, are also part of the problem: excessive energy consumption, (hazardous) waste generation, and resource depletion. Through their own research operations, science, research and laboratories have a significant carbon footprint and contribute to the climate crisis. Climate change requires a rapid response across all sectors of society, modeled by inspiring leaders. A broader scientific community that takes concrete actions would serve as an important step in convincing the general public of similar actions. Over the past years, grassroots movements across the sciences have recognized the overlooked impact of the scientific enterprise, and so-called Green Lab initiatives emerged seeking to address the environmental footprint of research. Driven by the voluntary efforts of researchers and staff, they educate peers, develop sustainability guidelines, write scientific publications and maintain accreditation frameworks. With this perspective we want to advocate for and spark leadership to promote a systemic change in laboratory practices and approach to research. Comprehensive evidence for the environmental impact of laboratories and their root-causes is presented, expanded with data from a current case study of the University of Groningen showcasing annual savings of 398 763 € as well as 477.1 tons of CO_2_e. This is followed by guidelines for sustainable lab practices and hands-on advice on how to achieve a systemic change at research institutions and industry. How can we expect industry, politics, and society to change, if we as scientists are not changing either? Scientists should lead by example and practice the change they want to see.

## Abstract 2

Traditional lab practices contribute to environmental issues through excessive energy consumption, hazardous- and single-use waste generation, and resource depletion. Sustainable laboratory practices are vital for the education of future scientists.

## Graphical abstract

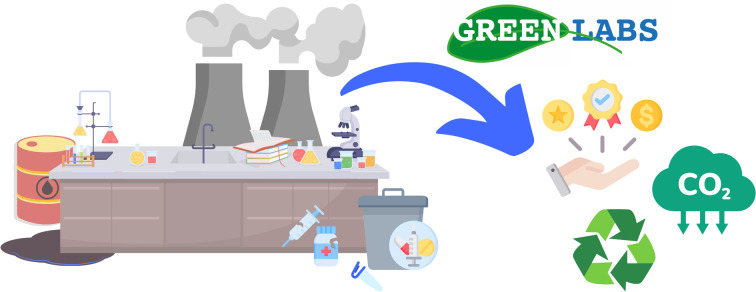

**Sustainability spotlight**

Researchers across the planet play a key role in enhancing the energy-, feedstock- and material-transition necessary to allow for circularity and thus mitigation of the climate crisis. Their contribution, education and skills are necessary to develop the sustainable future by meeting the UN's Sustainable Development Goals (SDGs). Scientific research is also vital for climate awareness and solutions, however, it paradoxically contributes to environmental issues through traditional laboratory practices. Operating laboratories and conducting experiments leads to excessive energy consumption, (hazardous) waste generation, and resource depletion. With this perspective we want to provide and spark quality education among scientists on how to improve as a community on research and laboratory operations (SDG No. 4). We provide institutional evidence and advice on a systemic change in academia, industry and (procurement) infrastructure to allow for responsible consumption (SDGs No. 9, 12).

## 1 Introduction

Scientific research and scientists are committed to advancing human knowledge, understanding nature, providing a basis for innovation, educating society and improving humanity.^1–4^ Building on fundamental discoveries, by strictly following the scientific method and the ethical conduct of research, scientists are the reason that society was able to drastically reduce the consequences of the COVID-19 pandemic.^5,6^ Their research is mainly conducted in laboratories, which can be seen as the hubs of discovery and innovation.^7–9^ At such research institutions, scientists have been at the forefront of fighting side-effects of the Anthropocene, where in the recent decade climate change, fossil resource depletion, and pollution have taken more centre stage and received more funding.^10^ Through the ground-breaking work of many scientists uncovering the dramatic extent and mechanistic underpinnings of climate change, society and political players have become more aware of the fact that the climate crisis represents one of the most pressing challenges ever faced by humanity.^11^ Anthropogenic greenhouse gas emissions are driving extreme weather events, food shortages, biodiversity loss and water insecurity and thus must be cut drastically to reach net zero by 2050, limiting further heating to 1.5 °C.^12–14^

**Fig. 1:**
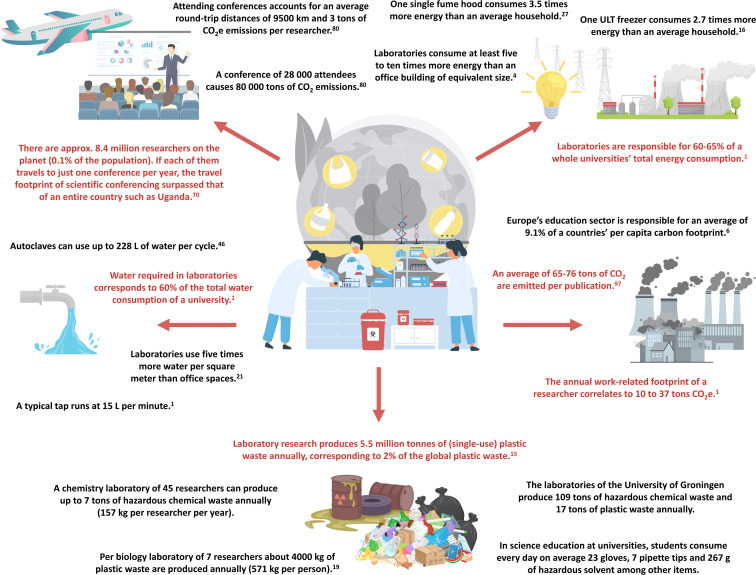
The overlooked environmental impact of laboratories and scientific research. Data without citation were generated through this research article.

Although scientific research has achieved remarkable successes and advantages, conventional laboratory methods carry unintended drawbacks. Scientists paradoxically contribute to environmental challenges despite their attempts to address them through research. Laboratory practices are marked by excessive energy consumption, waste generation, and resource depletion (Fig. 1).^7^ Laboratories have the highest mean heating and electricity consumption of an institution, being responsible for 60–65% of the whole university's total energy consumption.^1^ Laboratory research produces an estimated 5.5 million tonnes of (single-use) plastic waste annually, corresponding to 2% of the global plastic waste.^15^ Resources such as water required in laboratories for cooling and washing correspond to 60% of the total water consumption of a university.^1^ All together the annual work-related footprint of a researcher correlates to 10 to 37 tons of CO_2_ equivalents (CO_2_e), which is much higher than the Paris aligned annual carbon budget of 1.5 tons CO_2_e to maintain the climate.^1,7,16^

It is in our very nature as scientists that we want to understand life, molecules, materials, physical principles, and its laws of nature – however by our actions we are not only harming ourselves but also the planet surrounding us.^17–19^ While we investigate environmental problems and recommend sustainable practices and solutions across several sectors, there clearly exists friction between our scientific practices and their potential contribution to mitigate climate change and meet the UN Sustainable Development Goals (SDGs).^20,21^ Current laboratory practices are outdated^22^ and need thorough transformation alongside all other sectors of society.^3^ Over the past years, scientists – increasingly aware of these environmental concerns – have been demanding sustainable laboratory practices as a response and recognizing the importance of starting with changing their own operations.^5,8,23^

These sustainable laboratory practices are so far independent of the underlying research questions conducted in the laboratory and are often referred to as Green Labs.^1^ Green lab efforts focus on improving resource and energy efficiency, waste reduction, and environmental responsibility. In this way, Green Labs serve as the scientists' answers to the dissonance within their own sector and as an acknowledgement of academia's role in serving as a role model to educate and prepare future generations, researchers, innovators, and decision makers for the challenges ahead.

Fact-based analyses on the environmental impact of research and laboratory practices form the core of Green Lab movements and promise a reduction in the overall environmental footprint of a given institution towards a net-zero climate goal, all of which further contribute to cost reductions, without comprising or limiting the research or results obtained in a laboratory. Hence, scientists play a crucial role in not only providing solutions to climate change but also to renew and update their own workplace and laboratory practices. The education of students about sustainable laboratory practices will ultimately lead to a bottom-up paradigm shift in many organizations across society. Ultimately, a scientific community that is taking concrete actions serves as an important step in convincing the general public of similar actions.^16^

In this article we provide a comprehensive literature review on the environmental impact of laboratories, collecting most recent evidence for the systemic change necessary. We further explore the benefits, challenges, and implications of transitioning to green labs, with a concrete case study and supporting data of the University of Groningen, NL. The last section provides guidelines for sustainable lab practices and hands-on advice on how to achieve the desired systemic change. These guidelines synthesize relevant literature, a sequential plan on concrete green lab measures, accreditation programs, and success factors in transitioning to sustainable laboratories. Disciplines such as astronomy, biology, chemistry, computational science, life sciences, neuroscience, pharmacy, and physics will be covered. Given that the absence of resources is frequently highlighted as a significant knowledge gap, we envision this article as a comprehensive tool in understanding the relevance of sustainable laboratory practices and on how to improve as a scientific community. This article aids the scientific community in convincing peers and supervisors on the environmental and financial opportunities by improving their research practices and science.

## 2 Environmental impact of laboratories

Common research and laboratory practices are often not in line with UN Sustainable Development Goals, even though they are designed to address those goals. Through their work, scientists contribute to greenhouse gas emissions, plastic waste production, and toxic waste, among many other issues.

Scientific experiments are often resource intensive and wasteful, both in wet and dry lab (computational) facilities. Many experiments require temperature control, increased ventilation, vacuum, protective atmospheres, or high sterility. Next to operating equipment in a laboratory building, the manufacturing and disposal of (single-use) laboratory consumables can be resource- as well as energy-intensive. Also, computational laboratories are energy-intensive through running code, processing algorithms, and data generation in high-performance computing (HPC) facilities. To reach net-zero targets, research needs to be conducted in a way that protects the environment, reducing CO_2_ emissions, and preserve natural resources by allowing for regeneration and limiting waste.^2^ The following section will highlight the evidence and analyses on the environmental footprint of laboratories.

Direct and indirect carbon emissions are reported according to the Greenhouse Gas Protocol with the following three categories:^24^

Scope 1: direct emissions from refrigerants, on-site electricity generation, heating, and vehicles.

Scope 2: indirect emissions from electricity or energy purchased for heating or cooling of buildings, which is generated off-site.

Scope 3: indirect emissions across an organisation's whole value chain, such as products purchased from suppliers and sold to customers. This also includes travel, production of laboratory equipment, chemicals, materials, and waste disposal.

### 2.1 Energy and electricity

As each laboratory is different in its operations and research conducted, there are variations in the energy and electricity consumption.^25^ Generally, research laboratories consume more energy per square meter than any other sector except from ones operating data centres.^5^ Laboratories consume at least five to ten times more energy than an office building of equivalent size.^4,26^ This value can increase to up to 100 times more energy than office space, depending on the number of clean rooms and high process operations.^1^ A laboratory's heating, ventilation, and air conditioning system has air flow rates two to ten times higher than in office buildings, and ventilation comprises the single most energy-intensive service in laboratory buildings.^4,27^ In fact, laboratories have the highest mean heating and electricity consumption of an institution, being responsible for 60–65% of an entire university's total energy consumption.^1^

These numbers are correlated with the equipment being operated in these research spaces. Fume hoods and ultra-low temperature (ULT) freezers are among the most energy intensive:^28^

- One single fume hood consumes 3.5 times more energy than an average household.^27,29,30^

- One ULT freezer consumes 2.7 times more energy than an average household (20–25 kW h per day).^16,31,32^

In the US, approximately 500 000 to 1 500 000 fume hoods are being operated, with operating costs exceeding $6 billion per year.^29^ Harvard University estimates that 44% of the energy used in a lab is directly related to ventilation, with an average annual cost of operation of 4107 € per fume hood, and 3103 € per ULT freezer.^26,33^ Based on further reports, the other 10–50% of energy is used by (plug-in) equipment such as freezers, autoclaves or centrifuges, where up to 15% can be consumed by lighting.^18,28^ Standard freezers at temperatures of −20 °C or biosafety cabinets consume as much energy as 0.5 households.^18^ Some laboratories operate facilities such as X-ray light sources, synchrotrons and high-performance computing clusters, increasing the energy consumption drastically. Here power demands can range from MW scale, corresponding to tens of GW h of electricity annually, to hundreds of MWs in the case of the European Organization for Nuclear Research (CERN), which accounts for approximately 2% of Swiss electricity consumption.^1,34,35^

#### 2.1.1 Research data management and computation

As digitisation of research proceeds, more data is generated and needs to be stored. The carbon impact of an email is about the same as that of a disposable plastic bag and depending on attachments costs between 0.3 and 26 g CO_2_e. Global email communication is estimated at 150 million tons of CO_2_e accounting for 0.3% of the planet's anthropogenic carbon footprint.^36^

Running algorithms, artificial intelligence, high-performance computing and maintaining hardware all correlate with energy usage and ultimately with an environmental footprint.^23,37,38^ Tasks such as training an artificial intelligence model corresponds to 5 times the lifetime emissions of an average American car.^39^

Around 100–150 million tons of carbon dioxide emissions are generated annually by the digital sector, and the yearly electricity usage of data centres is over 205 TW h.^38,40^ This consumption surpasses the energy usage of entire countries, including Ireland and Denmark.^41,42^ The impact from electricity consumption only for supercomputing generates an average of 4.6 tons of CO_2_e per researcher working in astronomy per year.^43^ In particular, astronomers, who operate telescopes and observatories as well as high-performance computing centres, have a drastic annual carbon footprint of up to 37 tons CO_2_e emissions per researcher (60% from supercomputer usage, 17% from flying, 13% from operation of observatories, and 10% from powering office buildings.^44,45^

The estimated computing requirements of Australian astronomers is 400 million CPU core-hours (MCPUh) annually, with an anticipated increase to 500 MCPUh per year by 2025.^45^ The Institute of Research in Astrophysics and Planetology (IRAP) in France estimated that running observatories and generating observatory data correspond to 4100 tonnes of CO_2_ per year, the equivalent of 2050 petrol cars running all year.^17^

### 2.2 Water and other resources

Preserving water resources is another crucial aspect when it comes to laboratory sustainability. Water has to be cleaned, transported, stored and cleaned again before being returned to the environment. Next to the limited amount of fresh water, those actions require energy and have a carbon footprint.^46^ Laboratories use about five times more water per square meter than office spaces.^21,47^ Water usage can be associated with different purposes in a laboratory, of which most of the times it is used for cooling or washing. In fact, water as a resource required in laboratories corresponds to 60% of the total water consumption of a university.^1^ Autoclaves can use vast amounts of water to sterilize equipment, reagents, and waste. They are the primary water consumers in a laboratory, where, depending on the size and model of the autoclave (*i.e*. constant flow), one cycle can use up to 228 L of water, corresponding to 2955 L a day.^46,48^ Single-pass water cooling in a chemistry laboratory consumes 909 218 L of water per reaction per year and can lead to flooding.^48^ A typical tap runs at 15 L per minute, which next to cleaning of glassware is also utilized for cooling of reactions or as vacuum pump devices.^1^

Other resources such as helium are utilized for cooling in nuclear magnetic resonance (NMR) spectroscopy and in MRI imaging. It is further used as a carrier gas in gas chromatography/mass spectrometry (GC-MS) analysis.^1^ Liquid nitrogen is also used excessively as a cooling agent, and its usage can be optimized as well.

### 2.3 Waste

#### 2.3.1 Chemicals

Many experiments (when not computational) rely on the usage of chemicals in the form of reagents, catalysts, buffers, and solvents. Subsequent purification steps (separation or washing) further require chemicals. Analysis techniques also rely on chemicals, to improve measurement conditions. Altogether, chemicals come with health impacts and with a certain carbon footprint as they are sourced, produced, and disposed of. Traditional chemical manufacturing processes release 2.55 kg CO_2_e per kg of acetone and 1.85 kg CO_2_e per kg of isopropanol into the atmosphere.^49^ A study from France indicates, that their laboratories (468 employees with 315 fume hoods) consume up to 10 000 liters of acetone per year, of which incineration leads to 18.1 tons of CO_2_e annually.^25^ The less optimized a reaction, experiment size or purification technique, the more pollution of air, water and soil can be correlated with an experiment. If reactions or techniques are unreliable and thus not reproducible, tons of chemical waste are unnecessarily generated, which could have been avoided by reporting reaction conditions accordingly.

#### 2.3.2 Single-use plastics

Many research spaces rely on the use of single-use plastics and consumables, where biological and medical laboratories have larger plastic consumption through carrying out cell culture work or have higher requirements for sterility. Common types are polyethylene (PE), polypropylene (PP), polyethylene terephthalate (PET), polymethyl methacrylate (PMMA) or polystyrene (PS). Plastic products utilized in laboratories range from pipette tips, tubes, or filter bottles to gloves, weighing boats, well plates and packaging. More than 90% of the plastic in a pipette tip box comes from the box itself, highlighting the importance for its reuse.^50^ Single-use plastic usage of students may exceed more than 24 000 pieces per student per year.^51^ Per biology laboratory about 4000 kg of plastic waste is produced annually (7 researchers).^19,52^ World-wide laboratory research produces an estimated 5.5 million tonnes of (single-use) plastic waste annually, corresponding to 2% of the global plastic waste.^15^

#### 2.3.3 Glass

Glass is a great example on how to replace single-use consumables and plastics, which is standard especially in chemical laboratories. However, there are also glass disposables, which should be washed and reused to reduce the total amount of laboratory waste.

#### 2.3.4 Paper and cardboard

Printing of articles, slides, or protocols generates unnecessary paper waste, especially if being disposed of after a short period of time. Observations, notes, and experiments traditionally were documented on paper-based laboratory journals. Electronic lab journals provide an appealing alternative to paper-based laboratory journals, avoiding paper waste as well as physical storage.

Uncoordinated on-demand shipments of chemicals and equipment result in parallel shipments from the same manufacturer or supplier to an organization resulting in avoidable cardboard boxes and packaging material waste.

#### 2.3.5 Electronic waste and equipment

Many researchers rely on high performance computing and calculations. As technology and electronics are rapidly growing fields, outdated servers and computers are being disposed of as electronic waste (e-waste) regularly. The electronics supply chain needs to be maintained as well in order to produce better performing devices.^53^ Here unsustainable mining practices as well as e-waste disposal impact the environment throughout the lifecycle of research hardware.^41^ In 2019, about 53.6 million tons of e-waste were generated globally, of which about one-fifth is formally collected and recycled.^54,55^

#### 2.3.6 Animal models

In the UK, about 1.44 million animals were used for experiments in 2020.^56^*In vivo* models often fail to mimic clinical diseases adequately and thus harm human subjects.^57–62^ In fact, around 92% of drugs tested in animals as a preclinical step fail to pass the clinical stage.^63^ As the success rate from animal-model based studies to clinical evaluations can be less than 10%, the number of animals should be critically evaluated for ethical as well as sustainability reasons and alternatives considered.^56,64–66^

Furthermore, animal facilities can have a considerable impact on the energy consumption of an organization and thus should be reduced in size and optimized.

#### 2.3.7 Lab coats

Wet labs utilize cotton lab coats, which are washed and reused over the course of several years of a research project. However, as soon as staff departs the lab coats often stay behind, ending up in a storage cabinet or as waste.

### 2.4 Conferences and travel

The global aviation industry is responsible for the combustion of 5 million barrels of jet fuel per day, resulting in the daily release of 2.4 million tonnes of CO_2_ (3.8% of global carbon dioxide emissions).^6,16^ Flying across the Atlantic and back generates roughly the same carbon footprint as a typical year of commuting by car.^16^ Relative to the general public, scientists travel a lot. Academics are responsible for a considerable amount of greenhouse gas emissions due to flying.^3,67^ At the University of British Columbia, the travel related greenhouse gas emissions from business-related flights from employees contributed to two thirds of the total emissions of campus operations.^45,68^ These numbers, however, are reduced when complete (Scope 1–3) carbon impact calculations are conducted (*e.g.*, 16% of the carbon emissions of the University of Groningen are business-related flights).

Scientific discoveries often rely on collaborations, human interactions, and discussions. Attending conferences, workshops and seminars is thus a crucial aspect of being a researcher or academic. However, emissions related to in-person conference attendance accounts for half of an academic's flight emissions.^45,69^ There are 8.4 million researchers on the planet, where if assumed that each of them travels to just one conference per year, the travel footprint of scientific conferencing surpassed the total CO_2_ emissions of entire countries such as Uganda.^70^ Conference attendance accounts for 35% of a researcher's footprint (*e.g.*, 4 years of PhD study).^71^

The magnitude of the travel-related carbon footprint of the scientific community can be illustrated by analysing conferences such as the annual meeting of the Society for Neuroscience (SFN), which attracts about 30 000 attendees to the United States from across the globe.^72^ Comparing the city of origin of attendees to the meeting in Washington, DC in 2014, an estimated round-trip distance per person of 7500 km was obtained. Resulting in 22 000 metric tons of CO_2_e generated only through this conference, the emissions surpass the annual carbon footprint of about 1000 medium-sized laboratories.^16^

Other disciplines such as astronomy regularly estimate the footprint of conferences and institutes.^73–76^ The 2019 annual European Astronomical Society meeting in Lyon (European Week of Astronomy and Space Science) resulted in 1.9 tons CO_2_e per participant (1240), which are comparable to the annual per capita emissions of countries such as India.^73^ Attending the International Biogeography Society (IBS) meeting accounts for an average round-trip air travel distance of 9564 km and 3.0 tons of CO_2_e per researcher (a total of 857 tons of CO_2_e per conference).^77^

Another research study revealed that presenting a conference paper results in at least 800 kg of CO_2_e and increases for presentations located in popular but non-central locations such as Hawaii (1290 kg per paper).^78,79^

For the fall meeting of the American Geophysical Union 28 000 participants travelled 285 million kilometres towards and back, corresponding to twice the distance between the Earth and the Sun. Through attendance 80 000 tons of CO_2_e were emitted, which is about 3 tons CO_2_e per scientist equal to the weekly emissions of the city of Edinburgh, UK.^80^ Estimations from 2008 indicate that science travel emissions in that year accounted for 0.23% of all international aviation emissions, corresponding to 4.3 tons CO_2_ per capita.^78^

Flight frequency and emissions scale with scientist seniority, with an average senior staff member emitting 9.5–12 equivalent tonnes of CO_2_, a postdoc emitting 4 tons CO_2_e and a PhD candidate 2 tons CO_2_e per year.^45,81^ Most notably however, there is no relationship between scholarly success and air travel emissions based on metrics of academic productivity (*h*-index adjusted for academic age, discipline and seniority).^80,82^ When it comes to disciplines and flight-related emissions, astronomers emit 8.5 tons CO_2_e per researcher annually.^44^

The current modes of work, cooperation, mobility, and internationalisation are key aspects of the academic life, but conference models need to be optimized to mitigate future emissions.^3^

### 2.5 Buildings and construction

Many aspects of laboratory resource efficiency are determined during building design.^4^ With growing environmental concerns, the construction sector is improving its practices embedding sustainable design, cement, buildings, including sustainable cement production.^83^ This is much needed as about 40% of global anthropogenic emissions are attributed to buildings and construction, 11% of which results from manufacturing building materials and products such as steel, cement and glass.^84,85^ The impact of laboratory buildings must be considered during design, construction, operation and demolition. As laboratory buildings need to support heavy equipment and minimize vibrations for sensitive measurements and imaging, their embodied carbon is two times higher than that of commercial office buildings.^8^

The energy challenge that laboratory building designers are confronted with is the large volume of air ventilation needed to meet safety requirements (see above). Whereas office buildings require a ventilation standard of one air change per hour (ACH) or less, laboratory buildings require exchange rates of 6 to 10 ACH, corresponding to every 6 to 10 minutes.^4,27^ Laboratory buildings also require unusually high and stable plug loads, *i.e.*, the energy required to operate centrifuges, ovens, computers, or spectrometers in comparison to other institutional or commercial buildings.^4^

### 2.6 Greenhouse gas emissions

Summarizing the previous sections and the correlated Scope 1–3 emissions, estimations of the cumulative equivalent tonnes of CO_2_ can be made. The estimated annual carbon impact of biotech and pharmaceutical companies, excluding academic laboratories, is about 200 million tonnes of CO_2_e, being equal to half of the emissions of the United Kingdom (UK).^8,21^ Emissions from that sector were found to have risen every year (from 3.9% in 2021 to 5% in 2022).^86^ The carbon footprint of the pharmaceutical and biotechnology industry surpasses that of the semiconductor industry, as well as the forestry and paper industry.^21^ Emissions associated with clinical trials are connected to the energy consumed in the coordinating centres, trial related travel as well as material distribution and delivery.^65^ One single clinical trial produces around 180 tonnes of CO_2_ per year.^2,87^ Clinical trials and science are estimated to be responsible for the equivalent of 100 million tonnes of CO_2_ emissions annually.^65,88^ If clinical science were a country it would rank as the 40th largest emitting country in the world above Nigeria and Bangladesh, each of which has more than 100 million people.^65,88^ In 2015, the carbon emission intensity of the global pharmaceutical industry exceeded that of the automotive industry by 55%.^89^ Furthermore, the combined climate footprint of healthcare facilities, including hospitals and laboratories, accounted for 4.4% of the total global carbon emissions.^89^ Europe's education sector (0.91 Mt CO_2_e per capita) is responsible for an average of 9.1% of a country's carbon footprint per capita, which exceeds the often-discussed aviation sector corresponding to “only” 3.8% of total CO_2_ emissions.^6,85^

One lab at the Massachusetts Institute of Technology (MIT) spent about 30 000 $ on electricity and released 163 tonnes of CO_2_ per year.^18^ The average emissions per research laboratory are approximately 479 tons of CO_2_e.^90^ Greenhouse gas emissions from universities range from 1 to more than 37 tons CO_2_e emissions per employee.^3^ Differences in numbers can be attributed to incomplete inclusion of different sources of emissions in calculations. There is a wide gap between the numbers reported and thorough tracking of Scope 1–3 emissions such as transport, supply chain and building activities. Greenhouse gas emissions of molecular biology or chemistry research for example fall into four categories: real estate and infrastructure; travel and commuting; production, transport and disposal of equipment and chemicals; energy usage by heating, ventilation, and air conditioning of laboratory buildings.^7,27^

Several individual disciplines or research institutes aim for complete footprints: astronomy reports about 18–37 tons CO_2_e annually per researcher, chemistry approximately 5.6–9.6 tons of CO_2_e, and life sciences about 4–15 tons of CO_2_e, each of those on top of personal emissions (*i.e.*, private life).^3,7,25,43,44^ The main sources are air travel, electricity use and computing (up to 22 tons CO_2_e).^3^ In 2021 the average greenhouse gas emissions per capita in Europe were 7.7 tons of CO_2_e per year, indicating that scientists' research activities emit 2–5 times as much as an average person.^91^

Many funding organisations are yet to start reporting greenhouse gas emissions. Estimates indicate that an international panel of referees and group of applicants emit per interview more than 1 ton of CO_2_e.

Generally, where climate reports exist, they are often not comprehensive across all scopes of emissions and thus are underestimating its complete environmental footprint.^85,92^ For the ones aiming to be complete, the share of Scope 3 emissions of the total carbon footprint is 5–10× greater than the amount of the other categories.^3,6^ This corresponds to 80–90% of an organization's footprint being associated with Scope 3 emissions.^6,7,25,56^ Often air travel is reported to be the major source of emissions, because it is easily tracked and thus followed, but especially from organizations where all scopes of emissions are reported, other sources such as buildings, electricity, and supply-chain emissions may be equally or more important emission sources.^3,93^

The previous data drastically highlight the impact scientific research has on the planet, especially given the fact that researchers of all disciplines only account for 0.1% of the population.^94^ It is clear that while science allows us to understand and address the climate crisis, it also enables and contributes to it.^10^

## 3 Case study – Green Labs at the University of Groningen

Several universities across the world have ambitious goals regarding sustainability, whose efforts and actions can be compared in the UI Green Metric World University Rankings (https://greenmetric.ui.ac.id/). From all the 1183 participating universities worldwide, the University of Groningen (UG) was ranked 4th in the 2023 Green Metric Ranking.^95^ Categories such as setting & infrastructure, energy & climate change, waste, water, transportation, and education & research are compared where the University of Groningen achieved an overall score of 9450 (with 1325, 1175, 1800, 1000, 1800, and 1750 points in each category, respectively). Sustainability is one of the key values of the University of Groningen. This means that the UG aims to integrate sustainable development into facets such as research and education, its operational management structure and business operations. The university intends to set an inspiring example, promoting sustainability in a transparent way and actively involving, training, and facilitating students in sustainable activities at a regional, national, and international level.

### 3.1 Sustainability ambitions and organization

For the past ten years, the Green Office (GO) has worked on the UG's sustainable goals and ambitions, based on the Sustainability Roadmap (2.1 ESI†). Based on new legislation related to sustainability, the Netherlands' Climate Agreement, and the Sustainable Development Goals (SDGs) by the United Nations, the Green Office (as part of the Sustainability Programme) developed a Sustainability Roadmap through collaboration between academics, service units, faculties, staff members and students. The ambitions set out in the Roadmap have been the driving force behind its implementation and the sustainability policy within the UG, being among the front runners when it comes to sustainability. These ambitions and goals have been formulated around the central themes of Planet (the UG to become a CO_2_-neutral university by 2035), Performance (more involvement of students, staff members and external parties in sustainability) and People (a sustainable HR policy for a dynamic and healthy organization). All ambitions are represented in one visual overview (Fig. S4, ESI†), from biodiversity to sustainable tendering and from waste separation to interdisciplinary research (see the ESI† for more details).

Independently, a grassroots group for greener laboratories was formed at the Faculty of Science of Engineering (FSE) within the life sciences department in 2020. Another separate movement on sustainability of laboratories was growing at the Stratingh Institute for Chemistry in 2021, both of which combined to Green Labs RUG in 2022.

In line with these ambitions, the Faculty of Science and Engineering (FSE) launched the “*FSE is going green*” program in 2022. Several working groups were formed, consisting of staff and students, whose topics were based on an internal assessment of the most pressing sustainability issues within the faculty and ideas from staff and students. Next to adopting the previously mentioned Green Labs RUG team and incorporating it under the “*FSE goes green*” umbrella, other working groups include: sustainable canteens, food and events, travel behaviour of staff, greening our grounds and sustainable logistics. Efforts have already resulted in a sustainable canteen pilot, improved building insulation, accreditation of sustainable laboratories and advice about staff air travel reduction, based on a faculty-wide survey.

The grassroots movements focusing on sustainable laboratory practices (Green Labs RUG) achieved full top-down faculty support through the “*FSE is going green*” program, which also allowed for funding. These efforts are further in line with the sustainability roadmap, and thus supported and coordinated together with the Green Office. With the following sections we want to provide and highlight data that we gathered through improving and evaluating current laboratory practices. By expanding the literature precedent, we further provide more proof for the relevance of sustainable laboratory practices, its benefits and elaborate on certain actions.

### 3.2 Greenhouse gas emissions

**Fig. 2:**
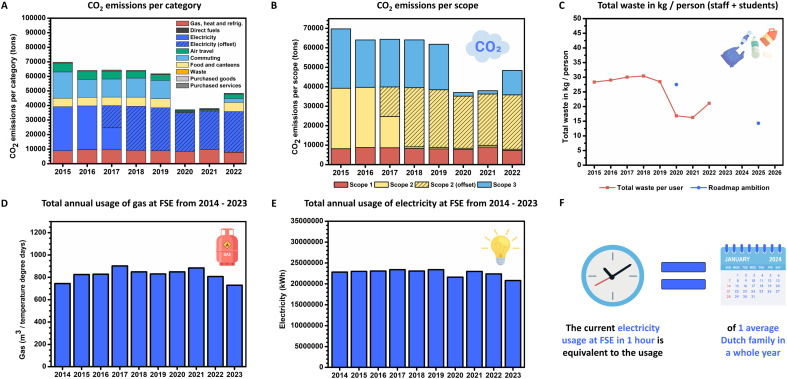
A case study on emissions and energy consumption of the University of Groningen (UG) and Faculty of Science and Engineering (FSE). (A) CO_2_e emissions per category of the University of Groningen (UG). Notes: since 2018 the CO_2_ emissions connected to electricity consumption (Scope 2) dropped significantly: parts of the electricity are geothermically self-produced (16 000 000 kW h annually) and 2% of the total energy use is self-produced through solar energy (1 385 250 kW h). Green energy certificates started in 2018 (53 126 001 kW h annually). Food data and correlated emissions were reported until 2019, but a change in the host of the canteens resulted in inaccurately reported data since 2020, thus the average of the years 2018/2019 was used to estimate the food emissions for the years from 2020 onwards. 2021 and 2022 had a significant lower air travel as well as commuting footprint due to the COVID-19 crisis. Commuting values stayed low in 2022 due to the implementation of home office opportunities and upgrading the public transport in Groningen to full electric. Air travel of 2022 increased back to pre-pandemic times. (B) CO_2_e emissions per scope of UG. (C) Waste production at UG and sustainability ambitions. (D) Annual usage of gas for heating at the Faculty of Science and Engineering (FSE) from 2014 to 2023; weather-normalized consumption *via* temperature degree days measured in Eelde (NL). (E) Annual usage of electricity at FSE from 2014 to 2023. (F) Comparison of electricity usage at FSE with an average Dutch household.

The CO_2_e emissions per category and scope of the UG are depicted in Fig. 2A and B. Emissions from gas usage for heating did not decrease significantly at UG in the period from 2015 to 2023 (Fig. 2A and B), resulting in steady Scope 1 emissions. The total gas usage at UG in 2022 was 3 825 124 m^3^, of which 2 234 140 m^3^ (2022) were used at FSE equalling to 58%. While being the main contributor to the gas consumption of the UG a trend is visible for FSE in Fig. 2D: after weather-normalizing *via* temperature degree days measured in Eelde (NL), the gas consumption decreased over the years from 2021 to 2023 by 17% (884 m^3^ in 2021, 808 m^3^ in 2022, 730 m^3^ in 2023 per temperature degree day, see Table S9 ESI† for more details).

Since 2018, the CO_2_ emissions connected to electricity consumption (Scope 2) dropped significantly: parts of the electricity are geothermically self-produced (16 000 000 kW h annually) and 2% of the total energy use are self-produced through solar energy (1 385 250 kW h). Green energy certificates started in 2018 (53 126 001–57 811 385 kW h annually), which are certified as wind- and solar energy from The Netherlands. Thus Scope 2 (currently produced and delivered through Vattenfall^©^) decreases through electricity certificates by vertiCer^©^ from 2018 onwards. These, however, should only be treated as a ‘transition period’ and not as a final solution to the emissions caused by energy and electricity. As of time of writing the authors are not aware of any further improvements or concrete plans related to the carbon impact of energy usage at UG, but recommend transitioning to a green energy provider.

The UG aims to provide complete calculations for Scope 3 emissions: while food data and correlated emissions were reported until 2019 a change in the host of the canteens resulted in inaccurately reported data since 2020, thus the average of the years 2018/2019 was used to estimate the food emissions for the following years. The Green Office is currently investigating these issues to track food data in the future.

2021 and 2022 had a significant lower air travel as well as commuting footprint due to the COVID-19 crisis. Commuting values stayed low in 2022 due to implementation of home office opportunities and upgrading the public transport in Groningen to full electric. Air travel of 2022 increased back to pre-pandemic times.

Scope 3 emissions cover all other categories, which in comparison to the previously mentioned categories are not contributing too much to the overall footprint of the UG. The emissions of waste are relatively low when compared to electricity, gas, food, commuting or air travel. While the footprint of waste should not be underestimated, these values put the future sections covering laboratory waste (chemicals and plastics) in perspective as still drastic numbers of hazardous and plastic waste are produced annually. The waste category in Fig. 2A includes all residual, plastic, organic, paper, coffee cup waste, as well as electronic, glass, and hazardous waste. Under ‘purchased goods’ the water consumption (*e.g.*, 154 425 m^3^ (2019); 93 573 m^3^ (2020), 130 769 m^3^ (2022)) is included.

It is crucial to mention that although the carbon footprint calculations of the university are almost complete, not all emissions of Scope 3 are included yet. Buildings, their construction, and reconstruction, are not included in the Scope 3 emissions of the university. However, as of 2024 there might be an opportunity as a new chemistry, physics, and engineering building (Feringa Building) will be finished, which will contain a great number of disciplines and laboratories of the Faculty of Science and Engineering. Here the material used for its construction should be included in future calculations of the UG, which could potentially be used as basis to extrapolate to the other buildings.

As for FSE, the move from one location to the other requires an inventory of precision instruments, laboratory, and office equipment, and of the furniture per lab and office, allowing for an extrapolation on furniture and laboratory equipment to the whole university. Thus, an inventory was made of office chairs, desks, drawer units, cabinets, meeting chairs and tables at the location Nijenborgh 4 (NB4). After conducting a life cycle assessment (LCA) including all relevant data (production, materials, resources, packaging materials and transportation) for all goods the CO_2_e of furniture at the NB4 location corresponded to 1032 tons (2.7.9 ESI†). We further calculated the annual CO_2_e emissions through correlating the emissions to average years of depreciation of ten years, resulting in 103 tons CO_2_e annually. Afterwards the m^2^ of office and meeting room space of each building at FSE was used to calculate the furniture footprint per building as well as the total, which corresponded to an annual CO_2_e footprint of 304 tons for FSE (Table S48 ESI†). These additional data will be applied in calculating more complete carbon emissions of 2023 onwards and were added in retrospective to the emissions from 2015 to 2022.

Currently, data storage and data centres are not included in the Scope 3 emissions. The UG operates its own data centres which are not included in the calculations. The university is further outsourcing much data at Google^©^, which is running on 100% renewable energy since 2016 equalling to zero emissions.^96^

Most of the carbon emissions of a university are connected to its educational- and research-practices in natural sciences (between 52 and 70%,^43,56^ see Section 2 Environmental Impact of Laboratories). At UG these are located at the Faculty of Science and Engineering covering all research related to science, technology, engineering, and mathematics (STEM) with disciplines such as astronomy, biology, chemistry, chemical engineering, computational science, life sciences, maths, materials science, pharmacy, physics, and more.

In Table 1 the total CO_2_ emissions of the UG in the years from 2017 to 2022 are put in perspective. For 2019 most of the carbon footprint of the university is Scope 3 emissions (72.2%, when electricity offsetting is included), which is mostly generated through research practices in STEM.^97^ In fact, the carbon impact of FSE corresponds to 43–53% of the total CO_2_e emissions of the whole university. The annual number of publications in the field of STEM are tracked through the Nature Index, which corresponds to 390 publications by FSE in 2019.^98^ Thus at FSE per publication 68 tons of CO_2_ are emitted, equalling to annual emissions of about 9 tons per person (2889) and 0.19 tons per m^2^. The carbon emissions of the authors of this article are depicted in Table 1 in further detail since the start of their academic career. While CO_2_ emissions scale with seniority as publications per year increase, all authors drastically exceed the annual carbon emissions per capita in Ethiopia, the annual emissions per car, and the Paris aligned annual carbon budget to limit temperature increase to 1.5 °C. The Paris aligned annual carbon budget is exceeded by T. Freese by a factor of 14×, by N. Elzinga by a factor of 2×, by M. Heinemann by 25×, by M. M. Lerch by 16× and by B. L. Feringa by 216×.

CO_2_ equivalent emissions of laboratories and research practices in the past years, and comparison of researchers and climate goals. Publications are used as measurable output of FSE, whereas universities provide further output as number of alumni and education. FSE emissions of 2019 were used as basis for the calculations of the authors, except for the academic career of Nils Elzinga (2022)

**Table:** 

Emissions at the University of Groningen and Faculty of Science and Engineering in perspective						
	2017		2019		2022	
	w/o offset	w/o offset	w/o offset	w/o offset	w/o offset	w/o offset
Total CO_2_e emissions (UG)	64 389 tons	49 213 tons	61 829 tons	32 275 tons	48 362 tons	20 417 tons
Scope 1	13.4%	17.5%	13.1%	25.0%	15.1%	35.8%
Scope 2	48.5%	32.6%	49.1%	2.5%	59.0%	2.8%
Scope 3	38.1%	49.8%	37.8%	72.5%	25.9%	61.4%
Total CO_2_e emissions (FSE)	28 255 tons	22 978 tons	26 642 tons	16 700 tons	25 419 tons	15 996 tons
Scope 1	17.8%	21.8%	16.8%	26.7%	16.7%	26.5%
Scope 2	38.4%	24.3%	38.3%	1.6%	37.8%	1.7%
Scope 3	43.8%	53.9%	44.9%	71.6%	45.5%	72.3%
# Of publications (FSE)^a^	372		390		498	
Emissions per publication	76 tons	62 tons	68 tons	43 tons	51 tons	32 tons
Emissions per person^b^	10 tons	8 tons	9 tons	6 tons	9 tons	6 tons
Emissions per m^2^^b^	0.20 tons	0.16 tons	0.19 tons	0.12 tons	0.18 tons	0.11 tons

a Source: Nature Index (https://www.nature.com/nature-index/).

b Calculation per staff of FSE (2889) as support staff is needed to maintain laboratories. Area: 140 782 m^2^.

c The calculation was conducted through the emissions per publication of 2019 and correlation with the average number of coauthors, which were calculated from the 20–40 most cited, and 20–50 most recent articles of each author.

d Source: https://www.atmosfair.de/en/and IPCC report.

**Table:** 

Authors' research related CO_2_ equivalent emissions in perspective (excluding personal emissions)						
Researcher	Tracked academic career	Number of publications	Average # of coauthors	Estimated CO_2_e emissions (tons)^c^	Publications per year	Annual CO_2_e emissions (tons)^c^
Thomas Freese	2020–2023	8	8.6	63	2.7	21
Nils Elzinga	2023–2023	1	14.0	4	1	4
Matthias Heinemann	2002–2023	100	8.6	792	4.8	38
Michael M. Lerch	2014–2023	22	6.8	221	2.4	24
Ben L. Feringa	1976–2023	1186	5.3	15 258	25.2	324
Annual emissions per capita in Ethiopia^d^						0.56
Annual emissions per car (12 000 km; middle class model)^d^						2
Paris aligned annual carbon budget (1.5 °C)^d^						1.5

### 3.3 Energy and electricity

As previously stated, since 2018 the CO_2_ emissions relating to electricity dropped significantly as green energy certificates are acquired (53 126 001–57 811 385 kW h). Further parts of the electricity at UG are geothermically self-produced (16 000 000 kW h annually) and 2% of the total energy use is self-produced through solar energy (1 385 250 kW h). This amount of geothermically self-produced energy is not even sufficient to cover the energy consumption of FSE for one whole month per year Fig. 2E. Generally, though, a trend is visible at FSE, that energy consumption is decreasing over the years from 2019 to 2023, with 2023 having the least energy consumption (from 23 394 175 kW h in 2019 to 20 800 040 kW h in 2023). While energy prices, media and awareness boosted sustainable energy measures within the UG and faculty, the Green Lab group has also made great progress since 2021 contributing to these improved values. As already mentioned, laboratories consume much more energy than other areas of the university, thus these efforts are taking effect on the annual energy consumption of FSE. Currently, the electricity usage at FSE in 1 hour is equivalent to the usage of 1 average Dutch family in a whole year, Fig. 2F.

Not included in the Scope 3 emissions are currently outsourced sustainable data storage at Google^©^. The UG currently uses 851 236.65 GB (17.10.23 at 9 am), which include stored emails by the authors: 2429 by T. Freese, 4002 by N. Elzinga, 791 by M. Heinemann, 30 129 by M. M. Lerch, 127 000 by B. L. Feringa (up to 26 g CO_2_e per email).^36^ Another fact that should be improved in the future is the carbon emissions of the website https://www.rug.nl/. The carbon emissions are related to the data transfer over wire, energy intensity of web data, energy source used by the data centre, carbon intensity of electricity and website traffic. Calculation of the carbon results for the homepage of the University of Groningen results in an energy rating F (https://www.websitecarbon.com/website/rug-nl/), corresponding to being worse than 87% of all web pages globally. Every time someone visits the webpage 1.81 g of CO_2_ is emitted. Over a year with 10 000 monthly page views, https://www.rug.nl/ produces 217.62 kg of CO_2_e using 492 kW h of energy.^99^

Efforts by the Green Lab team at the UG led to the measurements of energy consumption of several laboratory devices and equipment. The most energy consumption in laboratories is caused by fume hoods and air ventilation. Measurements at FSE by the Green Lab team revealed that flowrates are reduced from 600 m^3^ h^−1^ to 200 m^3^ h^−1^ by closing the sash, allowing for less energy required to supply input air.^100^ Since the implementation of sustainable laboratory efforts 46 freezers were increased in temperature from −80 °C to −70 °C, which saves 81 030 kW h annually equalling to the energy consumption of 27–32 Dutch households. Reducing the acceleration voltages of transmission electron microscopes while being idle (*i.e.*, not in use or overnight) results in 40% less energy consumption. One rotary evaporator consumes 1198 kW h per year, which can be reduced by 56% to 531 kW h by covering the water baths with hollow polypropylene balls.^100^ Oil baths are frequently utilized for conducting experiments on stirring plates at elevated temperatures. Replacing these oil baths with metal heating blocks reduced their energy consumption by 21%. We further conducted energy measurements on all devices in several individual laboratories (*i.e.*, excluding air ventilation). The whole energy mix consisted of 48% energy usage by two ULT-freezers at −80 °C, 9% by a vacuum concentrator with vapor trap and pump, 8% by one ULT-freezer at −70 °C, 4% by an oven running at 100 °C, and the remaining 31% consisting of several freezers, fridges, ice machines or incubators (Table S38† ESI). A complete list of energy consumption measurements on laboratory devices (balances, ultrasonic baths, vortex, pH meters, oven, freezers, fridges, LEDs and lamps, rotary evaporators, orbital mixer, air conditioning, stirring plates) is reported in Table S37 ESI.†

### 3.4 Travel

**Fig. 3:**
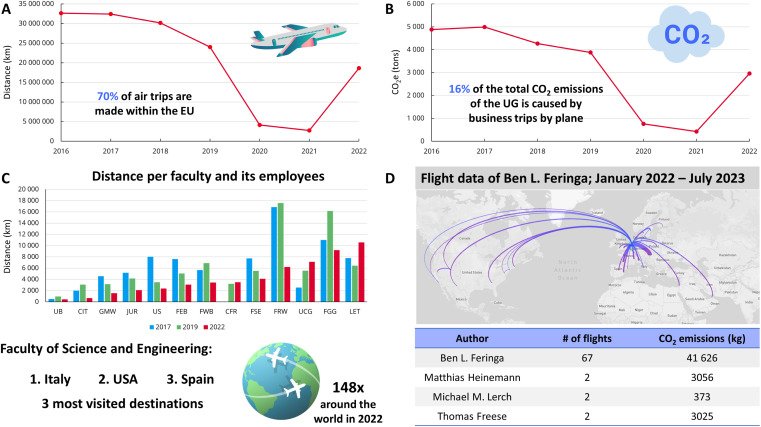
Travel related data and emissions of UG, FSE and authors. (A) Total distance of business travel of the University of Groningen. (B) Total CO_2_ emissions correlated with business travel of the University of Groningen. (C) Total distance of business travel per faculty from 2017 to 2022, where FSE is accounting for the highest business-related emissions. (D) Flight data and emissions of the authors of the article from February 2022 until July 2023.

Data of business travel of the UG have been recorded in annual reports by the Green Office and a travel agent since 2016 containing the departure and destination of flights and the total distance of the flight. Fig. 3A and B depict the total flight distance and CO_2_ emissions of all flights between 2016 and 2022 combined. Clearly visible is the significant decrease in flights in the years 2020–2021, which is a direct consequence of the global COVID-19 pandemic. Interestingly, 70% of air trips of the University of Groningen, Netherlands are made within the European Union (EU) and 16% of the total CO_2_ emissions of the UG are caused by business trips by plane. Here short flights (<800 km) cause 9.4% of the total emissions, whereas long flights (>2500 km) contribute to 68.2% of total emissions.

In 2019, the University launched a new business travel policy, stating that business travel can no longer be done by airplane to and from locations which are within a distance of 500 km and/or can be reached by train within 6 hours. In 2022, this policy was updated to a distance of 800 km and/or 9 hours of travel time. This new, extended travel policy eliminated 24.6% of the short-distance flights, and 6.5% of the total number of flights. On average, this new policy has led to a reduction of 2.5% of the total CO_2_ emissions of business travel within the University.

Fig. 3C depicts the distance of flights of the UG divided per faculty and its corresponding employees (FTE). Generally, the Faculty of Science and Engineering (FSE) is since 2017 until 2022 by far the biggest contributor to the total of the university's business travel (11 000 000 km in 2017, 8 000 000 km in 2019, 5 900 000 km in 2022, Fig. S9 ESI†). However, when correlated with the number of employees (2889) it performs well with an average distance of 4100 km in 2022. The 3 most visited destinations are Italy, USA, and Spain respectively and the total flight distance of 2022 corresponds to flying 148× around the planet earth. Under the “*FSE goes green*” program a working group ‘travel behaviour of staff’ has looked specifically into ways to reduce greenhouse gas emissions due to business travel, in particular by airplane. The target reduction is 30% by 2026, in line with the UG sustainability goals. In November 2022, a survey was conducted among all 2889 staff employed by FSE institutes with 362 valid responses, asking about travel behaviour, use of travel agency portal, online *vs.* physical meetings, awareness about UG mobility policies, and opinions on additional measures for reducing CO_2_ emissions connected with UG travels. Here two policies to reduce CO_2_ emission due to business travel were found to be the most acceptable: (1) tracking the annual carbon emission for each institute, and (2) imposing a climate contribution for airplane trips, which is used to subsidize train trips. Further conclusions were closing loopholes in existing policies, making current and planned policies on short-haul flight avoidance well known among staff, encouraging and facilitating online meetings, addressing staff questions and concerns about policies, and making the booking of international train trips easier.

We further gathered business travel data of the authors in the post-pandemic period of February 2022–July 2023. Here flight data cover invited talks, presentations, business meetings abroad and conferences. We already established that air travel increased back to pre-pandemic times from 2022 onwards, thus our data could also be representative for the years 2016–2019. The authors of this article are in different stages of their academic career: T. Freese (PhD candidate), M. M. Lerch (assistant professor), M. Heinemann (full professor), B. L. Feringa (senior professor, Nobel Laureate in Chemistry 2016). Generally, we observed that the number of flights and emissions increased with seniority: M. M. Lerch with 373 kg CO_2_e emissions in 19 months has the lowest carbon footprint, with the same number of flights as T. Freese (3025 kg) and M. Heinemann (3056 kg) as CO_2_ emissions are correlated with flight distances. Due to prominence, international recognition, duties and demand the carbon impact of B. L. Feringa is most probably one of the highest of staff at the University of Groningen with 67 flights and 41 626 kg CO_2_e emissions, thus flying 3.5× per month with CO_2_e emissions of 2191 kg on average. Hence B. L. Feringa's annual travel related footprint equals 26 290 kg CO_2_e emissions (42 flights per year). Interestingly, when correlated with the 324 tons of total CO_2_e emissions calculated previously (Table 1), B. L. Feringa's air travel corresponds to 8.11% of his total emissions. However, especially for scientists that are advocates for sustainability, educating audiences about circular, sustainable, or green research findings, there is most probably an optimum between the number of flights taken, their CO_2_ emissions and the CO_2_ emissions avoided by presenting their newest findings.^82^ Generally, the further researchers progress throughout their career, the more CO_2_ emissions are generated through air travel annually.

### 3.5 Waste

At the UG, all waste is weighed at the building level by the collector. Plastic and residual waste is weighed through compactors and the UG obtains the figures from the waste processor. The University of Groningen's waste policy was revised in 2019. From March 2021, the RUG started to separate waste at the source. Thus, not only post-separation is conducted, but the UG environment is also set up for waste separation by students and staff, utilizing waste islands. Residual waste is further separated into the raw material flows: coffee cups (no longer issued as of 2024), paper, PMD (plastics, metals, drinks), organic waste and a small residual waste fraction. PMD and residual waste are post-separated at the same waste processor. The amount of non-hazardous waste decreased by about 30% compared to previous years. The graph shows the amount of waste in kg per employee + student, see Fig. 2C.

Currently, a European tender for waste management is being organized. The UG's targets for 2025 included in the UG new waste policy are: 95% total waste separation (hazardous and non-hazardous) by 2025 and 15% reduction in the total amount of waste compared to 2020 (from 17 kg to 14 kg per person (staff + students), Fig. 2C). This means that UG is striving to make all non-hazardous waste circular by 2025.

Non-hazardous waste will be re-tendered in 2025. The Schedule of Requirements will include the sustainable processing of waste and a goal of fossil-free waste collection transport from 2023. From 2026, only emission-free logistics will be permitted in the city centre of Groningen and for all UG locations. This is in line with the mobility policy of the municipality of Groningen, whereby logistics in the city centre must be emission-free from 2030 onwards. The UG only works with local companies to recycle or process the separated waste.

#### 3.5.1 Non-hazardous waste

Since March 2021 waste has been separated at the source through waste bin islands. Waste is separated into five types of waste flows: paper, plastics, organic food waste, other waste, and cups. The waste bins have different colours designating the different types of waste and are emptied regularly.

Paper is collected in blue bins and then recycled by PreZero^©^. They wash the paper and break it down to pulp, which is subsequently pressed into new paper or used to manufacture toilet paper. The same process is applied to used paper cups.

Plastic and residual waste goes to Attero^©^, which puts it through subsequent separation. Separated mono-plastic streams are then recycled by melting and remoulding into new products, where possible. Such recycled plastic is suitable for the production of new bottles, among other uses. Renewi^©^ processes all food and organic waste. This type of waste is used to produce compost, a natural plant fertiliser.

Other residual waste and non-recyclable plastic waste are subjected to subsequent separation by Attero^©^. Residual waste can be processed through incineration. Waste incineration generates energy, which is captured and reused. Some of the residue that is left after incineration can be used in road construction. However, as of January 1st 2024, a new legislation was applied in the EU affecting UG staff, students and visitors: disposable cups and containers are no longer issued in UG buildings and canteens. The new standard is ‘*Bring your own*’. If visitors are at UG, it is possible to buy a reusable cup near the coffee machines for €1.

PhD candidates who defend a thesis at the Faculty of Science and Engineering (FSE) can be reimbursed for the printing costs, where the University and the FSE provide this reimbursement. As of February 1st 2024, the reimbursement for printing costs for a PhD thesis was decreased to 750 Euro from the previous 1600 Euro for each PhD candidate. It was observed that PhD candidates often overestimated the number of hard copies of their thesis. As a result, many theses ended up, unopened and unread, in the paper waste. Hence, decreasing the number of theses printed through decreasing reimbursement contributed to making the faculty more sustainable. For general printing the university uses Canon^©^ printers and paper, who pledges that the wood comes from sustainably managed forests and those emissions caused (*e.g.*, transport) are offset by supporting global projects carried out by ClimatePartner^©^.^101^

#### 3.5.2 Hazardous waste

Hazardous waste produced at UG's laboratories is collected separately. At the FSE orders for consumables (*e.g.*, gloves, pipette tips, other single-use consumables, or general laboratory glassware) are combined institute-wide to reduce waste and duplicates. The Stratingh Institute for Chemistry operates such a communal storage area with an additional in-house store to buy and sell frequent laboratory consumables directly, coordinating institute-wide ordering. Accordingly, packaging waste, logistics and transport are minimized where individual laboratories restock directly at such a warehouse.

**Fig. 4:**
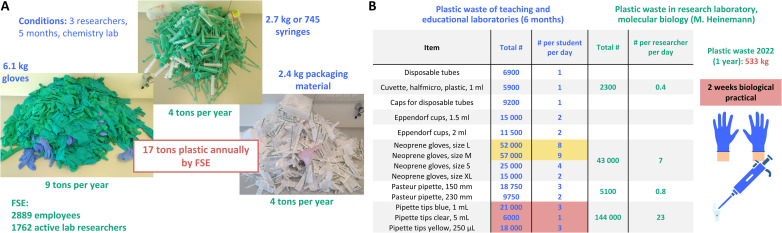
Plastic waste production at FSE, student education and research laboratories. (A) Plastic waste production in a chemistry laboratory (3 researchers in 5 months, blue) and extrapolation to the whole Faculty of Science and Engineering for its annual plastic waste production (green and red). The estimation of plastic waste through a model chemical laboratory becomes accurate as biology/life sciences plastic waste production is underestimated and the production of dry labs (physics, computation science *etc.*) is overestimated. (B) Plastic waste of teaching and educational laboratories of 6 months (left) and the annual plastic waste production of a molecular biology laboratory (right). Typical plastics were gloves (neoprene (green) and nitrile (blue)), syringes (cylinder (PP), plunger (PE)), packaging material waste (PP, PE), pipettes (PE and PS), and pipette tips (PP).

As mentioned, laboratories are located at the Faculty of Science and Engineering focusing on STEM research. In the beginning of 2022, the Green Labs team conducted a case study in a chemistry lab for 5 months involving three representative researchers, where all plastic waste was collected separately. During that time 6.1 kg of gloves, 2.7 kg of syringes and 2.5 kg of packaging material waste was produced (Fig. 4A). These data were extrapolated to 1762 active lab researchers of the 2889 employees of FSE for its annual plastic waste production. According to this extrapolation, roughly 9 tons of glove waste, 4 tons of syringe waste and 4 tons of packaging material waste are produced annually. Thus, at the faculty 1 367 794 individual gloves are used per year (6.59 g per glove). Taken together the laboratories at the Faculty of Science and Engineering produce 17 tons of plastic waste annually. The estimation of plastic waste through this model chemical laboratory becomes accurate as biology/life sciences plastic waste production is underestimated and the production of dry labs (physics, computational science *etc.*) is overestimated.

We further gathered data from student practical and educational labs for bachelor’s and master’s students studying at FSE. A typical course for chemical education (Synthesis 1) is running for 15 days with about 130 students. The plastic waste as well as the chemical waste production of student education was tracked for one semester of 6 months (Fig. 4B and ESI†). The data in Fig. 4B indicate the drastic numbers of plastic waste production covering disposable tubes, cuvettes, caps, Eppendorf^©^ cups, gloves, pipettes, and pipette tips. Here neoprene gloves in sizes L and M (52 000 and 57 000, respectively) cover a vast amount of plastic waste. Pipette tips are only used in 2 weeks of biological practical and are producing waste of a total of 45 000.

When compared to the total plastic waste production of a research laboratory focusing on molecular biology (Heinemann research group, Fig. 4B), these values can be put more into perspective: in 2022 the group of M. Heinemann produced 533 kg of plastic waste in total. In a biochemistry lab, where most activities involve molecular experiments using *Saccharomyces cerevisiae* and *Escherichia coli*, all types of plastics were weighed and summed up based on the number of orders placed over a year. We calculated that a lab consisting of 17 active lab members uses 533 kg of plastic consumables per year. This is 2.6 kg of tubes, tips, plates, syringes, and other plastics per person per month (31.3 kg per person per year). Residents in the EU produce an average of 34.6 kilogram of plastic waste annually, indicating that the researchers double their annual plastic impact through research activities.^102^ Going back to the plastic waste production of a chemical laboratory, the numbers from Fig. 4A can be calculated to 0.8 kg of gloves, syringes and packaging material per person per month, thus 9.4 kg of plastic waste production per person per year. Hence chemical research roughly produced a third of plastic waste when compared to biological research.

The biochemical research of the Heinemann group (17 members) produced a waste amount of 43 000 individual gloves in 2022, equalling to 7 gloves per person per day. Student education however corresponded to 298 000 gloves per year (149 000 gloves, 6 months), which equals to 23 gloves per student per day. These amounts are 6.9× times higher than the annual glove usage in a research group. While the Heinemann research group used 144 000 pipette tips in 2022, the student education with their 2 weeks biological practical produced a total of 45 000 in two weeks, which equals to 90 000 pipette tips per year if extrapolated (7 pipette tips per student per day). These values clearly indicate that sustainable laboratory practices such as switching to reusable glass alternatives are having a drastic and direct effect on student education, not only *via* mindset for a paradigm shift, but also on waste production and related hazardous waste costs.

**Fig. 5:**
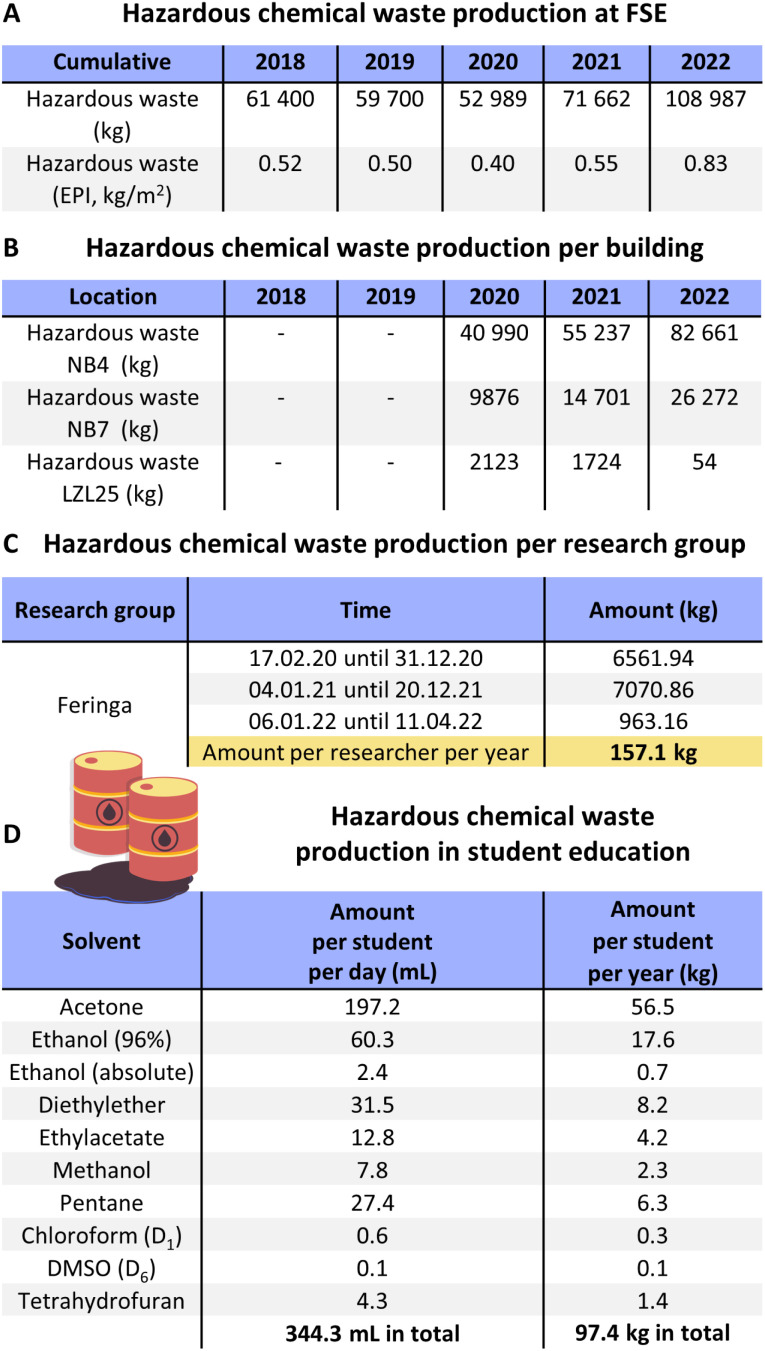
Hazardous chemical waste production at the Faculty of Science and Engineering, UG. (A) Cumulative and (B) separated per building, where NB4 covers chemistry, chemical engineering, student education, physics, and materials science. NB7 covers biology, life sciences, animal science, chemical biology, and industrial chemistry. Where biology laboratories produce more plastic waste than chemical laboratories, chemical research produces more hazardous chemical waste than life sciences. (C) Chemical waste production per research group; Feringa is mostly located in NB4. (D) Chemical waste production per student per day and year in practical courses for student education.

The Faculty of Science and Engineering also produces hazardous chemical waste as disposed solvents, liquids, or hazardous solid waste (solid chemicals or contaminated single use consumables). In Fig. 5 the hazardous chemical waste production is depicted. The amount of commercial and hazardous waste is expressed in Fig. 5A in total amount of kg and in EPI (kg per m^2^ of floor area). The cumulative scores for waste are provided for all the buildings managed by FSE (Nijenborgh 4 (NB4), Bernoulliborg, Energy Academy Europe, Location Zernikelaan 25 and Linnaeusborg (LB and NB7)) with a total floor area of 131 302 m^2^. Fig. 5B depicts the waste production per building, where NB4 covers the Stratingh Institute for Chemistry, the Engineering and Technology Institute Groningen (ENTEG), the Zernike Institute for Advanced Materials (ZIAM) and parts of the Groningen Biomolecular Sciences and Biotechnology Institute (GBB). In NB7 most of the research of GBB is taking place as well as all research of the Groningen Institute for Evolutionary Life Sciences (GELIFES). Some laboratories of the Stratingh Institute for Chemistry are also located at NB7. Generally, it can be assumed that most chemical research is taking place at NB4, whereas most biological research takes place at NB7 (with some cross-sections of laboratories). Through the STEM research taking place at FSE the annual hazardous chemical waste production has been increasing since 2018 from 61 400 kg to 108 987 kg. Here especially NB4 and NB7 are the main production locations for chemical waste, where since 2020 all numbers have been increasing. The chemical waste of the Stratingh Institute for Chemistry (150 staff members) increased from 40 990 kg in 2020 to 82 661 kg in 2022. Even during the COVID-19 pandemic in 2020 and 2021 the amount of chemical waste increased with lockdown measures in place. The same trend is visible in NB7, where the numbers increased from 9876 kg (2020) to 26 272 kg (2022). Thus, in both NB4 and NB7, the amount of hazardous waste has increased significantly. A major cause is the fact that since the beginning of 2021, the Health, Safety and Environment (HSE) department has initiated a major clean-up operation to dispose old chemicals, which is still being continued as of the time of writing. This operation started in NB4 in 2021 and in the beginning of 2022 also in the Linnaeusborg NB7, and it probably will continue until mid-2024, the reason being the already mentioned construction of the new building (Feringa Building) and the correlated move of the laboratories, where old, unused, and dangerous chemicals are being disposed of and not taken to the new facilities. In the LB, renovation of upper floors has been completed in 2021 and an organic chemical group has moved in, explaining the increase in hazardous waste in the LB.

After covering the chemical waste production of the faculty (Fig. 5A), with accuracy to each department and building (Fig. 5B), it is possible to zoom further into the research groups of the authors: Fig. 5C covers the chemical waste production per research group, where the Feringa group is located in both NB4 and NB7. In 2021 the Feringa group was home to 45 active researchers in the laboratories. The same year the Stratingh Institute at NB4 and NB7 produced a total of 69 938 kg of chemical waste, of which the Feringa group caused 7071 kg, equalling to 10% of the total waste production. With 45 laboratory members, the total hazardous chemical waste production of 7071 kg corresponds to 157 kg per person per year.

The previously introduced student education laboratories are also located at NB4. There the chemical and solvent waste production can also be used to give further perspective to these numbers (Fig. 5D): one student uses 72 L acetone (56 kg), 22 L of ethanol (96%, 18 kg), 12 L of diethyl ether (8 kg) and 10 L of pentane (6 kg) per year. In contrast to plastic waste production when compared to biological research, student education produces only about 62% hazardous chemical waste per person per year when compared to research laboratories (97 kg *vs.* 157 kg per person annually).

### 3.6 Progress and ongoing projects on green labs

**Fig. 6:**
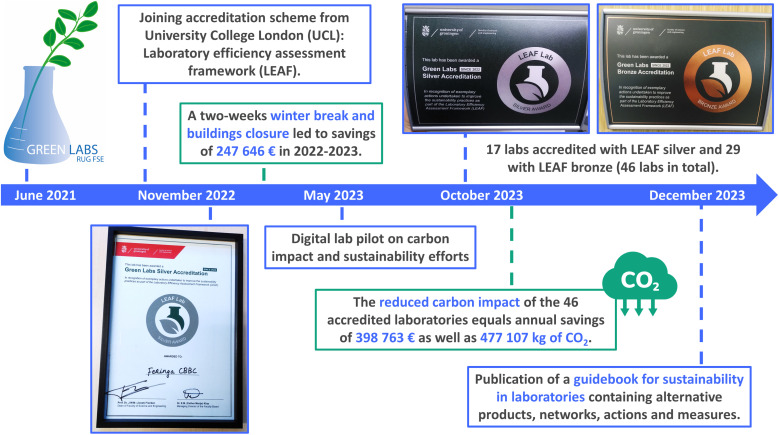
Our path to greener labs. Chronology of improvements to laboratory efficiency at UG and FSE, the reduced carbon impact and annual savings.

The Green Labs team at the Faculty of Science and Engineering of the University of Groningen started as a grassroots movement in June 2021, where like-minded researchers gathered and started to get informed about sustainable actions in the laboratories (Fig. 6). The team started to work on a guidebook, which would become a collection of easy-to-implement actions one could undertake in laboratories to save money, decrease the amount of waste, and reduce CO_2_e emissions. This guidebook was distributed and presented among all principal investigators of the Stratingh Institute for Chemistry and then further distributed through an open meeting at the Faculty of Science and Engineering, at which point further enthusiastic members were recruited. Throughout the two years, this guidebook (https://doi.org/10.26434/chemrxiv-2023-g3lmq-v4) became a comprehensive collection of alternative products, existing lab sustainability networks, hands-on advice, in detail examples, posters and stickers for printing, and recommendations.^100^ Since the beginning the work of the Green Lab team was directly supported by the Green Office of the UG as the impact of sustainable laboratories directly benefits the aims and goals of their roadmap. From May 2022 to December 2022 the Green Lab RUG group became a well-organized team and network of researchers, staff, students, and PIs. Tasks were distributed evenly, and the members formed and joined subgroups depending on their preference for topics.

At the “Stratingh Day” in June 2022, which is an institute-wide annual celebration and team building event, the Green Lab group, and external speakers on laboratory efficiency from Green Labs NL were invited to educate the whole institute of the importance and upcoming intentions to improve laboratory sustainability. During the same time, members from the Green Lab team joined several (online-)conferences organized by Green Labs NL, the Sustainable European Laboratories Network (SELs) and Green Labs Austria. There, more information was gathered as well as international support, evidence, resources, and network allowing for further growth of the grassroots movement. In the summer of 2022, the FSE launched the “*FSE is going green*” program, a framework through which funding became available and a managing board was established. In that board the Green Lab team was invited for a permanent seat. With funding for sustainable actions now being available, it was agreed to join the Laboratory Efficiency Assessment Framework (LEAF) in October 2022 after sufficient evidence for its importance was presented.

The Green Lab team established one of its members as a LEAF coordinator (T. Freese), who contacted founder M. Farley and the University College London to join the accreditation framework. The group received a training on the software as well as auditing and ran a pilot among the 6 active laboratories of the original Green Team members. In November 2022 the first two laboratories achieved silver accreditation (Feringa and Lerch group), whereas 4 additional laboratories successfully passed an audit for reaching the bronze level (Fig. 6). Together with staff from FSE and Green Lab members official door signs and awards were created under the banner of University of Groningen, recognizing its official accreditation. At an award ceremony in February 2023, the managing director of the Faculty of Science and Engineering, together with the dean, recognized and appreciated these efforts and awarded the 6 laboratories personally with their respective awards. To these award ceremonies all staff of FSE were invited to further expand the momentum and LEAF framework through QR-codes and sign-up documents. Through regular engagement *via* faculty and institute newsletters, regularly highlighting the progress and efforts of the Green Lab team, a steady growth of LEAF participants as well as Green Labs was achieved. With every expansion to new laboratories and institutes new interested members joined the core group and subgroups of Green Labs RUG, achieving continuity when staff or students left the university. This continuity is a crucial aspect of the success of a grassroots movement, to include PIs and staff with permanent contracts as soon as possible to achieve a steady foundation of a group.

After the first award ceremony the LEAF engagement grew and hence a team of “LEAF administrators” was assembled to increase the efficiency of laboratory audits. While dates, lengths, details and how to conduct an audit were organized through the LEAF coordinator, the LEAF administrator team became an important group of well-educated researchers and technicians on laboratory efficiency. As more laboratories were joining the framework official, unified stickers and posters were printed to close fume hoods when not in use or turn-off equipment, which were distributed among signed up laboratories. The design of the stickers was coordinated again with the Green Office together with Green Lab members, and funding achieved through the “*FSE goes green*” program.

Another major achievement was the inclusion of all student education laboratories for chemical, pharmaceutical and biological research (wet laboratories). Through support by the coordinator, lecturers and teaching assistants measured plastic and chemical waste production in student laboratories, organized the distribution of stickers and posters to turn off equipment and took part actively in auditing other laboratories. Rapidly, all student laboratories were working towards bronze and later silver accreditation. This was only possible because the *12 Principles of Green Chemistry* were already a core part of the bachelors and masters curriculum at the University of Groningen.^103^ With the successful silver accreditation of these 15 educational laboratories in October 2023, bachelor’s and master’s students are directly familiarized with concepts of sustainable laboratories. Perceiving this way of conducting experiments as standard, these students will automatically carry forward a more sustainable approach when joining research groups in the future for their research projects and or PhD theses, this way automatically expanding the Green Lab and LEAF framework into every research laboratory of the faculty. Another advantage is that teaching assistants in those educational labs are current PhD candidates, fulfilling their required teaching duty. As part of their preparation and training, these PhD candidates are educated about the sustainable goals, content and practices that need to be transferred as knowledge to the students. PhD candidates from research groups that are not familiar with sustainable laboratory practices or LEAF yet are automatically joining a lab for a certain amount of time, which is accredited with LEAF silver, then learning and applying those rules to transfer those to the students present in that laboratory. After the course is finished these PhD candidates continue with their own research in their respective research groups and may want to improve their laboratory practices within their own research group as well.

The efforts of the Green Lab team were further expanded and piloted from May 2023 until November 2023: the digital and dry labs of the faculty were included to assess their carbon impact and sustainability efforts, which included computational science, artificial intelligence, and mathematics. Two PIs of these research fields joined the Green Lab RUG team, and one was assigned as the coordinator of dry lab efficiency. Together with a local team of five (including the LEAF coordinator), an international pilot was completed to establish an accreditation framework with categories covering bronze, silver, and gold for dry labs specifically. All learnings, findings and recommendations were published in the guidebook of the Green Lab RUG team and the first dry lab achieved bronze in October 2023.

The Green Lab and LEAF engagement was recently able to include the activities of several institutes of FSE covering disciplines such as biology, chemistry, chemical engineering, computational sciences, life sciences or pharmacy. These efforts resulted ultimately in 46 accredited laboratories in October 2023, 17 of which achieved LEAF silver and 29 reached the LEAF bronze level (including disciplines such as chemistry, chemical engineering, biology, life sciences, pharmacy). At another award ceremony (which always includes social gatherings for networking and outreach), an art exhibition was organized. Here the hidden waste of research was put in focus, where staff and artists were able to exhibit pieces highlighting the impact of laboratories on the planet. The plastic waste collected from the chemical laboratory (Fig. 4) was gathered to be displayed as art resembling a big syringe or a flower made from gloves (2.7.8 ESI†). Drawing attention to glass waste, art pieces made from laboratory glass waste were displayed next to a large number of discarded, clean and unused laboratory coats. During Summer 2023 a movie (https://www.youtube.com/watch?v=Zk_CEmyHZZg) was made to highlight the environmental impact of laboratories to be used in social media and outreach, which was also shown and distributed since at the award ceremony, which took place during the Sustainability week organized by the Green Office RUG. There again, the dean of the Faculty of Science and Engineering awarded each laboratory with their respective award and all photos were shared in a news article of FSE, UG, and the Stratingh Institute afterwards. Finally, in February 2024 the efforts of the Green Lab team and LEAF were expanded to the University Medical Centre Groningen (UMCG) to improve laboratory practices in hospitals.^104^

Next to LEAF, several other subgroups achieved further successes: funding for the replacement of inefficient laboratory equipment was administered through “*FSE goes green*”, which led to faculty wide energy measurements and applications. Already in 2022, the Green Lab team started to work on ULT-freezers, which included measurement of energy consumption for all −80 °C freezers of the faculty (52 freezers operated and 46 measured). Subsequently, their temperature was increased to −70 °C (40–46 freezers increased in temperature, savings of 81 030 kW h annually). Freezers with undesired energy consumption (21) and age were first checked for their maintenance, and if sufficient replaced with energy friendly and newer models (6). Increasing freezer temperatures and replacing inefficient ones were further reported and submitted to the 2023 Freezer Challenge^105^ by My Green Lab^©^, which ultimately led to a total of 20.7 million kW h of energy saved and thus avoiding 14 663 tons of CO_2_e. In 2023 more than 2000 laboratories participated and since the launch of the challenge 44.7 million kW h worth of energy equalling CO_2_ emissions of 31 678 tons was saved, which corresponds to the energy consumed by roughly 6164 homes in one year. In 2023 more than 26 000 cold storage units and 170 organizations joined the efforts.^106^

Because laboratory buildings consume large amounts of energy, an annual winter break and building closure was implemented at the end of 2021. For financial and environmental reasons, all buildings of the faculty were closed for a full two weeks and all laboratory equipment, computers, office equipment, machines and devices were turned off and unplugged, if possible to do so, to reduce energy consumption. Heating was reduced and flow rates of fume hoods reduced or turned off. In the break of 2022–2023 the energy savings were further accelerated through communication and engagement by the Green Lab team: in the years from 2018 to 2021 the total energy consumption at FSE was on average 1 019 461 kW h of electricity and 160 489 m^3^ of gas during a two-week winter break, which is already much lower when compared to the average monthly consumption at FSE (2.7.4 ESI†). However, with Green Labs RUG we were able to further increase these efforts in the break of 2022–2023 with improved energy consumption of 798 977 kW h electricity and 102 333 m^3^ of gas, generating additional savings of 221 kW h and 58 m^3^ when compared to previous years, corresponding to additional cost savings of 117 959 € and 129 687 € respectively. In the break of 2023–2024 measures such as turning off 8 out of 12 fume hoods per laboratory in old buildings such as NB4 resulted in drastic additional savings of at least 48 000 € of electricity and 200 000 € of saved heating costs. Capitalizing on this success, we are currently investigating possibilities to turn off fume hoods when not in use, if ensured that all containers stored underneath are closed.^25^

In particular, for old buildings energy efficiency could be increased by retrofitting windows with insulation films, which increase heat gain by 55% and reduce heat loss by up to 40%, leading to lower usage of heating and cooling systems and ultimately reduce costs on energy bills. A coordinated effort among the whole NB4 (including the Stratingh Institute and GBB) provided a low-cost solution and quick installation of the films by laboratory researchers.

Another focus of the Green Lab RUG team is the reduction of waste production from laboratories: uncontaminated plastic waste and normal packaging (laboratory packaging plastics) can be disposed of as PD/PMD (plastics, metals, drinks) in regular recycling streams of the University, which is transported to Attero^©^. Also, styrofoam boxes are collected at central locations and recycled through take-back schemes.^107^ All LEAF bronze and higher accredited laboratories conduct these measures.

Gloves can be downcycled to furniture or gardening products through companies such as Terracycle^©^,^108^ but utilizing these schemes results in higher costs than burning as hazardous solid waste with PreZero™: currently, the costs for incineration of gloves are 1.23 € per kg. At FSE, of the 2889 employees, 1762 active lab researchers (1300 PhDs/postdocs/technicians and 462 guest researchers) produce 9 tons of glove waste annually (Fig. 4). Thus incineration would cost 11 080€ per year, but downcycling would lead to additional 36 085 € needed (total of 47 166 € annually per 9 tons transported in 137 boxes of 144 L or 66 kg at a price of 344 € per box).

Take-back schemes to original suppliers and manufacturers should be prioritized in all cases. Comparable, fully circular schemes exist for gloves through, *e.g.*, Gloovy^©^ (https://gloovyecogloves.nl/en) eco gloves, which is currently being piloted at the University Medical Centre Groningen (UMCG) and the Stratingh Institute for Chemistry. Here new gloves are produced from the glove plastic waste that is taken back from the laboratories (if uncontaminated).

Circular take-back schemes also exist for pipette tip boxes, where there are several models available and investigated by the Green Lab team for a university-wide coordination. In the future the goal is to include falcon tubes and pipette tips.

Companies such as Grenova^©^^109^ offer pipette tip dishwashers for washing and reusing pipette tips (25–40 times per tip).^107^ A pilot investigation and calculation by the Green Labs team led to the conclusion that buying a Grenova^©^ pipette tip dishwasher for the educational laboratories of FSE was not feasible due to the high amounts of solvent needed for cleaning (based on ethanol); if water and soap would be utilized it became feasible. Another pilot on autoclaving plastic syringes from a chemical laboratory for reuse in a chemical environment concluded that syringes were not clean enough as residues of chemicals or water were observed which could interfere with chemical reactions (Fig. S27, ESI†).

The waste stream subgroup of our Green Lab team is investigating several aspects to implement circular recycling schemes. Next to the glove pilot together with the UMCG, a take-back scheme pilot program^110^ by Merck^©^ and Sigma-Aldrich^©^ is ongoing.

Currently, a solvent recycling program is being investigated as a pilot program, where the Green Lab team plans to distill and recycle solvents such as acetone, methanol, or ethanol to reduce the amount of liquid hazardous waste.^25^ Previous reports show that these solvents could be reused for cleaning of glassware (and perhaps even making the pipette tip dishwashing feasible in educational laboratories).

Glass and flasks are washed and autoclaved at several locations within the faculty and university. Thus, switching from plastic consumables to glass alternatives is most recommended as it is better than any plastic recycling, as no waste is produced whatsoever.

Starting from March 2024, the Green Labs group implemented a marketplace for second hand laboratory equipment to reduce the number of devices that are disposed of and support groups with less income. Through LabMakelaar (https://www.labmakelaar.com/) devices are refurbished (*i.e.*, repaired, calibrated, and tested for functionality) and then transported to customers (2.7.7, ESI†).

Being in line with the cancellation of all disposable (coffee) cups at UG and their catering services, staff members had the option to obtain a collapsible and thus transportable cup together with a mug as Christmas present in 2023.

From 2024 onwards we are investigating to reduce the energy consumption in office spaces: if all 6390 employees of the UG have at least one computer screen (*i.e.*, monitor) in their office (excluding multiple screens), annual savings of 23 324 € and 86 297 kW h less energy consumption can be achieved if all employees would reduce their screen brightness from 100% to 75% (equivalent to the annual energy consumption of 27–35 Dutch households).

As of March 2024, the construction of the Feringa Building (2.6.1, ESI†) was completed which allowed for a reduced carbon impact associated with laboratory research of the former building (Fig. S10,† ESI). All laboratories were built on the north side of each wing, keeping the impact of sunlight to a minimum. The chemical, biochemical and physics laboratories are flexible and interchangeable as each one can be connected separately to the ventilation, power, and gas supply networks. Further sustainability aspects include:

- Optimal insulation.

- Heat reflective coating (HR) glass.

- 900 m^2^ solar panels (±120 000 W_P_ (watt-peak) of nominal power).

- LED lights in addition to natural daylight.

- Gasless heating.

- Geothermal heating and cooling system with heat pumps.

- Energy saving, automated closing fume hoods.

- 4 courtyards to enhance biodiversity.

Other achievements at FSE were the opening of a canteen (Bernoulli's Bistro, Fig. S14,† ESI) utilizing fresh and local ingredients, creating a completely vegetarian and at least 50% plant-based menu. In addition to a more sustainable menu, reusable cutlery is used, and sustainable packaging materials are prioritized. Plants on tables in the canteen were grown free of pesticides and live in second-hand pots. Other projects from “*FSE goes green*” also aim to improve and preserve biodiversity on the campus grounds (Fig. S15, ESI†).^111^ Currently, the team is further looking into the aspects of implementing more solar panels on the university buildings and parking areas and switching to green energy providers.^112^

Through their time and work for Green Lab RUG, some core members became Green Lab Ambassadors (https://www.mygreenlab.org/ambassador-program.html) expanding their reach beyond their own institute, faculty or university: members of the Green Lab RUG team were invited at international conferences as guest speakers to educate about laboratory sustainability and improvements. Outreach to other universities and institutes is a common aspect of the work of Green Lab members, where several presentations, meetings, newspaper interviews or media outlets are given on a regular basis to educate the scientific community about the relevance of sustainable laboratory practices (2.7.10, ESI†). Here outreach about laboratory sustainability and efficiency goes hand-in-hand with the principles of green and circular chemistry.^113–115^ Within the UG this outreach currently covers initiatives for improved laboratories in the departments of astronomy, physics, and materials science. In December 2023 the work and efforts have been concluded and published in the mentioned guidebook,^100^ which is often recommended together with the movie^116^ during outreach, external meetings or interviews to support future efforts of other universities or companies.

Over the past two years a well-organized Green Lab RUG team was established, which successfully grew out of the “grassroots-stage” into an established organization within the faculty with meetings every 4–6 weeks. Through their work, savings, and success the faculty hired in Summer 2023 a full-time energy and sustainability advisor with laboratory experience to join and support the Green Labs team and their efforts.

The current organogram of the Green Lab RUG team can be found in Fig. S17, ESI.† One member is assigned as chair of the group, who takes part at the “*FSE goes green*” meetings, is the main coordinator and administrator of the group, and contacts the Green Office or other institutes for expansion or support. Then the core Green Lab group consists of 8–10 members (staff, PIs, PhD candidates and students) covering the topics:

- Secretary (minutes, action points, communication).

- Energy, facilities, freezers, and fridges.

- LEAF coordinator.

- SELs and Green Labs NL communication.

- Laboratory waste: single-use plastics and solvents.

- Education, student practical, curriculum.

- Dry labs and computation science.

- Funding.

- Community meetings, award ceremonies, webpage, newsletters, and outreach.

Next to the full-time energy and sustainability advisor, a permanent member of the Green Office of the UG is present at the Green Lab meetings. Almost every core member covering these topics has a team of 5–10 subgroup members (*e.g.*, LEAF coordinator with LEAF administrators), who evaluate and establish solutions for detailed waste problems, conduct LEAF audits, support energy measurements, improve student education, or organize the webpage and award ceremonies.

As of October 2023, the Green Lab RUG team was able to achieve successful accreditation of 46 laboratories; 17 of those laboratories were accredited with silver and 29 with LEAF bronze. According to the calculators of the LEAF software, the reduced carbon impact of those 46 laboratories is equalling annual savings of 398 763 € as well as 477 107 kg of CO_2_ (Fig. 6, 10 372 kg of CO_2_ and 8669 € per lab per year). While these numbers are already very impressive and engaging, the impact of the Green Lab RUG team is even higher as measurements and replacement of inefficient laboratory equipment (50 000 kW h annually, 2.7.3 ESI†), the increase of ULT-freezer temperatures from −80 °C to −70 °C (81 030 kW h annually, Table S36, ESI†), and the savings during the winter closures (247 646 € per year, Table S41, ESI†) are not included in those calculations.

## 4 Guidelines to sustainable laboratory practices

By now it should be evident that the environmental performance of science and laboratories is not optimal. Scientific research is at the centre of creating more sustainable materials or processes and thus should be at the forefront of developing and embedding sustainability into their own practices.^2^ As academic science is funded by public money, there is a social responsibility to operate with the environment and future in mind.^18^ There are several Green Lab examples and initiatives (>146) seeking to address the environmental footprint of research.^2^ Driven by the voluntary efforts of researchers, they are educating other researchers, developing sustainability guidelines and maintaining accreditation frameworks. Without the need to reinvent the wheel, scientists across the planet can join these initiatives, form a grassroots group themselves, gather all the information and recommendations needed and ultimately improve their own research operations. Here accreditation processes and scientific publications can guide the progress more effectively. Importantly, these sustainability considerations are in alignment with other priority areas in the research system, such as reproducibility and open science.^117^

### 4.1 Benefits of Green Labs

One obvious benefit of sustainable laboratory practices is the reduction in energy consumption and carbon footprint.^25^ Another is that they lead to efficient waste management and hazardous waste reduction. Thirdly, a minimization of resource depletion and use of single-use plastics is achieved. Taken together these and the following points ultimately lead to economic benefits and thus cost savings:

- By having standard operating procedures (SOPs) and frameworks to share negative results, there is greater reproducibility and less need for repeating experiments.

- As solvents or chemicals are shared and where possible recycled, there is no need to buy as frequently.

- Sharing equipment and lab spaces reduces the energy consumption and costs.

- Turning off equipment when idle minimizes energy consumption and associated costs.

- Production of less hazardous waste consequently leads to reduction of costs of disposal.

- Switching to reusable glass alternatives over single-use consumables reduces waste and procurement.

It is evident that these changes are reducing the carbon impact of laboratories, but they also lead to considerable savings in research expenses. There are several examples that laboratories save up to 15 800 € per year, shrink their non-chemical waste by more than 95% and reduce the single-use plastics consumption by 69%.^19,52,118^ For instance, 4000 kg of waste produced by seven staff members per year could be reduced down to 130 kg by avoiding single-use plastics and recycling.^19,52^ Hazardous chemical waste was reduced by 23% equalling 300 L per year and the electricity consumption of an institute with a size of 11 000 m^2^ was reduced by 26%.^19^ By recycling solvents such as acetone annual savings of 3527 € were achieved at the University of Colorado, which are further increased by savings on disposal costs for hazardous waste.^119,120^ A program to regularly close fume hoods at Harvard University saves the university 183 000 € annually.^33^ These efforts were expanded by routinely sharing leftover chemicals, equipment and materials through a campus-wide initiative – saving a combined total of 250 000 € per year.^121^ Stanford University operated in 2008 about 2000 −80 °C freezers, which were costing the university between €5.6 and 6.2 million euros per year to operate, thus increasing the temperature to −70 °C resulted in drastic savings.^122^ Some departments saved through bulk purchasing and recycling of solvents another 195 000 € per year.^121^ At the University of Colorado green initiatives provide cost avoidance of 231 000 € per year.^120^ Through our actions at UG we have been able to save 10 372 kg of CO_2_ and 8669 € per lab per year. Thus, implementing ecological awareness into the laboratory can save up to 40% of a researcher's funding over one year.^123^ Environmental sustainability is often thought of as expensive, but by incorporating these strategies less chemicals, paper, energy, or plastics are used.^124^ The savings generated outperform by far the small initial costs of implementing sustainability measures such as buying filters or setting up a recycling scheme. Furthermore, the money saved can be reinvested back into research.

It is worth noting that calling these efforts “sustainable” is a luxury of developed countries and well-funded research institutes. In other regions of the planet, these sustainable efforts have long been standard, as they are proven to be the better economic option. There, researchers conduct their research in the most economical way possible, without calling it sustainable. Visiting researchers from those areas are often surprised by the other, more wasteful, working culture, and scientists can improve their sustainability efforts drastically by learning from other countries' well-established standards.

Reduced resource consumption not only translates to direct cost reduction but also to optimized funding allocation: sustainable laboratories are more likely to receive funding for research projects, as they are optimizing every aspect of their research including carbon footprint, efficiency, and productivity. Operating green labs can make the difference in receiving funding for certain projects and are powerful tools to maximize the impact of proposals.^120^ In fact, more and more funding agencies incorporate sustainability into their assessment criteria, making it necessary to meet those in our research institutes.

### 4.2 Sequential implementation of green lab measures

#### 4.2.1 Get informed

Decide to act as soon as possible and do not wait to be an expert. Scientists often are or become experts in their field by knowing every aspect of their system and all associated literature. However, it is not necessary to become an expert in laboratory sustainability to make a difference. It is better to start now instead of tomorrow, thus obvious and often very simple changes can be implemented as soon as possible with as little effort as possible.^3^ As most actions are already well-established and covered in this and other articles, they can be replicated in any institution without much delay.^1,8,100^ Gather the information needed to convince colleagues and understand the aspects of the environmental impact of laboratory practices. Do not reinvent the wheel but share documents and articles (Table 2). The references of this article as well as Table 2 serve as a comprehensive overview for sustainable laboratories. In particular, the resource entries 1–6 of Table 2 are strongly recommended.

Resources on sustainable laboratories

**Table:** 

Resources
(1) Sustainable laboratories (https://www.rsc.org/policy-evidence-campaigns/environmental-sustainability/sustainability-reports-surveys-and-campaigns/sustainable-laboratories/) (report by the Royal Society of Chemistry)
(2) Wellcome report (https://wellcome.org/reports/advancing-environmentally-sustainable-health-research): advancing environmentally sustainable health research
(3) A guidebook for sustainability in laboratories (https://doi.org/10.26434/chemrxiv-2023-g3lmq-v4)
(4) Allea report (https://allea.org/portfolio-item/towards-climate-sustainability-of-the-academic-system-in-europe-and-beyond/): towards climate sustainability of the academic system in Europe and beyond
(5) CaRe 2021: catalogue of recommendations for sustainability in the Max Planck society https://doi.org/10.17617/1.mpsn.2021.01
Sustainability in science Wiki https://sustainability.wiki.gwdg.de/

**Table:** 

Networks	Region	
Green Your Lab	United States and Global	http://greenyourlab.org/
Sustainable European Laboratories (SELs)	Europe	https://sels-network.org/
Max Planck Sustainability Network	Germany	https://www.nachhaltigkeitsnetzwerk.mpg.de/
Green Labs NL	Netherlands	https://www.greenlabs-nl.eu/
Laboratory Efficiency Action Network (LEAN)	United Kingdom	https://www.lean-science.org/
Green Labs Austria	Austria	https://greenlabsaustria.at/
Sustainable Labs Canada	Canada	https://slcan.ca/
Labos 1point5	France	https://labos1point5.org/
Green Labs Portugal	Portugal	https://greenlabs.pt/
Irish Green Labs	Ireland	https://irishgreenlabs.org/

**Table:** 

Accreditation frameworks and schemes	
Green Impact	https://greenimpact.nus.org.uk/
Laboratory Efficiency Assessment Framework (LEAF)	https://www.ucl.ac.uk/sustainable/leaf-laboratory-efficiency-assessment-framework
My Green Lab certification	https://www.mygreenlab.org/green-lab-certification.html
GreenED Framework for Environmentally Sustainable Emergency Medicine and Health Care	https://greened.rcem.ac.uk/
Framework for building sustainability and green building rating: LEED (Leadership in Energy and Environmental Design)	https://www.usgbc.org/leed

**Table:** 

Non-profit organizations	
International Institute for Sustainable Laboratories (I^2^SL)	https://i2sl.org/
My Green Lab	https://www.mygreenlab.org/
Beyond Benign	https://www.beyondbenign.org/

**Table:** 

Green chemistry	
American Chemical Society and the ACS Green Chemistry Institute	https://www.acs.org/greenchemistry.html
Green Chemistry Teaching and Learning Community (GCTLC)	https://gctlc.org/
NMR impurities of solvents and emerging green solvents	http://www.nmrimpurities.com/

**Table:** 

Dry labs and computational science		
Green Algorithms	Carbon and energy calculator	https://www.green-algorithms.org/

**Table:** 

Other tools and resources		
Labconscious	Open resource database	https://www.labconscious.com/
Laboratory Benchmarking Tool	Carbon and energy calculator	https://lbt.i2sl.org/
GES 1point5	Carbon and energy calculator	https://apps.labos1point5.org/ges-1point5
The Caring Scientist	Podcast	https://podcasters.spotify.com/pod/show/caring-scientist
Association for the Advancement of Sustainability in Higher Education	Resources, network, framework on sustainability performance	https://www.aashe.org/

**Table:** 

Travel	
Carbon offsetting to research on sustainable jet fuels	https://www.atmosfair.de/en/
Calculation tool on energy consumption and CO_2_ emissions in passenger transport	https://ecopassenger.org/

Information can be further obtained through networks such as *Green Your Lab* (http://greenyourlab.org/) or the *Sustainable European Laboratories Network* (https://sels-network.org/), as well as through non-profit organizations such as the *International Institute for Sustainable Laboratories* (https://www.i2sl.org/). *I2SL* organizes an annual conference, provides workshops and resources. Similarly *My Green Lab*^©^ (http://www.mygreenlab.org/) as a non-profit organization provides information, organizes the annual *freezer challenge* (https://www.freezerchallenge.org/), educates through their ambassador program (https://www.mygreenlab.org/ambassador-program.html), and has certification (https://www.mygreenlab.org/green-lab-certification.html) programs for laboratories as well as for products through their *ACT*^©^*label* (https://act.mygreenlab.org/). Another option is Labconscious^©^ (https://www.labconscious.com/), which constitutes a blog offering advice on laboratory waste, green chemistry, energy and water, while also facilitating networking to other networks and groups.

For very detailed information with hands-on advice, proof and additional measurements, we recommend our *Guidebook for Sustainability in Laboratories*^100^ (Table 2, entry 3). There researchers will find more details on measures that can be undertaken in laboratories of any kind. Thus, our previously published guidebook is complementary to this article and together a comprehensive review on laboratory sustainability is presented with a great number of actions to improve laboratory efficiency.

#### 4.2.2 Form grassroots initiatives, expand the network and acquire top-down support

The first step in achieving change is to connect with like-minded colleagues and building a team. Within such a team you can share ideas, expertise, plan and implement actions.^8^

Ideally, a Green Lab grassroots group for sustainability advocacy is formed, which has several benefits:

- Identifying and implementing possible measures is much easier in a familiar setting.

- There is an existing relationship with colleagues, hence a network to work within.

This way scientists can connect with each other, exchange ideas and distribute tasks. Sustainable laboratory practices will be possible to be implemented right from the start, as individual actions do not need much convincing or communication with other stakeholders.^8^

As soon as a group of researchers is formed it is wise to connect with other Green Lab teams across the globe to exchange information and share experiences. There are several networks out there ranging from regional, national to international and global (see Table 2; Green Your Lab (http://greenyourlab.org/), SELs (https://sels-network.org/), Green Labs NL (https://www.greenlabs-nl.eu/), *etc.*). These networks organize symposia and educational workshops, provide information, and create a sense of community. As every university or institution started at some point, connecting with other Green Teams can provide further valuable information. Obtaining national and international support, exchanging advice, ideas and resources avoid duplication efforts. Building or joining a network improves workflows locally, nationally, and internationally. It also provides a sense of community, which can help to overcome certain obstacles regarding laboratory sustainability (Section 4.3). Ultimately, a transformative change towards sustainability requires collective action.^125^

For further success, it is important to include senior staff (PIs and technicians) with full time contracts as soon as possible, as they provide institutional weight and leverage to a grassroots initiative while also providing stability and continuity over time. Grassroots initiatives in academia face high staff turnover and because some scientists are more engaged with sustainability and environmental actions than others, ongoing pilot studies, calculations or policy changes towards sustainable practices may fizzle out as soon as key members leave.^5^ Therefore it is crucial that grassroots groups are supported on an institutional level (*i.e.*, management and organization), enjoying top-down support and especially include senior staff and PIs. The reach of sustainable laboratory practices is then able to grow and sustained through different institutions, even when members depart.^7^

This growing network often comes with the benefit that more members are joining, decreasing the individual task load but creating a stronger team, being able to act and exert influence across boards and hierarchy.^5^ Involving senior staff sends a strong signal to an organization on the importance of sustainability. Experienced colleagues can bring valuable, alternative perspectives, the opportunity to facilitate investments and power to change policies.^8^ This way sustainability becomes part of the agenda of various committees and allows for a holistic and realistic approach.^3,5^ By educating students and incoming staff directly with sustainable laboratory practices and science, a cultural change and paradigm shift is achieved. This institutional change and acceptance will facilitate the implementation of future actions while ensuring continuity.^7^

Effectively the initiative is then growing to a more organized arrangement, where the team gathers every 6–8 weeks, having a structured agenda, meeting notes and action points. It is also recommended to embrace a subgroup system, where not the whole green team but parts of it focus in separate subgroups on subtasks that require more time:

- Waste and plastic recycling.

- Accreditation standard programs.

- Energy measurements and efficiency.

- Outreach and communication.

Through this approach the organization and actions are structured and thus are more likely to be adopted by senior staff, thereby influencing policies and managing structures of a university or company.^8^

Top-down support is essential for further growth and requires active communication with management and governing bodies. To achieve top-down support, gathering data or evidence in support of planned changes, evidence of cutting costs, and a clear path to carbon neutrality are essential. Here universities or companies need to acknowledge that they ultimately will save money and need to reach carbon neutrality, so supporting these grassroots initiatives will be of great benefit and deliver a business case. Sustainability or green offices are often the first point of contact at universities and can assess carbon footprints and aid in the development of roadmaps to achieve carbon neutrality. These administrative and managing offices often align with goals for sustainable laboratory practices and Green Labs and hence will accelerate institutional change.^7^ Navigating the political nature of a large research organization will help researchers acquire further interpersonal and management skills, in addition to a better understanding of organizations and funding landscapes. Automatically these scientists will obtain transferable skills outside of research practices, which are desired in economy and society, especially in view of the training aspects for young researchers for future industrial and societal jobs. By achieving top-down support the initial grassroots movement can grow very fast to a recognized project within an organization and implement changes on a large scale.

Institutional support will provide access to budgets for events, workshops, and other activities to enhance sustainability within the institution. Furthermore, Green Lab grassroots initiatives should be granted funding to replace inefficient laboratory equipment and implement changes. The resulting green cost savings should be reinvested into the fund, allowing for extended sustainable measures through a steady self-filling income to the green team budget.^5^

Successful top-down support ultimately translates into a seat at management meetings, allowing for discussing institute-wide issues and making decisions, especially when related to sustainability. This can be achieved through hiring a sustainability manager at institutes or research organizations to support, promote and implement the ideas and evidence-based recommendations of the Green Lab team.^1,5^ Appointing a sustainability manager at the department or higher level facilitates a coordinated strategy on sustainability and meeting self-imposed targets of an institution.

One may ask, why a grassroots approach often is recommended as effective: virtually every sustainability action or movement started out of personal drive and dedication. Actions are undertaken in the free time of an individual and lead to demand. The identification of potential improvements is often directly visible to employees in their own working environment, which are less obvious for management.^7^ Then people demand change from the bottom-up, become a lobby and then are heard. As grassroots groups are more agile than big institutions, exploration and piloting of new procedures and practices become easier, which then can be presented to the management as soon as they are established.^7^

With the urgency of the climate crisis and necessity for a sustainable future scientists should not wait for an institution or company to change top-down but initiate efficient transformation as soon as possible. When top-down enforcements or bottom-up demands are met alone, they usually create tensions or face rejection.^7^ Utilizing the best of both worlds, bottom-up grassroots movements should be top-down supported, meeting each other in the middle to accelerate and enhance common sustainability goals for institutional change. The whole Green Lab initiative and its success is based on a bottom-up approach and leads to evidence-based research, changes in funding criteria, cost savings, waste reduction and improvements of products.

#### 4.2.3 Enhance education, improve communication, and stimulate a behavioural shift

The field of sustainable laboratory and science practices requires different types of communication ranging from sharing technical information to advocacy and engagement. Success and behavioural shift in science is directly connected to acknowledging the urgency and nature of environmental issues. Crucially, members of the Green Lab team need to learn that there are different types of motivation regarding sustainable actions: while for some it is the urgency or fear, others are inspired by optimism and solutions. Researchers advocating for sustainable laboratory practices have to utilize clear, honest and evidence-based communication.^126^

In particular, because humans like to stay in their comfort zone, having a reluctant attitude to going beyond current frontiers, there will be resistance to action, which at times can feel frustrating for a researcher promoting sustainable actions. It is important to focus on a positive dialogue: in cases where the environment is a polarizing topic, the communication should be focusing on co-benefits such as costs or health. If a policy change at a workplace is the ultimate goal, the focus should be to maintain good relationships with co-workers and accepting smaller changes rather than winning an argument over a bigger one. Constructive dialogs and positive relationships will be a good investment in the long term.

Feeling frustration with colleagues and peers should not discourage a certain grassroots movement or scientist to demand change. Such feelings can be overcome by realizing a sense of community through conversations with like-minded scientists, friends, and colleagues. The climate crisis and biodiversity loss can feel remote or impersonal to some people, leading them to rather act locally on direct needs within their own barriers. Information alone often falls short in driving behavioural shifts. People are always looking for information that aligns with their personal values, aspects that resonate with their own identity.^127^ Taking the time to listen and having empathy leads to understanding of certain motivations. Here effective communication while engaging with other people is key to achieve behavioural changes and to keep people on board.

Individual researchers can implement and influence sustainable actions on different levels. Next to personal actions, these practices should be part of their teaching, communication with colleagues and ultimately encourage groups and institutions to adopt these changes; we owe it to the young talents we educate for their future. Engagement in public debates will further accelerate sustainable scientific research.^7^

When it comes to policy changes, funding, or procurement we recommend developing an evidence-based plan on sustainable laboratory actions. These can only be granted when recommendations are backed up with not only white papers or publications from other institutions, but also with calculations on their ‘business-case’ by in-house measurements and numbers highlighting the benefits. Often sustainability efforts save costs and are in-line with climate goals of a company or university (see Section 4.1 Benefits of Green Labs). These technical solutions, however, should be communicated with a certain understanding of diplomacy to facilitate and stimulate institutional change.^7^ Ultimately, having an institutional policy with stated goals and SMART (specific, measurable, achievable, relevant, time-bound) targets is a useful driver for change.^128^

Importantly, success stories create momentum for the next desired change. These successes should be communicated through newsletters of the institute, faculty, university, or companies. This way work is acknowledged and endorsed, resulting in people being more likely to join the grassroots movement and its visible impact is publicly supported by the management.^5^ The momentum generated keeps up engagement and opens doors for the next step. Here regular talks and presentations at institute meetings on the topic of laboratory sustainably by staff and invited speakers encourage wider actions and support recruitment of new members.^5^ We recommend that a Green Lab team should have access to the digital infrastructure of an institution (and university) with a subpage on its main webpage. This way news, progress, and resources can be shared easily, and their work should be highlighted regularly on digital information screens.^5^

It is crucial to communicate internally and externally to build awareness and achieve action within the scientific community. Environmental sustainability is often thought of as expensive, and bold decisions on sustainable laboratory practices may be received as limiting researchers' freedom.^6^ Often trade-offs are mentioned between sustainability and factors such as safety, health, regulation, costs, and research. Group leaders and PIs taking part in sustainable laboratory actions should include these aspects in their performance reviews and funding applications to educate the community of their benefits.^5^

Give feedback to people that already implemented changes on how they contribute to the sustainability improvements of the laboratories. This will keep them engaged and prone to push for more changes.

Build a culture of informed and active colleagues, sharing knowledge. Reminding colleagues of certain sustainable actions such as turning off equipment can be facilitated through stickers or posters.^30^ Inclusion of senior staff ensures the creation of a cultural change on sustainability at all levels of an institution. We further recommend once per year a team building or community activity for the researchers involved in supporting sustainable laboratory actions.

One commonly mentioned advice is to turn-off equipment and computers, when they are not in use. Switching off all non-essential electrical equipment raises awareness of the amount of electricity wasted as a result of leaving equipment on unnecessarily. Just by mass-switching off equipment during the weekend, a reduced electricity consumption of 6% can be achieved, saving 16 000 kW h equal to 7 tons of CO_2_e and 1861 €.^92^ Turning off equipment is a crucial aspect and by far one of the most important ones. It is also a great one to demonstrate a successful shift in mindset: rather than educating and telling researchers to turn off equipment, a successful paradigm shift is achieved when people think as equipment being switched-off as default. Only when people need certain equipment, they switch it on for the time it needs to be operated.

It is crucial to educate scientists, but especially students about the environmental impact of laboratories and science. As environmental awareness usually is already engrained in younger generations through media and society, they are more open and often demanding better practices when it comes to conducting research. Teaching students directly green lab practices, while teaching them standard laboratory techniques provides them with a great toolkit for their and our future. Ultimately a paradigm shift is achieved as soon as those students proceed on their academic path through Bachelors, Masters and PhD programs, finally also in future jobs in industry. This way, initial reluctance towards changes from senior staff will fade away as younger researchers enter the laboratory environment directly applying the new standard of sustainable laboratory practices.

Members and scientists of the Green Lab team will acquire transferable skills on effective communication with a variety of stakeholders. They will also develop skills on presentation, engagement and ultimately managing, all while being able to handle research projects as their main-role. These scientists are effectively growing themselves to the leaders of the sustainable future, while creating it.

#### 4.2.4 Join an accreditation standard program

We recommend joining an accreditation scheme as soon as possible. This way sustainability efforts are guided *via* a formal scheme, which enhances organization and rewarding sustainable efforts undertaken. While providing a framework of established actions, supported by evidence-based publications and whitepapers, they also help planning and structuring work, drastically reducing time to gather information and implement changes. Researchers and team members will be able to have their efforts recognized through an accreditation and auditing process. This ultimately leads to reputational benefits by receiving an award for the workplace, attracting future students and researchers. These accreditations also support spreading the environmental actions from one lab to the other through community engagement, peer-support, and peer accreditations. The rewards can seal the deal for funding organizations to grant projects and thus for PIs in joining these frameworks and Green Teams. On top of these aspects Green Team members not only can grow as sustainability experts and consultants, but also will learn how to become auditing professionals of value for their personal career.^1^ Regular audits ensure that research practices stay as efficient as possible and a ranking system (*e.g.*, bronze, silver, and gold) is creating not only engagement but also a competitive mindset within an institution.

Examples of frameworks include Green Impact, the Laboratory Efficiency Assessment Framework (LEAF (https://www.ucl.ac.uk/sustainable/leaf-laboratory-efficiency-assessment-framework)) or the *My Green Lab* (https://www.mygreenlab.org/green-lab-certification.html) certification. While MyGreenLab^©^ focuses on enlisting individual laboratories (2076 participating labs in 2023) to implement sustainable changes, LEAF^©^ operates on the institutional level (105 institutes in 16 countries with 2900 labs and 4300 users in November 2023).^2,129–131^

LEAF^©^ is an online software platform including a framework, which outlines requirements and measures to achieve various levels of standard.^2^ Online calculators on emissions and savings, technical guides, as well as training to assist with implementing the framework (*e.g.*, auditing) are included. Its costs are between 1280 and 3025 € excluding VAT per institution depending on its size, allowing for direct inclusion of all laboratories per organization.^2^ The framework provides actions for the categories: waste, people, purchasing, equipment, IT, sample and chemical management, research quality, teaching criteria, ventilation, and water (Fig. S1, ESI†). Laboratories are accredited bronze, silver, or gold depending on the performance, and the (re-)certification process runs on an annual basis.

The self-assessment through the My Green Lab (MGL) Certification is performed through an online survey covering the categories: community, recycling and waste reduction, resource management, purchasing, green chemistry and green biologics, water, plug load, fume hoods, cold storage, large equipment, infrastructure energy, field work, animal research, and travel (Fig. S3, ESI†).^2^

Depending on an online self-assessment survey, MGL provides recommendations to further improve laboratory sustainability. Here 50% of lab members must complete the survey and assessment.^2^ After actions have been implemented, the lab personnel re-take the assessment survey to quantify their progress through a calculated score and certification level. Certification levels are bronze, silver, gold, platinum and green and are achieved in accordance with the score calculated by MGL (Fig. S2, ESI†). Recertification is required after two years. The expenses are between 319 and 456 € per academic lab and 2554–3649 € per commercial lab.

If more focus on healthcare and medicine is necessary, then the *GreenED* (https://greened.rcem.ac.uk/) Framework for Environmentally and Sustainable Emergency Medicine and Health Care is recommended. Other frameworks for building sustainability and a green building rating system are *LEED* (https://www.usgbc.org/leed) *(Leadership in* Energy *and Environmental Design)* and the *Labs2Zero* Energy *Score* (https://www.i2sl.org/lab-energy-score). These tools give recommendations on several categories such as ventilation, equipment, procurement, waste, chemicals, or research quality and offer carbon and cost calculators.

The accreditation scheme of choice should be first evaluated and piloted within a smaller group of laboratories, to be able to share results relevant to the organization, company, or university. After successful completion, the framework should be rolled out to all institutes and laboratories in the organization with the previously gathered top-down support from operational decision makers.

#### 4.2.5 Implement measures based on impact

There are several measures one can implement immediately without fully completing the previously discussed steps towards green labs.

The following recommendations (Table 3) can essentially be implemented immediately and do not comprise or interfere with the research conducted and are proven to be safe.

Sustainable laboratory measures based on impact

**Table:** 

Research and education^7^
- Enhance reproducibility by conducting research at the highest quality possible, saving resources and time.
- Provide detailed information on reaction conditions, procedures, and data to enhance reproducibility.
- Record and share negative results to avoid unnecessary reproduction attempts.
- Educate students and new lab members on sustainable laboratory and research practices.

**Table:** 

Travel and conferencing
- Avoid air travel as much as possible and prioritize travel by train.
- Prioritize local conferences accessible by train.
- Attend meetings and conferences online rather than in person.
- Attend only the most important conferences overseas *via* air travel.
- Use resources on journey planning, which is discouraging of airplanes.
- Unavoidable flights should be offset with verified carbon standard projects supporting jet fuel research.

**Table:** 

Energy efficiency
- Prioritize variable air volume (VAV) fume hoods over constant volume (CV) air supply systems.^4^
- Closing the sashes of fume hoods reduces its energy consumption between 40 and 67%, in addition to being safer.^1,134^ Equipping fume hoods with sensors that trigger automatic sash closing facilitates this action.
- Increase the temperature of a ULT freezer from −80 °C to −70 °C to reduce energy consumption by 30–40% as sample stability and recovery are not affected.^31,32,137^
- Maintain an inventory list, share freezer space, and organize regular freezer cleanings to remove unneeded samples, frost buildup and dust accumulation.^31,32^ Join https://www.freezerchallenge.org.
- Turn off equipment, when not in use. Devices should be turned-off by *default* and only be switched *on*, when needed. Here multiplugs, timers and switches can facilitate a behavioural change, while stickers can serve as reminders.
- Utilize and run equipment such as autoclaves, ovens, and dishwashers only when full.
- Replace overhead lights with LED bulbs.^4^

**Table:** 

Data centres and computations
- Prioritize digital, paperless options such as digital laboratory journals and online clouds and minimize printing.
- Run calculations at times and locations with the highest amount of green energy.^129^
- If privacy allows, prioritize data centres in locations with greater sustainable source of electricity to minimize carbon footprint.^129^
- Evaluate the set point temperature in server rooms to reduce active cooling.
- Calculate the carbon footprint of the research and include those in cost-benefit analyses.^184^
- Improve the efficiency of code, prioritize C++, and optimize hardware.^23,24^

**Table:** 

Water
- Retrofit/update autoclaves with systems that recirculate or reduce water consumption, which can save about 32 000 L of water per week.^8^
- Implement aerators on taps.
- Utilization of waterless condensers.
- Cooling devices and systems should only operate in closed loops and rely on recirculated water.

**Table:** 

Chemicals
- Avoid the generation of surplus quantities.
- Implement an online chemical search and location system (so called inventory) and regularly maintain its content. Make sure that chemicals are findable and accessible.
- Share chemicals with other labs/group-members and consult the chemical search system if the compound needed is already available before ordering a new one.
- Conduct reactions in the smallest volumes possible (*i.e.*, rightsizing experiments) and check for their success before upscaling.
- Minimize the number of physical experiments *via* computational modelling and simulations, where applicable.^1^
- Utilize efficient robotic, automation, and artificial intelligence (AI) tools for high-throughput experiment optimization (‘*lab of the future*’).^215–219^
- Purchase the smallest possible quantities of chemicals sufficient for a given experiment.
- Prioritize benign and less hazardous reagents and solvents.^143–145^
- Recycle solvents and chemicals for cleaning.

**Table:** 

(Single-use) consumables
- Reduce, reuse, recycle.^20,123^
- Replace single-use plastics with glassware.^19,52^
- Reuse plastic where possible.^19,20,123,220^ Results are not affected through reusing glass or plastic as no carryovers or contamination is observed.^52^
- Reduce shipments and packaging.
- Consult suppliers and producers if there is a take back scheme for used consumables.
- Try to implement a recycling scheme for plastic consumables such as gloves, pipette tips or plastic tubes if contamination can be excluded.

**Table:** 

Glass waste
- If feasible, glassware should undergo repairs; if repair is not possible only then opt for disposal.

**Table:** 

Logistics and procurement
- Reduce the number of shipments and packaging by coordinating orders from across the institute/group.
- Coordinate orders of commonly used items *via* ‘central stores’ within the institute, which store items in bulk and supply demands on site.
- Prioritize local and responsible suppliers with a detailed sustainability plan.
- Ask manufacturers and suppliers about life cycle assessments, take-back schemes, and more sustainable alternatives to standard products.

**Table:** 

Resource efficiency
- Whenever feasible, laboratory equipment should undergo repairs; if repair is not viable, disposal and replacing with new equipment should be considered as the last option.
- Reuse equipment, computers, and furniture internally at locations/groups/projects that are in need of specialized equipment. This way group resources are preserved as well as less waste is being produced. Equipment that is not needed anymore but still intact should be donated or sold *via* second-hand refurbishing schemes.
- Implement SOPs and report every detail of experiments, this way replication and reproducibility is enhanced, and less waste is generated.

**Table:** 

Working environment, commuting and finance
- Develop a sustainable travel policy prioritizing low-carbon forms of travel.^3^
- Prioritize public transport or biking whenever possible.
- Provide a network of cycle paths, bike sheds, and other related facilities.
- Offer a free train, metro and bus pass for staff and students.
- Provide charging possibilities for electric vehicles.
- Provide technical equipment for home–office and virtual meetings.^3^
- Equip buildings with solar panels to provide self-generated renewable energy.
- Switch to a sustainable electricity provider (solar, wind).^3^
- Improve the retirement plans of staff, by switching to a sustainable solution and an ethical pension provider.^221^
- Move the bank and institution accounts to a financial institute committed to sustainable goals
- Utilize waterless urinals to significantly reduce water usage.^104^
- Prioritize plant-based (vegetarian and vegan) menu options over meat-based diet and avoid food waste.^3^
- Support actions on nature and biodiversity on the campus.
- Prioritize https://www.ecosia.org/ as the search engine for internet searches.

Nevertheless, it should be recognized that each research organization is unique and that it may be required to develop an own (adapted) approach to implement environmental actions.^104^

Generally, manufacturers that make an effort to reduce packaging waste and offer take-back schemes should be prioritized in tenders seeking minimal packaging.^50^

When it comes to reusing single-use plastics or glassware there is a common misconception regarding the costs for washing, often argued through the production, footprint and costs of certain solvents used as well as the time and salary of the person cleaning the disposables or operating the dishwasher. These arguments have been disproven through LCAs and cost analyses.^20^ In fact, re-use strategies not only reduce the carbon footprint up to 11-fold but benefit the finances of a laboratory, even when wages for support staff for washing are included. These aspects are further accelerated through a central wash facility, scaling up the number of items being re-used.^20^

For concerns about contamination and loss of precision several examples on reusing plastics in microbiology laboratories exist, where results are not affected thus no carryovers or contamination are observed.^52,107,132^ In such cases savings of 516 kg of plastic waste per laboratory (7 researchers) per year were achieved, avoiding autoclaving and incineration.

Circular supply chains for plastic products can reduce emissions by over 80%.^133^ Usually, clinical incineration of single-use consumables is causing half of the emissions of protective wear and plastics.^133^ Thus opting for circular recycling reduces lifetime emissions by up to 74%, but is currently only feasible through fully circular glove companies such as Gloovy Eco Gloves^©^ (https://gloovyecogloves.nl/en).^133^ In such cases reduction in use and exploring glass alternatives are crucial.^134^ Often suppliers and producers offer take-back schemes for used consumables, which should be prioritized.^135^

Chemicals and equipment should be shared among laboratories and staff. An online chemical search and location system (so-called inventory) facilitates these actions. Crucially, real-time information and automatic updates on the status and locations of chemicals need to be maintained to reduce time spent searching for chemicals and enhance productivity.^136^

During the holiday season it is strongly recommended to develop an action plan to switch off all devices and equipment as well as lowering the heating, or other measures for optimal lab use.^92^ These measurements should be combined with an annual laboratory cleaning (*e.g.*, cleaning and organizing fridges and freezers).^56^

Inefficient laboratory equipment should be replaced with devices having better performance. Laboratory users should aim for equipment with the best performance:^137^

- ULT freezers with electricity usage of ≤13.5 watts per litre per day.

- Refrigerators and freezers of 2.5 watts per litre per day or 1.5 kW h per day.

Utilization of computational infrastructure can be optimized by switching calculations from an average data centre to a more efficient one to reduce the carbon footprint by 34%.^42^ The location of a data centre affects the carbon impact of calculations depending on the source of energy.^138^ Thus we recommend to provide access to low-carbon computing facilities and dynamically shift jobs from data centres across multiple locations with green energy mixes (*e.g.* solar, wind).^100^ Generally an inventory of maintained hardware should be made, and energy consumption measured during computer simulations and idle times. An overview of high-performance computing (HPC) facilities, their power usage effectiveness (PUE) and energy source is necessary to assess feasibility to move computations to different computing facilities based on energy source and energy usage. Evaluate the set point temperature in server rooms to reduce active cooling.^23^ Scientists should consider ‘digital temperance’: careful evaluation about the collection of specific data for research projects rather than collecting as much as possible and give a thorough thought to the storage and analysis of these data.^41,139^

It should further be noted that this list (Table 3) does not go in as much detail as our previously published guidebook.^100^ For more details and information we recommend consulting all aspects of our guidebook.

#### 4.2.6 Transforming research itself

There are steps to be taken beyond improving laboratory efficiency. Effectively every research project can be improved by replacing outdated techniques.^140^ As climate change was not of much concern 50 years ago, scientists were not concerned with improving the energy used, code written, or chemicals used, as long as the desired outcome of a certain experiment or application was achieved in high yields and reasonable amounts of time. Instead of blindly relying on and repeating these traditional standards, researchers should create the new standards of tomorrow.

As fossil fuel depletion is of great concern, there exists extensive literature on green and sustainable chemistry, establishing biobased feedstocks and building blocks.^141,142^ Here the *12 Principles of Green Chemistry* cover all aspects of experiments and reactions.^143^ These can be expanded by the *12 Principles of Circular Chemistry*.^109,144^ With a background in chemistry, the following aspects are worth highlighting:

- Focus on the use of sustainable solvents (these can often be easily substituted without affecting the reaction outcome).^145–148^

- Consider the optimization of your purification process: an extraction, distillation, or recrystallization process can be faster, less expensive, and less solvent consuming than column chromatography.^149^

- Consider the optimization of the synthesis by applying the *12 Principles of Green Chemistry*.^150–152^ Try to find more benign, *e.g.*, biobased, building blocks and synthesize the desired compound in a catalytic reaction in a sustainable solvent.^113,115,153^ Prevent waste by designing and executing the experiments with high technical standards.^154^

- Achieving full atom economy (*e.g.*, click chemistry)^155^ presents the most efficient reaction with practically no waste.^156,157^ Here sustainable feedstocks such as biomass, plastic waste and CO_2_ should be valorised.^158–160^ The value of a procedure is enhanced the more circularity is applied.^114,144,161,162^

Crucially, these aspects should also find their way into the curriculum of Bachelors, Masters and High School programs.^155^ There are several examples to improve chemistry education and to stimulate a systematic thinking approach into the minds of early researchers and students.^164–169^ We cannot expect future scientists to create sustainable products if they are not taught how to think sustainable.^170–172^ Students need to be familiar with concepts such as LCAs, green, circular and sustainable chemistry early on.^173–176^ Transforming chemistry education, which prepares the next generation of chemists to enhance the safety, effectiveness and most importantly reduce environmental impact is a key goal of Beyond Benign^©^ (https://www.beyondbenign.org/). As of March 2024 there were 150 Green Chemistry Commitment (GCC) (https://www.beyondbenign.org/he-green-chemistry-commitment/) signers, which aims to expand the community and education of chemists through a flexible framework for green chemistry curriculum and training.^177^ It further provides access to funding opportunities and a benchmark to track progress on learning and research objectives. As all science should strive to become more sustainable and with our background in wet labs and chemistry, we acknowledge a new standard: the field of Green Chemistry should become just chemistry.^178^

### 4.3 Challenges in transitioning to green labs

There are several key challenges often associated with sustainable laboratory practices, including lack of time and data, missing budget, logistics for procurement or waste disposal, lack of involvement in institutional decisions and support from management.^5^ While every grassroots initiative is facing some of these hurdles, general and surprisingly easy change to sustainable laboratories is possible. Hereby providing guidance through this article, every successful step and sustainable action builds momentum for the next.

#### 4.3.1 Voluntary work, staff turnover, and time

Sustainable laboratory initiatives are largely being conducted by individual researchers, voluntary and unfunded next to their main profession, driven by their personal commitment to sustainability.^130^ Often young researchers (PhD candidates and postdocs) are the most passionate and main driver behind the formation of grassroots initiatives. These grass-root movements should be supported by the management and should grow into a team including full-time personnel (*e.g.*, technicians) and senior staff. The latter is crucial as continuity is needed for sustainability initiatives to prevent loss of knowledge and momentum through staff turnover due to non-permanent contracts of PhD candidates and postdocs.^5^ Although climate awareness and sustainability goals are part of the agenda of many institutions, the work and progress initiated through Green Labs often remains voluntary. These factors directly translate into time constraints, where staff are developing Green Lab initiatives alongside their primary duties. It is crucial that management, supervisors, and colleagues recognize the ‘voluntary’ work on sustainable laboratory practices as important, rather than ‘unproductive or lost’ and these should be rewarded in the career track. It should be acknowledged that engagement in green groups adds value to an institute instead of taking time from doing research.^1,5^ While still being able to follow their main profession, there should be a working culture accepting and even promoting work on sustainable laboratories as members acquire transferable skills outside of research practices, useful for the economy and society.

#### 4.3.2 Data, frameworks, and network

The lack of time of researchers to monitor, quantify, conduct experiments, or gather data, results in a missing easy-to-access central depository of knowledge, overarching and connecting all sustainable lab initiatives and tools. The availability of data, knowledge and expertise facilitating information-based decision-making and prioritisation is an often-reported challenge when it comes to sustainable laboratories.^1,135^ Across many initiatives a lack of high-quality evidence to assess their impact is observed and more research is needed.^1,2^ Depending on the field, however, there are networks and depositories existing or under development. There are still studies missing to assess every aspect of the environmental footprint of laboratories. This is also connected to the fact that many universities and companies are not reporting their carbon impact, and if so, not a comprehensive view on all Scope 1–3 emissions.^3^ There are also more data needed on improved numbers after implementation of sustainable laboratory measures, especially beyond high-income country settings. Just recently however, funding was made available to conduct research on the sustainability of laboratory practices, which will improve these aspects in the future.^179^

Although many guidelines, frameworks and networks already exist, they are mostly located in high-income countries and cover not all areas of scientific research. For example, no similar frameworks for computational research or qualitative research exist to date.^2^ Also knowledge gaps exist in understanding the sustainability of health research and carbon emissions in the health research system, which just recently are tackled through a Green Surgery Report (https://ukhealthalliance.org/sustainable-healthcare/green-surgery-report/).^180^ Networks are further missing in low- or middle-income settings. A coordinated approach is necessary to alleviate the burden on individual researchers. Universities, journals, and funders need to work together to advance environmentally sustainable research across all sectors.^2^

#### 4.3.3 Financial support

Data and frameworks available are mostly based on unfunded and voluntary research of researchers working overtime, reflecting time and money as missing resources. Hence several grassroots initiatives find themselves struggling with budget.^5^ Lack of funding often plays a role for larger institutional changes such as new waste streams and logistics. Fortunately, there are many sustainable measures and actions one can undertake without a budget, which even reduce costs (Table 3). Grant agencies need to introduce incentives to, *e.g.*, reduce plastic waste and make greener lab practices a requirement in the application process.^15^ Fortunately, this landscape is about to change, as more funding bodies are including sustainability criteria as necessity into their grant applications already as well as funding research on environmental aspects of research. Also, universities are setting net-zero targets and are starting to support and acknowledge Green Lab grassroots initiatives.

#### 4.3.4 Decision-making and management

The lack of top-down support for grassroots initiatives is an often-reported challenge, despite the fact that many universities and industry have pledged to specific targets on sustainability in the upcoming decades. Such a lack of support can result in too less involvement in decision-making.^5^ However, next to the university's sustainability targets, the previously mentioned funding bodies will act as an external force in pushing for improvements within academia or industry, to make them still eligible for receiving funding. If a research organization wants to meet its own targets, it will need support from the management and the engagement of staff and students.^5^ The insular, federalistic, and individualistic nature of academic research and departments frequently hampers acceptance and adoption of policies created by the upper management.

Changing the research and consumption behaviour of staff, without local and bottom-up engagement is a challenging aspect.^5^ Thus, grassroots groups in sustainable laboratories are essential for each university and industry meeting their own goals, being eligible for funding and delivering on emission reductions. Thus, by including full-time senior staff into a grassroots initiative it will be easier to exert influence across hierarchy, achieving recognition and top-down support.

#### 4.3.5 Logistics

Responsible waste management is dependent on appropriate services and infrastructure, which may not yet be available in all countries or regions. While some local recycling provisions and take-back schemes of manufacturers exist, these are not broadly available and can thus be a major bottleneck in the waste handling and desires of Green Lab teams, organizations, companies, and universities. Local recycling contractors are often hesitant to take materials or plastics from laboratories, because of concerns about contamination.^107^ However, as the sustainable laboratories are growing, manufacturers have already started to appreciate their customers' demands and implement more and more take-back schemes, improved on packaging and shipments, and offer biobased and biodegradable products as well as recycling options.^181^ It is crucial as the sustainable laboratory movement is becoming a bigger lobby that suppliers and manufacturers increase shifting away from traditional products towards real green alternatives.

#### 4.3.6 Travel

Prioritizing trains over air travel is often hampered by additional costs associated with rail travel. However, these costs do not reflect the real costs of both modes of transport as air travel fares do not include VAT, energy taxes or environmental compensation.^73^ From an environmental perspective, it should be made clear to policy makers that cheap and subsidized flights should not be the standard and that subsidies should rather be used for rail travel. Longer travel times (‘lost time’) are often mentioned as an argument against rail travel. It is worth noting that rail travel opens up an often-better possibility to work in a more spacious train and be as much, if not more, efficient than in an airplane. Furthermore, the time when compared from door-to-door, the time efficiency of airplanes *vs.* trains are equalling each other out, if often not much better by (night)trains. A two-hour flight usually translates to at least four hour of door-to-door travel time.^73^ With train travel there is no need for arriving two hours before departure, luggage, and security check-ins, waiting at a gate and afterwards for the luggage to be received. Moreover, train stations are often easily accessed by bikes and offer bike parking facilities, bridging the ‘last-mile’ from door-to-door.

### 4.4 Success factors and opportunities for sustainable laboratories

The Green Lab network and supporting community is finding its way into national and international programs, funding organizations, science policy, and corporate management. The associated scientists are effectively becoming an advocacy group, that lobbies for systemic change from stakeholders and funding organizations to suppliers and end users.

#### 4.4.1 Manufacturing and procurement

There exists an environmental impact factor label by emphasizing Accountability, Consistency, and Transparency (ACT (https://act.mygreenlab.org/)) during manufacturing, energy and water use, packaging, and end-of-life. Allowing for informed purchasing as the sustainability aspects of devices, equipment and other products are verified (currently 3000 products labelled). Notably, the words “green” and “sustainable” are quite unregulated, while manufacturers are profit-driven still.^131^ Purchasers need to evaluate critically if the authenticity of marketing messages is genuine.^131^

Suppliers are starting to meet the growing demand for sustainable products such as providing alternatives for single-use plastic, implementing take-back schemes, and developing greener chemicals and solvents.^123^ Currently at Merck^©^ and Sigma-Aldrich^©^ a pilot program^110^ on plastic recycling and take-back schemes is assessed to develop circular recycling solutions, reduce the amount of plastic in the value chain and offer cost-effective re-processing of plastic waste into products and packaging within their supply chain.^110^ Also packaging and deliveries are being improved where examples include companies such as MilliporeSigma^©^ recently switching from expanded polystyrene (EPS) foam to cardboard alternatives, saving 23 tons of EPS annually.^182^

In November 2023 My Green Lab^©^ started together with four major pharma companies, AstraZeneca^©^ (https://www.astrazeneca.com/sustainability.html), GSK^©^, Amgen^©^, and Bristol Myers Squib^©^ the Converge^183^ initiative. Harnessing the collective power of the pharmaceutical industry they collectively request that suppliers with significant laboratory operations to certify their laboratories through sustainable laboratory frameworks by 2030, while also providing sustainable products.

Demand is a crucial driving force, herein; the more researchers request suppliers to assess and declare the carbon footprint of their products, the more likely suppliers will provide and act on these data with improved products.^10^ This momentum hopefully keeps on growing as more and more scientists are educating others and future generations of students. Improving environmental awareness, responsibility and training in the laboratories will lead to a prospering lab-supply industry.^123^

#### 4.4.2 Resources and reproducibility

The field of sustainable laboratory research is gaining rapid momentum over the past years.^1,2^ There are well-developed tools, guidebooks and approaches existing, all of which are coordinated through several Green Lab Networks globally.^1,2^ To date, two main certification and accreditation programmes are existing for reducing the environmental impact of (wet) laboratories: *LEAF* and *My Green Lab Certification*.

The sustainability of dry labs and computational research is also coordinated through a network and provides numerous calculators to measure the carbon footprint of various types of computations, models and algorithms.^23,100,184^ One example is the Green Algorithms (https://www.green-algorithms.org/) calculator: an open-access tool to estimate the environmental impact of algorithms used without affecting the existing code and covering a wide range of hardware configurations.^2,38^ Since its introduction in 2020, the calculator has been utilized by about 15 000 users across over 20 000 sessions, averaging to approximately 200 users per week globally.^2^

Medical and clinical research has fewer well-established resources, but recent initiatives are busy developing or improving measurement protocols, standards, and tools.^185–188^ In November 2023 with the Green Surgery Report (https://ukhealthalliance.org/sustainable-healthcare/green-surgery-report/) the UK Health Alliance on Climate Change published an impressive first guide aiming to reduce the environmental impact of surgical care while maintaining high quality patient care.^180^ The data and guidance presented are based on evidence, case studies, cover barriers and the key contributors of emissions in healthcare (single-use items, energy consumption, anaesthetic gases).^104,180^ Additionally, there are innovative cleaning technologies (https://envetec.com/generations/) emerging (*e.g.*, Envetec^©^ (https://envetec.com/)) for the treatment of medical waste: Northwell Health^©^ (https://www.northwell.edu/), as New York's premier healthcare provider, is adopting such techniques to sustainably treat over 226 796 kg of regulated medical waste annually onsite, with projected decrease of waste-related Scope 3 emissions by 90%.^189–192^

Also, aspects of sharing negative results are being improved not only within research groups and internal presentations, but also through dedicated journals. The Journal of Trial and Error (https://journal.trialanderror.org/) aims to close the gap between what is researched and what is published. Ultimately these frameworks allow the reduction of reproducibility issues, waste production and time loss through repeating experiments.^117^

#### 4.4.3 Funding and publishing

There exist several initiatives such as the Million Advocates for Sustainable Science (https://www.sustainablescienceadvocates.org/) or Bringing Efficiency To Research (BETR) Grants (https://betrgrants.weebly.com/) organized by *My Green Lab*^©^ and I^2^SL to demonstrate support for a systemic change within the global science funding system.^89^ Signing and participating scientists request action by research funders to update funding structures with environmental standards on sustainability. Impressively, several funding bodies are already including sustainability criteria as necessity into their grant applications.^88^ Examples include, but are not limited to, the US National Institutes of Health^193^ (NIH) with their Green Labs Program, the Marie Skłodowska-Curie Actions Green Charter^194^ promoting sustainable research activities into mobility and training of researchers, Deutsche Forschungsgemeinschaft^195^ (DFG) anchoring environmental sustainability in funding activities, the Wellcome charitable foundation^196^ enforcing a carbon offset policy, the environmental sustainability strategy by UK Research and Innovation^197^ (UKRI), Cancer Research UK^198^ or the Science Foundation Ireland^199^ (SFI) with funding available for sustainable laboratory certifications.^200^ Specific funding tools for research on environmental aspects of research have emerged, such as the Sustainable Laboratories Grant by the Royal Society of Chemistry^179^ (RSC) or UK's Medical Research Council.^201^

As for publishers, examples exist such as the journal *Research in Engineering Design*, which developed a Research Environmental Impact Disclosure statement as a requirement to provide an environmental impact statement for the submission of journal articles or grant applications.^1^

This progress clearly marks a shift in the funding landscape for academia and industry, where mandatory standards for resource-efficient science are set. Political pressure (top-down) as well as institutions and researchers/reviewers (bottom-up) need to advance these developments further through requests, committee-work, and target setting to update funding requirements/conditions.^10^ Ultimately, it is advised that Green Lab certifications such as *LEAF* or *My Green Lab* become requirements on par with ethical, health, and safety reviews in grant applications.

### 4.5 Collaborative partnerships for a sustainable future

There is clear evidence that people are concerned about climate change – especially younger generations demand sustainable development incorporated into their practices.^6^ Two thirds of students would accept a salary sacrifice of 15% to work for an institution with a good environmental and social record, indicating that younger generations care more about health, safety, and a stable planet.^6^

#### 4.5.1 Credibility

There are several studies reporting that scientists' personal behaviour has an impact on public perception: individuals perceived carbon-conscious climate scientists as more credible and trustworthy compared to those with significant carbon footprint.^202^ Consequently, the audience reported a greater willingness to consider taking climate action in their own lives after listening to these scientists discuss strategies to reduce energy use.^79^ Thus, researchers worldwide play a major role in achieving a sustainable future.^203^

#### 4.5.2 Conferencing and travel

One of the most effective ways to reduce emission of greenhouse gases is to cut down on long-distance air travel.^16,204^ There are 8.4 million researchers globally as of 2015, who, with the current career norms, are expected to travel several times a year to scientific meetings.^205^ Large scientific communities with an annual meeting should cut back to one large meeting every two years or less.^16^ Generally, it is hard to justify hosting conferences in distant vacation spots (*e.g.*, Hawaii) due to their substantial carbon footprint, lacking real benefits to the local scientific community.^80,205^

Scientific societies, researchers and funders should work together to improve the format and organization of conferences:^205,206^

- Frequency.

- Size.

- Location with local hubs.

- All talks live streamed and recorded.

- Electronic posters.

- Electronic-only program books.

- Carbon neutrality *via* virtual conferencing.

- Reduce energy and resource use.

- Sustainable catering (*e.g.*, plant-based).

- Food waste management.

- Visa-free attendance.

Meetings should rather be organized around local hubs (*e.g.*, America, Europe, Asia) running in a parallel and synchronous fashion, where attendees travel as much as possible *via* train or other ground-based transportation, still allowing for in-person networking opportunities and social interactions. Such a multi-location in-person model should focus on hubs being located in central rather than remote cities of each continent, to which keynote speakers are invited locally. Regional society meetings provide benefits such as low hosting costs as they allow for more economic public venues.^205^ Analysing attendance patterns indicate Chicago, Tokyo and Paris as suitable host cities, which could reduce the combined travel emissions of conferences by 80%.^80^ As the conference is taking place at all locations at the same time, people should have access to online-presentation rooms to be able to follow talks taking place live at a different hub. Livestreaming and recordings made available online will benefit other researchers globally, promote inclusivity, and increase the scope of audiences reached.^205^ This multi-location in-person model, where participants only travel to nearby locations to interact with other ‘local’ scientists benefit the scientists as personal productivity is enhanced as time will not be lost by driving to an airport or waiting to board a plane.

Other aspects include the elimination of merchandise, utilizing compostable conference name badges, reducing plastic waste usage (*e.g.*, disposable cutlery) to a minimum, eliminating food waste, or encouraging attendees to bring their own reusable materials such as cups or making notes electronically on portable devices.^205^ International meetings are frequently planned 5–10 years ahead through booking of convention centres, a reason more to start thinking about more sustainable alternatives rather sooner than later.^16^

Grant review panels organized by funding organisations and other similar activities, that do not require face-to-face meetings, should prioritize remote video-conferencing. Generally the scientific community should make online communication, conferencing and video-calls the standard.^3,73,207^ Online portals allow for attending more meetings in a time efficient manner, increasing the outreach.^208^ If adopted on a global scale, reductions in long distance travel by the scientific community would drastically reduce carbon emissions.^16,209^ Actions of scientists adhering to this new *status quo* should be valued and should influence policies in a way that subsidies/funding for air travel prices are stopped and shifted to *e.g.*, train travel enhancing efficiency.

#### 4.5.3 Publishing and education

Consumption of resources and energy impact is directly connected to publications and papers: researchers and PIs should promote science with a focus on quality instead of quantity. This way sustainability of science is drastically improved, while also tackling ‘academic treadmills’ of pushing out several publications on the same topics fast.^7^

Researchers should further share their experience and knowledge on sustainability programmes, not only within their own institution but also through creating resources and publications to further enable the scientific community for a systemic change with additional evidence and data.

#### 4.5.4 Buildings and construction

As the efficiency and sustainable aspects of laboratories are already determined during the design phase it is crucial to focus on low-energy design.^210^ Scientists, architects, and designers need to collaborate to facilitate common and fundamental project goals such as energy efficiency and renewable energy sources. The layout of laboratory and office spaces need to match environmental standards, as sustainable laboratory practices are really thriving under optimized building conditions.

The prevailing approach to comprehensive building sustainability is currently the LEED program (https://www.usgbc.org/leed) (Leadership in Energy and Environmental Design) by the U.S. Green Building Council. LEED^©^ serves as a framework for rating and certifying buildings and their systems, offering guidance in areas such as energy and water conservation, use of healthy and sustainable construction materials, indoor air quality, and other aspects during construction and renovation.^30^

Energy decisions should be based on the full life cycle of devices and equipment, making variable air volume (VAV) fume hoods the standard over constant volume (CV) air-supply systems for energy-efficient operation.^4^ Office and noncritical support spaces should be segregated from laboratory space to enhance airflow. Generally, it is wise to segregate spaces and, when feasible, cascade air from one room to the other (*e.g.*, air conditioning or heat pump in between cooled/heated spaces).^4^ It is wise to include controls, timers and occupancy sensors in devices with diverse loads such as lights, computers and fume hoods.^4^

The baseload energy consumption of science buildings *vs.* the usage by the users, *i.e.*, running the building (heating, cooling, ventilation, lighting, *etc.*) *vs.* the activities in the building (science activities, instrument use, computers *etc.*), often averages at 75–80% to keep buildings operational and thus 20–25% of energy consumption is associated with users (Table S12,† ESI). We recommend acquiring such knowledge for individual institutions to enhance directing of funds and efforts to maximize the sustainability impact.

Finally, in industry as well as in academia it should be considered to investigate on-site power generation through renewable energy (photovoltaic (PV) for footpath, parking-area and roofing materials) as it has a positive economic impact. Furthermore, heat pumps can drastically reduce the costs for domestic heating replacing gas usage.^4^ Depending on the location, green power through electricity providers utilizing hydropower, wind farms or PV systems should be examined. Laboratory efficiency can be enhanced through sharing laboratory- and facility spaces, where a previous study demonstrated space savings of up to 30%.^120^ These aspects complement the sharing of chemicals and laboratory equipment, fostering a collaborative research space.^120^

#### 4.5.5 Relevance and scale

Advocating and working on sustainable laboratory practices and science often has a bigger direct impact on the carbon footprint of the university than a research project. Changes with local and immediate impact need to be applied directly and can contribute to direct savings. Such efforts should be prioritized over research projects with possible long term beneficial outcomes to industrial application or scale. Sustainable laboratory practices, when communicated effectively, create momentum towards institutional but also societal changes. Regular community events, presentations, workshops, and panel discussions in the wider network of Green Labs are important to keep up the momentum and help engaging other researchers. Sustainable science outreach facilitates further expansion to other institutions.^8^

Educating and implementing the new standards of sustainable laboratory practices starting from laboratory practicals and PhD programs will ultimately carry over to corporate research, as soon as these young researchers move on their career path and apply their green standards in their new working environment. As universities are interlinked and collaborating with industry, those practices will become recognized there as well.

### 4.6 Guiding sustainable laboratory practices for a global impact

Many gaps in understanding and reducing the environmental footprint of laboratories remain. The challenge to provide more and higher-quality evidence to assess the impact of science and laboratory research should be tackled by aiming for comprehensive carbon footprint assessments of organizations. Covering Scope 1 to 3 emissions through the entire value-chain is a crucial aspect in evaluating environmental and cost benefits through systemic change.^3,6^ While carbon offsetting schemes can be part of the solution in short term, effectively bridging the gap until sufficient data are collected and technologies developed, institutions should not rely mainly on those compensations without actually reducing emissions in line with global targets.^6^

As science has the obligation to be a leading sector in this transformation, it is crucial to be honest about challenges and mistakes made, educating stakeholders, other scientists, policy makers, and society worldwide about concrete ways to improve sustainability and operations.^6^ As institutions all over the world face similar challenges in reaching carbon neutrality, implementing high impact measures, connecting as a network, and providing guidance to each other is crucial, to enhance progress and avoid ‘re-inventing the wheel’.^6^ Scientists, companies, organizations, universities, institutions, suppliers and funding agencies need to work together collectively and share insights to effectively reduce their carbon footprint.^6^ If we are successful, significant benefits are achieved: if only half of all American laboratories would reduce their energy use by 30%, their total annual energy consumption could be reduced equivalent to 840 000 households, €1.14 billion and 19 million tons of carbon dioxide emissions.^4^ Such a systemic change would correspond to removing 1.3 million cars from highways or preventing harvesting 56 million trees.^4^

#### 4.6.1 Leadership

It takes ambitious leaders to establish sustainable practices into laboratories and science. Institutions have to express their commitment and have to be appreciative about researchers' work in transforming outdated practices. As companies and universities ultimately will save money and need to reach carbon neutrality, those leaders need to be supported as much as possible. They should be consulted in decision-making, supported *via* green offices, receive funding, and achieve full top-down support.^6^ Those scientists becoming committed leaders will take ambitious decisions and drive the progress forward.

Furthermore, bottom-up engagement and grassroots initiatives are essential for a sustainable transformation process as behavioural change by individuals is directly affecting Scope 3 emissions.^6^

Ultimately, while there are already great tools, frameworks, and networks available on the importance of sustainable science and laboratory practices, those actions alone will not be sufficient. It is necessary that those efforts are amplified through larger bodies:

1. Funding organizations need to make it a requirement that scientific research has to be conducted by adhering to sustainable laboratory standards and practices acknowledging environmental responsibility.

There should be policies in place mandating sustainable practices and setting targets.^1,211^ Applicants must discuss the climate impact of their project in their application and should be allowed to choose the least carbon-intensive instead of the economically cheapest way to travel.^3^ Committee work should be virtualized in online meetings whenever possible.

2. Suppliers and manufacturers need to provide sustainable product alternatives with similar or better properties than usual standards. These alternatives need to be cost competitive and broadly advertised to support a shift in the scientific community. Full life cycle assessments of products should be made available to guide costumers to sustainable products. Consumers have a responsibility in requesting them frequently. Single-use consumables need to be evaluated to reduce plastic waste production, where mono-streams facilitate recycling. Deliveries and logistics should be moving away from on-demand to weekly or biweekly, thereby reducing emissions from delivery and packaging. Packaging itself should not rely on plastics. Take-back recycling schemes for solvent bottles, gloves and other plastics need to be established to achieve circularity.

3. Conference organizers should move away from annual meetings and reduce the meeting frequency to biannually or less. Here options for virtual attendance need to be provided and a multi-location in-person model has been strongly recommended to minimize the environmental effects of travel.^73^ Splitting the conference from one major location to at least three accessible hubs, will reduce travel impacts while also promoting equity and inclusivity.

4. Publishers and journals should recognize their responsibility in advocating for sustainable science and laboratory practices. While publishing a growing number of articles addressing the environmental impact of research, they also have a responsibility to raise awareness on sustainable strategies, actions, and policies.

5. Universities, scientists, and individual researchers need to educate one another on sustainable knowledge, skills, and experimental design. These include green chemistry, life cycle assessments, and sustainable laboratory operation and practices. Environmental impact and its reduction need to be included in internal and external evaluations of laboratories, departments, and organizations.^1^ Sustainability is as important as health and safety and should be incentivised in policies.^124^

In the past, the academic system has experienced numerous changes, often prompted by society.^3^ In the face of the climate challenge, the academic system has the potential to undergo again a transformative shift, this time towards sustainability.^212^ Our generation of scientists and researchers has the opportunity and obligation to limit the most extreme outcomes of the climate crisis.^127,213^ In contrast to many other societal sectors, the academic system benefits from independent academics being the key decision makers in shaping framework conditions for the future and most importantly educating our new generation. Hence, the academic system is strategically well positioned to engage in a self-directed transformation to climate sustainability.^3,214^ We urge and encourage our colleagues worldwide, irrespective of their roles or levels in the scientific community to participate in these collective endeavours to create a sustainable future!

## 5 Conclusion

Scientific research and laboratories are consuming excessive amounts of energy, generate (toxic) waste and deplete resources. Bottom-up green lab efforts have emerged to address many of these challenges and provide examples of best practices. To date, there are several budding Green Lab examples and initiatives seeking to address the environmental footprint of research. Driven by voluntary efforts of researchers, they educate peers, develop sustainability guidelines, write scientific publications, and maintain accreditation frameworks. This article aids as a comprehensive tool in understanding the relevance of sustainable laboratory practices and on how to improve as a scientific community. We presented evidence for the environmental impact of laboratories, expanded it with recent data by the University of Groningen, followed by guidelines for sustainable lab practices and hands-on advice on how to achieve and maintain a systemic change.

The University of Groningen's Faculty of Science and Engineering emits 68 tons of CO_2_e per publication, equalling to annual emmisions of about 9 tons per person and 0.19 tons per m^2^. The joined laboratories of the University of Groningen produce 109 tons of hazardous chemical waste and 17 tons of plastic waste annually. Typically, a chemistry laboratory (45 active laboratory researchers) can produce up to 7 tons of hazardous chemical waste annually, corresponding to 157 kg per person per year. Similarly, a biology laboratory (17 active lab members) produces about 533 kg of plastic waste equalling 32.4 kg per person per year. By applying sustainable laboratory practices the Green Labs RUG team achieved a reduced carbon impact equalling annual savings of 398 763 € as well as 477.1 tons of CO_2_e, which corresponds to savings of 10 372 kg of CO_2_e and 8669 € per lab per year. Efficient equipment management in a two-week winter break led to additional 247 646 € of savings in 2022–2023. The majority of students and university employees demand climate action in academia and science, and our data are further demonstrating a business case for investing in sustainability.^6^

Driving lasting change will require ambitious leaders and sustainability experts, opening opportunities for new job roles, professional development, and further innovation.^1^ Scientists should not be part of the problem, but part of the solution! If we, as scientists and researchers, believe what we are publishing, should we not be the first ones to act? How can one expect industry, politics, and society to change, if we as scientists are not changing anything either? Scientists should lead and educate by example, improve their practices using the scientific method, and be the change they want to see!

## Author contributions

T. F., M. M. L., B. L. F. conceptualized the research project and coordinated it. T. F. and N. E. conducted the research and its validation with the support of M. H. and M. M. L. T. F. prepared the manuscript with input from N. E., M. H., M. M. L., B. L. F. All authors reviewed the manuscript.

## Conflicts of interest

There are no conflicts to declare.

## Note added after first publication

This article replaces the version published on 18th March 2024, which contained errors in citations linked to the sentences beginning 'Depending on the location...' and 'Laboratory efficiency can...'. The correct citation to reference 120 has replaced the original, incorrect citation to reference 114.

## Supplementary Material

SU-002-D4SU00056K-s001

## References

[cit1] Royal Society of Chemistry , Sustainable Laboratories: A Community-wide Movement toward Sustainable Laboratory Practices, Cambridge, 2022

[cit2] Smith P. , Feijao C., Ang C., Politi C., Flanagan I., Qu M. and Guthrie S., Welcome Report: Advancing Environmentally Sustainable Health Research, London, 2023

[cit3] ALLEA , Towards Climate Sustainability of the Academic System in Europe and beyond, ALLEA, Berlin, 1st edn, 2022

[cit4] EPA , Murray K., Lintner W., Carlisle N. and Sartor D., Laboratories for the 21st Century: an Introduction to Low-Energy Design, 2008

[cit5] Dobbelaere J., Heidelberger J. B., Borgermann N. (2022). J. Cell Sci..

[cit6] Borgermann N., Schmidt A., Dobbelaere J. (2022). One Earth.

[cit7] Winter N., Marchand R., Lehmann C., Nehlin L., Trapannone R., Rokvić D., Dobbelaere J. (2023). EMBO Rep..

[cit8] Durgan J., Rodríguez-Martínez M., Rouse B. (2023). Immunol. Cell Biol..

[cit9] Farlie F., Palmer G. A., Cohen J., Calcagni C., Gorbunova A., Lawford Davies J., Loscher C., O'Raghallaigh R., Sharp T., Smale D., Sörme P., Thiel C. L., Alteri A., Campbell A., Crompton K., Mortimer S., Pisaturo V., Tolpe A., Alikani M. (2024). Reprod. BioMed. Online.

[cit10] Farley M. (2022). Nat. Rev. Mol. Cell Biol..

[cit11] Rockström J., Steffen W., Noone K., Persson Å., Chapin F. S., Lambin E. F., Lenton T. M., Scheffer M., Folke C., Schellnhuber H. J., Nykvist B., De Wit C. A., Hughes T., Van Der Leeuw S., Rodhe H., Sörlin S., Snyder P. K., Costanza R., Svedin U., Falkenmark M., Karlberg L., Corell R. W., Fabry V. J., Hansen J., Walker B., Liverman D., Richardson K., Crutzen P., Foley J. A. (2009). Nature.

[cit12] Steffen W., Richardson K., Rockström J., Cornell S. E., Fetzer I., Bennett E. M., Biggs R., Carpenter S. R., De Vries W., De Wit C. A., Folke C., Gerten D., Heinke J., Mace G. M., Persson L. M., Ramanathan V., Reyers B., Sörlin S. (2015). Science.

[cit13] IPCC , Lee H. and Romero J., IPCC, 2023, pp. 1–184

[cit14] Robinson A., Lehmann J., Barriopedro D., Rahmstorf S., Coumou D. (2021). npj Clim. Atmos. Sci..

[cit15] Urbina M. A., Watts A. J. R., Reardon E. E. (2015). Nature.

[cit16] Nathans J., Sterling P. (2016). Elife.

[cit17] Valero M. V. (2023). Nature.

[cit18] Madhusoodanan J. (2020). Nature.

[cit19] Bowler J. (2022). Nature.

[cit20] Farley M., Nicolet B. P. (2023). PLoS One.

[cit21] Jain N. (2022). Nat. Rev. Methods Primers.

[cit22] Fardet T., Hütten M., Lohmann S., Medawar E., Milucka J., Roesch J. H., Rolfes J. D., Schweizer J. (2020). Front. Sustain..

[cit23] Lannelongue L., Aronson H.-E. G., Bateman A., Birney E., Caplan T., Juckes M., McEntyre J., Morris A. D., Reilly G., Inouye M. (2023). Nat. Comput. Sci..

[cit24] Callahan W. , James Fava S. A., Wickwire S., Sottong J., Stanway J. and Ballentine M., Corporate Value Chain (Scope 3) Accounting and Reporting Standard Supplement to the GHG Protocol Corporate Accounting and Reporting Standard, GHG Protocol Team, 2011

[cit25] Estevez-Torres A., Gauffre F., Gouget G., Grazon C., Loubet P. (2024). Green Chem..

[cit26] Woolliams J., Lloyd M., Spengler J. D. (2005). Int. J. Sustain. High. Educ..

[cit27] Kitzberger T., Kotik J., Pröll T. (2022). Energy Build..

[cit28] May M. J. (2016). Science.

[cit29] Mills E., Sartor D. (2005). Energy.

[cit30] Aldred Cheek K., Wells N. M. (2020). Front. Built Environ..

[cit31] Gumapas L. A. M., Simons G. (2013). World Rev. Sci. Technol. Sustain. Dev..

[cit32] Bousema T. , The temperature setting of freezers: -70 is the new -80, https://www.freezerchallenge.org/uploads/2/1/9/4/21945752/minus-70-is-the-new-minus-80_3.pdf, accessed 24 August 2023

[cit33] Gilly Q. , Fume Hood Strategy, White Paper, 2023, pp. 1–12

[cit34] Environment Report 2019-2020, HSE unit at CERN, https://hse.cern/environment-report-2019-2020, accessed 7 November 2023

[cit35] King P. , Akeroyd F., Cross G., Mayers J., Payne S., Sivia D., Terry A., Thompson H., van Duijn J., Clements D. and Howells S., ISIS Annual Report 2005 - Review of the Year, Part of the ISIS Facility, 2005

[cit36] Berners-Lee M. , The Carbon Footprint of Everything, Greystone Books, London, Revised Edition., 2022

[cit37] Lannelongue L., Inouye M. (2023). Nat. Rev. Methods Primers.

[cit38] Lannelongue L., Grealey J., Inouye M. (2021). Advanced Science.

[cit39] Training a single AI model can emit as much carbon as five cars in their lifetimes, MIT Technology Review, https://www.technologyreview.com/2019/06/06/239031/training-a-single-ai-model-can-emit-as-much-carbon-as-five-cars-in-their-lifetimes/, accessed 7 November 2023

[cit40] Koot M., Wijnhoven F. (2021). Appl. Energy.

[cit41] Samuel G., Lucassen A. M. (2022). Digital Health.

[cit42] Grealey J., Lannelongue L., Saw W. Y., Marten J., McRossed D Sign G., ric, Ruiz-Carmona S., Inouye M. (2022). Mol. Biol. Evol..

[cit43] Martin P., Brau-Nogué S., Coriat M., Garnier P., Hughes A., Knödlseder J., Tibaldo L. (2022). Nat. Astron..

[cit44] The climate issue (2020). Nat. Astron..

[cit45] Stevens A. R. H., Bellstedt S., Elahi P. J., Murphy M. T. (2020). Nat. Astron..

[cit46] Top 9 Actions to Take in the Lab to Improve Water Efficiency, https://www.mygreenlab.org/blog-beaker/top-9-actions-to-take-in-the-lab-to-improve-water-efficiency, accessed 7 November 2023

[cit47] Watch D. and Tolat D., Sustainable Laboratory Design, 2016

[cit48] My Green Lab Ambassador Program, https://www.mygreenlab.org/ambassador-program.html, accessed 12 January 2024

[cit49] Liew F. E., Nogle R., Abdalla T., Rasor B. J., Canter C., Jensen R. O., Wang L., Strutz J., Chirania P., De Tissera S., Mueller A. P., Ruan Z., Gao A., Tran L., Engle N. L., Bromley J. C., Daniell J., Conrado R., Tschaplinski T. J., Giannone R. J., Hettich R. L., Karim A. S., Simpson S. D., Brown S. D., Leang C., Jewett M. C., Köpke M. (2022). Nat. Biotechnol..

[cit50] Arnott A. , Cheek K., Alves J. and Pickering A., Sustainable Lab Consumables Guide, 2021

[cit51] Fostier M., Grady R. (2020). Biochem..

[cit52] Kilcoyne J., Bogan Y., Duffy C., Hollowell T. (2022). PLOS Sustain. Transform..

[cit53] Rae C. L., Farley M., Jeffery K. J., Urai A. E. (2022). Brain Neurosci. Adv..

[cit54] Rautela R., Arya S., Vishwakarma S., Lee J., Kim K. H., Kumar S. (2021). Sci. Total Environ..

[cit55] Forti V. , Balde C. P., Kuehr R. and Bel G., The Global E-Waste Monitor 2020: Quantities, Flows and the Circular Economy Potential, United Nations University/United Nations Institute for Training and Research, International Telecommunication Union, and International Solid Waste Association, 2020

[cit56] Bakkalci D., Farley M., Kessler F., Cheema U. (2023). Environ. Sustainability.

[cit57] Van Norman G. A. (2019). J. Am. Coll. Cardiol. Basic Trans. Sci..

[cit58] Perel P., Roberts I., Sena E., Wheble P., Briscoe C., Sandercock P., Macleod M., Mignini L. E., Jayaram P., Khan K. S. (2007). Br. Med. J..

[cit59] Akhtar A. (2015). Camb. Q. Healthc. Ethics.

[cit60] Wadman M. (2023). Science.

[cit61] Moutinho S. (2023). Nat. Med..

[cit62] Doke S. K., Dhawale S. C. (2015). Saudi Pharm. J..

[cit63] Hutchinson I., Owen C., Bailey J. (2022). Animals.

[cit64] Kramer M., Font E. (2017). Biol. Rev..

[cit65] Burnett J., Clarke M., Darbyshire J., Haines A., Lilford R., Ramos M., Roberts I., Shakur H., Siegfried N., Wilkinson P. (2007). BMJ.

[cit66] Rossi G., Manfrin A., Lutolf M. P. (2018). Nat. Rev. Genet..

[cit67] Burtscher L., Dalgleish H., Barret D., Beuchert T., Borkar A., Cantalloube F., Frost A., Grinberg V., Hurley-Walker N., Impellizzeri V., Isidro M., Jahnke K., Willebrands M. (2021). Nat. Astron..

[cit68] Wynes S. and Donner S. D., Addressing Greenhouse Gas Emissions from Business-Related Air Travel at Public Institutions: A Case Study ofthe University of British Columbia, 2018

[cit69] Grémillet D. (2008). Nature.

[cit70] Viglione G. (2020). Nature.

[cit71] Achten W. M. J., Almeida J., Muys B. (2013). Ecol. Indic..

[cit72] Desiere S. (2016). EuroChoices.

[cit73] Burtscher L., Barret D., Borkar A. P., Grinberg V., Jahnke K., Kendrew S., Maffey G., McCaughrean M. J. (2020). Nat. Astron..

[cit74] Moss V. A., Balaguer-Nuñez L., Bolejko K., Burtscher L., Carr A., Di Teodoro E. M., Gregory B., Hanko E., Hill A. S., Hughes A., Kaper L., Kerrison E. F., Lockman F. J., Lowson N., Stevens A. R. H. (2022). Nat. Astron..

[cit75] Burtscher L., Balaguer-Núñez L., D'Orazi V., Barret D., Beuchert T., Dil E., Janiuk A., Mingo B., Poggio E. (2022). Nat. Astron..

[cit76] van der Tak F., Burtscher L., Zwart S. P., Tabone B., Nelemans G., Bloemen S., Young A., Wijnands R., Janssen A., Schoenmakers A. (2021). Nat. Astron..

[cit77] Stroud J. T., Feeley K. J. (2015). Ecography.

[cit78] Spinellis D., Louridas P. (2013). PLoS One.

[cit79] Rosen J. (2017). Nature.

[cit80] Klöwer M., Hopkins D., Allen M., Higham J. (2020). Nature.

[cit81] Ciers J., Mandic A., Toth L. D., Veld G. O. t. Sustainability.

[cit82] Wynes S., Donner S. D., Tannason S., Nabors N. (2019). J. Cleaner Prod..

[cit83] Schneider M., Romer M., Tschudin M., Bolio H. (2011). Cem. Concr. Res..

[cit84] International Energy Agency (IEA) , Global Status Report for Buildings and Construction: towards a Zero-Emissions, Efficient and Resilient Buildings and Construction Sector, 2019

[cit85] Helmers E., Chang C. C., Dauwels J. (2021). Environ. Sci. Eur..

[cit86] My Green Lab, The Carbon Impact of Biotech & Pharma, 2023

[cit87] Subaiya S., Hogg E., Roberts I. (2011). Trials.

[cit88] Luck M. and Farley M., Research Professional News, 2023

[cit89] Greever C., Ramirez-Aguilar K., Connelly J. (2020). FEBS Lett..

[cit90] Mariette J., Blanchard O., Berné O., Aumont O., Carrey J., Ligozat A., Lellouch E., Roche P.-E., Guennebaud G., Thanwerdas J., Bardou P., Salin G., Maigne E., Servan S., Ben-Ari T. (2022). Environ. Res.: Infrastruct. Sustainability.

[cit91] EU-27: per capita GHG emissions 1990-2021|Statista, https://www.statista.com/statistics/986460/co2-emissions-per-cap-eu/, accessed 24 January 2024

[cit92] Robinson O., Kemp S., Williams I. (2015). J. Cleaner Prod..

[cit93] Larsen H. N., Pettersen J., Solli C., Hertwich E. G. (2013). J. Cleaner Prod..

[cit94] Rodríguez-Martínez M. (2022). Biologis.

[cit95] UI GreenMetric, https://greenmetric.ui.ac.id/, accessed 22 January 2024

[cit96] 24/7 Clean Energy – Data Centers – Google, https://www.google.com/about/datacenters/cleanenergy/, accessed 15 January 2024

[cit97] EMBL , 2022 Energy & Carbon Footprint Report, 2022

[cit98] Nature Index annual tables, https://www.nature.com/nature-index/annual-tables/, accessed 29 December 2023

[cit99] rug.nl – Website Carbon Calculator, https://www.websitecarbon.com/website/rug-nl/, accessed 29 December 2023

[cit100] Freese T. , Kat R., Lanooij S. D., Böllersen T. C., De Roo C. M., Elzinga N., Beatty M., Setz B., Weber R. R., Malta I., Gandek T. B., Fodran P., Pollice R. and Lerch M. M., ChemRxiv, 2023, preprint, pp. 1–74, https://doi.org/10.26434/chemrxiv-2023-g3lmq-v4

[cit101] Welke milieukeurmerken heeft het papier van Canon? – Canon Help Center, https://hogeschool-van-amsterdam.zendesk.com/hc/nl/articles/9896479611666-Welke-milieukeurmerken-heeft-het-papier-van-Canon, accessed 30 December 2023

[cit102] Plastic packaging waste in EU rose by 23% in ten years; Netherlands best for recycling|NL Times, https://nltimes.nl/2022/10/23/plastic-packaging-waste-eu-rose-23-ten-years-netherlands-best-recycling, accessed 30 December 2023

[cit103] Anastas P. T. and Warner J. C., Green Chemistry: Theory and Practice, Oxford University Press, New York, 1998

[cit104] Yusuf E., Luijendijk A., Roo-Brand G., Friedrich A. W. (2022). Clin. Microbiol. Infect..

[cit105] https://www.mygreenlab.org/blog-beaker/record-breaking-success-2023-freezer-challenge-reduces-emissions-by-21-million-kwh

[cit106] 2023 Freezer Challenge Winners - International Laboratory Freezer Challenge, https://www.freezerchallenge.org/award-winners-2023.html, accessed 22 January 2024

[cit107] Howes L. (2019). ACS Cent. Sci..

[cit108] https://zerowasteboxes.terracycle.nl/products/wegwerp-pbm-zero-waste-box

[cit109] https://grenovasolutions.com/tipnovus-4-2/

[cit110] Plastic Recycling Programs, https://www.sigmaaldrich.com/NL/en/services/support/recycling/biopharma-recycling-program, accessed 23 January 2024

[cit111] Roggema R. (2021). Sustainability.

[cit112] The Carbon Neutral Laboratory, The University of Nottingham, https://www.nottingham.ac.uk/chemistry/research/centre-for-sustainable-chemistry/the-carbon-neutral-laboratory.aspx, accessed 23 August 2023

[cit113] Anastas P., Eghbali N. (2010). Chem. Soc. Rev..

[cit114] Keijer T., Bakker V., Slootweg J. C. (2019). Nat. Chem..

[cit115] Anastas P. T., Zimmerman J. B. (2003). Environ. Sci. Technol..

[cit116] https://www.youtube.com/watch?v=Zk_CEmyHZZg

[cit117] Baker M., Penny D. (2016). Nature.

[cit118] Madhusoodanan J. (2020). Nature.

[cit119] CU Green Labs and Richardson J., Solvent Recycling and Reuse, Environmental Center, University of Colorado, Boulder, https://www.colorado.edu/ecenter/greenlabs/solventrecycling, accessed 6 September 2023

[cit120] Greever C. , Nahreini T. and Rimrez-Aguilar K., A Case Study of the Biochemistry Cell Culture Facility at the, University of Colorado, Boulder, Colorado, 2018

[cit121] Dolgin E. (2021). Nature.

[cit122] Sustainable Labs – Harvard Office for Sustainability, https://sustainable.harvard.edu/schools-units/sustainable-labs/, accessed 13 January 2024

[cit123] Bistulfi G. (2013). Nature.

[cit124] Mailand N., Mirsanaye A. S. (2023). Mol. Cell.

[cit125] Otto I. M., Donges J. F., Cremades R., Bhowmik A., Hewitt R. J., Lucht W., Rockström J., Allerberger F., McCaffrey M., Doe S. S. P., Lenferna A., Morán N., van Vuuren D. P., Schellnhuber H. J. (2020). Proc. Natl. Acad. Sci. U. S. A..

[cit126] Chapman D. A., Lickel B., Markowitz E. M. (2017). Nat. Clim. Change.

[cit127] Sustainable behaviour? Information alone is not enough | News articles, University of Groningen, https://www.rug.nl/news/2024/01/information-alone-is-not-enough, accessed 30 January 2024

[cit128] Doran G. T. (1981). Manage Rev..

[cit129] Seydel C. (2023). Nat. Methods.

[cit130] Tozer L. (2023). Nature.

[cit131] Farley M. (2023). Mol. Cell.

[cit132] Alves J., Sargison F. A., Stawarz H., Fox W. B., Huete S. G., Hassan A., Mcteir B., Pickering A. C. (2021). Access Microbiol..

[cit133] Ragazzi Id I., Farley M., Id K. J., Id I. B. (2023). PLOS Sustain. Transform..

[cit134] Sawyer A. (2019). Biotechniques.

[cit135] Kernaghan S. M., Coady T., Kinsella M., Lennon C. M. (2024). RSC Sustainability.

[cit136] MilliporeSigma , Chemical Waste: the True Cost of Inefficient Inventory Management, 2020

[cit137] Farley M. , Arnott A., Smith D., Bennet M. and Lewis A., Sustainable Laboratory Equipment Metering, Procurement, and Operations Guide, 2020

[cit138] Souter N. E. , Lannelongue L., Samuel G., Racey C., Colling L., Bhagwat N., Selvan R. and Rae C., OSF Preprints, 2023, 10.31219/osf.io/7q5mh PMC12007543 40799714

[cit139] Chevance G., Hekler E. B., Efoui-Hess M., Godino J., Golaszewski N., Gualtieri L., Krause A., Marrauld L., Nebeker C., Perski O., Simons D., Taylor J. C., Bernard P. (2020). Lancet Digital Health.

[cit140] Freese T., Meijer J. T., Feringa B. L., Beil S. B. (2023). Nat. Catal..

[cit141] Tuck C. O., Pérez E., Horváth I. T., Sheldon R. A., Poliakoff M. (2012). Science.

[cit142] Sheldon R. A. (2016). Green Chem..

[cit143] Prat D., Wells A., Hayler J., Sneddon H., McElroy C. R., Abou-Shehada S., Dunn P. J. (2015). Green Chem..

[cit144] Flerlage H., Slootweg J. C. (2023). Nat. Rev. Chem.

[cit145] Cseri L., Kumar S., Palchuber P., Szekely G. (2023). ACS Sustain. Chem. Eng..

[cit146] Prat D., Hayler J., Wells A. (2014). Green Chem..

[cit147] Byrne F. P., Jin S., Paggiola G., Petchey T. H. M., Clark J. H., Farmer T. J., Hunt A. J., Robert McElroy C., Sherwood J. (2016). Sustainable Chem. Processes.

[cit148] Alfonsi K., Colberg J., Dunn P. J., Fevig T., Jennings S., Johnson T. A., Kleine H. P., Knight C., Nagy M. A., Perry D. A., Stefaniak M. (2008). Green Chem..

[cit149] Freese T., Fridrich B., Crespi S., Lubbe A. S., Barta K., Feringa B. L. (2022). Green Chem..

[cit150] Zimmerman J. B., Anastas P. T., Erythropel H. C., Leitner W. (2020). Science.

[cit151] Hermens J. G. H., Freese T., Alachouzos G., Lepage M. L., van den Berg K. J., Elders N., Feringa B. L. (2022). Green Chem..

[cit152] Hermens J. G. H., Freese T., Van Den Berg K. J., Van Gemert R., Feringa B. L. (2020). Sci. Adv..

[cit153] Anastas P. T., Zimmerman J. B. (2016). Chem.

[cit154] Freese T., Meijer J. T., Brands M. B., Alachouzos G., Stuart M. C. A., Tarozo R., Gerlach D., Smits J., Rudolf P., Reek J. N. H., Feringa B. L. (2024). EES Catal..

[cit155] Fu Y., Wu K., Alachouzos G., Simeth N. A., Freese T., Falkowski M., Szymanski W., Zhang H., Feringa B. L. (2023). Adv. Funct. Mater..

[cit156] Sheldon R. A. (2012). Chem. Soc. Rev..

[cit157] Trost B. M. (1991). Science.

[cit158] Farkas V., Nagyházi M., Anastas P. T., Klankermayer J., Tuba R. (2023). ChemSusChem.

[cit159] Sun Z., Bottari G., Afanasenko A., Stuart M. C. A., Deuss P. J., Fridrich B., Barta K. (2018). Nat. Catal..

[cit160] Brienza F., Cannella D., Montesdeoca D., Cybulska I., Debecker D. P. (2024). RSC Sustainability.

[cit161] Mahaffy P. G., Matlin S. A., Holme T. A., MacKellar J. (2019). Nat. Sustain..

[cit162] Zuiderveen E. A. R., Kuipers K. J. J., Caldeira C., Hanssen S. V., van der Hulst M. K., de Jonge M. M. J., Vlysidis A., van Zelm R., Sala S., Huijbregts M. A. J. (2023). Nat. Commun..

[cit163] Chemistry education needs a green reset , Nature, 2022, 604, 598 35478242 10.1038/d41586-022-01109-z

[cit164] Sharma R. K., Yadav S., Gupta R., Arora G. (2019). J. Chem. Educ..

[cit165] Cunningham A. D., Ham E. Y., Vosburg D. A. (2011). J. Chem. Educ..

[cit166] Hill N. J., Hoover J. M., Stahl S. S. (2013). J. Chem. Educ..

[cit167] Edgar L. J. G., Koroluk K. J., Golmakani M., Dicks A. P. (2014). J. Chem. Educ..

[cit168] Nigam M., Tuttle D., Morra B., Dicks A. P., Rodriguez J. (2023). Green Chem. Lett. Rev..

[cit169] Bastin L. D., Nigam M., Martinus S., Maloney J. E., Benyack L. L., Gainer B. (2019). Green Chem. Lett. Rev..

[cit170] Etzkorn F. A., Ferguson J. L. (2023). Angew. Chem., Int. Ed..

[cit171] Orgill M. K., York S., Mackellar J. (2019). J. Chem. Educ..

[cit172] Sheldon R. A. (2023). Green Chem..

[cit173] Jessop P. G., MacDonald A. R. (2023). Green Chem..

[cit174] Cannon A., Edwards S., Jacobs M., Moir J. W., Roy M. A., Tickner J. A. (2023). RSC Sustainability.

[cit175] MacKellar J. J., Constable D. J. C., Kirchhoff M. M., Hutchison J. E., Beckman E. (2020). J. Chem. Educ..

[cit176] Cannon A. S., Keirstead A.
E., Hudson R., Levy I. J., MacKellar J., Enright M., Anderson K. R., Howson E. M. (2021). J. Chem. Educ..

[cit177] Cannon A., Warner J., Vidal J., O'Neil N., Nyansa M. M. S., Obhi N., Moir J. W. (2024). Green Chem..

[cit178] Lane M. K. M., Rudel H. E., Wilson J. A., Erythropel H. C., Backhaus A., Gilcher E. B., Ishii M., Jean C. F., Lin F., Muellers T. D., Wang T., Torres G., Taylor D. E., Anastas P. T., Zimmerman J. B. (2023). Nat. Sustain..

[cit179] Royal Society of Chemistry , Sustainable Laboratories Grant, https://www.rsc.org/prizes-funding/funding/find-funding/sustainable-laboratories-grant/, accessed 7 January 2024

[cit180] Brighton & Sussex Medical School , Centre for Sustainable Healthcare and UK Health Alliance on Climate Change, Green Surgery: Reducing the Environmental Impact of Surgical Care (v1.1), London, 2023

[cit181] Omura T., Isobe N., Miura T., Ishii S., Mori M., Ishitani Y., Kimura S., Hidaka K., Komiyama K., Suzuki M., Kasuya K., Nomaki H., Nakajima R., Tsuchiya M., Kawagucci S., Mori H., Nakayama A., Kunioka M., Kamino K., Iwata T. (2024). Nat. Commun..

[cit182] SMASH Packaging Progress, https://www.sigmaaldrich.com/NL/en/life-science/ssbi/smash-packaging/smash-packaging-progress, accessed 23 January 2024

[cit183] https://www.mygreenlab.org/blog-beaker/my-green-lab-unveils-converge-a-collaborative-supply-chain-initiative-bringing-sustainability-to-the-forefront-of-the-pharmaceutical-industry

[cit184] Lannelongue L., Grealey J., Bateman A., Inouye M. (2021). PLoS Comput. Biol..

[cit185] Taha Uçar K. (2023). J. Health Sci. Med..

[cit186] GreenED Framework for Health Care, https://greened.rcem.ac.uk/, accessed 15 August 2023

[cit187] Cooke E., Cussans A., Clack A., Cornford C. (2022). J. Clim. Change Health.

[cit188] Sustainability|RCEM, https://rcem.ac.uk/sustainability-in-rcem/, accessed 14 January 2024

[cit189] About Envetec, https://envetec.com/, accessed 6 March 2024

[cit190] Envetec GENERATIONS - Envetec, https://envetec.com/generations/, accessed 6 March 2024

[cit191] Northwell Health, https://www.northwell.edu/, accessed 6 March 2024

[cit192] Northwell Collaborates With Envetec to Become First Health Care System in US to Implement Innovative Clean Technology to Treat Regulated Medical Waste - Envetec, https://envetec.com/news/2024/01/northwell-collaborates-with-envetec-to-become-first-health-care-system-in-us-to-implement-innovative-clean-technology-to-treat-regulated-medical-waste/, accessed 6 March 2024

[cit193] https://nems.nih.gov/green-teams/Pages/NIH-Green-Labs-Program.aspx

[cit194] https://marie-sklodowska-curie-actions.ec.europa.eu/about-msca/msca-green-charter

[cit195] https://www.dfg.de/en/service/press/press-releases/2023/press-release-no-28

[cit196] https://wellcome.org/grant-funding/guidance/carbon-offset-policy-travel

[cit197] https://www.ukri.org/publications/ukri-environmental-sustainability-strategy/

[cit198] https://www.cancerresearchuk.org/sites/default/files/cancer_research_uk_position_statement_on_environmental_sustainability_of_research_2022_1.pdf

[cit199] https://www.sfi.ie/research-news/publications/SFI-Climate-Strategy.pdf

[cit200] https://www.sfi.ie/sustainable-lab-cert/

[cit201] https://www.ukri.org/opportunity/environmental-sustainability-in-life-sciences-and-medical-practice/

[cit202] Attari S. Z., Krantz D. H., Weber E. U. (2016). Clim. Change.

[cit203] Westlake S. , The power of leading by example with high-impact low-carbon behaviour: emulation, trust, credibility, justice, Cardiff University, 2022, https://orca.cardiff.ac.uk/id/eprint/159995/

[cit204] Hiltner K. , A Nearly Carbon-Neutral Conference Model, 2020

[cit205] Sarabipour S., Khan A., Seah Y. F. S., Mwakilili A. D., Mumoki F. N., Sáez P. J., Schwessinger B., Debat H. J., Mestrovic T. (2021). Nat. Hum. Behav..

[cit206] Holden M. H., Butt N., Chauvenet A., Plein M., Stringer M., Chadès I. (2017). Nat. Ecol. Evol..

[cit207] Skiles M., Yang E., Reshef O., Muñoz D. R., Cintron D., Lind M. L., Rush A., Calleja P. P., Nerenberg R., Armani A., Faust K. M., Kumar M. Nat Sustainability.

[cit208] Remmel A. (2021). Nature.

[cit209] Raven R., Hadfield P., Butler B., Eagleton J., Giraud G., Jacob M., Markard J., Schiller K., Swilling M., Tshangela M. (2023). Glob. Sustain..

[cit210] Poliakoff M., Licence P., George M. W. (2018). Curr. Opin. Green Sustainable Chem..

[cit211] Macfarlane A. R., Ben-Ari T., Blanc G., Bozzato D., Calmer R., Haslett S., Holste S., Jardé E., Rixen C., Ruché D., Schneebeli M., Smith M. M., Thielke L., Vandevelde S., Wheeler H. C. (2024). Front. Sustain..

[cit212] Wassénius E., Bunge A. C., Scheuermann M. K., Resare Sahlin K., Pranindita A., Ohlsson M., Blandon A., Singh C., Malmcrona Friberg K., Villarrubia-Gómez P. (2023). Sustainability Sci..

[cit213] Newman R., Noy I. (2023). Nat. Commun..

[cit214] Hickel J., Kallis G., Jackson T., O'Neill D. W., Schor J. B., Steinberger J. K., Victor P. A., Ürge-Vorsatz D. Nature.

[cit215] Slattery A., Wen Z., Tenblad P., Sanjosé-Orduna J., Pintossi D., den Hartog T., Noël T. (2024). Science.

[cit216] Manzano J. S., Hou W., Zalesskiy S. S., Frei P., Wang H., Kitson P. J., Cronin L. (2022). Nat. Chem..

[cit217] Rohrbach S., Šiaučiulis M., Chisholm G., Pirvan P. A., Saleeb M., Mehr S. H. M., Trushina E., Leonov A. I., Keenan G., Khan A., Hammer A., Cronin L. (2022). Science.

[cit218] Molga K., Szymkuć S., Gołębiowska P., Popik O., Dittwald P., Moskal M., Roszak R., Mlynarski J., Grzybowski B. A. (2022). Nat. Synth..

[cit219] Wu T. C., Aguilar-Granda A., Hotta K., Yazdani S. A., Pollice R., Vestfrid J., Hao H., Lavigne C., Seifrid M., Angello N., Bencheikh F., Hein J. E., Burke M., Adachi C., Aspuru-Guzik A. (2023). Adv. Mater..

[cit220] Houghton C., Saurya S., Foster B. (2022). Biochem.

[cit221] Rethinking your pension may just be the greenest thing you can do|New Scientist, https://www.newscientist.com/article/mg24833040-200-rethinking-your-pension-may-just-be-the-greenest-thing-you-can-do/, accessed 7 January 2024

