# Supplementary material for: The relevance of sustainable laboratory practices
Source: RSC Sustain. 2024 Mar 18;2(5):1300–36. doi: 10.1039/d4su00056k (PMC11078267; doi:10.1039/d4su00056k)
Supplement: SU-002-D4SU00056K-s001 [file SU-002-D4SU00056K-s001.pdf]

Electronic Supplementary Material (ESI) for RSC Sustainability.
This journal is © The Royal Society of Chemistry 2024

Supplementary Information

for

The relevance of sustainable laboratory practices

Thomas Freese,[a] Nils Elzinga,[b] Matthias Heinemann,[c] Michael M. Lerch,*[a] Ben L. Feringa*[a]

a. Stratingh Institute for Chemistry, University of Groningen, Nijenborgh 4, 9747AG Groningen, The Netherlands

b. Green Office, University of Groningen, Broerstraat 5, 9712 CP Groningen, The Netherlands

c. Groningen Biomolecular Sciences and Biotechnology Institute, University of Groningen, Nijenborgh 4, 9747 AG Groningen, The Netherlands

E-Mail: m.m.lerch@rug.nl, b.l.feringa@rug.nl

Table of Contents

1. Sustainable laboratory accreditation frameworks 3
  1.1 Laboratory Efficiency Assessment Framework (LEAF) 3
  1.2 My Green Lab Certification 4
2. Case study of the University of Groningen 5
  2.1 Roadmap Sustainability 2021-2026 (Green Office, Groningen) 5
    2.1.1 Ambitions per theme 5
  2.2 University of Groningen - CO_2e calculations 8
    2.2.1 Environmental performance indicators 11
    2.2.2 Institutional data 12
    2.2.3 UG's CO_2e emissions, energy consumption and waste. 13
  2.3 Faculty of Science and Engineering (FSE) - CO_2e calculations, electricity, and gas consumption. 16
    2.3.1 Institutional data 16
    2.3.2 FSE CO_2e emissions 17
    2.3.3 FSE electricity and gas consumption 18
  2.4 Travel at the University of Groningen 21
    2.4.1 Introduction 21
    2.4.2 Results - University 22
    2.4.3. Results - Faculty and departments 23
  2.5 Health, Safety & Sustainability (HSE) reports FSE 27
    2.5.1 HSE report 2021 27
    2.5.2 HSE report 2022 32
  2.6 FSE is going green 37
    2.6.1 Feringa Building 40
    2.6.2 Travel behavior of staff at the Faculty of Science and Engineering 42
    2.6.3 Food and canteens 50
    2.6.4 Biodiversity 53
  2.7 Green Labs RUG 54
    2.7.1 Recommended structure of a green lab grassroot team 54
    2.7.2 Laboratory efficiency assessment framework (LEAF) at FSE 55
    2.7.3 Energy measurements, list of equipment, funding 60
    2.7.4 Winter break closure 2022-2023 at FSE 70
    2.7.5 Travel data of the authors 74
    2.7.6 Hazardous waste production at FSE 76
    2.7.7 Marketplace for second-hand laboratory equipment 80
    2.7.8 Art from laboratory waste 81
    2.7.9 Scope 3 - Furniture CO_2e calculations at FSE 84
    2.7.10 Interviews and outreach 86
3. References 87

1. Sustainable laboratory accreditation frameworks

1.1 Laboratory Efficiency Assessment Framework (LEAF)

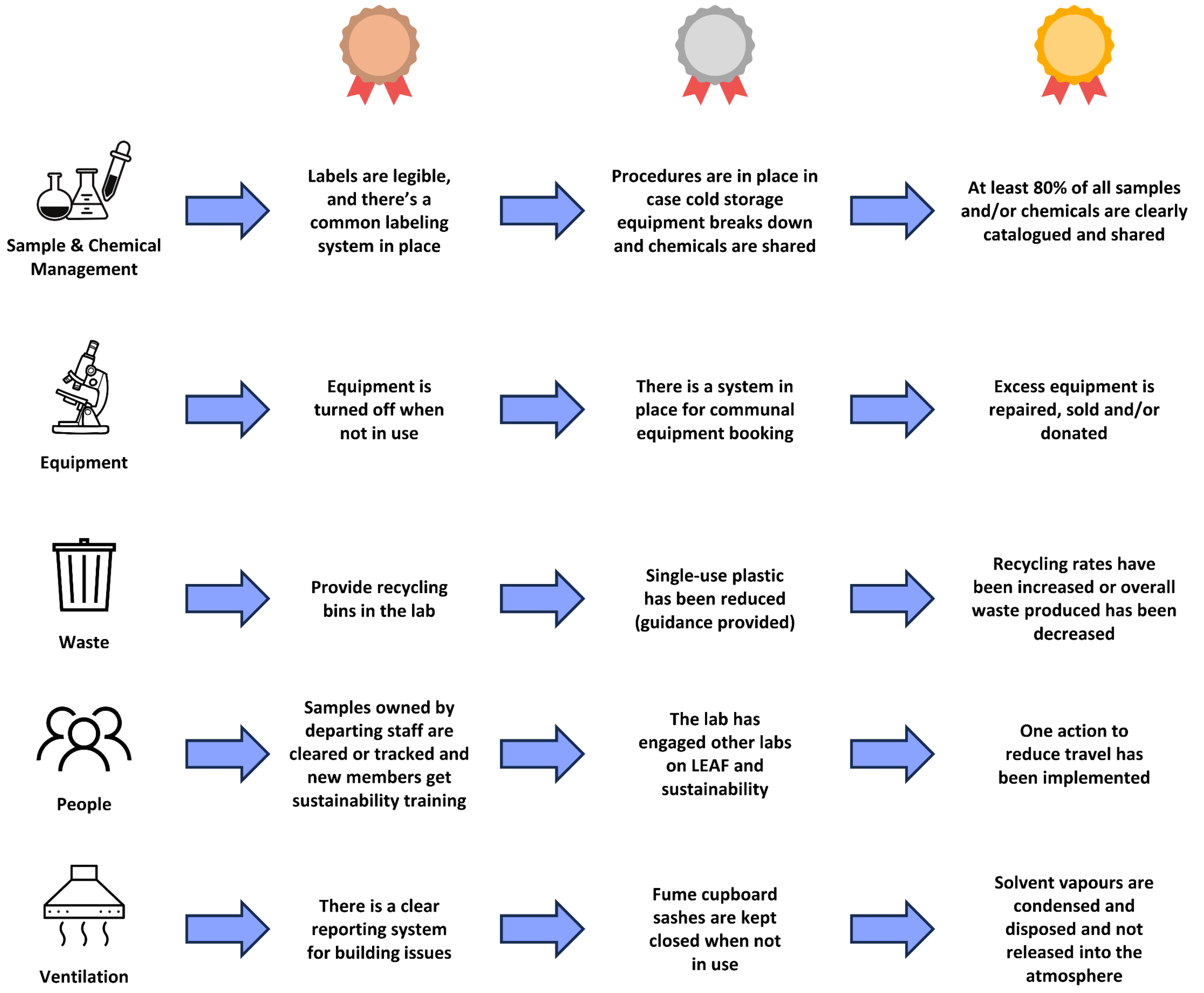

Figure S1: Key criteria and different levels of the Laboratory Efficiency Assessment Framework (LEAF); detailed descriptions available upon request at University College London (UCL). *Adapted with permission of the UCL and the LEAF developers (https://www.ucl.ac.uk/sustainable/case-studies/2020/aug/take-part-leaf)* .

1.2 My Green Lab Certification

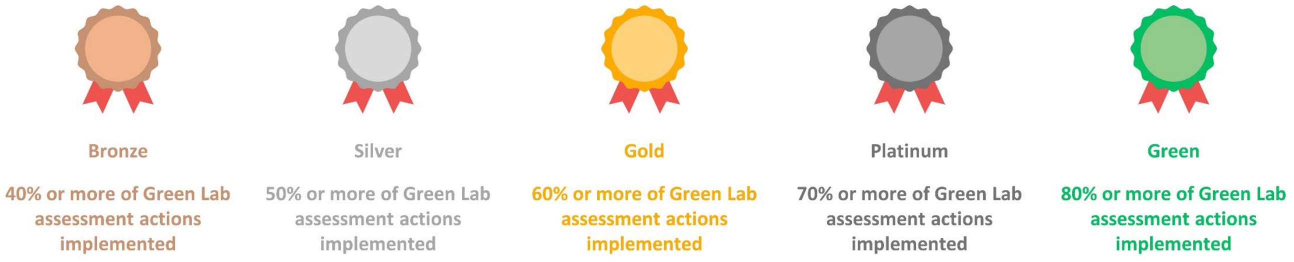

Figure S2: Accreditation levels of the My Green Lab certification. More details can be found at: https://www.mygreenlab.org/green-lab-certification.html.

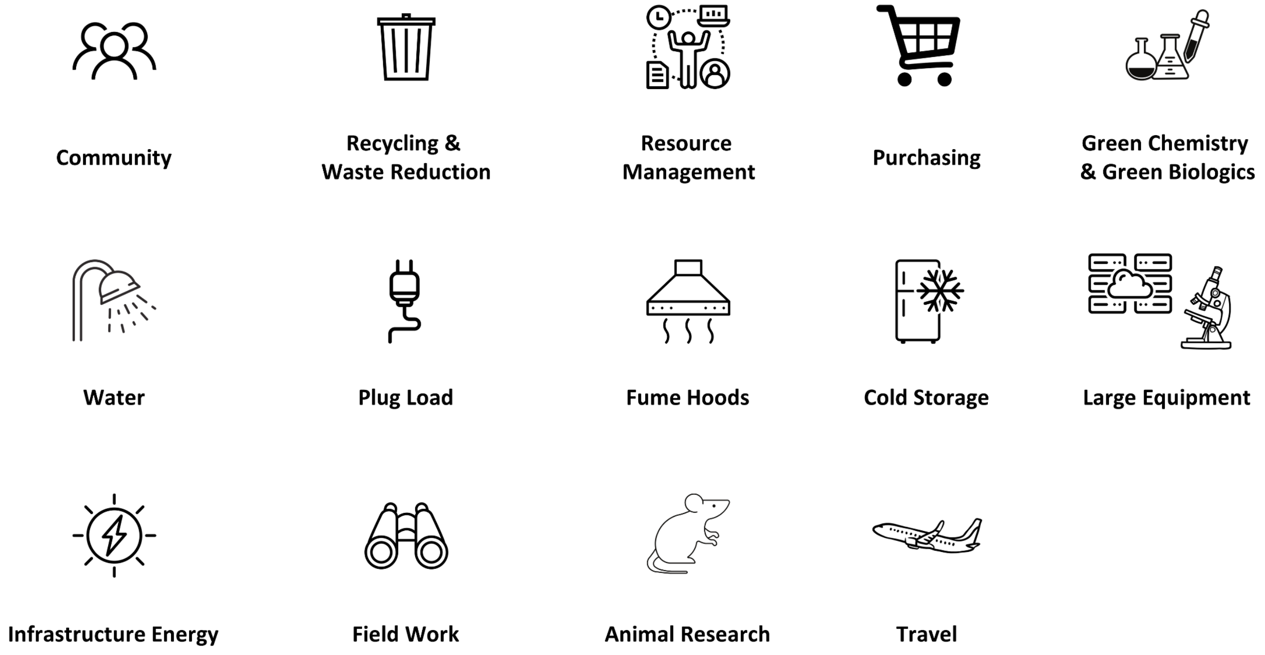

Figure S3: Topics covered through the certification process by My Green Labs. More details can be found at: https://www.mygreenlab.org/green-lab-certification.html/#certificationprocess.

2. Case study of the University of Groningen

2.1 Roadmap Sustainability 2021-2026 (Green Office, Groningen)

The following sections are adapted with permission of the Green Office, University of Groningen. The roadmap can be found in more detail at https://www.rug.nl/about-ug/profile/facts-and-figures/duurzaamheid/roadmap-sustainability.

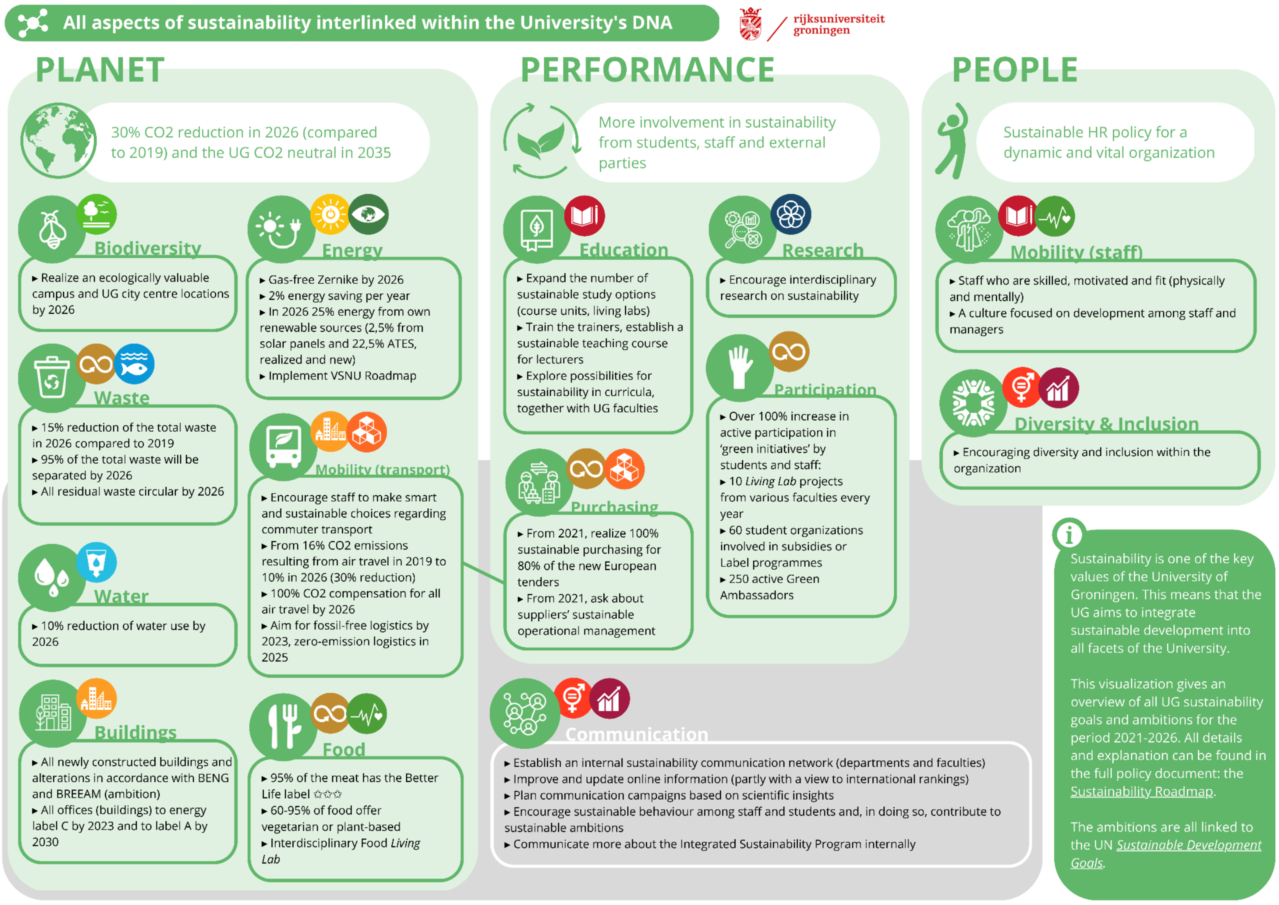

Figure S4: Visualization of the sustainability roadmap 2021-2026 of the UG by the Green Office. *Credit: F. W. Nijp (Green Office).*

Based on new legislation of the Netherlands' Climate Agreement and the United Nations (UN) Sustainable Development Goals (SDGs), the Green Office, together with various partners within the UG (such as the University Services Department, the Property and Investment Projects department (VGI), the Sustainability Sounding Board, various academics and the HR department), have planned Sustainability Roadmap for the period 2021-2026. The progress will be reported annually and is part of the UG's PDCA (Plan Do Check Act Cycle). The ambitions have once more been formulated for **Planet** (the green side of sustainability), **Performance** (sustainability in teaching and research) and **People** (sustainable employability and development of staff) but are also linked to the United Nations' Sustainable Development Goals.

2.1.1 Ambitions per theme

The following ambitions have been drawn up for the key themes **Planet**, **Performance** and **People** and for Communication. Policies have already been drawn up and a budget has been made available for some of these ambitions. Following approval, the remaining ambitions will be worked on further, in collaboration with the relevant departments or by the Green Office itself.

2.1.1.1 Planet *UG towards a CO_2-neutral University by 2035*

In May 2019 the Netherlands' Climate Agreement and the sustainability roadmap for Dutch universities was commissioned by the Dutch government and by the Association of Universities in the Netherlands (UNL) (Dutch climate policy: https://www.government.nl/documents/reports/2019/06/28/climate-agreement; sustainability roadmap of UNL: https://www.universiteitenvannederland.nl/en). The UG will adhere to the goals from the climate agreement for 2030 and 2050, with the ultimate goal of reducing the CO_2 emissions to zero. In 2016, the UG signed the Groningen Energy Neutral 2035 roadmap by the Groningen Municipality (https://gemeente.groningen.nl/groningen-co2-neutraal-2035), in which it was agreed that the UG will comply with the Municipality of Groningen's goals of being CO_2 neutral by 2035. This means that the UG must be CO_2 neutral by 2035, irrespective of the VSNU agreements.

From 2021, there will be stricter regulations for newly constructed buildings regarding energy use, with the aim of constructing buildings that are almost energy neutral. In 2050, there must be an energy saving of 27% compared to 2005, while the remaining energy must be generated in accordance with the climate agreement. In doing so, the new ambitions that are listed in **Table S1** will be leading in the UG's new strategic accommodation plan.

Table S1: Roadmap ambitions regarding the planet category.

**Ambitions in accordance with the statutory requirements (compliant with the Environmental Protection Act)**
**CO_2**<br>➔ 30% CO_2 reduction by 2025 compared to 2019.
**Energy**<br>➔ In accordance with statutory requirements, 2% energy saving per year.<br>➔ All offices (buildings) to energy label C by 2023 and to label A by 2030.<br>➔ Newly constructed buildings and alterations in accordance with BENG (almost energy-neutral buildings).
**UG ambitions (determined based on feasibility)**
**CO_2**<br>➔ CO_2 neutral by 2035.
➔ In 2026, 25% of energy from own renewable sources:<br>◆ 2.5% from solar panels.<br>◆ 22.5% ATES (Aquifer thermal energy storage), realized and new.<br>➔ Implement VSNU Roadmap:<br>◆ gas-free Zernike by 2026.<br>◆ aim for BREEAM standard 'excellent'.
**Waste**<br>➔ By the end of 2026, 95% of the total waste (hazardous and non-hazardous) will be separated.<br>➔ 15% reduction of the total waste in 2026 compared to 2019 (from 29 kg to 25 kg per staff member/student).<br>➔ All residual waste circular by 2026.
**Biodiversity**<br>➔ Realize an ecologically valuable (target species policy) campus and UG city centre locations by 2026.
**Water**<br>➔ Reduction in the amount of water per user (staff member or student) of 5% in the short term (compared to 2019) and in total 10% in the long term (by 2026).
**Transport mobility**<br>➔ Encourage staff to make smart and Mobility sustainable choices regarding commuter transport.<br>➔ From 16% CO2 emissions resulting from air travel in 2019 to 10% in 2026 (30% reduction).<br>➔ 100% CO2 compensation for all air travel by 2026.<br>➔ Aim for fossil-free logistics by 2023, ahead of the Municipality of Groningen's policy: emission-free logistics by 2025 – extend this proposed municipal policy from the city centre to all UG locations.
**Food**<br>➔ 95% of the meat has the Better Life label ☆☆☆.<br>➔ 60-95% of the food offered in canteens and by catering is vegetarian or plant-based (depending on the location).<br>➔ Establish an interdisciplinary Food Living Lab to study and facilitate the transitions to sustainable food systems including plant-based food.
**Hydrogen**<br>➔ Explore the possibilities for the application of hydrogen at Zernike campus.

2.1.1.2 Performance *More involvement in sustainability from students, staff and external parties*

The new goals for teaching and research will be formulated in consultation with the new schools and with that of Sustainable Society in particular (https://www.rug.nl/rudolf-agricola-school/). New ambitions have been formulated to encourage bottom-up green initiatives to involve students and staff in making the UG more sustainable: (1) ambassadors who organize activities to make their own faculty more sustainable, (2) green committees; small interdisciplinary groups comprising staff who wish to help solve the specific environmental problems at the UG (*e.g.* plastic waste in laboratories), (3) study associations that can apply for grants and obtain sustainable labels and (4) GO Living Lab projects whereby students collaborate with GO for curricular projects that are related to sustainability (https://www.rug.nl/about-ug/profile/facts-and-figures/duurzaamheid/wat-kun-je-doen/samenwerken-in-onderzoek-?lang=en) . Work is in progress on the goals that are listed in the table below.

Table S2: Roadmap ambitions regarding the performance category.

**Teaching**<br>➔ Expand the number of sustainable study options (course units, living labs).<br>➔ Train the trainers, establish a sustainable teaching course for lecturers.<br>➔ The Sustainability Sounding Board's teaching and research group will start talks with faculties to explore how sustainability can be incorporated more into the curriculum without deviating from the content of the teaching programme.<br>➔ Within the new Schools, specific attention will be paid to sustainable teaching, including in the form of interdisciplinary Minors that are yet to be developed and that will be accessible to all UG students.
**Research**<br>➔ Encourage interdisciplinary research on sustainability.
**Bottom-up involvement**<br>➔ By 2026, over 100% increase in active participation in 'green initiatives' by students and staff:<br>◆ 250 active ambassadors and at least four interdisciplinary green committees.<br>◆ 60 student organizations involved in subsidies or label programmes.<br>◆ 10 Living Lab projects from various faculties every year.
**Purchasing**<br>➔ From 2021, realize 100% sustainable purchasing for 80% of the new European tenders.<br>➔ From 2021, ask about suppliers' sustainable operational management:<br>◆ Appropriate working conditions, by investigating this for 100% of the new tenders and achieving a higher % every year.<br>◆ SROI (Social Return on Investment), by investigating this for 100% of the new tenders and achieving a higher % every year.<br>◆ Regional purchasing, by requesting a tender from one regional supplier, one preferred supplier and one other supplier for multiple private tenders and achieving a higher % every year.

2.1.1.3 People *Sustainable HR policy for a dynamic and vital organization*

The University's HR department will be responsible for the sustainable HR policy and its implementation. The key themes are Sustainable Employability and Diversity & Inclusion. The main focus areas of these are listed below.

Table S3: Roadmap ambitions regarding the people category.

➔ Staff who are skilled, motivated and fit (physically and mentally).<br>➔ A culture focused on development among staff and managers.<br>➔ Encouraging diversity and inclusion within the organization.

2.2 University of Groningen - CO_2e calculations

Data on sustainability is tracked by a CO_2 model and calculator created by the Green Office of the University of Groningen. It is used for calculating the carbon footprint of the University. Details and excel calculations are made available upon request.

Goal

Calculating the organizational carbon footprint is a complex process. The tools available for this are generally not flexible and offer no insight/background information about the calculations or the data obtained after the calculations. As a result, most CO_2 footprint calculations are performed by experts. The tool developed here is intended to offer non-experts the opportunity to calculate a carbon footprint.

What is a Carbon Footprint

With a carbon footprint, the total amount of greenhouse gas (Greenhouse Gas (GHG)) emissions of a company/institution/person is calculated in tons of CO_2 equivalents (CO_2e). The Intergovernmental Panel on Climate Change (IPCC) developed the CO_2e by determining the "Global Warming Potential" (GWP) for all types of emissions. (GWP is the environmental damage caused by 1 kg CO_2 = 1 kgCO_2e or 1 kg Nitrogen Oxide (NO) = 298 kg CO_2e). When determining the carbon footprint, we speak in terms of CO_2 that is used as a replacement for CO_2e. Carbon footprint emissions are classified into 3 focus areas (scopes):

> **Scope 1:** direct emissions caused by sources belonging to a company/organisation/person (*e.g.* emissions from commercial vehicles).
>
> **Scope 2:** defined as emissions that are caused as a result of the activities of a company/organization or person, but are not directly owned and/or controlled by the company/organization or person (*e.g.* the purchase of non-self-produced electricity).
>
> **Scope 3:** other indirect GHG emissions; this is an optional category in the GHG Protocol, these emissions occur as a consequence of the activities of a company/organization/person. The exact categories that are included in this model for each scope are explained and defined in the reporting information section.

Specifically for the University of Groningen the following list are the emission sources that are included in each scope. It explains the calculations, emission factors, data and assumptions taken for each source, which should be included in applicable sustainability reports.

For **scope 1**:

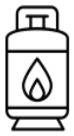

Natural Gas: Natural gas is the largest emission source for the UG.

Source: https://co2emissiefactoren.nl (2020)

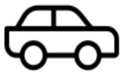

Company transport: These are the emissions of the fleet of cars that the UG owns and operates. The calculation is performed by multiplying the total liters of fuel used in the cars for each fuel type by their relevant emission factor.

Source: https://co2emissiefactoren.nl (2021)

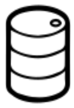

Refrigerants: Refrigerants have a GWP between 2000-4500 and are therefore required to be in the Carbon Footprint. Typically, the consumed amounts are very low compared to other categories. In the last five years, refrigerants had a 0 impact on the total emissions of the UG.

Source: https://co2emissiefactoren.nl (2021)

For **scope 2**:

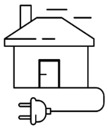

Purchased electricity: Since 2018, the UG only purchases green energy in the Netherlands which has an emission factor of 0.

Source: https://co2emissiefactoren.nl (2020)

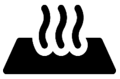

Purchased heat: The UG purchases some heat, which is multiplied by the relevant emission factor, the impact is very small compared to the total impact of the UG.

Source: https://co2emissiefactoren.nl (2016)

The following list includes the emission sources that are included in **scope 3** explaining the calculations, emission factors, data and assumptions taken for each source.

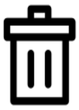

Waste: The model uses five waste categories: plastic, organic, paper, residual and hazardous waste. Each has its own emission factor. There exist many more categories of waste (TU Delft uses around 15 for example), but these five are the most significant for the UG.

Sources: previous model version (2015), TU Delft climate report (2019 & 2020 - https://www.tudelft.nl/en/2021/tu-delft/tu-delft-maps-own-co2-emissions-in-detail)

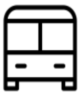

Commuting (employees & students): The CO_2-emissions of commuting are estimated for both students and employees. Important to note here is that most transport in Groningen involves green transport sources (biking, electric buses & public/ national trains by NS).

Employees: The calculation for employee commuting uses the number of full-time equivalents (FTEs), in combination with an assumption on the amount of working days per week and a total of 46 work weeks per year. In order to then calculate the actual emissions, an estimation is made about the percentage use of different transportation types (*i.e.* green transport, bus, car, *etc.*), and the corresponding distance that is travelled. This is multiplied by two (return trip) and combined with the data on FTEs, workdays and work weeks to calculate the final number.

Students: The calculation for students is similar to that of employees. We use the number of students, the number of days that they attend the University in person and the amount of working weeks in a year (40). And combine this with estimations on transport types and distances again. Sources: https://co2emissiefactoren.nl (2020 & 2021)

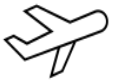

International travel (employees): For calculating the impact international travel of employees, the total amount of flight km's are multiplied with emission factors for flying. A distinction is made between regional and intercontinental flights because the kg CO_2 emission per flight km factor depends on the total length of a single flight. Also, international train km's are added in the 2022 model, since it also impacts carbon emissions.

Source: https://co2emissiefactoren.nl (2015)

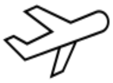

Student exchange travel: To estimate the impact of student exchange travel, we use the number of students going on exchange in a year (or an estimation). Following, these students are ordered by the continent of their exchange (using an estimation in % per continent). Each continent has its own emission factor, which is based on an assumption about the transport mode (car, train, flight) and data for each transport. In addition, the distance for each continent is fixed to a central point in the continent, which gives the amount of travel Km's. We assume a return journey with no stop-overs for flights.

Sources: https://co2emissiefactoren.nl (2015)

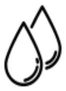

Purchased resources (water): Currently, the only purchased resource in the model is water. The amount of water used by the UG is multiplied by the relevant emission factor.

Source: https://co2emissiefactoren.nl, *via* the previous version of the model (2015)

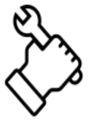

Purchased services: Currently, the model incorporates two types of purchased resources: data storage & construction. The reliability of the emission factors for these categories is low and should be taken with caution. The UG mainly uses data storage providers that pledges to be carbon neutral. The environmental impact of construction projects can be huge, but the reliability of data associated with them is low. It is advised to improve gathered data in future planning.

Data storage: Data storage is split up in two categories: green storage and non-green storage. Green storage is used by Microsoft and Google, non-green storage includes all other providers (https://www.google.com/about/datacenters/cleanenergy/ and https://www.microsoft.com/en-us/sustainability/energy)
Source: https://www.wired.com/story/amazon-google-microsoft-green-clouds-and-hyperscale-data-centers/ & https://medium.com/stanford-magazine/carbon-and-the-cloud-d6f481b79dfe

Construction: The existing data for construction emissions is currently too unreliable, emission factors are therefore set to 0. Construction projects can have a very large impact, so more reliable data needs to be acquired in the future.

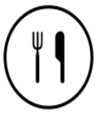

Food emissions: The food emission calculation depends on the availability of the data. The most reliable calculation would use data on the amounts of food consumed per food type in Kg, obtained from the food supplier. If this data is missing, an alternative calculation can be used that is based on the number of breakfasts/lunches/meals consumed in an entire year.

Food data per kg: The impact of the food is calculated by splitting up all food types in 30+- different food categories that have roughly similar emissions. The amounts of foods consumed for each category (in Kg) are then multiplied by the relevant emission factor for each of these categories. The data is obtained from www.foodfootprint.nl and based on Dutch market average data from the RIVM (2021, https://www.rivm.nl/en/dutch-national-food-consumption-survey).

Note: Keep in mind that consumption of products can be grouped into categories and that one of the products mentioned below can serve as the overall conversion factor of the category since their conversion factor is equal or nearly equal.

Food data per meal: Alternatively, an estimation can be made on the total amounts of meals (breakfasts/lunches/dinners) consumed at the UG catering facilities in a year. These values are then multiplied by the average impact of a Dutch breakfast/lunch/dinner. These averages are based on data from the Voedingscentrum. There is also an option to put in a vegetarian lunch/dinner, which have lower emissions.

Sources: www.foodfootprint.nl (2021, based on RIVM data), Voedingscentrum.

2.2.1 Environmental performance indicators

The Health, Safety, and the Environment Service (AMD) of the University of Groningen have developed a set of Environmental Performance Indicators (EPIs) to measure environmental performance. The EPIs enable comparison of the environmental performance of buildings, faculties or even universities and commercial companies. Since 1996, the University of Groningen has recorded these EPIs, enabling the formulation of new environmental performance aims.

Emission factors:

The emission factor categories match the input categories mentioned above. The input data is converted to CO_2 emissions *via* emission factors.

https://www.co2emissiefactoren.nl/
(main source used by organizations for scope 1, 2, 3 data from the Netherlands)

https://foodfootprint.nl/
(contains all food data (based on RIVM data from 2021)

https://www.tudelft.nl/2021/tu-delft/tu-delft-brengt-eigen-co2-uitstoot-gedetailleerd-in-kaart

(TU Delft emission report of 2020, which contains some specific scope 1, 2, 3 data that is not included on www.co2emissiefactoren.nl)

2.2.2 Institutional data

Table S4: Institutional data of the University of Groningen in 2023. *Adapted with permission from the Green Office, UG, from* https://www.rug.nl/about-ug/profile/facts-and-figures/.

Category	Type	Value	Unit
**Business context**	Name of company	University of Groningen	-
	Institutional sector	Education	-
	Reference year	2022	year
	Country of residence	Netherlands	-
	Foundation year	1614	year
**Business data**	Number of employees	6390	#
	Number of FTEs external employees	0	#
	Number of academic staff (FTE)	3750 (40% international)	#
	Number of full professors	400 (24% female)	#
	Number of PhD candidates	4400 (52% international)	#
	Number of PhD theses	640	#
	Number of research publications (excluding dissertations)	8500	#
	Number of patents	14	#
	Number of students	36681	#
	Number of international students	9900	#
	Number of faculties	11	#
	Number of alumni	140 000	#
	Number of Master's degree programs	120	#
	Number of Bachelor's degree programs	45	#
	Total Gross Floor Surface (GFS) of company ground	8.54E+05	m^2
	Total Gross Floor Surface (GFS) of company buildings	462651	m^2
	Number of company buildings (estimate)	120	#
	Number of cars	3	#

Disclaimer:

Due to missing numbers the carbon emissions of the University of Groningen are underestimated. The University strives to complete these numbers in the upcoming years. The electricity used is not necessarily produced green (*via* wind or solar) as it is delivered through Vattenfall© (https://www.vattenfall.nl/). The UG decides to buy green certificates produced through wind- and solar energy by vertiCer© (https://verticer.eu/nl/) to compensate for its footprint: From 2018 onwards 100% of the energy was compensated through these certificates until including 2022. However, in 2023 the number of green energy certificates were reduced to cover only 50% of the total energy consumption.

Included in the calculation:

Scope 1: gas, transport, refrigerants
Scope 2: electricity, heating
Scope 3:

- waste (residual, plastic, organic, paper, disposable coffee cup, electronic waste, glass waste, hazardous waste, other separated waste)
- student commuting
- employee commuting
- employee international travel (flights, international train, car)
- water consumption
- food and canteens
- office furniture of the Faculty of Science and Engineering

Missing data in Scope 3:

- *student exchange travel*
- *buildings and construction work*
- *furniture, IT, computers, and office equipment of other faculties*
- *high precision instruments, devices and laboratory equipment*

2.2.3 UG's CO_2e emissions, energy consumption and waste.

Detailed information on UG's policies on aspects such as travel, mobility, food, waste, buildings, energy can be found at https://www.rug.nl/about-ug/profile/facts-and-figures/duurzaamheid/beleid/. *The following data is adapted from these published reports.*

Table S5: CO_2e emissions of the University of Groningen in the years of 2015-2022 and progress overview. *Adapted from the calculations of the Green Office, UG.*

Year	2015		2016		2017		2018		2019		2020		2021		2022	
Category (CO_2e tons)	w/o offset	w/ offset	w/o offset	w/ offset	w/o offset	w/ offset	w/o offset	w/ offset	w/o offset	w/ offset	w/o offset	w/ offset	w/o offset	w/ offset	w/o offset	w/ offset
Total emissions	69 716	69 716	64 064	64 064	64 389	49 213	64 078	33 669	61 829	32 275	37 069	10 292	37 946	11 543	48 362	20 417
Scope 1	8120	-	8726	-	8626	-	8245	-	8076	-	7706	-	9055	-	7307	-
Scope 2	31 090	0	30 991	0	16 067	15 176	844	30 409	806	29 554	685	26 776	795	26 403	578	27 944
Scope 3	30 506	-	24 347	-	24 520	-	24 580	-	23 393	-	1902	-	1693	-	12 532	-
Emissions per publication (FSE)	n/a	n/a	n/a	n/a	173	132	n/a	n/a	159	83	n/a	n/a	n/a	n/a	97	41
Emissions per employee (6390)	11	11	10	10	10	8	10	5	10	5	6	2	6	2	8	3
Emissions per m^2 (462 651)	0.15	0.15	0.14	0.14	0.14	0.11	0.14	0.07	0.13	0.07	0.08	0.02	0.08	0.02	0.10	0.04
Electricity	30 307	0	30 133	0	15 181	15 176	0	30 409	0	29 554	0	26 776	0	26 403	0	27 944
Gas, heat, refrig.	8873	-	9533	-	9479	-	9064	-	8856	-	8372	-	9835	-	7796	-
Direct fuels	30	-	51	-	33	-	25	-	26	-	19	-	16	-	89	-
Food and canteens	5790	-	5874	-	5916	-	6236	-	6241	-	312	-	312	-	6239	-
Commuting	18 014	-	12 304	-	12 392	-	13 032	-	12 546	-	449	-	488	-	2774	-
Air travel	5977	-	5420	-	5463	-	4664	-	3944	-	649	-	339	-	2932	-
Purchased services	0.00	-	0	-	0	-	0	-	0	-	0	-	0	-	0	-
Purchased goods, water, furniture	419	-	421	-	426	-	339	-	335	-	322	-	329	-	330	-
Waste	306	-	328	-	324	-	309	-	327	-	170	-	225	-	258	-

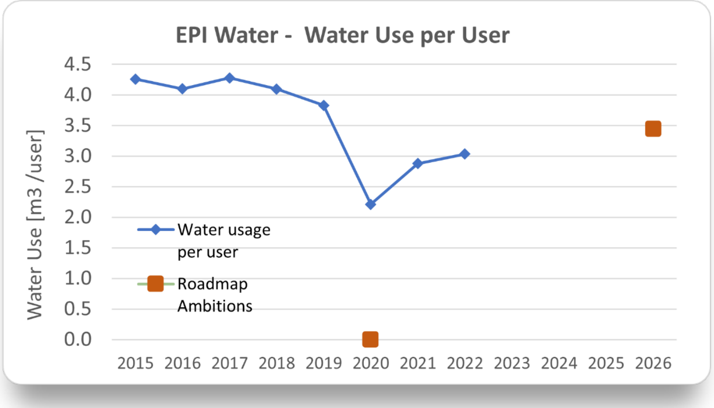

10% reduction of water use by 2026.

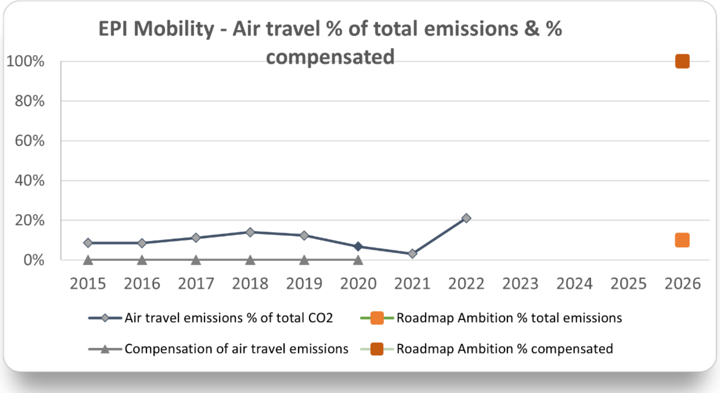

From 16% CO_2 emissions resulting from air travel in 2019, to 10% in 2026 (30% reduction).

100% CO_2 compensation for all air travel by 2026.

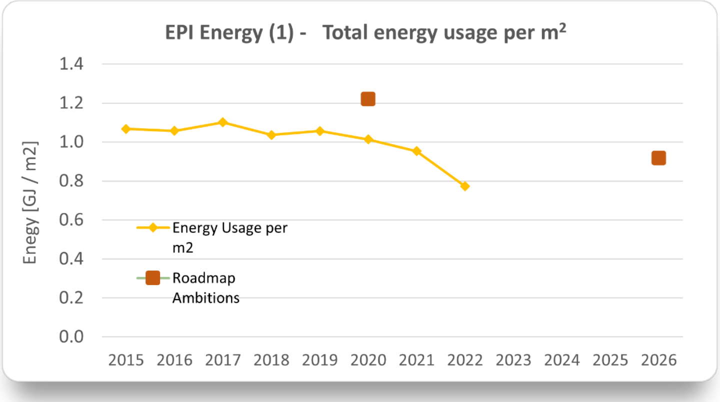

2% reduction of energy usage per year.

25% of energy from own renewable sources.

2.5% from solar panels.

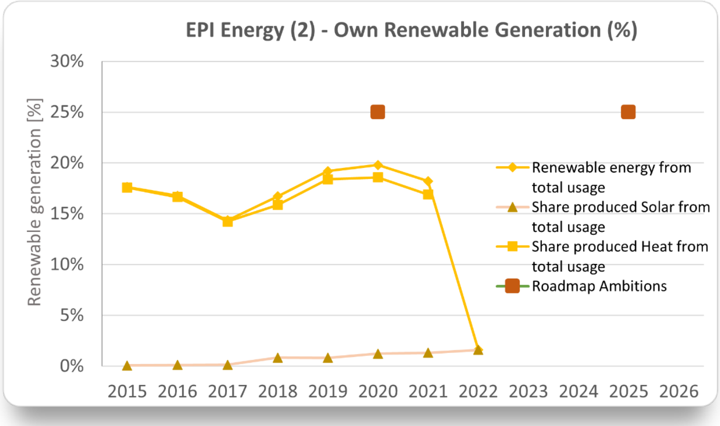

Figure S5: Water, mobility and electricity progress and targets.

Non-hazardous waste

Given that the layout of many buildings does not allow to use a large enough waste disposal container to collect the waste flows separately, bags are transported separately *via* large container. Once they have been taken to the collection point, they are taken out and thus separated again by waste flow. Plastic and residual waste are the exception. Rather than being separated, they are sent to the same company for processing.

More information on waste separation can be found at: https://www.rug.nl/about-ug/profile/facts-and-figures/duurzaamheid/beleid/afval and https://www.rug.nl/society-business/facility-services/afvalbeleid?lang=en.

Table S6: *Waste separation bin at the UG.*

	This should go in	This should not go in (and goes, except for glass, in other waste)
paper	• Dry and clean paper<br>• Newspapers & notebooks<br>• Window envelopes<br>• Clean tissues	• Dirty / wet paper<br>• Drink cartons<br>• Greasy pizza boxes<br>• Sandwich wrappings from the canteen
plastics	• Clean plastic bags / bowls / yoghurt cups*<br>* Scraped empty is sufficient. It does not need to be rinsed with water.	• Fruit nets (e.g. mandarines)<br>• Chips bags<br>• Aluminum packaging<br>• Cans<br>• Pens<br>• Bowls with food scraps
organic food waste	• Fruit and vegetable waste<br>• Food scraps	• Tea bags<br>• Food scraps in wrappings or containers
other waste	• Dirty packaging<br>• Aluminum packaging (chips bags, granola bar wrappings)<br>• Everything you're unsure about<br>• Everything with a paper / plastic mix	• Glass
cups	• Empty coffee cups	• Coffee cups with liquid in it<br>• Coffee cups with other waste in it<br>• Soup bowls from the canteen

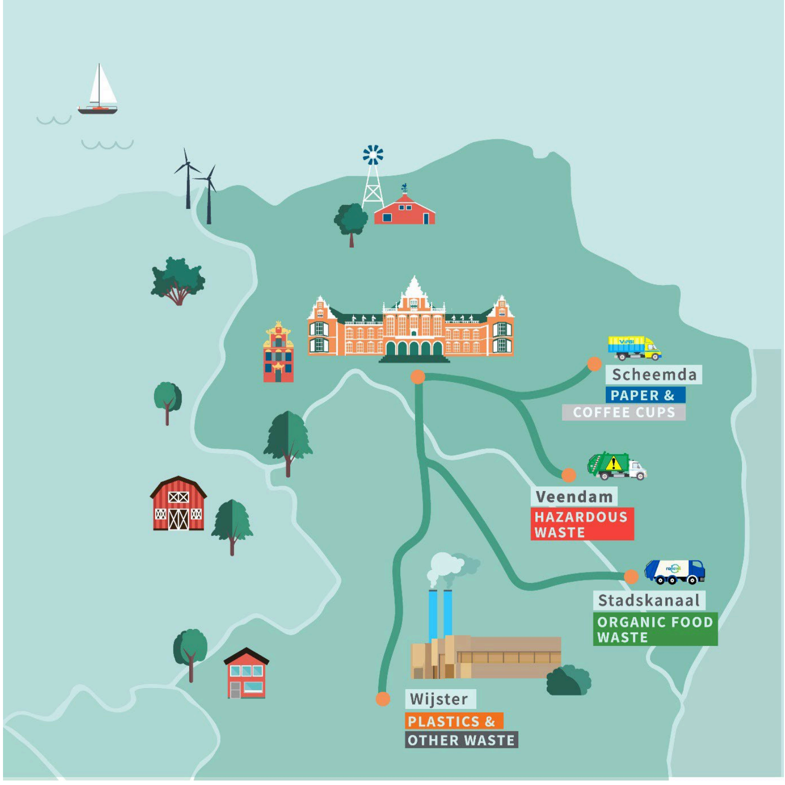

Figure S6: The university's waste separation, collection sites and processing companies and their locations in 2023. *Reproduced with permission from the Green Office, UG.*

2.3 Faculty of Science and Engineering (FSE) - CO_2e calculations, electricity, and gas consumption.

2.3.1 Institutional data

Table S7: Institutional data, size, area and number of employees of FSE and each building.

Category	Type	Value	Unit
**Business context**	Name of company	Faculty of Science and Engineering	-
	Institutional sector	Education and research	-
	Reference year	2023	year
	Country of residence	Netherlands	-
**Business data**	Number of employees	2889	#
	Number of active lab researchers	1762	#
	Senior staff	318 (11%)	#
	Junior research staff (PhD, postdocs, technicians)	1300 (45%)	#
	Teachers/lecturers	145 (5%)	#
	Temporary unemployed staff (guest researchers)	462 (16%)	#
	Support staff	665 (23%)	#
	Total Gross Floor Surface (GFS) of company buildings (NVO)	140 782	m^2
	Total Gross Floor Surface (GFS) of company buildings (FNO)	94 950	m^2
	Total Gross Floor Surface (GFS) of company buildings (WO)	80 067	m^2
	Number of lab spaces	557	#
	Number of company buildings (estimate)	9	#

Building	Area (m^2)	Area (m^2)	Area (m^2)
	**NVO**	**FNO**	**WO**
Nijenborgh 4 (NB4)	49 944	32 272	28 468
Linnaeusborg (NB7)	37 772	25 373	21 084
Bernoulliborg	12 295	8591	5889
EAE	12 032	6040	4775
ZL25	12 450	7576	5530
Herdershut	506	389	151
Lutjewad	175	169	43
A. Deusinglaan	10 871	10 871	10 871
FEB	4738	3669	3256
**Sum**	**140 782 m^2**	**94 950 m^2**	**80 067 m^2**

BVO	NVO	NO		FNO		VVO
Bruto vloer-oppervlakte	Netto vloer-oppervlakte	Nuttig vloer-oppervlakte	Afdeling vloer-oppervlakte	Vloeroppervlakten van werkplekken, inclusief circulatieruimte	50% *)	Ver-huur-baar vloer-opper-vlakte 85% *)
				Supplementaire afdelingsruimte	5% *)	
			Speciale ruimten, kantine, archief, vergaderruimte, repro		15% *)	
			Sanitaire ruimten, werkkasten, garderobe			
		Verkeersruimte (gangen, trappenhuizen, liften, entree, hallen)			20% *)	
		Technische installatieruimten				
	Constructieoppervlakte (wanden, kolommen)				10% *)	

*) De in de rechterkolommen gegeven waarden zijn indicatief

2.3.2 FSE CO_2e emissions

All data on the energy, electricity and gas consumption per faculty can be obtained from https://rug.erbis.nl/. *The following sections are adapted and calculated through D. Jager, N. Elzinga and T. Freese and are published with permission of the Faculty of Science and Engineering, University of Groningen.*

Table S8: CO_2e emissions of FSE in the years 2017, 2019 and 2022, correlated to number of publications (*Source: Scopus and Pure*), employees and area.

Year	2017		2019		2022	
Category (CO_2e tons)	w/o offset	w/ offset	w/o offset	w/ offset	w/o offset	w/ offset
Total emissions	28 255	22 978	26 642	16 700	25 419	15 996
Scope 1	5017	-	4466	-	4236	-
Scope 2	10 862	5585	10 214	271	9618	271
Scope 3	12 376	-	11 963	-	11 564	-
# of publications at FSE	372		390		498	
Emissions per publication (FSE)	76	62	68	43	51	32
Emissions per employee (2889)	10	8	9	6	9	6
Emissions per m^2 (140 782)	0.20	0.16	0.19	0.12	0.18	0.11
Electricity	10 554	5277	9943	0	9423	0.00
Gas, heat, refrig.	5310	-	4725	-	4413	-
Direct fuels	15	-	12	-	18	-
Food and canteens	2675	-	2822	-	2821	-
Commuting	5603	-	5672	-	5637	-
Air travel	2036	-	1457	-	1149	-
Purchased services	0	-	0	-	0	-
Purchased goods, water, furniture	377	-	322	-	319	-
Waste	88	-	92	-	40	-

2.3.3 FSE electricity and gas consumption

Table S9: Electricity and gas consumption of FSE from 2014 – 2023. Normalized gas usage per temperature degree days measured in Eelde.

Year	Electricity usage per year (kWh)	Gas usage per year (m^3)	Temperature degree days in Eelde, 01.01 until 31.12.	Gas usage per year / temperature degree days (m^3 / temperature degree day)
2014	22 827 268	1 978 348	2656.26	744.79
2015	22 995 709	2 383 369	2886.40	825.72
2016	23 088 363	2 537 471	3062.20	828.64
2017	23 412 207	2 650 570	2937.90	902.20
2018	23 072 079	2 459 234	2895.69	849.27
2019	23 394 175	2 360 201	2840.69	830.85
2020	21 607 915	2 320 715	2732.35	849.35
2021	22 974 499	2 698 832	3053.15	883.95
2022	22 390 579	2 234 141	2765.34	807.91
2023	20 800 040	1 968 902	2698.22	729.70

Table S10: Monthly electricity consumption at FSE from 2019 – 2023.

Electricity usage per month (kWh)	2019	2020	2021	2022	2023
Jan	2 125 407	2 069 513	2 076 099	1 980 005	1 845 972
Feb	1 922 753	1 914 191	1 872 175	1 829 056	1 747 838
Mar	2 113 221	1 911 268	2 016 038	2 010 771	1 940 200
Apr	1 892 935	1 528 089	1 897 199	1 849 245	1 719 319
May	1 919 025	1 659 662	1 876 851	1 879 707	1 775 522
Jun	1 830 311	1 784 979	1 833 445	1 837 297	1 730 038
Jul	1 901 399	1 619 741	1 879 311	1 835 135	1 696 247
Aug	1 890 149	1 590 643	1 832 733	1 830 914	1 657 700
Sep	1 869 985	1 829 063	1 838 885	1 775 835	1 626 315
Oct	1 978 193	1 889 016	1 917 018	1 830 467	1 709 920
Nov	1 939 352	1 849 950	1 932 644	1 848 522	1 704 063
Dec	2 011 444	1 961 801	2 002 101	1 883 626	1 646 907
**Sum**	**23 394 174**	**21 607 916**	**22 974 499**	**22 390 580**	**20 800 041**

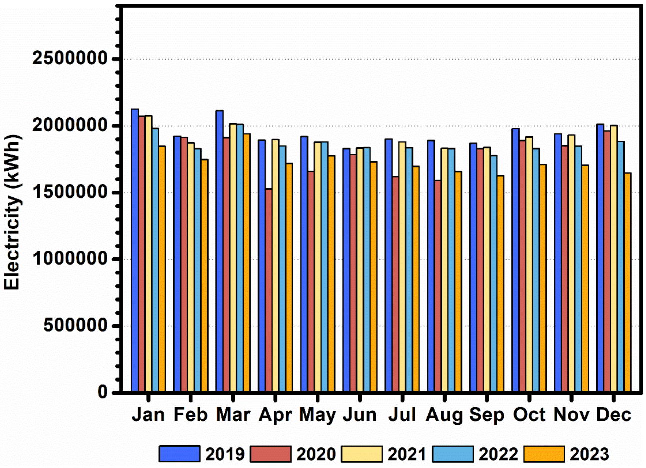

Figure S7: Monthly correlation of electricity usage at FSE in kWh from 2019-2023. *Adapted with permission from the Faculty of Science and Engineering, source:* https://rug.erbis.nl/.

Table S11: Monthly correlation of gas consumption at FSE of the years 2019-2023.

Gas usage per month (m^3)	2019	2020	2021	2022	2023
Jan	366 810	301 452	403 473	339 874	274 853
Feb	255 094	286 962	345 449	301 368	239 325
Mar	256 159	296 131	324 114	296 918	253 418
Apr	205 448	200 656	303 239	226 982	202 231
May	174 840	176 137	207 214	131 232	136 730
Jun	76 917	84 827	77 326	81 361	68 710
Jul	77 528	90 332	61 287	63 494	61 251
Aug	59 245	54 587	77 604	51 648	50 236
Sep	104 531	102 864	94 370	103 672	52 873
Oct	188 554	182 919	197 065	125 148	135 528
Nov	301 989	229 220	268 598	209 390	228 478
Dec	293 085	314 628	339 094	303 055	265 269
**Sum**	**2 360 200**	**2 320 715**	**2 698 833**	**2 234 142**	**1 968 902**

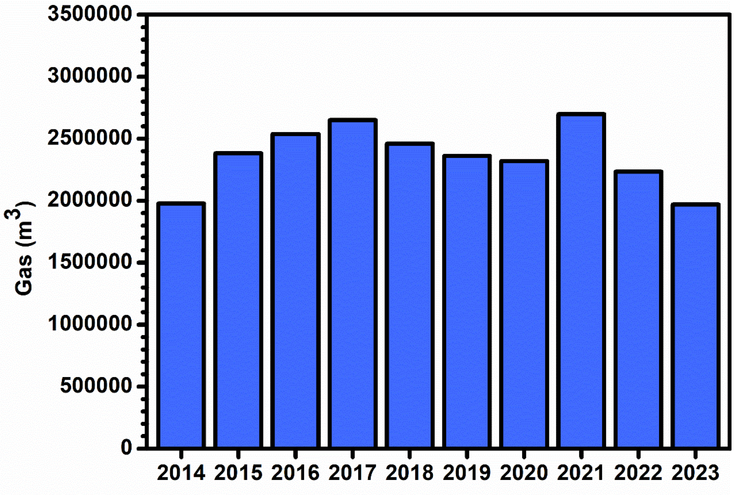

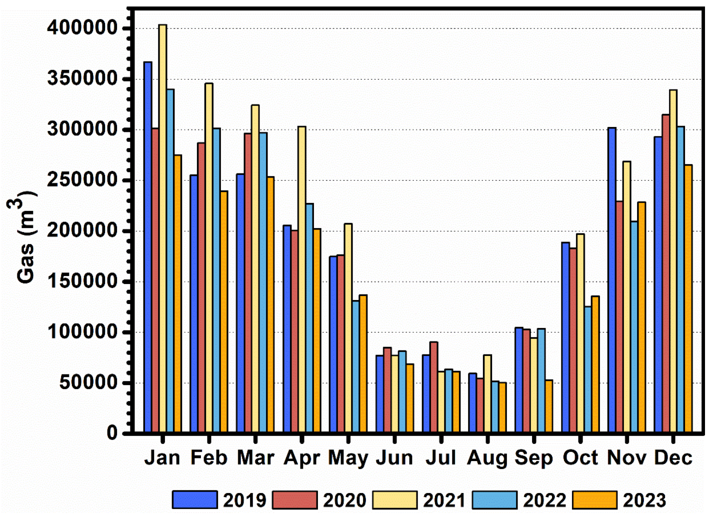

Figure S8: Gas consumption in m³ at the Faculty of Science and Engineering from 2014-2023 (top). Monthly correlation of gas consumption at FSE of the years 2019-2023 (bottom). Although activity on the campuses was reduced in 2020 and 2021 (COVID-19), research practices and laboratory work continued. Instead of applying a full lock-down, researchers worked in shifts. Thus, the energy consumption as well as heating (gas usage) continued. Also, air ventilation (*i.e.* electricity) was increased to allow for a safe working environment. *Adapted with permission from the Faculty of Science and Engineering, source:* https://rug.erbis.nl/.

Table S12: Baseload energy consumption of all science buildings at FSE versus the usage by the users, *i.e.* running the building (heating, cooling, ventilation, lighting etc.) *versus* the activities in the building (science activities, instrument use, computers etc.).

Baseload energy consumption	Gas (m^3)	Electricity (kWh)
Nov-22	209390	1848522
Feb-23	239325	1747838
Average	224357.5	1798180
Winter break 2022 (two weeks, no users)	102333	798977
Normalization to a month (4 weeks)	204666	1597954
**Baseload energy consumption**	**91.2%**	**88.9%**

We furthermore calculated, the baseload energy consumption of all science buildings at FSE *versus* the usage by the users, *i.e.* running the building (heating, cooling, ventilation, lighting etc.) *versus* the activities in the building (science activities, instrument use, computers etc.). By utilizing the data from the 2022 winter break (see **Section 2.7.4**) and normalizing to 4 weeks, we could compare the energy consumption in November 2022 and February 2023 (*i.e.* with users at similar outside temperatures, thus degree days as December/January) with the winter break (*i.e.* no users) to estimate the baseload energy consumption. It should be noted that air ventilation and fume hoods were not yet reduced or turned off, resembling still numbers associated to 'user activities':

Laboratories:

Temperature within *generally climate-controlled* laboratories will be reduced to a technically minimal and responsible level. Ventilation is not being adjusted.

Ventilation and room temperature of *specifically climate-controlled* areas, such as climate cells, laser laboratories, animal facilities etc. remain unchanged.

Offices:

Ventilation and the room temperature in offices will be reduced to a technically minimal and responsible level.

Both laboratories and offices:

All staff are requested to switch off all equipment, including computers, that can be safely switched off during the winter holiday closure.

At the Faculty of Science and Engineering the baseload energy consumption was calculated at 91.2% for gas usage and 88.9% for electricity usage. We furthermore consulted an energy & sustainability advisor, indicating that the baseload energy usage averages at 80%, thus 20% of energy consumption is usually associated to users. Other institutions show that about 75% of energy for a science building is used in just keeping the building operational (*i.e.* the Estates Department responsibility) and that about 25% is due to the activity of the researchers. Such knowledge allows for directing of funds and efforts to maximize sustainability impact.

2.4 Travel at the University of Groningen

Analysis of travel data 2016-2022

2.4.1 Introduction

Data of business travel of the University of Groningen (UG) have been recorded in annual reports of the travel agent since 2016. The data files that are provided by the travel agent contain:

- The departure and destination of flights;
- The total distance of the flight;
- The CO_2 emissions related to the flight.

Regarding the last point, it is unclear how the travel agent calculated this. It is also not clear whether or not the emissions include only the emissions of carbon dioxide, or if it encapsulates all emissions (and hence represents the emissions in CO_2 equivalents). Lastly, it is unknown if different emissions for business and economy class flights are accounted for. Since business class seats take up more space on an airplane, they cause the plane to transport passengers with lower efficiency, leading to higher emissions per passenger. Business class tickets cause 1.3 to 4 times as much emissions as economy class.[1,2] For the sake of transparency and consistency, we will therefore calculate the CO_2 emissions from flights by using the same emission factors that are used in the CO_2 footprint program of the UG. These are listed in **Table S13** below.

Table S13: Flight distance categories and emission factors.

Type	Distance	Emission factor (g CO_2e km^{-1})
Short	< 800 km	300
Middle	800 - 2500 km	200
Long	> 2500 km	150

Analysis of the flight data is conducted because the total emissions due to business travel by airplane need to be recorded to calculate the annual CO_2 footprint of the University. It is also used to estimate the impact of the newly updated travel policy of the UG will have in terms of emission reduction, and to calculate a CO_2 budget for each faculty/department.

The data files provided for each year do not all contain the same data. For the years 2016 and 2020, only a summarized version was available, where only the total number of flights, flight distance and emissions were reported. For other years the file contained information on each individual flight that was booked. In this category, 2018 stood out as it only listed the distance and emission of each flight. The remaining years (2017, 2019, 2021 and 2022) contained complete information of each flight and also mentioned through which department the flight was booked. Thus, in parts of this analysis, only the years 2017, 2019 and 2022 were utilized. 2021 is omitted in these cases due to COVID-19 pandemic measures.

2.4.2 Results - University

Table S14 depicts the distribution of short, middle- and long-distance flights in terms of the number of flights, the distance, and the emissions:

1. The short flights (*i.e.* the flights that are affected by the UG business travel policy) cause less than 10% of the total emissions;
2. The long flights, which account for ~30% of the total number of flights, contribute to approximately 70% of the total emissions.

Table S14: Distribution of different flight types over the total number of flights, distance, and emissions.

	Number	Distance	Emissions
Short	28.1%	4.9%	9.4%
Middle	42.2%	18.1%	22.4%
Long	29.8%	77.0%	68.2%

Table S15: Most visited countries per year and the number of trips taken to that country.

Entry	2017		2019		2022		Total	
1	United States	670	United Kingdom	603	Italy	477	United States	1542
2	United Kingdom	656	United States	519	United States	353	United Kingdom	1527
3	China	515	Italy	459	Spain	327	Italy	1318
4	Italy	382	Spain	286	United Kingdom	268	Spain	966
5	Spain	353	Germany	198	Portugal	221	China	714
6	Germany	226	China	185	Greece	177	Germany	542
7	France	218	France	166	Thailand	139	France	495
8	Switzerland	213	Switzerland	153	Ireland	120	Portugal	495
9	Denmark	150	Philippines	149	Germany	118	Switzerland	463
10	Austria	146	Portugal	145	France	111	Greece	384

2.4.3. Results - Faculty and departments

Figure S9 depicts the total flight distance per faculty or department and the figure in the main article shows the distance divided by the total number of employees of that faculty or department (expressed in FTEs). An overview of the number of FTEs (permanent appointments) for each faculty/department was requested at the HR-department of the University. Note that for Campus Fryslân was founded in 2018, resulting in lower carbon emissions. Both figures (main article and ESI) are sorted in ascending order of the 2022 data.

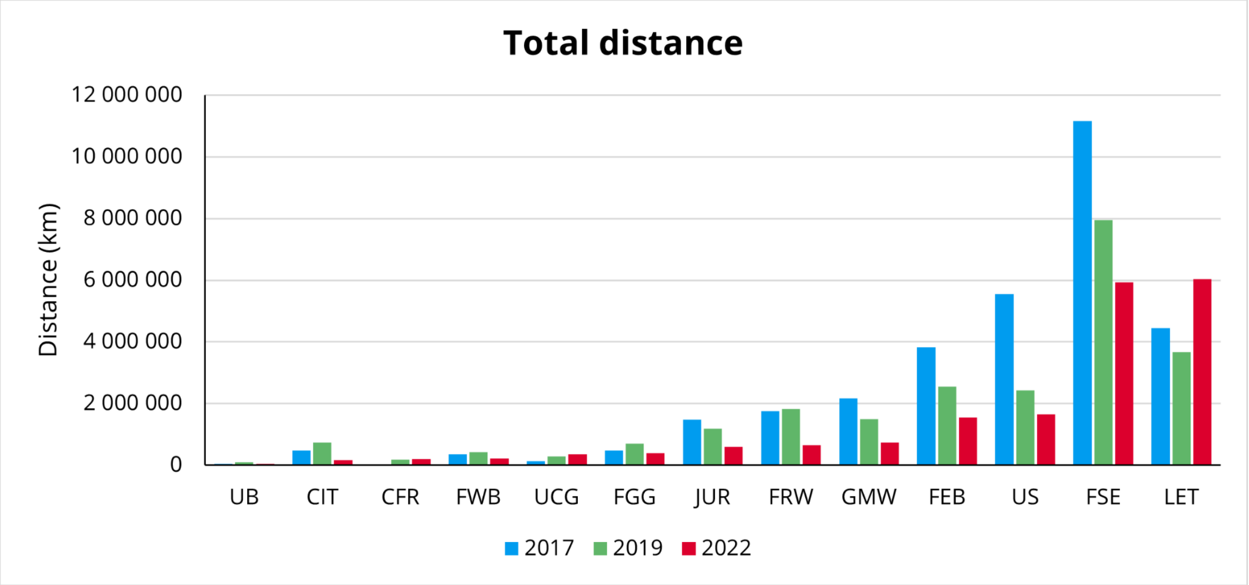

Figure S9: Total flight distance of each faculty or department of UG.

Table S16: List of abbreviations of faculties and departments.

CFR	Campus Fryslân
CIT	Center for Information Technology
FEB	Faculty of Economy and Business
FGG	Faculty of Theology and Religious Studies (from Sep 2023: Fac. of Religion, Culture and Society)
FRW	Faculty of Spatial Sciences
FSE	Faculty of Science and Engineering
FWB	Faculty of Philosophy
GMW	Faculty Behavioural and Social Sciences
JUR	Faculty of Law
LET	Faculty of Arts
UB	University Library
UCG	University College Groningen
US	University Services<br>ℹ Since October 2022 the following departments were fused to form one support organization: Facility Management, Property and Investment Projects and the Bureau of the University

Acknowledgements: *The travel data has been adapted with permission from the Green Office, University of Groningen and we greatly acknowledge G. Boesjes (Green Office) for collection and interpretation of data. The following images on the air travel data have been reproduced with permission of the Green Office and data visualized through M. van den Boom (prev. Green Office).*

AIRTRAVEL BUREAU OF THE UNIVERSITY

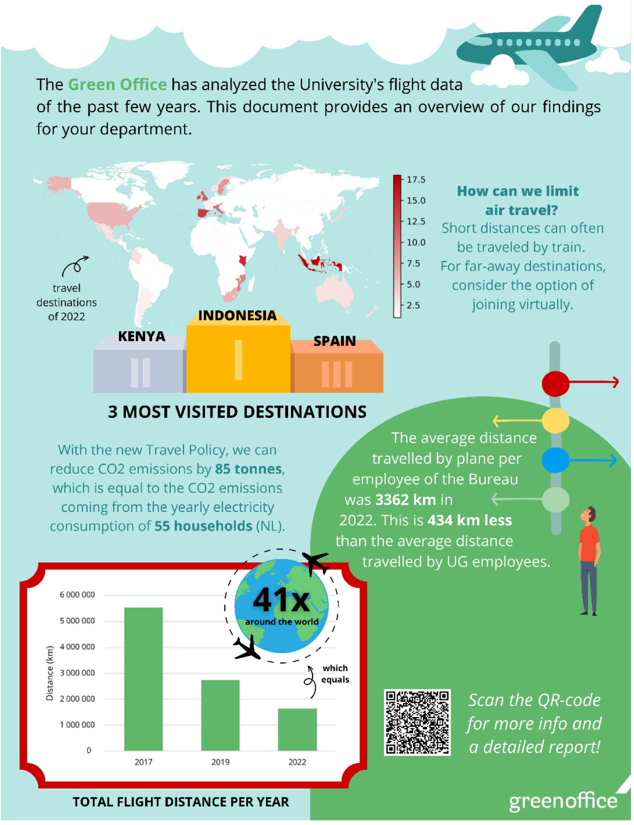

AIRTRAVEL CAMPUS FRYSLÂN

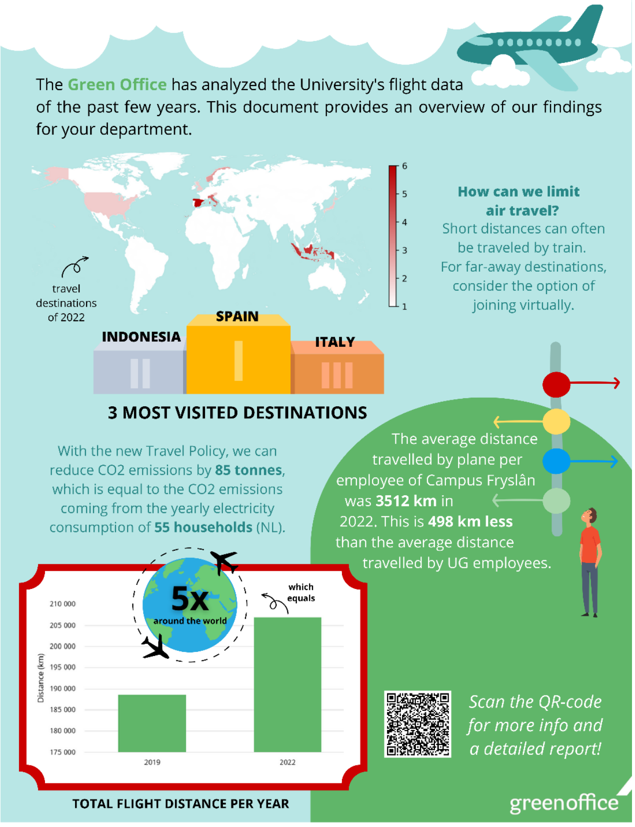

AIRTRAVEL CENTER FOR INFORMATION TECHNOLOGY

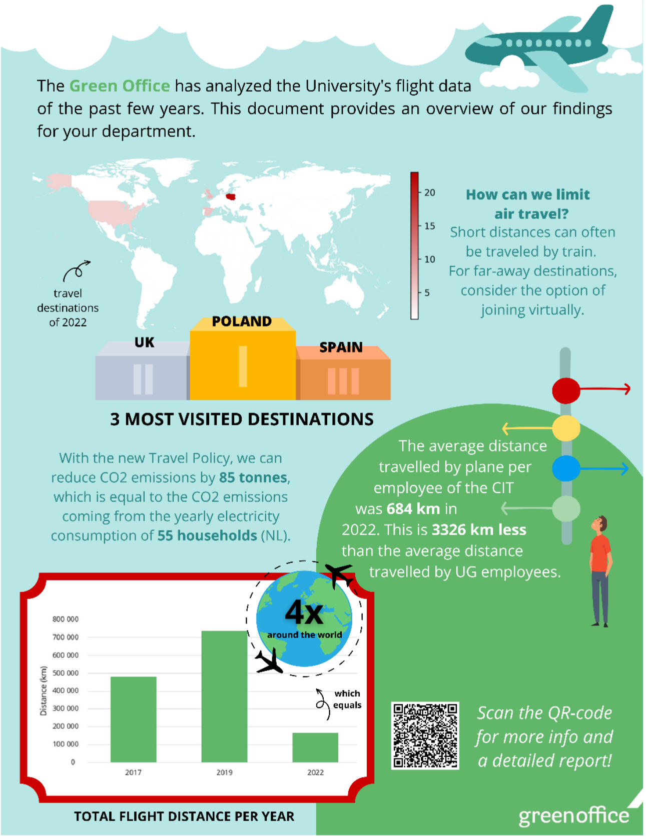

AIRTRAVEL FACULTY OF ECONOMICS & BUSINESS

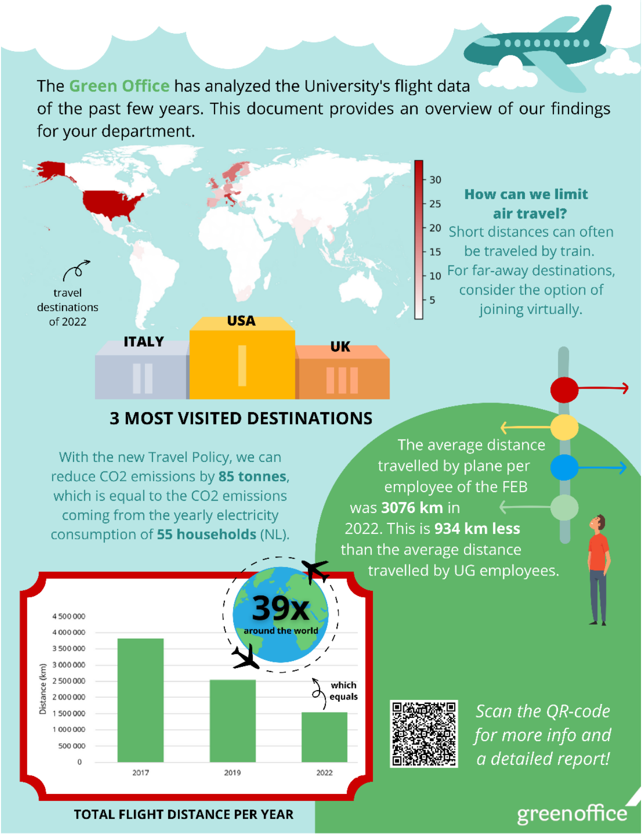

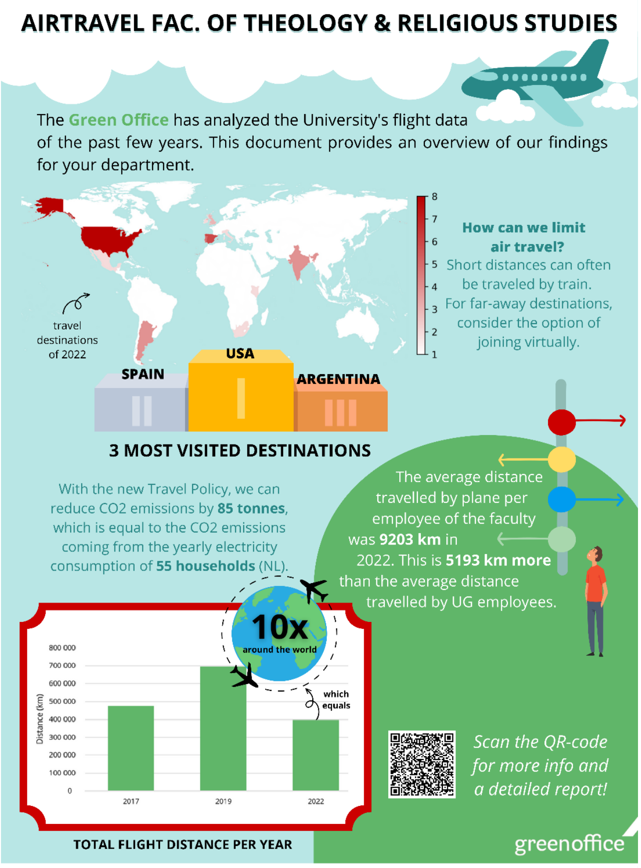

AIRTRAVEL FAC. OF THEOLOGY & RELIGIOUS STUDIES
The Green Office has analyzed the University's flight data of the past few years. This document provides an overview of our findings for your department.
travel destinations of 2022
How can we limit air travel?
Short distances can often be traveled by train. For far-away destinations, consider the option of joining virtually.
SPAIN
USA
ARGENTINA
3 MOST VISITED DESTINATIONS
With the new Travel Policy, we can reduce CO2 emissions by 85 tonnes, which is equal to the CO2 emissions coming from the yearly electricity consumption of 55 households (NL).
The average distance travelled by plane per employee of the faculty was 9203 km in 2022. This is 5193 km more than the average distance travelled by UG employees.
10x around the world
which equals
Distance (km)
2017
2019
2022
TOTAL FLIGHT DISTANCE PER YEAR
Scan the QR-code for more info and a detailed report!
greenoffice

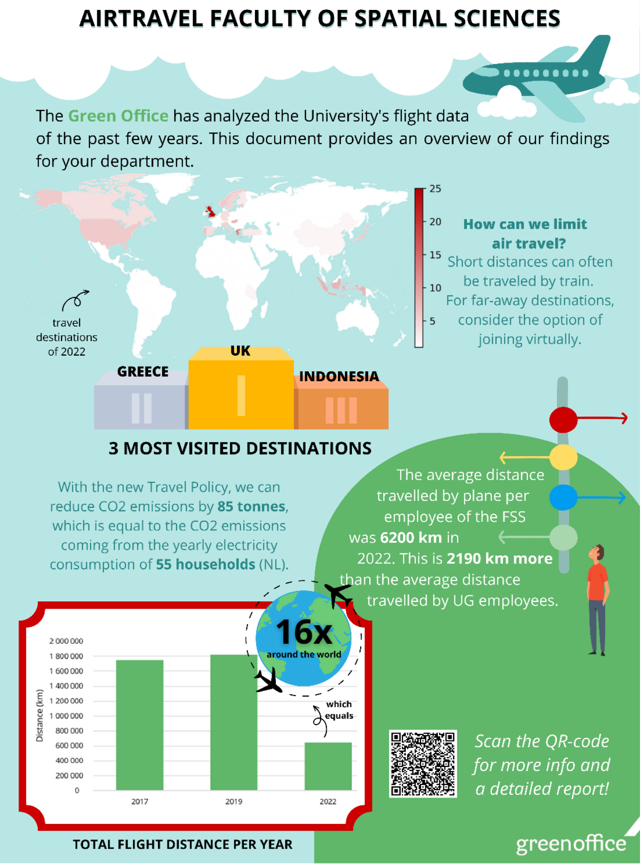

AIRTRAVEL FACULTY OF SPATIAL SCIENCES
The Green Office has analyzed the University's flight data of the past few years. This document provides an overview of our findings for your department.
travel destinations of 2022
How can we limit air travel?
Short distances can often be traveled by train. For far-away destinations, consider the option of joining virtually.
GREECE
UK
INDONESIA
3 MOST VISITED DESTINATIONS
With the new Travel Policy, we can reduce CO2 emissions by 85 tonnes, which is equal to the CO2 emissions coming from the yearly electricity consumption of 55 households (NL).
The average distance travelled by plane per employee of the FSS was 6200 km in 2022. This is 2190 km more than the average distance travelled by UG employees.
16x around the world
which equals
Distance (km)
2017
2019
2022
TOTAL FLIGHT DISTANCE PER YEAR
Scan the QR-code for more info and a detailed report!
greenoffice

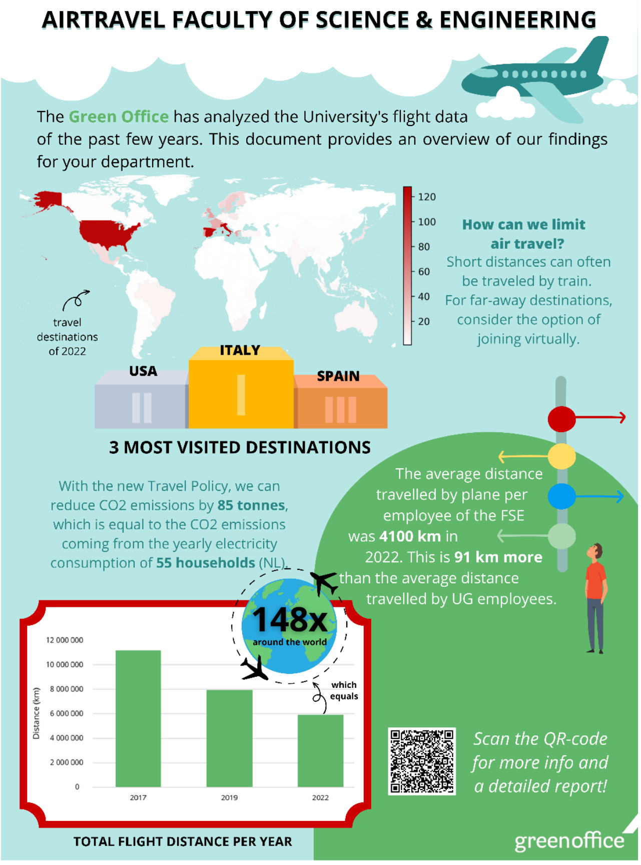

AIRTRAVEL FACULTY OF SCIENCE & ENGINEERING
The Green Office has analyzed the University's flight data of the past few years. This document provides an overview of our findings for your department.
travel destinations of 2022
How can we limit air travel?
Short distances can often be traveled by train. For far-away destinations, consider the option of joining virtually.
USA
ITALY
SPAIN
3 MOST VISITED DESTINATIONS
With the new Travel Policy, we can reduce CO2 emissions by 85 tonnes, which is equal to the CO2 emissions coming from the yearly electricity consumption of 55 households (NL).
The average distance travelled by plane per employee of the FSE was 4100 km in 2022. This is 91 km more than the average distance travelled by UG employees.
148x around the world
which equals
Distance (km)
2017
2019
2022
TOTAL FLIGHT DISTANCE PER YEAR
Scan the QR-code for more info and a detailed report!
greenoffice

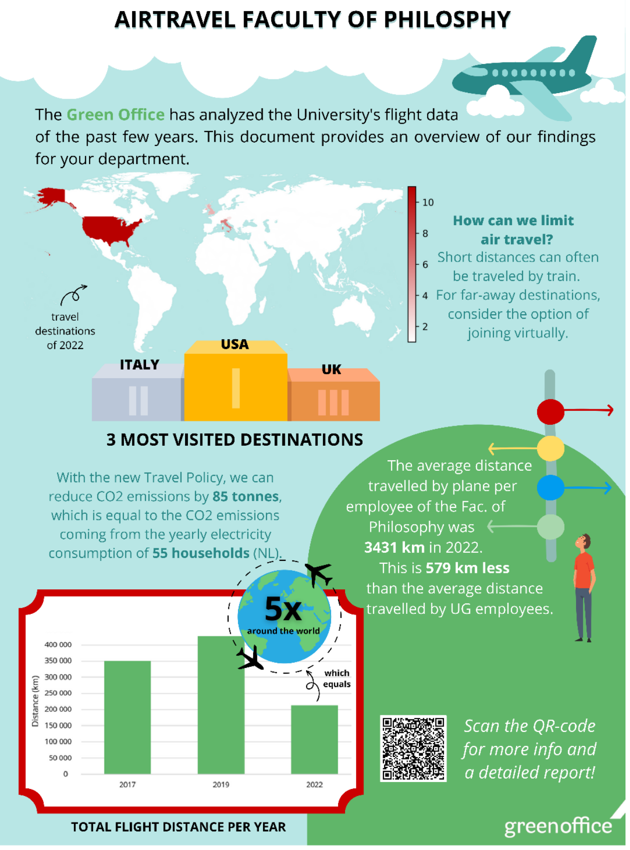

AIRTRAVEL FACULTY OF PHILOSPHY
The Green Office has analyzed the University's flight data of the past few years. This document provides an overview of our findings for your department.
travel destinations of 2022
How can we limit air travel?
Short distances can often be traveled by train. For far-away destinations, consider the option of joining virtually.
ITALY
USA
UK
3 MOST VISITED DESTINATIONS
With the new Travel Policy, we can reduce CO2 emissions by 85 tonnes, which is equal to the CO2 emissions coming from the yearly electricity consumption of 55 households (NL).
The average distance travelled by plane per employee of the Fac. of Philosophy was 3431 km in 2022. This is 579 km less than the average distance travelled by UG employees.
5x around the world
which equals
Distance (km)
2017
2019
2022
TOTAL FLIGHT DISTANCE PER YEAR
Scan the QR-code for more info and a detailed report!
greenoffice

AIRTRAVEL FAC. OF BEHAVIOURAL & SOCIAL SCIENCES

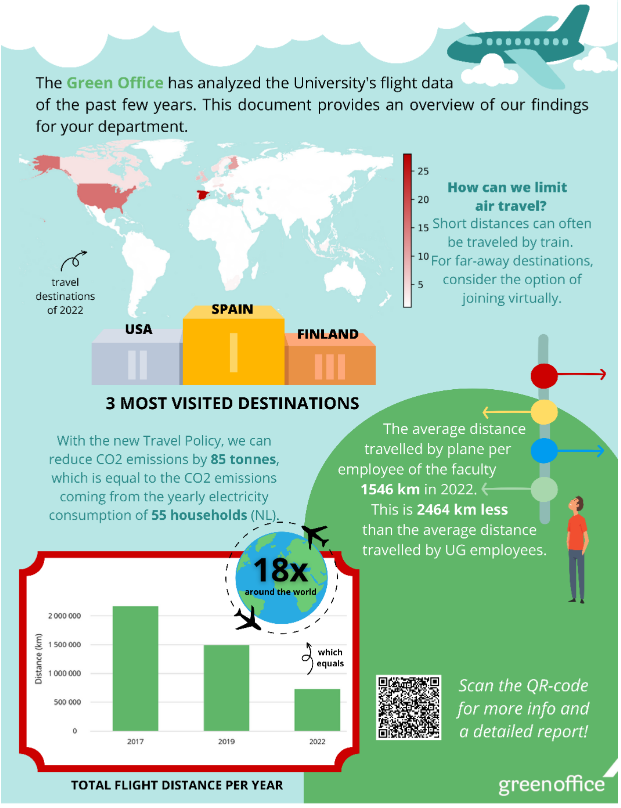

AIRTRAVEL FACULTY OF LAW

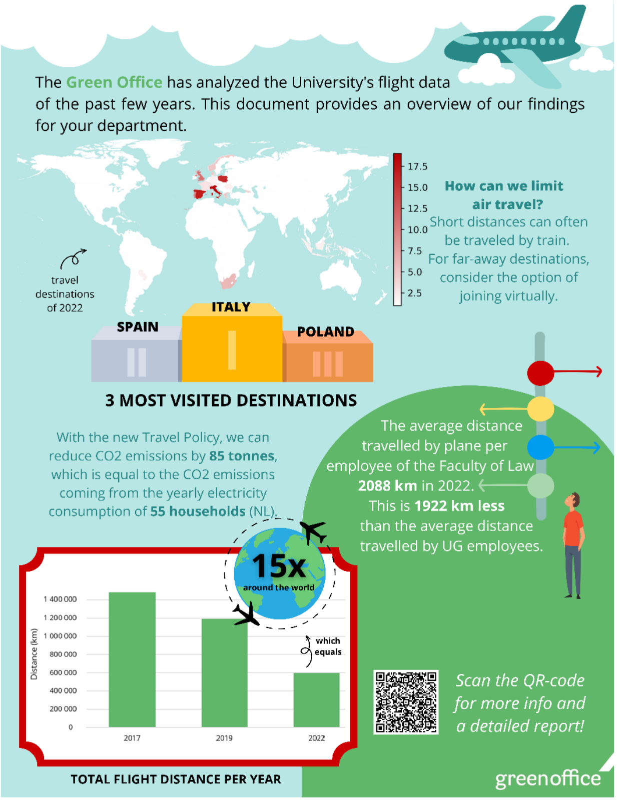

AIRTRAVEL FACULTY OF ARTS

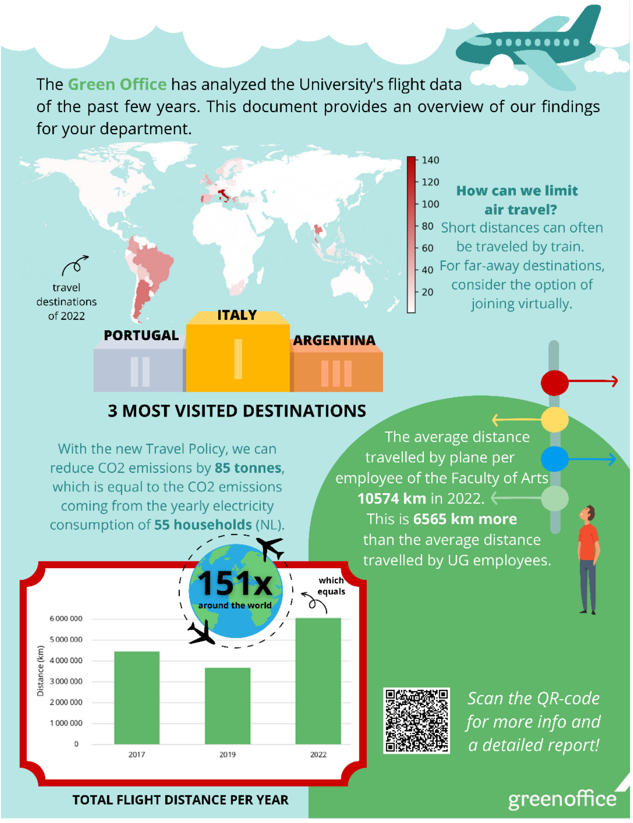

AIRTRAVEL UNIVERSITY COLLEGE GRONINGEN

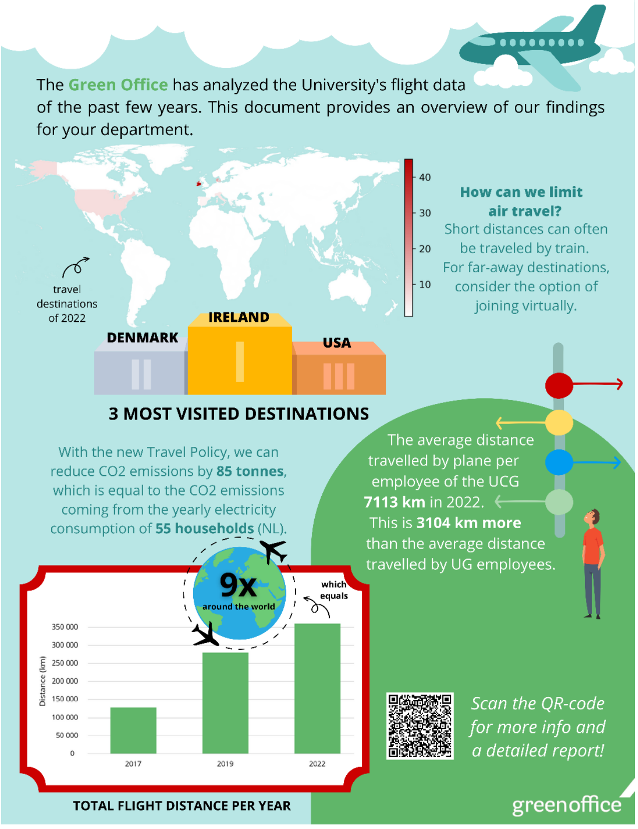

2.5 Health, Safety & Sustainability (HSE) reports FSE

2.5.1 HSE report 2021

2.5.1.1 Energy consumption (environmental performance indicators)

Although the COVID-19 pandemic was a major issue in 2021, research in the laboratories continued at an almost normal level. As of May 2021 onwards, full research work was allowed to resume.

The national advice to air as much as possible and open windows and doors as much as possible during the working day contributed to higher gas consumption. This is especially true since it was 3.3% colder than the long-term average over the whole year in 2021 (https://www.climate.gov/news-features/understanding-climate/climate-change-global-temperature). Old buildings such as NB4 are particularly sensitive to this.
Research activities at Lutjewad in Hornhuizen have increased considerably. This is clearly reflected in the electricity consumption. After being stable for many years, it has now almost doubled. But as mentioned, this is entirely work-related.
A positive step that has been made is that the new built Herdershut on Schiermonnikoog is equipped with a heat pump for heating the building. Due to the construction of the new Herdershut, electricity consumption has increased fivefold this year, but next year it will be back to its previous, stable low level.

The main part of FSE's natural gas consumption takes place in Nijenborgh 4 and the Linnaeusborg (steam humidification for the animal testing facility). The remaining part of the heat energy in FSE buildings is generated electrically by means of heat pump systems linked to heat/cold storage in the ground.

In LB heating system consists mainly of concrete core activation and underfloor heating. Concrete core activation is such a slow control that it is not switched based on time, which would result in an uncontrollable installation. The underfloor heating is also hardly ever switched on the basis of time.
In terms of air treatment, the air treatment systems are generally switched to low at night and to high again during the day, whereby the air treatment in teaching rooms is really switched off when the buildings are not open.

In NB4 the heating is generally switched on during the day and off in the evening, but this varies per building section. This is because the heating is not controlled in the same way in every building section (*via* the building management system BMS). There is also a lot of variation in the air conditioning, some systems run from Monday to Friday from 07:00 to 20:00 and then switch off, others run 24/7.

The air treatment systems and heating are only parts of the total energy consumption, as various other installations, such as chillers are operated as well. A significant part of the heat pump capacity is used for cooling. In fact, the FSE buildings that use heat pumps for cooling and heating have an annual surplus of heat because, there is more demand for cooling than for heating.

In short, turning the thermostat down a few degrees could save energy for NB4. For all the other buildings, it is much more complex.
The tables below depict the total consumption of gas and electricity for all Zernike buildings managed by FSE.

Table S17: FSE total gas and power consumption.

Cumulative	2017	2018	2019	2020	2021
Gas consumption (m^3)	2 644 325	2 459 234	2 355 395	2 320 714	2 698 832
Power consumption (kWh)	23 367 239	23 072 196	23 410 661	21 607 914	22 962 523

The following figures are EPI scores of power and natural gas consumptions (adjusted for floor area).

Table S18: FSE's energy use (gas + electricity) in EPI scores (GJ / m^2).

Cumulative	2017	2018	2019	2020	2021
Gas + Gas consumption (GJ/m^2)	2.48	2.41	2.29	2.15	2.35

Nijenborgh 4	2017	2018	2019	2020	2021
Gas consumption (GJ/m^2)	1.51	1.13	1.13	1.17	1.37
Power consumption (GJ/m^2)	1.40	1.49	1.48	1.42	1.47
**Total**	2.92	2.62	2.61	2.59	2.84

Linnaeusborg	2017	2018	2019	2020	2021
Gas consumption (GJ/m^2)	0.40	0.32	0.22	0.14	0.17
Power consumption (GJ/m^2)	2.36	2.54	2.56	2.29	2.51
**Total**	2.76	2.86	2.78	2.43	2.68

Bernoulliborg	2017	2018	2019	2020	2021
Gas consumption (GJ/m^2)	0.11	0.08	0.07	0.07	0.02
Power consumption (GJ/m^2)	0.84	0.92	1.03	0.93	0.92
**Total**	0.95	1.00	1.10	0.99	1.00

Energy Academy Europe (EAE)	2017	2018	2019	2020	2020
Gas consumption (GJ/m^2)	0.00	0.00	0.00	0.00	0.00
Power consumption (GJ/m^2)	0.74	1.02	0.61	0.57	0.60
**Total**	0.74	1.02	0.61	0.57	0.60

Locatie Zernikelaan 25 (LZL25)	2017	2018	2019	2020	2021
Gas consumption (GJ/m^2)	-	-	-	0,15	0,30
Power consumption (GJ/m^2)	-	-	-	0,76	0,22
**Total**	-	-	-	0,91	0,52

2.5.1.2 Waste MPI (kg per m^2)

The amount of commercial and hazardous waste is expressed in total amount of kg and in EPI (kg/m^2 of floor area). The cumulative scores for waste are provided below for all the buildings managed by FSE (Nijenborgh 4, Bernoulliborg, Energy Academy Europe, Locatie Zernikelaan 25 and Linnaeusborg (LB)) with a total floor area of 131 302 m^2.

Table S19: FSE's hazardous waste production.

Cumulative	2018	2019	2020	2021
Hazardous waste (kg)	61 400	59 700	52 989	71 662
Haz. waste (EPI, kg / m^2)	0.52	0.50	0.40	0.55

Table S20: FSE's hazardous waste production per location.

Location	2018	2019	2020	2021
Hazardous waste NB4	-	-	40 990	55 237
Hazardous waste NB7	-	-	9876	14701
Hazardous waste LZL25		-	2123	1724

In both NB4 and NB7, the amount of hazardous waste increased significantly. The increase for NB4 could be explained by the fact that since the beginning of 2021, the HSE department has initiated a major clean-up operation to dispose of old chemicals in anticipation of the move to a new building in 2024.
In the LB an additional floor (9th) has been renovated and opened for research activities. Conducting mostly synthetic organic chemistry experiments, the research group produces about the same or more than the organic chemical group on the eighth floor. This explains the significant increase in hazardous waste in the LB.

Table S21: FSE's commercial waste production.

Cumulative	2018	2019	2020	2021
Commercial waste (kg)	144 580	150 584	110 667	66 522
Com. waste (EPI, kg/m^2)	1.22	1.27	0.84	0.51

Table S22: Commercial waste in detail.

Cumulative	Amount (kg) 2020	Amount (kg) 2021
Paper	38 165	34 629
Coffee cups	105	120
Electrical equipment	5122	2628
Furniture	3500	9260
Mesh and bedding	19 080	-
Bulky industrial waste	13 360	-
Flammable industrial waste	1150	-
B-wood	5180	1320
Residual waste	52 460	-

2.5.1.3 Surface water emissions

FSE was granted a licence under the Surface Water Pollution Act (Wvo, https://www.helpdeskwater.nl/secundaire-navigatie/english/waterlinks-and/) for both the Physics and Chemistry complex and Linnaeusborg. On the basis of the duty of care specified in this licence, the faculty is required to justify the disposal of a predefined series of hazardous substances on a regular basis. The licence specifies norms for concentrations of volatile halogenated hydrocarbons (VOX compounds), volatile aromatics (BTEX compounds), extractable organic chlorine in water (EOX) and heavy metals (zinc, copper, lead, nickel and tin, as well as mercury for Nijenborgh 4).

The number of samples taken complies with the frequencies specified for the individual components in the Wvo. The table below summarizes the average concentrations at the various sample points per location over the past seven calendar years.

Table S23: Average concentrations measured at the Physics and Chemistry complex (Nijenborgh 4).

Component	VOX (µg/l)	BTEX (µg/l)	EOX (µg/l)	Zn, Cu, Cr, Pb, Ni, Sn (µg/l)	Mercury (µg/l)
(Norm)	*(≤100)*	*(≤100)*	*(≤100)*	*(≤3000)*	*(≤3)*
Average for 2021	<10	1,3	3,0	613	1,0
Average for 2020	<10	4,7	3,4	756	0,5
Average for 2019	20	3,3	1,1	521	0,7
Average for 2018	6,4	3,0	<100	456	0,3
Average for 2017	9,4	0,7	<100	180	0,2
Average for 2016	13,8	4,3	<100	297	0,2
Average for 2015	19,4	4,5	<100	283	0,1

Table S24: Average concentrations measured at Linnaeusborg (NB7).

Component	VOX (µg/l)	BTEX (µg/l)	Zn, Cu, Cr, Pb, Ni, Sn (µg/l)
(Norm)	*(≤100)*	*(≤100)*	*(≤3000)*
Average for 2021	<10	83	7062
Average for 2020	19	76	82718
Average for 2019	14	34	6933
Average for 2018	20	22	1982
Average for 2017	5	12	2267
Average for 2016	6	86	1849
Average for 2015	9	120	665

All concentrations for Nijenborgh 4 are well below the norm, although the one for Mercury doubled. This is caused by a one-time high concentration of 4 µg/L in the first quarter in sample well no 2. And there was also an elevated value of 2.3 µg/L in the last quarter. The cause of this can no longer be determined. Based on European regulations, HSE repeatedly urges all scientists to use as little mercury and mercury-containing products as possible and to return unused mercury or mercury-containing chemicals as much as possible

For the Linnaeusborg, the value of the heavy metals was dropping. There were renovations going on in the building in 2019-2020 causing the spike. The higher values were caused by copper and zinc. Chromium, lead, nickel, and tin (environmentally and health wise much more damaging metals) never exceeded the standard and were in most even below the detection limit, in terms of concentration.

The increased value for BTEX (the volatile aromatic components taken together) was caused by a one-time high value of 1200 ug/L in the first quarter in sample well 10. Immediately after this value was reported, action was taken by the HSE department by approaching the departments that discharge *via* this sample well. After all, the policy regarding liquid hazardous waste at FSE is that has to be disposed of in dedicated, labelled containers and not the *via* drain.

2.5.1.4 Biological safety organization

Many activities involving biological agents take place within the Faculty of Sciences and Engineering (FSE). Examples of these agents are pathogenic organisms, genetically modified organisms (GMO) and animal by-products. Safety and management play a major role in these activities. This is mainly to prevent exposure of employees to these agents and to ensure that used biological agents remain within the laboratories to protect humans, animals, and the environment outside the university against exposure to these agents.

The Biosafety Organization (BO) plays an important role in formulating policy on these safety issues, all based on the Dutch legislation and regulations. Besides formulating policy, the BO is also responsible for its implementation within FSE and the UG. In doing so, they manage the use of biological agents and ensure that employees carry out their work in accordance with the applicable protocols and procedures. They also provide advice and training on the formulated policies and procedures to staff and policy officials within the faculty and university.
The BO currently consists of two biological safety officers (BSO) who work closely with the biological safety coordinator within the Health, Environment and Sustainability Department (AMD) of the university. The work mainly focuses on the laws and regulations concerning the handling of genetically modified organisms and biological agents, the use of animal by-products and the access to and the fair sharing of benefits relating to the use of genetic resources (the Nagoya Protocol).

Genetically modified organisms 'old permits'
Due to a change in the law concerning the use of GMOs in 2013, all permits issued before this change (the 'old permits') must be re-evaluated to see whether the activities comply with the new legislation.

Nagoya Protocol
The Nagoya Protocol is a legislation concerning the access to genetic resources originating outside the Netherlands and the fair distribution of benefits relating to their use with the country of origin. To implement this legislation within the UG, the BO has, in cooperation with the legal department of the UG, drawn up the necessary internal guidelines, general procedures and a handbook in recent years.
In preparation of performing inventories on this legislation within FSE research groups, lots of time was spent on understanding the content of this complicated unfinished legislation. In this way, the BO can properly advise researchers within the UG and assist them better in taking the right follow-up steps to comply with the legislation. A digital assessment tool has been developed as well to enable researchers to perform assessments user-friendly and independently whether genetic sources used are in scope of this legislation.

2.5.1.5 Sustainability FSE

The faculty values the sustainability of its operations and focuses on the factors of people and the environment. During renovations, attention is paid to ergonomics, both in the lab and in the office. In addition, during renovations from a technical point of view, the most sustainable solution is always chosen, even if it is financially less favourable.
The sustainability factor is also considered during European tendering processes. Manufacturers and suppliers can score if they drive electric vehicles for example or use less packaging material or can demonstrate in some other way that they are working in an environmentally friendly way.
Employees are also regularly reminded that sustainability starts with them and that it is therefore important that fume cupboard windows are closed as much as possible when leaving the lab and that lighting and, where possible, electrical equipment are turned off. The use of an own mug is encouraged.
In addition, the Health, Safety & Environment department participates in a project of the Green Office (called “the Green Lab”) with the aim to reduce the amount of waste in laboratories and to separate/recycle plastic waste.

2.5.2 HSE report 2022

2.5.2.1 Energy consumption at FSE (environmental performance indicators)

From a sustainability perspective several energy consumption measures have been undertaken. An energy-saving team was created at both UG and faculty level to try to save as much energy as possible as fast as possible. This team consists of technicians from the electrical, mechanical and water management fields, staff from Vastgoed & Beheer and staff with knowledge of the specific buildings at user-, safety- and technical level. In addition, all kinds of initiatives within institutes and research groups to save energy (and develop ideas for this) were developed. The faculty has appointed a Projectsecretaris duurzaamheid FSE who keeps track of this and who is able to bring groups together.

In order to save energy in a (reasonably) simple way in the short term, the following measures have been taken and projects started:

- Closing of FSE buildings between Friday Dec. 24th and Monday Jan. 9th.
- The comfort times in terms of heating and lighting have been adjusted and made uniform for the FSE buildings. Roughly speaking, the comfort times in the FSE buildings are between 8 am and 8 pm, during weekdays. During this period, rooms in these buildings are heated to 19°C (instead of 22°C) in winter and cooled to 26°C (instead of 21°C) in the summer.
- The (excessive) lighting in the common areas of buildings (where it was easily feasible) was also reduced to night mode during comfort times.
- All building-wide technical installations were checked for the most efficient operation possible.
- During Christmas and the New Year, most of the UG buildings were closed for two weeks plus one day during which heating and lighting were reduced to weekend mode. During this closure, users were asked to also turn off all equipment that is normally (due to having to keep it operational) on at night and on weekends.
- During the Christmas closure, all local water heaters (hot water supplies) were turned off. When they were started up, they were equipped with a time switch; outside of comfort times, the water heaters are going to be turned off. During this startup, employees were asked to be critical of need for hot water at that location.
- Employees were asked to turn off computers and peripherals as much as possible when leaving the office. Likewise, chargers etc. should be unplugged.
- Users of -80 freezers have been asked to raise the temperature to -70 C if possible.
- Users of energy-guzzling equipment have been asked to replace them with modern, more energy-efficient ones whenever possible. The FSE board is willing to assist financially with this.
- All lamps are replaced by LED-lamps during corrective maintenance.
- An initiative is underway to apply insulating foil to all windows in NB4, a poorly insulated building. Users are also asked to place reflective foil behind the radiators.
- An initiative is in progress to dispose old chemicals, old appliances and (-80) freezers and refrigerators. Disposal is at the expense of FSE in order to stimulate and motivate groups. This with the aim to reduce the number of fireproof cabinets and chemical cabinets, freezers, and refrigerators.
- An initiative is currently being developed to remove 4 large boilers in basement NB4 and provide the laboratories with local, small boilers as a hot water supply in desired areas. In fact, the 4 large boilers are kept at 70°C 24/7. A lower temperature is not possible due to expected leakage and from a legal point of view (Legionella).
- The initiative to remove all local food preparers and other household electrical equipment in offices, coffee rooms and in-between spaces in NB4 in particular was initially rolled out but withdrawn after many objections. Until the move to the Feringa Building, which has multiple pantries on all floors, this equipment will not be removed.

In all this, it should be noted that in terms of climate and air treatment of all laboratories, clean rooms and animal housing, no changes have been made to the settings and times of these installations. The safety of employees and the living conditions of animals within FSE are extremely important and therefore unaffected. No concessions are made in this area, so to speak. Certain research/equipment is also considered when determining the minimum temperature in the buildings.

Some projects are being worked out to achieve longer-term savings that require larger investments:
- A process is already ongoing for European tendering for replacement of lighting in all UG buildings for LED lighting.
- Solar panels will be installed wherever possible to generate our own electricity.
- Similarly, solar boilers will probably be installed wherever possible to generate hot water.
- After the initial relocation of the research groups in NB4, particularly from parts 17 and 18, the remaining spaces will be used in such a way that heating and air handling will be as efficient and cost-effective as possible.
- Ventilation of general areas according to demand.
- Optimizing of data storage.
- Using a measurement plan to gain insight into where, when, and how much is consumed.
- Explore alternatives to the steam plant for air treatment of the animal facility.

Table S25: FSE total gas and power consumption.

Cumulative	2018	2019	2020	2021	2022
Gas consumption (m^3)	2 459 234	2 355 395	2 320 714	2 698 832	2 235 349
Power consumption (kWh)	23 072 196	23 410 661	21 607 914	22 962 523	22 392 866

The next figures are EPI scores of power and natural gas consumptions (adjusted for floor area).

Table S26: FSE's energy use (gas + electricity) in EPI scores (GJ / m^2), cumulative.

Cumulative	2018	2019	2020	2021	2022
Gas + Gas consumption (GJ/m^2)	2.41	2.29	2.15	2.35	2.35

Table S27: FSE's energy use (gas + electricity) in EPI scores (GJ / m^2), per building:

Nijenborgh 4	2018	2019	2020	2021	2022
Gas consumption (GJ/m^2)	1.13	1.13	1.17	1.37	1.12
Power consumption (GJ/m^2)	1.49	1.48	1.42	1.47	1.44
Total	2.62	2.61	2.59	2.84	2.56

Linnaeusborg	2018	2019	2020	2021	2022
Gas consumption (GJ/m^2)	0.32	0.22	0.14	0.17	0.16
Power consumption (GJ/m^2)	2.54	2.56	2.29	2.51	2.41
Total	2.86	2.78	2.43	2.68	2.57

Bernoulliborg	2018	2019	2020	2021	2022
Gas consumption (GJ/m^2)	0.08	0.07	0.07	0.02	0.03
Power consumption (GJ/m^2)	0.92	1.03	0.93	0.92	0.90
Total	1.00	1.10	0.99	1.00	0.93

Energy Academy Europe (EAE)	2018	2019	2020	2020	2022
Gas consumption (GJ/m^2)	0.00	0.00	0.00	0.00	0.00
Power consumption (GJ/m^2)	1.02	0.61	0.57	0.60	0.61
Total	1.02	0.61	0.57	0.60	0.61

Locatie Zernikelaan 25 (LZL25)	2018	2019	2020	2021	2022
Gas consumption (GJ/m^2)	-	-	0.15	0.30	0.22
Power consumption (GJ/m^2)	-	-	0.76	0.22	0.28
Total	-	-	0.91	0.52	0.50

2.5.2.2 Waste MPI (kg per m2)

The amount of hazardous waste is expressed in total amount of kg and in EPI (kg / m^2 of floor area). The cumulative scores for waste are provided below for all the buildings managed by FSE (Nijenborgh 4, Bernoulliborg, Energy Academy Europe, Locatie Zernikelaan 25 and Linnaeusborg) with a total floor area of 131 302 m^2.

Residual waste from the UG is collected and processed by Attero. Post-separation of *e.g.* plastics and drinks cartons is conducted. Organic material is subsequently separated from the waste, which is fermented to produce Green Gas. This gas is of natural gas quality and is offered to the gas network in the Netherlands. About 225 tonnes of residual waste was delivered and about 22.5 tons of were fermented, *i.e.* 10%.
After the burning process, iron is extracted from the bottom ashes. This is burnt clean due to the cremation process and can be offered directly for recycling (4.6 tons in 2022).
During the incineration process of the remaining waste the energy generated is supplied to the neighbouring industry in the form of heat. The company also has a turbine that converts the heat (steam) into electricity. This electricity is then offered to the electricity grid.

Table S28: FSE's hazardous waste production in total.

**Cumulative**	2018	2019	2020	2021	2022
Hazardous waste (kg)	61400	59700	52989	71662	108987
Haz. waste (EPI, kg/m^2)	0.52	0.50	0.40	0.55	0.83

Table S29: FSE's hazardous waste production per location.

**Location**	2018	2019	2020	2021	2022
Hazardous waste NB4	-	-	40990	55237	82661
Hazardous waste NB7	-	-	9876	14701	26272
Hazardous waste LZL25		-	2123	1724	54

In both NB4 and the Linnaeusborg, the amount of hazardous waste increased significantly in 2022. Since the beginning of 2021, the HSE department has initiated a major clean-up operation to dispose of old chemicals, which is still being continued up to time of writing. It started in NB4 in 2021 and in the beginning of 2022 also in Linnaeusborg (will continue until mid-2024).

2.5.2.3 Surface water emissions

FSE was granted a license under the Surface Water Pollution Act (Wvo) for both the Physics and Chemistry complex and Linnaeusborg. Based on the duty of care specified in this license, the faculty is required to justify the disposal of a predefined series of hazardous substances on a regular basis. The license specifies norms for concentrations of volatile halogenated hydrocarbons (VOX compounds), volatile aromatics and hydrocarbons (BTEX compounds), extractable organic chlorine in water (EOX) and heavy metals (zinc, copper, lead, nickel, and tin, as well as mercury for Nijenborgh 4).

The number of samples taken complies with the frequencies specified for the individual components in the Wvo. The table below summarizes the average concentrations at the various sample points per location over the past eight calendar years.

The so-called Environment Law ("Omgevingswet") is currently being developed by the central government. This law is likely to take effect from 1 January 2024. In this Environment Law, 26 existing laws on spatial planning, nature protection, environment, quality of life, water, building regulations and much more will be combined.

Table S30: Average concentrations measured at the Physics and Chemistry complex (Nijenborgh 4).

Component	VOX (µg/l)	BTEX (µg/l)	EOX (µg/l)	Zn, Cu, Cr, Pb, Ni, Sn (µg/l)	Mercury (µg/l)
(Norm)	*(≤100)*	*(≤100)*	*(≤100)*	*(≤3000)*	*(≤3)*
Average for 2022	<1	4,3	0,2	337	0,3
Average for 2021	<10	1,3	3,0	613	1,0
Average for 2020	<10	4,7	3,4	756	0,5
Average for 2019	20	3,3	1,1	521	0,7
Average for 2018	6,4	3,0	<100	456	0,3
Average for 2017	9,4	0,7	<100	180	0,2
Average for 2016	13,8	4,3	<100	297	0,2
Average for 2015	19,4	4,5	<100	283	0,1

Table S31: Average concentrations measured at Linnaeusborg (NB7).

Component	VOX (µg/l)	BTEX (µg/l)	Zn, Cu, Cr, Pb, Ni, Sn (µg/l)
(Norm)	*(≤100)*	*(≤100)*	*(≤3000)*
Average for 2022	<1	7	12887
Average for 2021	<10	83	7062
Average for 2020	19	76	82718
Average for 2019	14	34	6933
Average for 2018	20	22	1982
Average for 2017	5	12	2267
Average for 2016	6	86	1849
Average for 2015	9	120	665

Due to an improvement in analytical technique, the lower limit of detection has now become 1 µg / L for VOX.

2.5.2.4 Biological safety organization

Many activities using biological agents take place within the Faculty of Sciences and Engineering (FSE). Examples of these agents are pathogenic organisms, genetically modified organisms (GMO) and animal by-products. Safety and management play a major role in these activities to prevent exposure of employees to these agents and to ensure that used biological agents remain within the laboratories to protect humans, animals, and the environment outside the university against exposure to these agents. In 2022 no biological incidents have been reported.

The AMD is coordinating the Biological Safety Organization of the UG. Together with the Biological Safety Officers (BVF's) of the FSE, the coordinator is responsible for the legal care of biological safety in the field of:

- Genetically modified organisms (GMO).
- The Nagoya Protocol.
- Biological agents.
- Animal by-products.

Full details of the activities in 2022 are accounted for by the annual report for Biological Safety; "Jaarverslag 2022, Zorgsysteem Biologische Veiligheid Rijksuniversiteit Groningen".

2.5.2.5 Sustainability

The Faculty of Science and Engineering (FSE) is developing various initiatives and workgroups on sustainability, including the labs. The Green Labs group focuses on reducing waste, energy, and water consumption in labs with the LEAF program (a user-friendly software program that helps scientists perform their lab work in a more climate-friendly manner).
Early 2022, a good step was made in the European tendering process for the disposal of hazardous waste. The WIVA drums used until then for biological waste, animal by-products and human waste (ADL1) were replaced for a type made of recycled plastic.

2.6 FSE is going green

In June 2022 the Faculty Board of FSE launched a new sustainability program: ‘FSE is going green’. Project groups were formed around ideas on sustainability. All projects will be initiated and performed in cooperation with the UG Green Office. *All images and figures in the following sections are reproduced with permission of the Faculty of Science and Engineering and Green Office, University of Groningen (Source: https://www.rug.nl/research/fse/sustainability-research/sustainable-research-practices?lang=en).* *We acknowledge and thank E. W. Zinkstok (prev. Science Linx) for visualizing data and communication.*

Sustainability projects at FSE:

(1) Food and beverages at canteens and events

(2) Buildings and energy use

(3) Green labs initiative

(4) Travel behavior of the staff and (ERASMUS) students

(5) Housing of staff and room use

(6) Green logistics at the Faculty

(7) Grounds around the Faculty buildings

(8) Creating sustainability awareness for staff and students

(9) Teaching students about sustainability

(10) Making sustainability research more visible

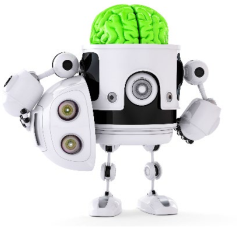

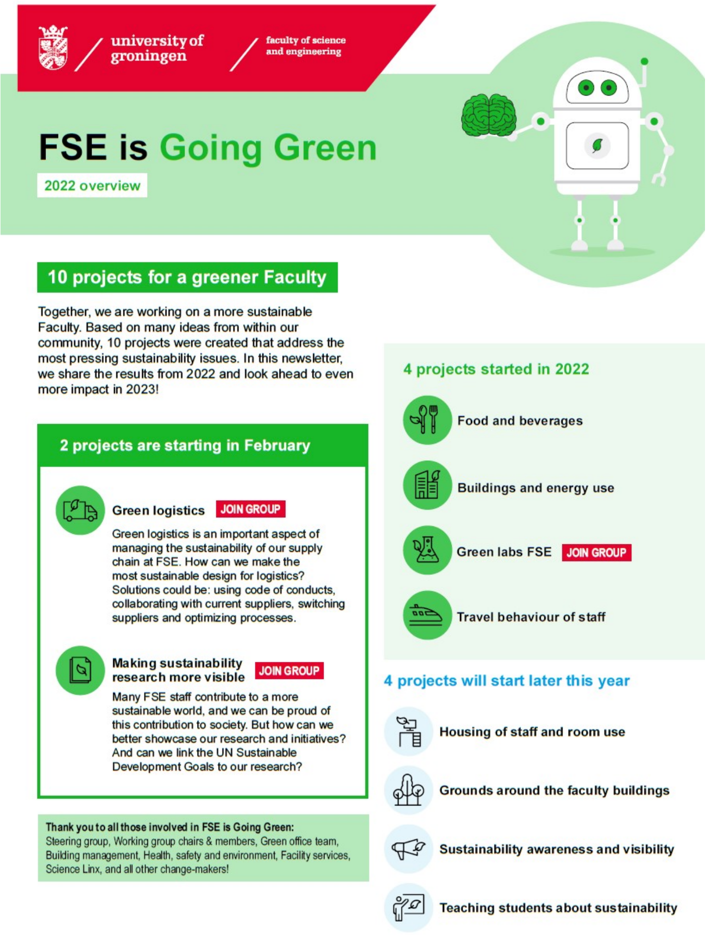

Food and beverages at canteens and events

Project 1

Aim

To increase the variety and quality of plant-based and vegetarian food at the canteens and for catering, and to reduce food waste

Impact in 2022

- Created more awareness for sustainable food through visibility of project and survey
- Conducted a survey amongst FSE staff and students: 578 respondents

How to help the change

- **By choosing plant-based and vegetarian options in canteens and for catering**, you directly support the change by creating a higher demand
- **Bringing your own coffee mug** reduces wasting disposable cups (these are difficult to recycle). You get a 10% discount at canteens and the Zernike food court when you buy coffee or tea using your own mug

Why

Food production causes **26%** of anthropogenic greenhouse gas emissions[1]

Switching to a plant-based diet can be beneficial for human health, planetary restoration and reducing greenhouse gas emissions

Plans for 2023

- Pilot to make **Bernouilliborg canteen more sustainable**, where changes are tested such as: improved labeling, reusable cutlery, more local/seasonal products and more plant-based and vegetarian alternatives
- Promoting and improving **plant-based catering for FSE events**, possibly making plant-based/vegetarian catering default (FSE signed a letter from the Green Office for UG-wide vegetarian catering)
- Pilot for a **food waste calendar for students**, where leftover food from canteens can be picked up and is not wasted

These plans need to be discussed and coordinated with Beijk, the company that takes care of the canteens and catering

1. Poore, J. & Nemecek, T. (2018). Reducing food's environmental impacts through producers and consumers. Science 360 (6392): 987-992.

Buildings and energy use

Project 2

Aim

To reduce our energy use by 30% in the coming 3 years

Impact in 2022*

- Lights were permanently put on night setting in Nijenborgh 4
- Thermostats were lowered throughout FSE buildings
- Comfort hours (when heating is on) reduced throughout FSE buildings

* part of these impacts were initiated by the Energy task force

How to help the change

- **Help insulate Nijenborgh 4:** research groups can organize team insulation sessions. Or insulate your own lab and/or office with the available packages (including window insulation film and radiator foil).

MORE INFORMATION

Why

The current electricity usage at FSE in 1 hour is equivalent to the usage = of 1 average Dutch family in a whole year

Plans for 2023

- **Insulation measures in Nijenborgh 4:** installing window insulation film (and other measures) to reduce heat loss. This will reduce gas consumption with a minimum of 91.000 m³ per year (=equivalent of >77 families)

  VIEW UKRANT VIDEO

- **Further reduce energy and gas consumption** through:
  - Targeted energy measurements
  - Replacing inefficient equipment
  - Installing local electric boilers
  - More lights on night setting & UG-wide plan to replace all lights with LED
- **Solar panels** will be installed on roofs of FSE buildings where possible (and where they are not yet present)

JOIN GROUP

Green Labs & LEAF

Project 3

Aim

To reduce (plastic) waste, water and energy use in our labs

Why

Labs use 10x more electricity and 4x more water than office spaces of the same size[1]

Labs also produce 5.5 million tonnes of plastic waste worldwide per year[2]

Impact in 2022

The pilot of the LEAF (Laboratory Efficiency Assessment Framework) project has resulted in:

- **4** labs received bronze LEAF award (3x Stratingh, 1x GELIFES)
- **2** labs received silver LEAF award (2x Stratingh)
- **11** labs are busy with accreditation and more have requested to start

41 ULT freezers were turned up to -70°C (from -80°C), saving the same energy as >13 households per year

Green lab guide book was created and distributed

Community meetings were held to discuss sustainability in labs

Plans for 2023

- Defrosting and cleaning ULT freezers and continuing efforts to turn up more freezers to -70°C
- All FSE labs participate in the LEAF project to make labs more sustainable
- Organizing regular community meetings & spreading green lab guides widely
- Pipette tip washing project: reducing the amount of plastic waste by switching to reusable pipette tips (starting with pilot project for students only)

How to help the change

- **Join a community meeting!** Here we discuss the LEAF project and how you can join, and share other sustainable guidance. The next meeting is on Feb 21st from 16:00. SIGN UP FOR THE MEETING
- **Join our working group.** We have had great success in 2022 thanks to some very enthusiastic change-makers, but we need your help to keep the momentum going. We also need LEAF coordinators who can help roll out this project. JOIN GROUP
- **Plan a day to defrost and clean your ULT freezers,** and use our green lab guidebook to make other sustainable changes in your lab. DOWNLOAD GREEN LAB GUIDE

1. Kaye, C. (2020). Media Briefings 2020. #3 Efficiency & Sustainability: Igniting Innovation. https://www.agilent.com/content/dam/about/newsroom/media-room/igniting-innovation/Efficiency-Sustainability-Igniting-Innovation-Media-Briefing.pdf
2. Urbina, M., Watts, A. & Reardon, E. (2015). Labs should cut plastic waste too. Nature, 528 (479). https://doi.org/10.1038/528479c

Travel behaviour of staff

Project 4

Aim

To reduce CO_2 emissions from air travel with 30% by 2026, in line with the sustainability roadmap of the University of Groningen

Why

16% of the total CO_2 footprint of the UG is caused by business trips, mostly by plane

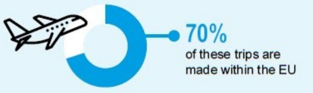

Impact in 2022

Conducted survey among FSE staff about (air) travel behaviour: 362 respondents filled in the survey - results are expected in February

Plans for 2023

- Analyzing, publishing and presenting the survey results in a report
- Giving advice to the Faculty on which measures would be best suited for FSE to reduce CO_2 emissions from air travel

How to help the change

- Traveling abroad for work? Take the **train as much as possible.** As of 2023, it is UG policy to travel by train for journeys up to 9 hours. READ MORE ABOUT THE NEW POLICY
- **Consider attending meetings remotely.** Especially for short and targeted meetings, online should become the default. The faculty has good video conference facilities. FIND ROOMS WITH ONLINE MEETING FACILITIES

2.6.1 Feringa Building

The old laboratory complex (Nijenborgh 4, constructed in 1969) for research and education in chemistry, physics and engineering was technically outdated and did not align with modern environmental and energy standards. After thorough investigation (renovations *vs.* new building), the University of Groningen decided to build the Feringa Building to replace Nijenborgh 4. The building will include the education and research in chemistry, physics, astronomy, and engineering, covering the following institutes:

- Stratingh Institute for Chemistry
- Engineering and Technology Institute Groningen (ENTEG)
- Zernike Institute for Advanced Materials (ZIAM)
- Groningen Biomolecular Sciences and Biotechnology Institute (GBB)
- Van Swinderen Institute for Particle Physics and Gravity
- Kapteyn Astronomical Institute
- SRON Netherlands Institute for Space Research

The Feringa opened on March 1st, 2024, and includes:

- 64 000 m^2 gross floor area
- Size: 260 m long, 63 m wide
- Five stories high, with an extra floor for installations
- Space for 1400 students and 850 staff members
- A large lecture hall with 420 seats, can be divided in 180 and 240 seats
- Restaurant with 250 seats
- 3 km of lab tables and 450 fume hoods
- Clean rooms, low vibration laboratories and 30 laser labs
- Air volume flow of 900 000 m^3/h

Sustainability aspects:

All the laboratories are built on the north side of each wing, keeping the impact of sunlight to a minimum. The chemical, biochemical and physics laboratories are flexible and interchangeable as each one can be connected separately to the ventilation, power, and gas supply networks.

- Optimal insulation
- Heat reflective coating (HR) glass, better insulation than standard double glazing glass
- 900 m^2 solar panels (± 120 000 W_P (watt-peak) of nominal power)
- LED lights in addition to natural daylight
- Gasless heating
- Geothermal heating and cooling system (‘WKO’) with heat pumps
- Energy saving auto close fume hoods
- 4 courtyards for biodiversity

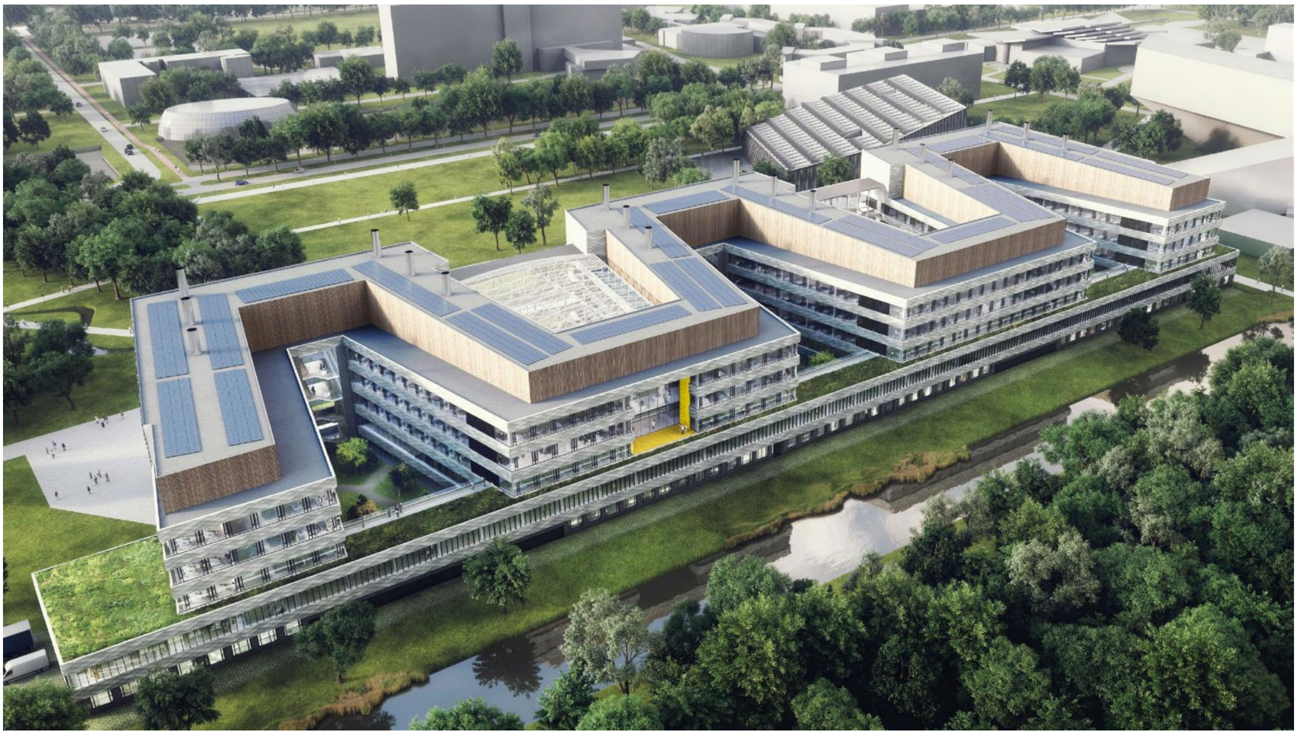

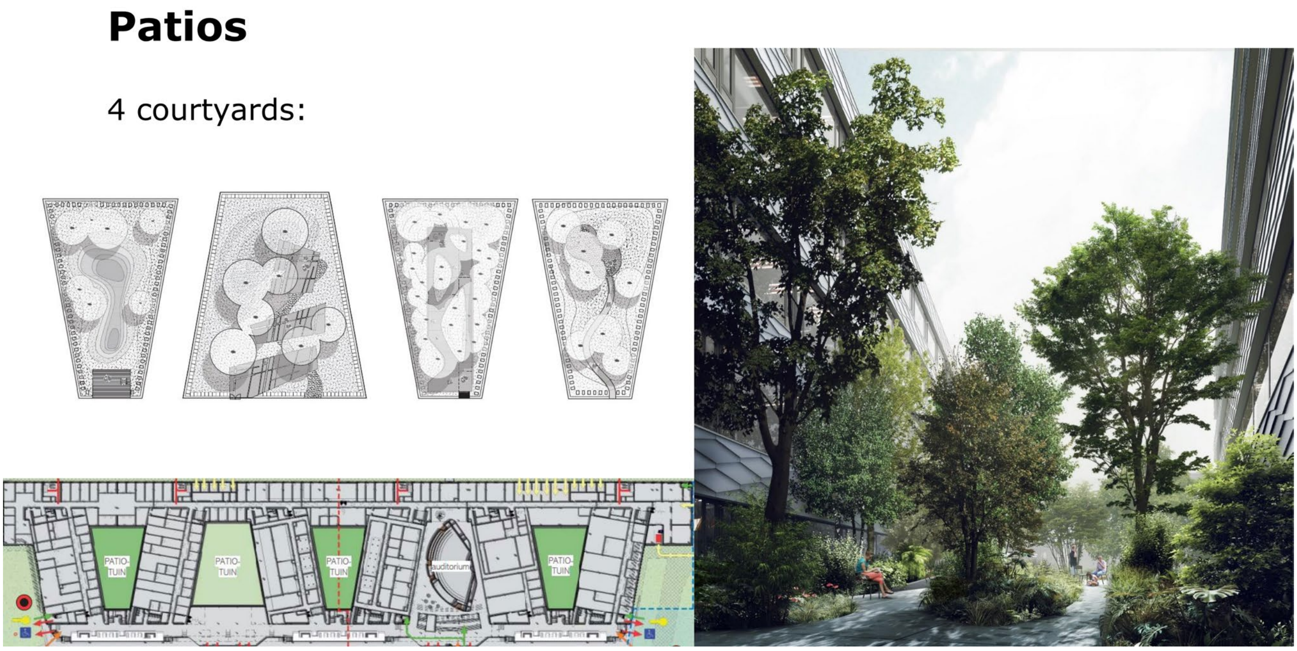

Figure S10: Design of the Feringa Building (top) including the 4 courtyards to enhance biodiversity (bottom) and solar panels (900 m^2), HR glass, heat pumps, automated closing fume hoods, LED and natural light.

2.6.2 Travel behavior of staff at the Faculty of Science and Engineering

The following sections are reproduced or adapted and were calculated through F.F.S. van der Tak and are published with permission of the Faculty of Science and Engineering, University of Groningen.

'FSE is going green: travel behavior of the staff'

The results of the survey held by the 'FSE is going green' working group 'Travel behavior of the staff', are depicted below.

The University of Groningen aims to reduce CO_2 emissions from air travel by 30% by 2026 (compared to 2019). To achieve this goal, UG-wide measures regarding work-related travel behavior will be implemented. In this light, the recommendations in the report are valuable and the Faculty Board has therefore submitted them to the Green Office, which is taking the lead in developing university-wide measures.

In its survey, the 'Travel behavior' working group proposes four measures, two of which are stimulating measures (monitoring CO_2 emissions and a climate contribution for air travel) and two are more restrictive measures (a CO_2 budget and a mandatory reduction). The Board does not rule out the possibility that more restrictive measures will be necessary in the long term, in order to achieve the intended reduction in CO_2 emissions.

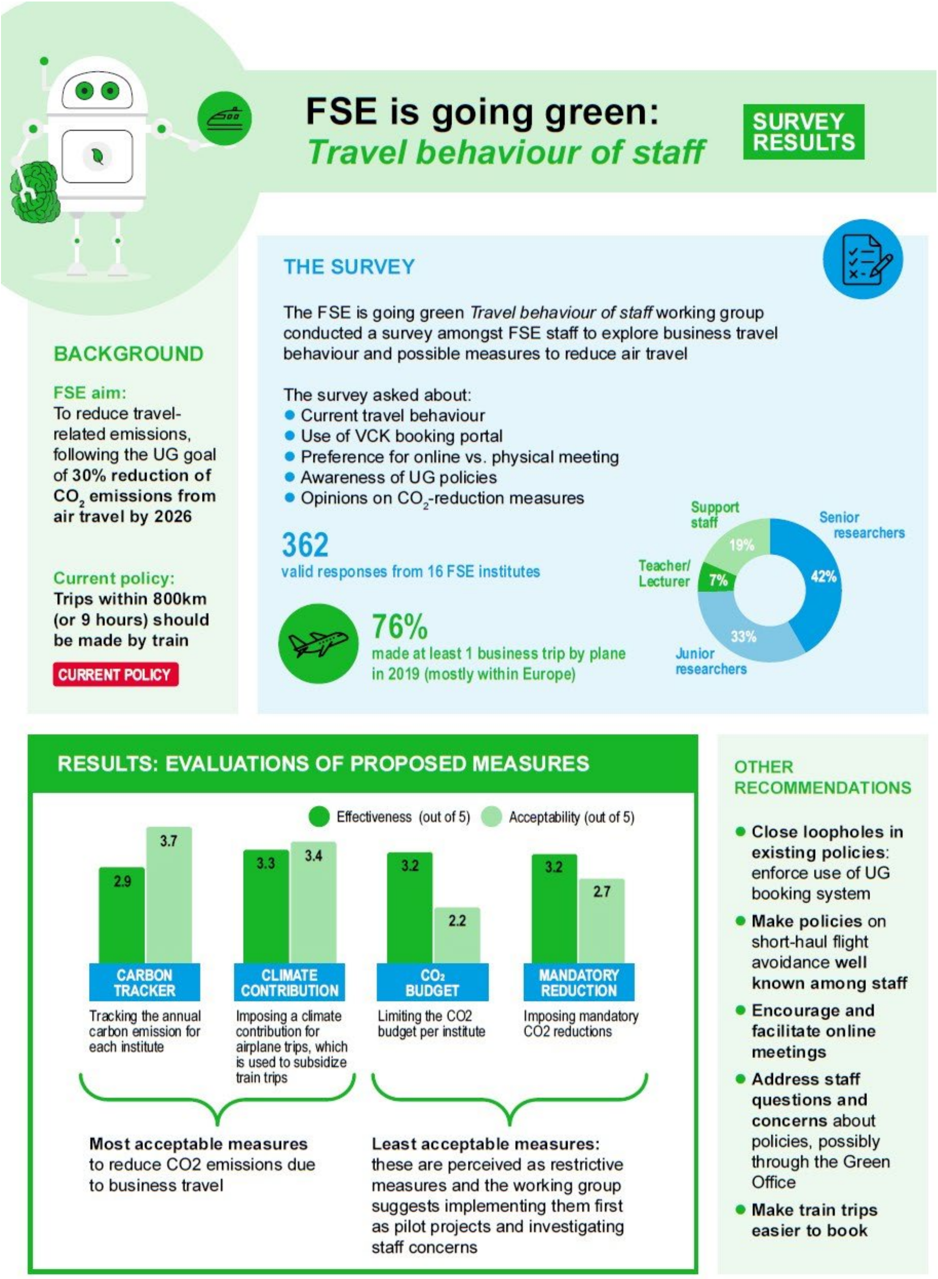

2.6.1.1 Summary

In a survey among FSE staff, two policies to reduce CO_2 emission due to business travel were found to be the most acceptable: tracking the annual carbon emission for each institute, and imposing a climate contribution for airplane trips, which is used to subsidize train trips. We recommend implementing these policies after working out their practical details. In contrast, imposing mandatory CO_2 reductions and limiting the CO_2 budget per institute are perceived as restrictive measures, which we suggest implementing only as pilot projects, after investigating staff concerns. In addition to introducing new policies, we recommend closing loopholes in existing policies, making current and planned policies on short-haul flight avoidance well known among staff, to encourage and facilitate online meetings, to address staff questions and concerns about policies, and to make the booking of international train trips easier.

2.6.1.2 Context and scope

In Spring 2022, the Faculty board started the FSE is Going Green program,[4] which aims to make FSE operations more environmentally sustainable by limiting resource use, waste production, and greenhouse gas emission. The working group Travel behavior of the staff has looked specifically into ways to reduce greenhouse gas emissions due to business travel, in particular by airplane. The target reduction is 30% by 2026, in line with the UG sustainability goals.[5] The working group did not consider commuting and trips within the Netherlands, as these were found to be minor sources of CO2 in a 2020 Arcadis study of the nine NWO institutes, and a 2021 study of astronomy research in the Netherlands.[6] The UG policy on parking permits addresses pollution by commuting.

2.6.1.3 Approach

Since 2019, policy at UG has been that trips within 500 km (or 6 hours train travel time) from Groningen cannot be taken by airplane; as of 2023, this radius is extended[1] to 800 km (or 9 hours).[7] In November 2022, we conducted a survey among all 2889 staff employed by FSE institutes, asking about travel behavior, use of travel agency portal. online *vs.* physical meetings, awareness about UG mobility policies, and their opinions on additional measures for reducing CO_2 emissions connected with UG travels. An open box was added at the end of the survey to collect staff members' comments and suggestions about the topic. The target group includes junior (45%) and senior (11%) scientific staff, teaching staff (5%), support staff (23%), and researchers on temporary contracts such as PhD students and postdocs (16%). Undergraduate students were not part of the survey, since FSE has no legal means to influence their travel behavior. See Appendix A for the full text of the questionnaire. We received 362 valid responses (13%) from 16 institutes and offices across FSE. The full results of the survey are in Appendix B, and the main findings are summarized below.

---

[1] *Implemented after the survey was conducted*

2.6.1.4 Results on preferred policies

We presented respondents with four additional measures:

1. Launch a carbon tracker showing how much CO_2 each institute emits by traveling.

2. Introduce a small (*e.g.* 5%) climate contribution for airplane trips, which is used to sponsor train trips through first class tickets, frequent traveler cards, etc.

3. Impose mandatory emission reduction targets for flights on all institutes (scaled with institute size).

4. Give each department a limited CO_2 budget for traveling (scaled with institute size).

We asked participants to indicate how effective (*i.e.*, in reducing the CO_2 emission caused by staff members' flying behavior) and acceptable (i.e., with minimal impact on their work) they found each measure. The table and figure below report these scores on a 5-point scale.

	EFFECTIVENESS		ACCEPTABILITY	
	Mean	Median	Mean	Median
Carbon tracker	3.0	3	3.7	4
Climate contribution	3.3	3	3.4	4
CO_2 budget	3.2	3	2.2	2
Mandatory reduction	3.2	3	2.7	3

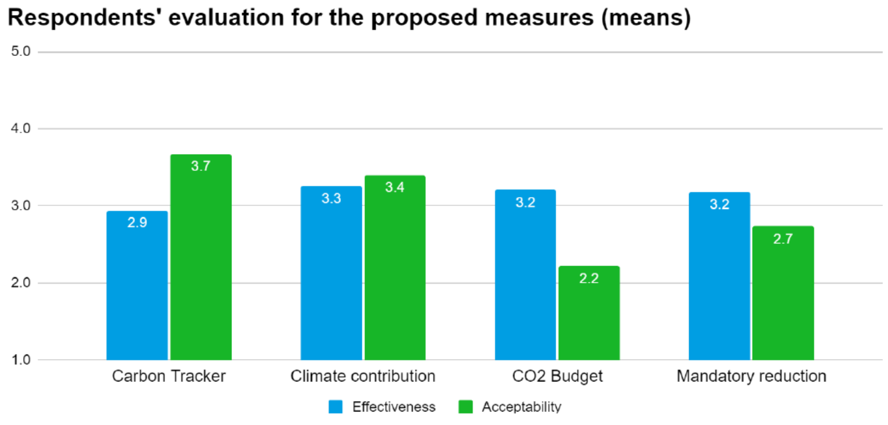

[2] Responses with at least the first question related to UG mobility completed are considered valid.

Figure S11: Respondents' evaluation of proposed travel policies and measures.

2.6.1.5 Other results

Travel behavior: Among the staff members who indicated traveling in 2019, the majority (65%) took 1 to 5 flights. Senior researchers clearly travel the most. The most chosen destination was Europe (131 flights out of 196). Some institutes fly much more than others.

Portal use: Booking trips via VCK (UG travel agent) is done always by 24%, usually by 34%, sometimes by 21%, and never by 21% of respondents. The latter number is surprisingly high, and is especially high (41%) among support staff. This is unexpected since FSSC is supposed to refuse reimbursement of trips which are booked outside the portal.

Policy awareness: The large majority of respondents were aware of the UG mobility policy implemented in 2019 (78%) and supported it to some extent (84%). When asked about the 2023 update of the policy, about half of them were informed about it (47%) and indicated supporting it to some extent (63%). Few people indicated being strongly against these two policies (3% and 9% for the old and new updated policy respectively).

Meeting preference: Attending meetings online works in most or all cases for 18%, in ~half the cases for 43%, and in some or no cases for 40% of respondents. The type of meeting is likely to play a role, as small targeted (business / committee) meetings may be more suitable for an online setting than larger open-ended meetings such as brainstorms and conferences.

Further suggestions: Finally, the respondents came with questions and suggestions of their own. The most common remarks were: trains are often delayed or crowded; policies need to be fair toward staff with special needs; some airplane travel will always be needed. A common question is how exactly travel times and distances are calculated.

2.6.1.6 Discussion

Policy acceptance: For all four proposed measures, the differences in acceptability between institutes are small, suggesting that the variation in acceptability between measures is real. Push measures (*i.e.*, taxes or restrictions) are often perceived as less acceptable than pull measures (e.g., discounts), but they usually are more effective. Research suggests using combinations of both measures. In addition, taxes and monetary contributions are perceived to be more effective and acceptable when revenues are allocated within the same domain (e.g., climate contribution for flying going to subsidize train travel).[10,11,12] We suggest implementing first the two policies that were considered to be more acceptable (*i.e.*, carbon tracker and climate contribution). We then suggest investigating in more depth the less acceptable policies (*e.g.*, CO_2 budget and mandatory reduction), and targeting the concerns that emerge (e.g., previous research suggests that perceived unfairness might play a substantial role).[12] In addition, implementing the more restrictive policies as pilots might be a solution, as the acceptability of a policy tends to increase after its implementation.[13]

Perceived effectiveness: The four proposed measures are seen by FSE staff as about equally effective (see Table above) to reduce CO_2 emissions. This is surprising as differing trends between effectiveness and acceptability may be expected. It may be that staff members have difficulty separating effectiveness and acceptability. The perceived effectiveness of the four measures varies considerably between institutes, which may indicate differences in travel culture. Alternatively, this variation may again indicate respondents' uncertainty as to what measures would be effective. Literature indeed suggests that people are not accurate when estimating the effectiveness of such policies.[10] For example, pricing is known to affect air travel substantially.[8]

2.6.1.7 Caveats

Demographics: The distribution of respondents over staff types mostly follows the intrinsic distribution, except that senior researchers are overrepresented and junior researchers (PhDs and Postdocs) underrepresented. The response fraction varies considerably between institutes, possibly because of the way that the questionnaire was distributed (*via* the institute directors). Future follow-up surveys may use stratified sampling to be representative of all career stages and scientific disciplines across FSE.

Sample size: The total number of respondents is high enough to draw conclusions for FSE as a whole.[14] Only a few institutes have enough responses for meaningful subsamples,[15] so we interpret our survey results only in aggregate. Due to the way that the survey was introduced, the sample is of a convenience nature, rather than strictly controlled, but we nevertheless consider it useful for our purpose.

Potential bias: The responses to our survey may be biased because climate-minded staff are more likely to respond. Such bias is especially likely for the question about post pandemic travel behavior, where 65% claim to have flown less in 2022 than in 2019, and 85% of these claim that sustainability is a reason for this change. Adding a lottery (the chance of winning a small prize) may help against this bias in future surveys.

2.6.1.8 Recommendations and further steps

Two policies are likely to meet with broad acceptance among FSE staff: tracking the annual carbon emission for each institute, and imposing a small climate contribution for airplane trips, which is used to sponsor train trips. More restrictive policies, such as limiting the CO_2 budget of each institute and imposing mandatory reductions in CO_2 emission, may meet with some resistance, and should only be implemented as pilot projects or after studying the concerns of FSE staff.

Before the new policies can be implemented, they need to be worked out further. In particular, the carbon tracker could use the open-source tool developed in,[9] but it must be clarified where the tool gets its input data. For the contribution measure, the questionnaire suggested a 5% surcharge, but the final number may be different. The contribution could be charged through the UG travel portal, but it needs to be worked out how the funds would be redistributed.

Besides considering new policies, we recommend the following steps:

1. Close loopholes in existing policies. In exceptional cases, requests for bookings using a travel agency other than the internal UG agency may be granted, but ~20% of staff never using the portal is clearly too much. The high acceptance rate of the old and new UG travel policies suggests that this behavior is due to a lack of awareness, rather than a sign of resistance. We do, however, note that booking train trips via VCK, especially across multiple countries, is not always straightforward.

2. Make policies well known. A significant fraction (~55%) of FSE staff was unaware of the upcoming travel policy one month before its implementation, and ~20% unaware even of the one introduced in 2019. Through newsletters and messages to new staff, travel regulations should be announced more prominently. The UG travel portal can help reminding too.

3. Encourage and facilitate online meetings where possible. Short and/or targeted events such as thesis defenses, colloquia/seminars, and business/committee meetings should become online/hybrid as much as possible. Part of this policy may be establishing an online etiquette (turning cameras on, identifying with full name, etc.). Rooms with video equipment should be available to all staff.

4. Address questions and concerns about policies. The responses suggest that most FSE staff are willing to adopt a pro-train policy, but that they have questions about its practical implementation. For broad acceptance of policies it is important that these concerns are properly addressed.

5. Make train trips easy to book from the UG portal. Staff would be much more willing to travel by train if the booking process were smooth and convenient. This should be a requirement for the 2023 version of the UG travel portal.

2.6.1.9 Appendix: Full results of the survey

Demographics

The majority of respondents are from Bernoulli (17%) and ENTEG (14%). Respondents indicating 'others' are from FTD, Science Linx, SSE/ENTEG; 9 of them did not indicate their institute (Fig 1). The majority of respondents are Senior researchers and Junior researchers, i.e., PhDs and Post-docs (Fig 2).

Figure 1.

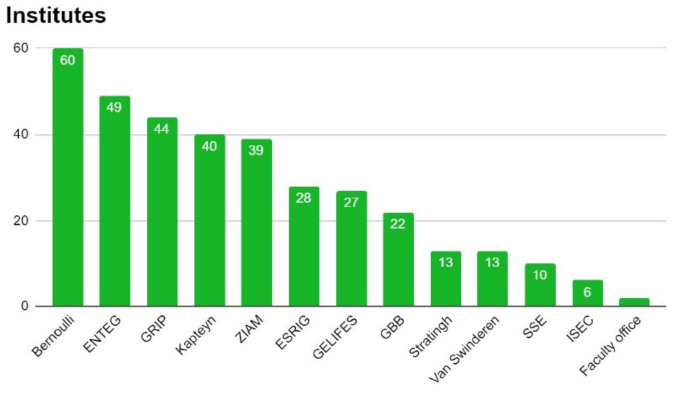

**N=362*

Figure 2.

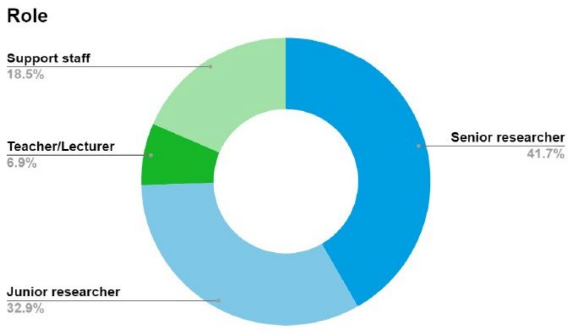

**N=362*

Proposed measures

Participants were asked to indicate on a 5-point scale [6] how effective (i.e., in reducing the CO_2 emission caused by staff members' flying behavior) and acceptable (i.e., with minimal impact on their work) they found each measure. Respondents evaluate the effectiveness of each policy to a similar extent. The carbon tracker was evaluated as most acceptable, followed by the climate contribution and the mandatory reduction. The least acceptable measure was the CO_2 budget.

Figure 3.

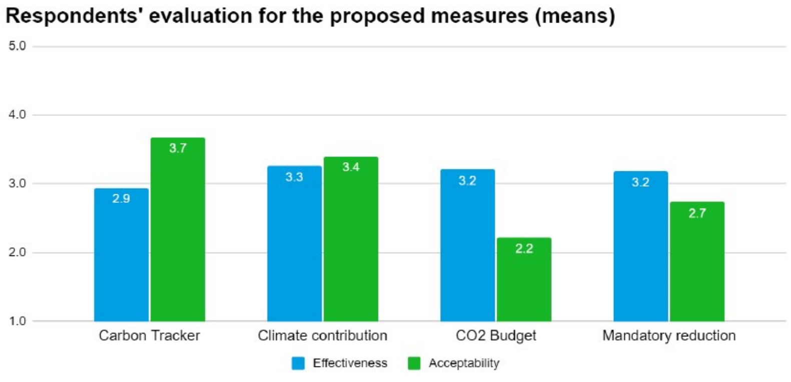

**N=362*

Business trips by plane in 2019

Ca. half of respondents indicated traveling in 2019 for business trips. The majority (65%) took between 1 to 5 flights in 2019 (Fig 4). The most chosen destination was Europe: 131 flights (Fig 5).

Figure 4.

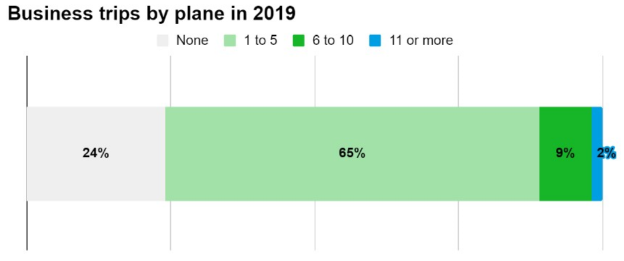

*N=202

Figure 5.

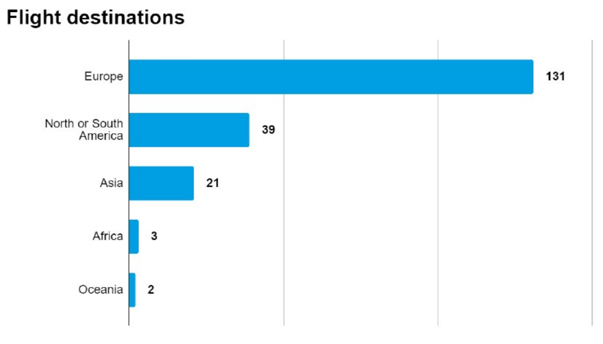

*N=133

VCK use

Only 23% of respondents use VCK to book travels outside the Netherlands. 22% reported never using it (Fig. 6) and this number doubles when considering support staff (42%). The use of VCK is perceived as quite positive, but not particularly fast and quite difficult (Fig 7).

Figure 6.

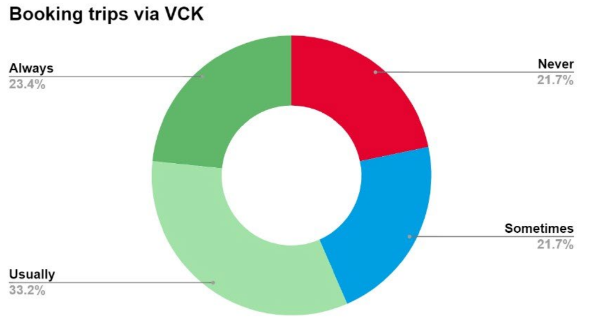

*N=184

Figure 7.

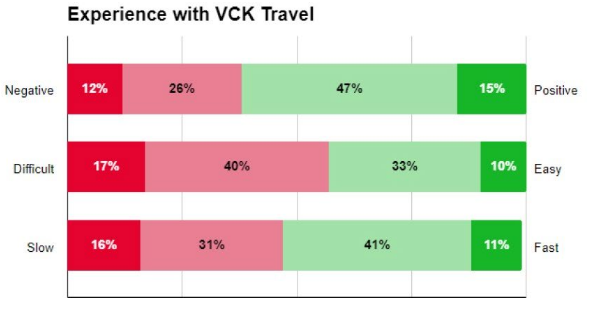

*N=184

Support for mobility policies

The large majority of respondents were aware of the UG mobility policy implemented in 2019 (78%) and the new mobility policy becoming effective in January 2023 (63%).

Figure 8.

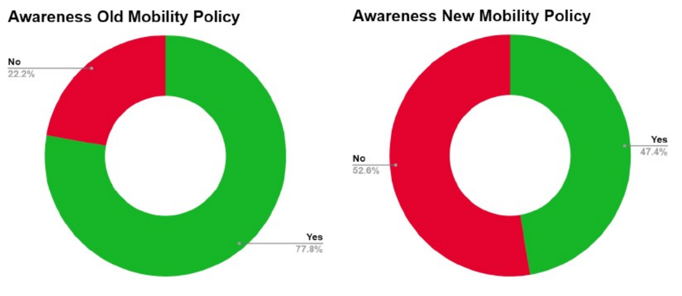

*N=342

Participants were asked on a 7-point scale to indicate the extent to which they were in favor or against the two policies. The large majority of respondents were aware of the UG mobility policy implemented in 2019 (78%) and supported it to some extent (84%). When asked about the new updated version of the policy (900 km/8 h), about half of them were informed about it (47%) and indicated supporting it to some extent (63%).

Figure 9.

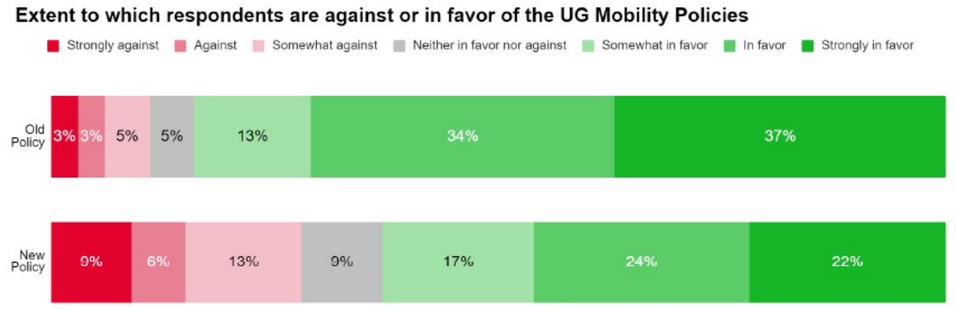

Meeting preferences

When asked about meeting preferences, 40% of respondents stated that online attendance is an acceptable alternative to in-person attendance about half of the time.

Figure 10.

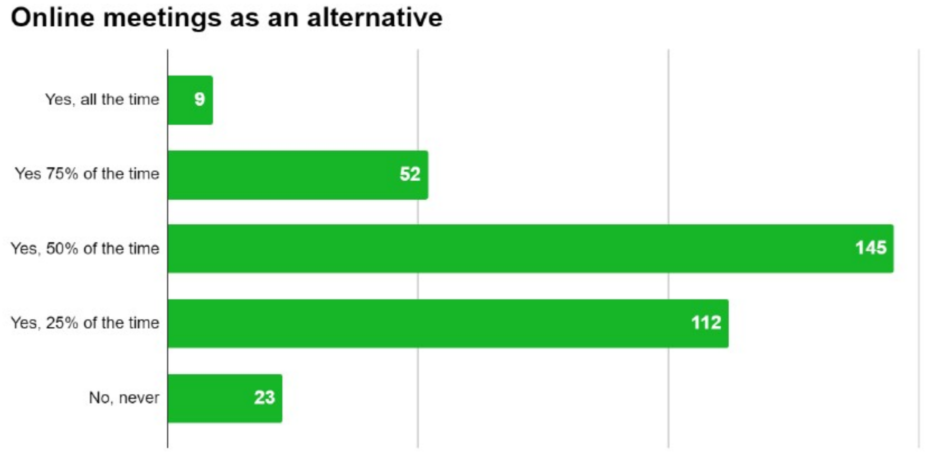

*N=341

2.6.3 Food and canteens

In light of the '*FSE is going green*' program the '*Food, beverages and canteens working group*' initiated a pilot for a more sustainable canteen. In collaboration with Contract Management (US), Beijk Catering and the UG Green Office a plan was made and survey conducted to improve sustainability of canteens at FSE. *We hereby acknowledge all efforts and support of I. Maltagliati to enhance progress towards sustainable catering.*

Context

There has been a survey within the FSE (staff and students, 578 valid respondents), launched by the food working group and the Green Office. This survey showed that sustainability and food variety were points to improve. It also showed that policies towards more plant-based food are supported by most respondents. *See appendix A for the main results.*

<u>*Opinions on canteens:*</u>
food variety and sustainability scored low: respectively 77% and 64% of the respondents think these aspects should be improved.

<u>Willingness to pay more for:</u>
warm meals (on average 21% more), healthy options (17% more), sustainable options (16% more).

<u>Policy acceptance:</u>
64% of responders think that plant-based food should be a default during events.

<u>Policy acceptance:</u>
66% of responders agree that there should be one day with only plant-based food per week in the university canteens.

<u>Policy acceptance:</u>
80% of respondents agree that 50% of food served in the university canteens should be plant-based.

75% of responders were not aware that there is a 10% discount on warm beverages for bringing your own mug.

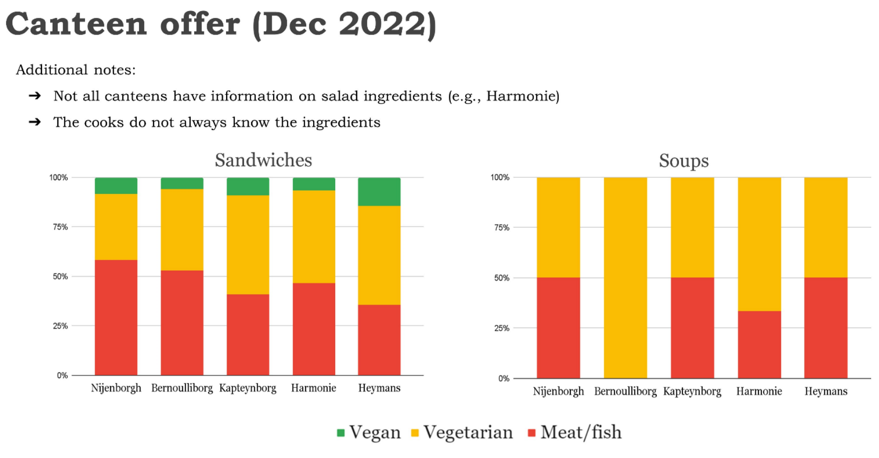

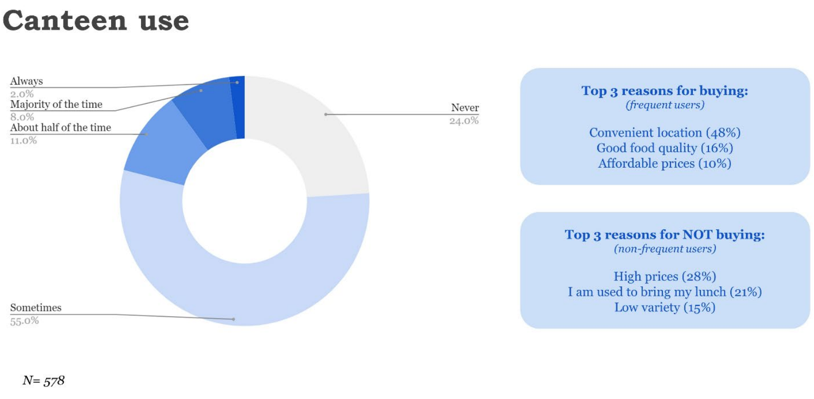

Figure S12: Survey responses on canteen offer and canteen use.

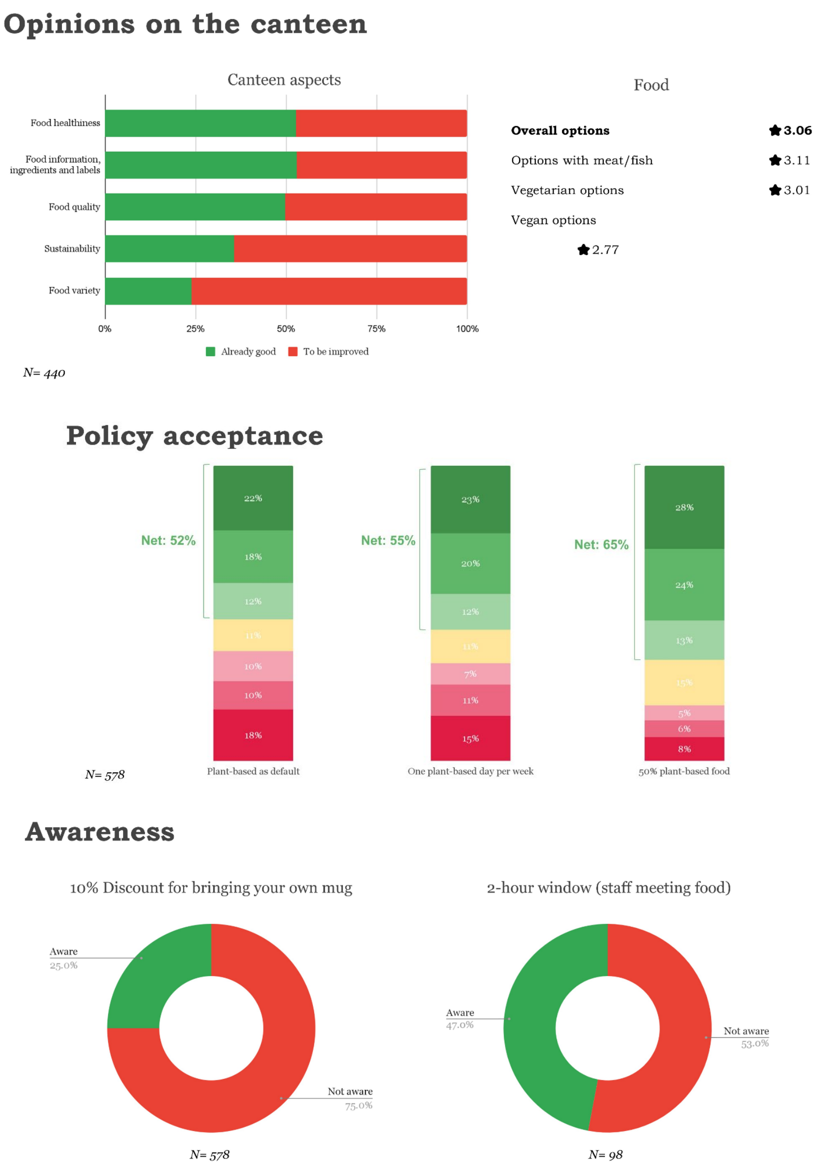

Figure S13: Survey results on opinions on the canteens, policy acceptance and awareness.

Bernoulli Bistro

With the results of the survey the working group was able to establish sustainable new canteen at the Zernike Campus of UG, which covers the whole FSE: Ther Bernoulli Bistro launched in 2023 offers 100% vegetarian fresh food which is bought locally. The canteen operates with reusable tableware explicitly, and packaging is non-plastic thus recyclable.

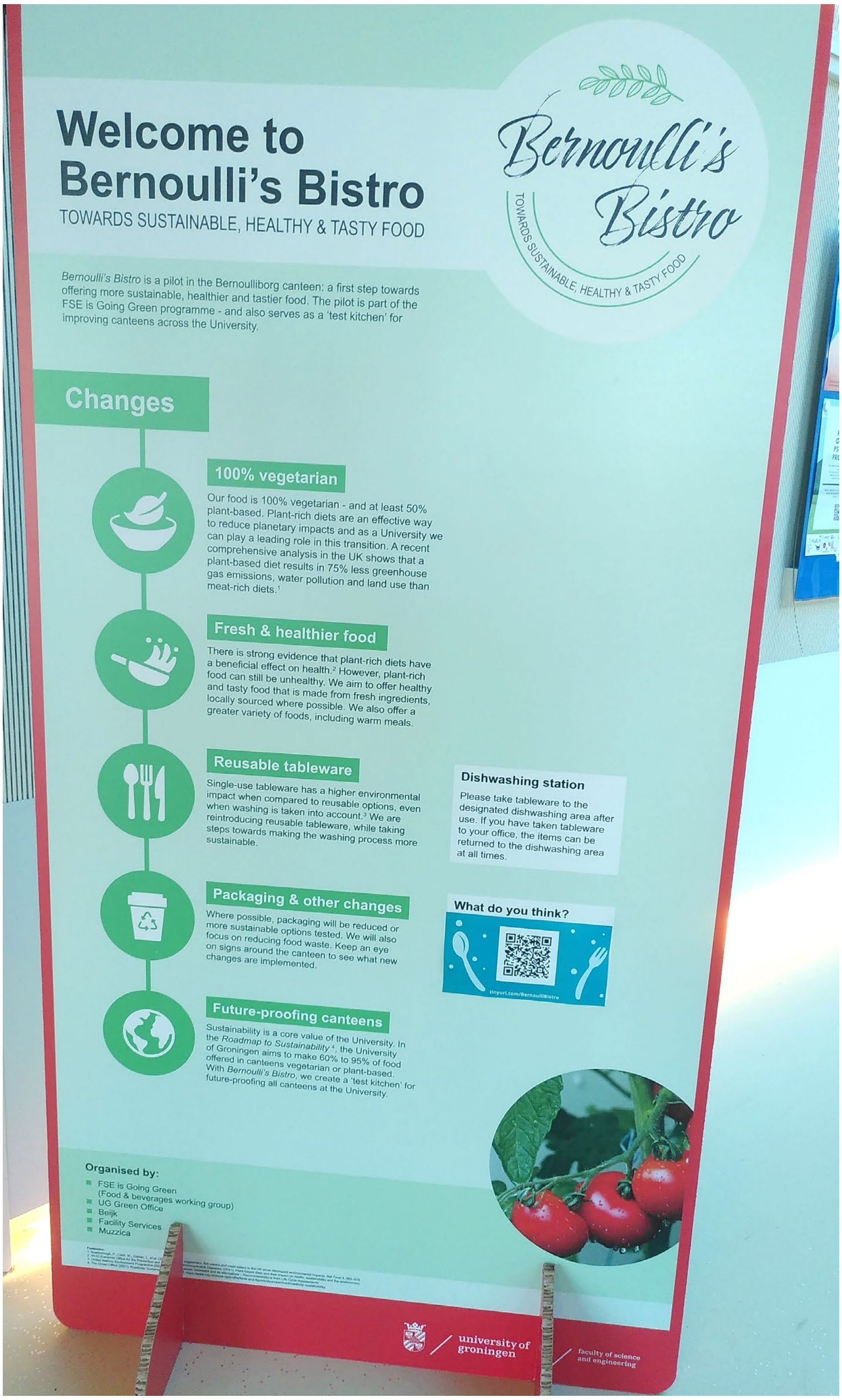

Figure S14: Bernoulli Bistro for sustainable food and canteens.

2.6.4 Biodiversity

The project Grounds around the Faculty Building (henceforth abbreviated as GAF) is one of the 10 working groups of FSE is Going Green, a program focusing on improving sustainability at the UG. An important partner for GAF is the Green Office, which coordinates projects university-wide and the Terrain Management department, which is responsible for ground maintenance. The working group focuses on the Grounds around the Faculty Buildings at Zernike. There is also a publication covering the sustainable and nature-driven design of the Zernike Campus of the University of Groningen.[21]

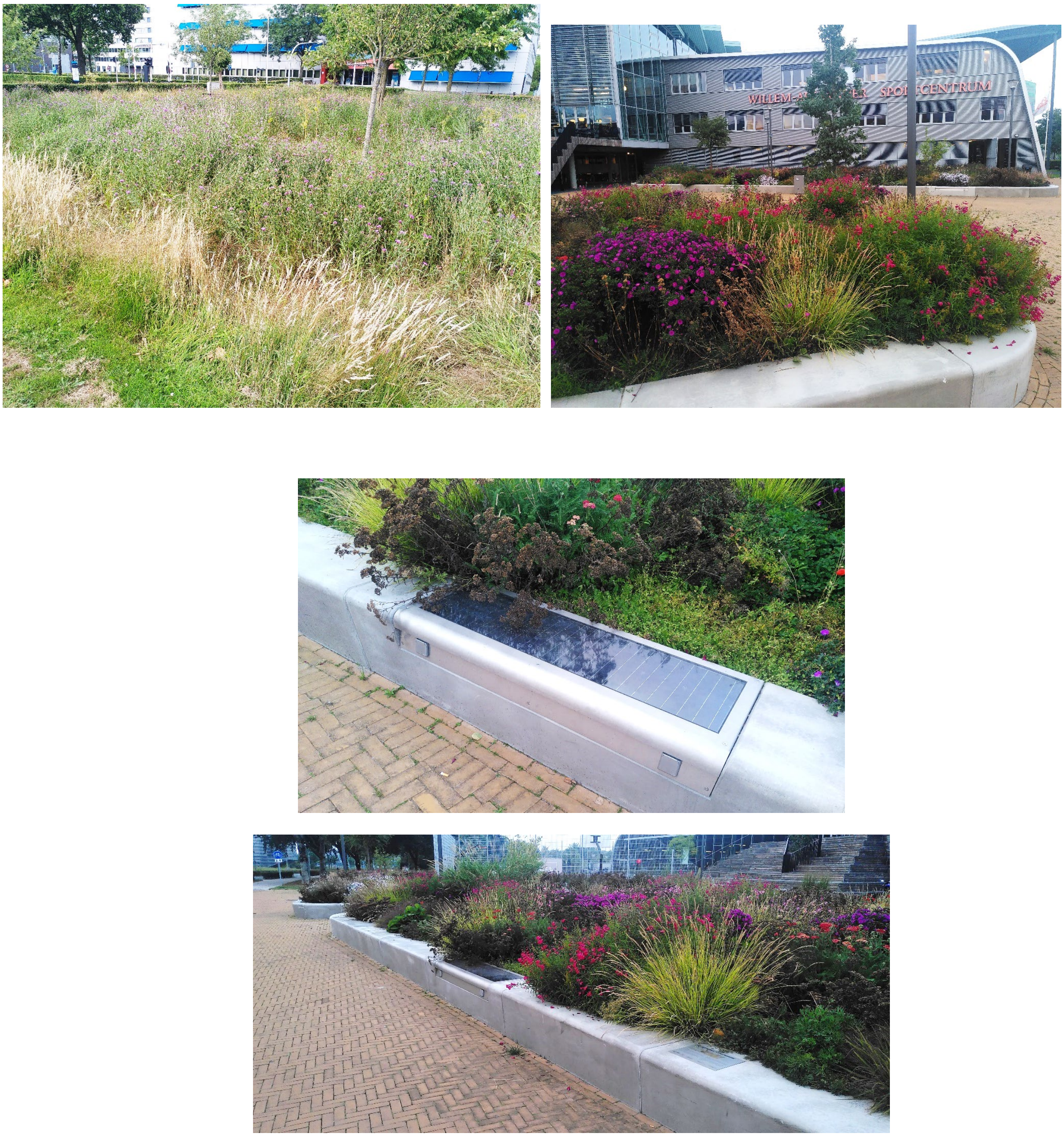

Figure S15: Free flower and grass areas for insects and bees (top left). Smart watering areas, which are solar fueled to pump water to the dry soil to keep the plants alive depending on temperature and humidity (top right and bottom).

2.7 Green Labs RUG

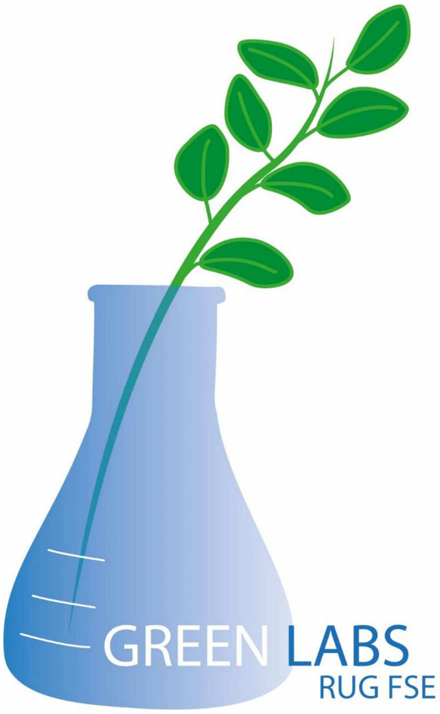

Figure S16: Logo of the Green Labs RUG team. *Credit: M. M. Weber.*

2.7.1 Recommended structure of a green lab grassroot team

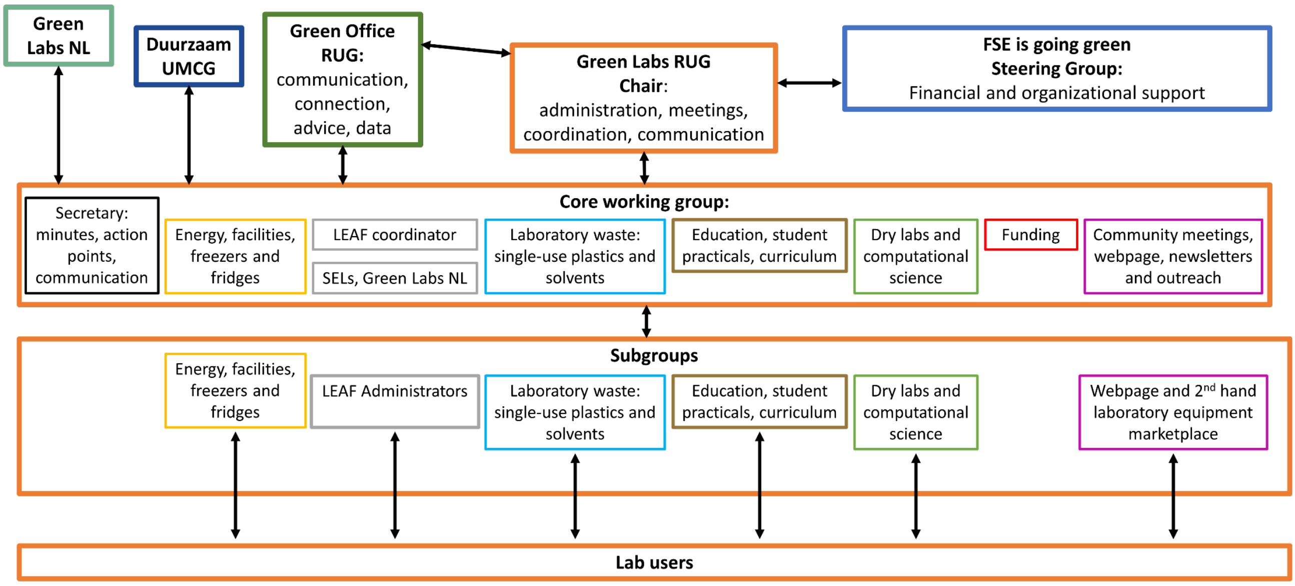

Figure S17: Recommended organizational structure of a Green Lab grassroot group covering all relevant aspects.

2.7.2 Laboratory efficiency assessment framework (LEAF) at FSE

The Green Lab initiative and the Laboratory Efficiency Accreditation Framework (LEAF) made quite some progress over the past two years. As of October 2023, there are 17 labs accredited with LEAF silver and 29 with LEAF bronze (46 labs in total). Student educational labs in Nijenborgh 4 are now fully accredited and assuring that future generations of students are directly familiarized with the concept of sustainable laboratory practices. There are several institutes represented in the LEAF accreditation such as GELIFES, Stratingh, ENTEG, GRIP, Bernoulli, GBB. Besides that, dry labs such as Computational Science and Mathematics are now able to join the LEAF framework as criteria and action points were expanded towards digital research. SRON and the Kapteyn institute are the most recent institutes to join the sustainable laboratory efforts.
All alternative products, networks, actions, and measures are put in a comprehensive guidebook written by the Green Lab team. Also, an outreach movie about sustainable laboratories has been made featuring Ben L. Feringa being fully supportive of the program in the future. Several future action points are being worked on at the moment such as improving and recycling of laboratory plastic consumables and a solvent recycling facility.

Extrapolating the reduced carbon impact of the accredited laboratories to all 46 accredited laboratories equals annual savings of **398 763 euros** as well as **477 107 kg of CO_2**. These numbers are especially impressive when considering that many old laboratories still contain fume hoods with fixed air floor (thus are not reducing its flow when closed) and are chemical science related. Thus, the there is no ultra-low temperature freezer existent in those spaces, both of which would increase the impact of these laboratory measures even further.

Table S32: Annual savings and emission numbers before and after accreditation of a 2-year-old laboratory in Linnaeusborg (Feringa CBBC, 5172.932), pricing is based on the electricity prices of December 2023.

Feringa CBBC	Before		After		Difference	
**Category**	**Annual Costs (€)**	**CO_2 Emissions (kg)**	**Annual Costs (€)**	**CO_2 Emissions (kg)**	**Annual Costs (€)**	**CO_2 Emissions (kg)**
**Waste**	3722.29	1612.94	3722.29	1612.94	0	0
**Fume Cupboards**	20451.19	28390	20009.37	27680	441.82	710
**ULT Freezers**	n/a	n/a	n/a	n/a	n/a	n/a
**Refrigerators**	408.36	260	398.5	260	9.86	0
**Water**	561.6	n/a	374.4	n/a	187.2	n/a
**Biosafety Cabinets**	n/a	n/a	n/a	n/a	n/a	n/a
**– 20 °C Freezers**	452.57	271	434.5	260	18.07	11
**IT**	1123.2	870	699.75	540	423.45	330
**Miscellaneous Equipment**	865.49	670	761.44	590	104.05	80
**Sum**	**27 585 €**	**32 074 kg**	**26 400 €**	**30 943 kg**	**1184 €**	**1131 kg**

Table S33: Annual savings and emission numbers before and after accreditation of a 2-year-old laboratory in Linnaeusborg (Lerch CBBC, 5172.944), pricing is based on the electricity prices of December 2023.

Lerch group	Before		After		Difference	
Category	Annual Costs (€)	CO_2 Emissions (kg)	Annual Costs (€)	CO_2 Emissions (kg)	Annual Costs (€)	CO_2 Emissions (kg)
Waste	3722.29	1612.94	3722.29	1612.94	0	0
Fume Cupboards	16966.88	23940	16525.06	23220	441.82	720
ULT Freezers	n/a	n/a	n/a	n/a	n/a	n/a
Refrigerators	209.1	140	199.25	130	9.85	10
Water	678.6	n/a	161.46	n/a	517.14	n/a
Biosafety Cabinets	n/a	n/a	n/a	n/a	n/a	n/a
– 20 °C Freezers	452.57	271	434.5	260	18.07	11
IT	795.6	620	410.59	320	385.01	300
Miscellaneous Equipment	1834.25	1430	490.43	380	1343.8	1050
Sum	**24 659 €**	**28 014 kg**	**21 944 €**	**25 923 kg**	**2716 €**	**2091 kg**

Table S34: Annual savings and emission numbers before and after accreditation of 15 old laboratories in Nijenborgh (educational laboratories), pricing is based on the electricity prices of December 2023.

Educational labs	Before		After		Difference		Difference of 15 laboratories	
Category	Annual Costs (€)	CO_2 Emissions (kg)	Annual Costs (€)	CO_2 Emissions (kg)	Annual Costs (€)	CO_2 Emissions (kg)	Annual Costs (€)	CO_2 Emissions (kg)
Waste	347.64	148.55	347.64	148.55	0	0	0	0
Fume Cupboards	37506.52	53360	30959.91	43540	6546.61	9820	98199.15	147300
ULT Freezers	n/a	n/a	n/a	n/a	n/a	n/a	n/a	n/a
Refrigerators	198.18	140	198.18	140	0	0	0	0
Water	840.32	n/a	37.44	n/a	802.88	n/a	12043.2	n/a
Biosafety Cabinets	n/a	n/a	n/a	n/a	n/a	n/a	n/a	n/a
– 20 °C Freezers	376.06	280	346.5	260	29.56	20	443.4	300
IT	419.33	330	58.24	50	361.09	280	5416.35	4200
Miscellaneous Equipment	3357.99	2610	1533.54	1190	1824.45	1420	27366.75	21300
Sum	**43 046 €**	**56 869 kg**	**33 481 €**	**45 329 kg**	**9565 €**	**11 540 kg**	**143 469 €**	**173 100 kg**

2.7.2.1 Guide on how to audit laboratories

- Before going to the respective lab, open the answers in the LEAF software beforehand and go through them. Write down notes/questions about things that are a little unclear and ask during the auditing process.
- Things to look out for during auditing (bronze):
  - waste funnels with lids used or round bottom flasks inside funnels of waste canisters
  - fume hoods closed when not in use

  - devices, equipment and light turned off when not in use
  - devices and equipment have all stickers to indicate how to turn off (also stickers on fume hoods and rotavaps)
  - multiplugs in use to make turning devices off convenient
  - rotary evaporator has evaporation balls placed inside the water bath

  - freezer temperatures are increased to from -80 to -70 and from -25 to -15
  - fridge temperatures are increased from 4 degrees to 7 degrees

  - introduction procedure on lab guidebook reading in place, where safety manager (*i.e.* Armico) also asks questions about it and safety, Lab guidebook can be provided by us: https://doi.org/10.26434/chemrxiv-2023-g3lmq-v2 or https://doi.org/10.59877/FCXC3888
  - departing staff has a procedure where chemicals are transferred to another person

  - there is a chemical sharing system/inventory in place, chemicals are shared with others and ordered in minimal amounts
  - equipment is shared with others via google calendar

  - there are standard operating procedures (SOPs) for all devices

  - waste separation takes place
  - glass weighing boats to replace plastic one

- After the process (takes roughly about 1h), consider giving out the accreditation or in case things are missing to delay it until further notice
- Write a concluding email to the responsible sustainability manager and the lab manager covering all the points of action, that might be missing (cc other LEAF coordinators and administrators)
- Ask also if criteria in the LEAF software are unclear that those need to be updated by the users, specifically ask for confirmation for these changes before accepting the submission for the medal
- After you received the confirmation *via* email from the sustainability lab manager (on action points within the lab as well as updating the criteria in the online tool), you can approve the submission
- Send a final email to the lab manager and sustainability manager and congratulate them for a greener lab.
  - Mention in that email that the baseline and progress calculators withing the LEAF software need to be filled in to advance to a silver level (this has to be checked in the next step)
- Irregular and spontaneous checks of labs will take place throughout the year if they comply with the requested changes and the LEAF accreditation
- Each lab needs to apply annually for new accreditation, so new checks should be conducted in the beginning of each year, which should be faster as the level of accreditation should have been maintained throughout the year
- It could be that lab users need a reminder for receiving a new accreditation in January of each year, which also may enhance engagement to advance to a higher level, the new check occurs the same as previous ones

2.7.2.2 Checklist for finishing group members: Postdocs, PhD-students, and Master students

Example of Feringa group, Stratingh Institute for Chemistry

Responsibilities	Checked by student	Checked by supervisor
All vials have been labeled and stored properly. A list of all compounds to be stored has been compiled.*		
All relevant synthesized chemicals have been passed on to another PhD student.*		
All personal commercial chemicals have been relocated		
The N_2 line has been cleaned		
The fume hood has been cleared and cleaned		
The personal drawer and bench have been cleared and cleaned		
<u>If using an oil pump</u>: the oil has been changed and the vacuum has been checked	Serial no pump:<br>Lowest vacuum: mbar	
<u>For membrane pumps</u>: the vacuum has been checked	Serial no pump:<br>Lowest vacuum: mbar	
All paper lab books have been handed to the supervisor and a pdf has been made.		
All analytical data has been properly stored**		
White lab card and office key have been given to responsible person		Checked by responsible person:

* For compiling the list, please include: experiment number, date of synthesis, chemical structure, amount of the product, the appearance and any other extra helpful comments. The list also needs to contain the newly assigned owner. Please discuss proper location of samples with your lab manager/supervisor/PI. The list should also be provided to the laboratory manager to be added to the chemical database. Use a label writer to label the vials.

**According to the group data management plan and in agreement with your supervisor/PI.

Name: Name of supervisor:
Personal email for future contact:
Signature of student Signature of supervisor

2.7.2.3 Accreditation award and door signs.

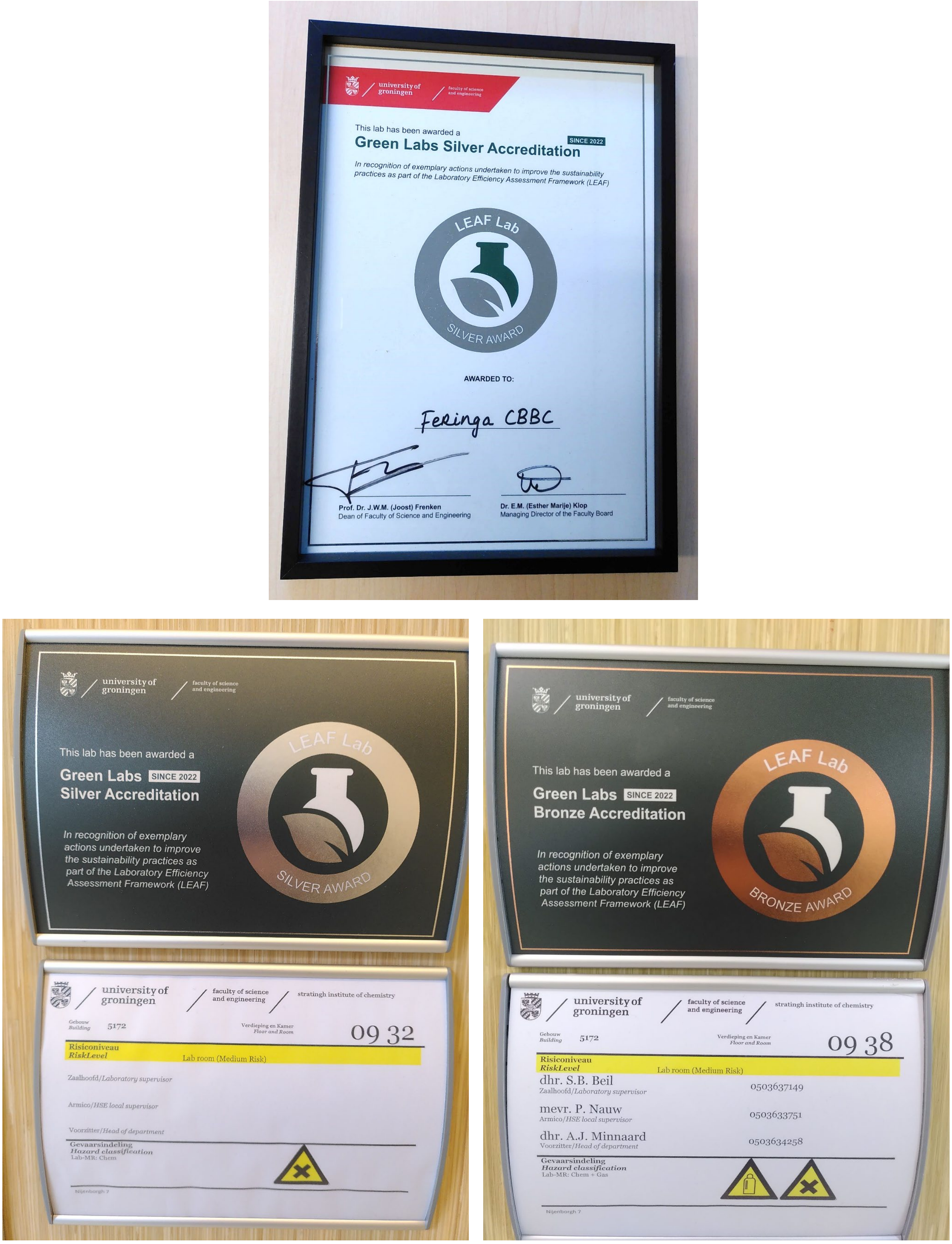

Figure S18: Green Labs Accreditation through LEAF. Award being presented to each laboratory (top). Silver and bronze door signs next to location and description of laboratory (bottom).

2.7.3 Energy measurements, list of equipment, funding

Energy fund (2023)

In 2023 many lab groups of FSE have applied for an energy fund to replace inefficient laboratory equipment, of which the majority has been approved. Many old devices will be substituted for more energy-efficient ones, which will not only improve job satisfaction but also save more than 50 000 kWh on a yearly basis.

2.7.3.1 Fume hoods

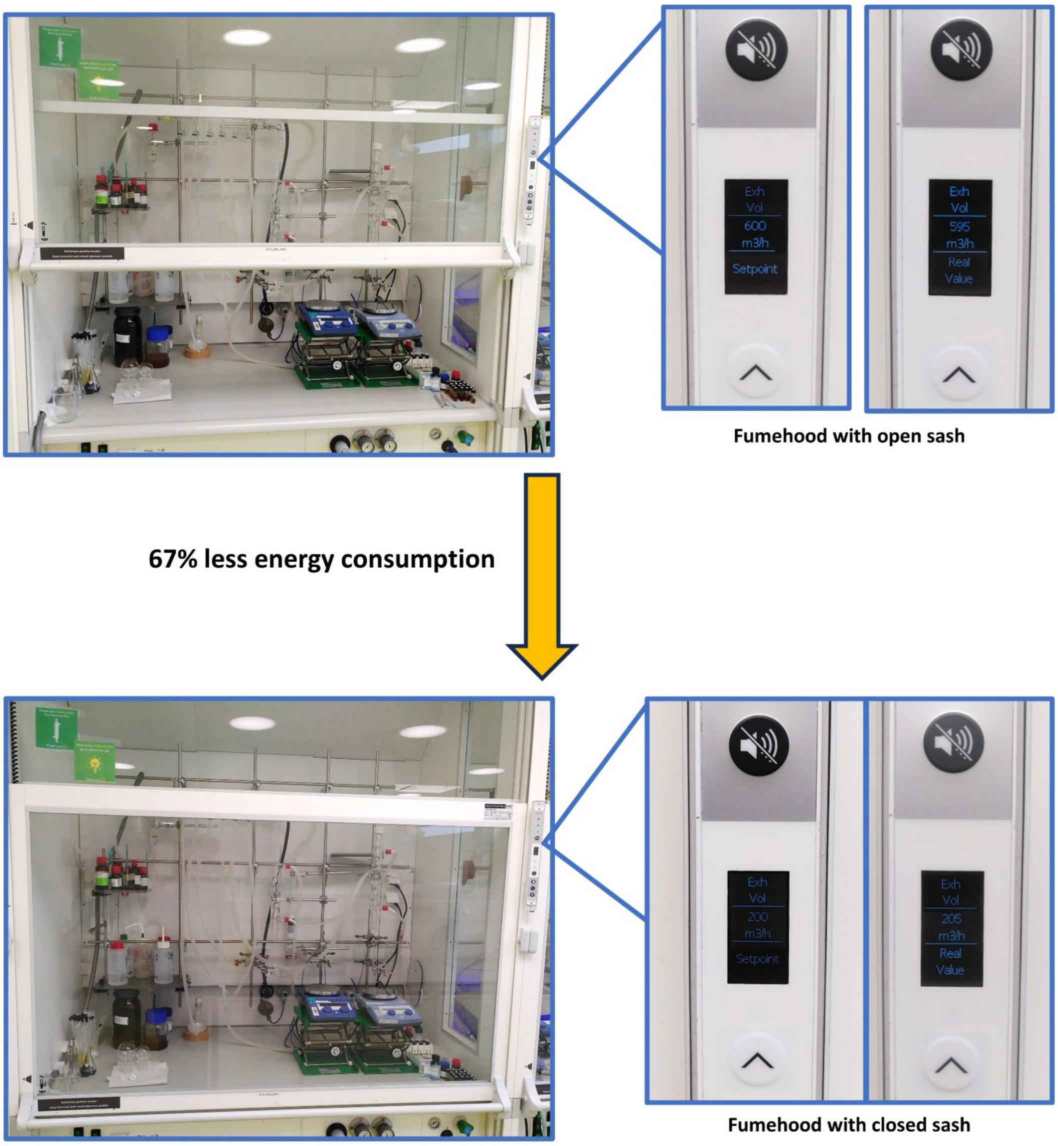

Figure S19: Keep fume hoods closed as much as possible, it reduces energy consumption to supply input air by 67%.

Note: Most fume cupboard fan systems work at a constant rate and reduction in flow rate of a single fume hood is not the same as reduction in energy. Extract fans on the roof probably do not ramp down when a fume cupboard sash is lowered but operate at a constant power. However, less energy may be required to supply input air by closing the sashes *via* reduction of flowrates. Ultimately, we recommend night setbacks on the fans on the roof to reduce energy consumption most effectively.

Table S35: Measurements on automated closing time of fume hood sashes in different laboratories.

Fumehood Experiment	Sash opening stand	Start Time	Closing Time	Time (min)
**Lab 932**				
Fumehood left	High	0:14s	5:15s	5
Fumehood left	Middle	0:14s	5:15s	5
Fumehood Middle	High	0:16s	5:29s	5
Fumehood Middle	Middle	0:14s	7:14s	7
**Lerch lab**				
Fumehood left	Middle	0:14s	3:18s	3
Fumehood Middle	High	0:14s	3:18s	3
Fumehood Right	Middle	0:14s	3:18s	3
**Total average closing time for automated sensor**				**4.4 min**

In new laboratories fume hoods are often equipped with a light sensor and an automated closing system. Here the sash closes automatically after 5 minutes. However, lab users should check regularly on the light sensors ensuring they are still functional to keep the automated closing system running. This automated closing system should stay enabled at all times and not be turned off. Any issues should be reported to facilities management immediately.

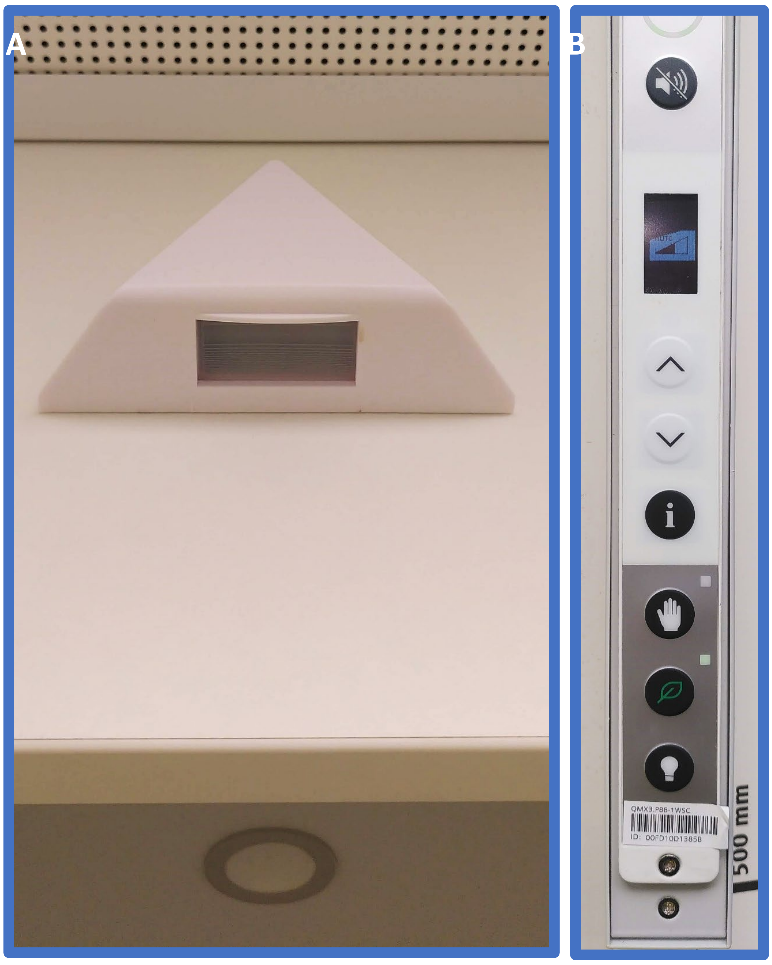

Figure S20: a) Ensure that the light sensors are functional at all times. b) Do not change the settings of the fume hood to keep the automated closing system enabled.

2.7.3.2 Ultra-low temperature (ULT) freezers

Table S36: Energy consumption and measurements of ULT freezers at –80 °C or –70 °C. *The following data has been acquired through R.J.W. Visser, N. Elzinga, D. Lijdsman, T. Freese, M. Kammuller, and R. Kat. and is being published with permission of the Faculty of Science and Engineering, University of Groningen. a) Energy consumption before freezer cleaning. b) Energy consumption after freezer cleaning.*

Model	Energy use (average per year kWh)	Energy use (average per day kWh)	Manufacturers energy consumption (kWh)	Temperature setpoint	Volume	$W L^{-1} d^{-1}$
Eppendorf CryoCube FC570	2781	7.6	10.5	-70	570	13.4
Eppendorf CryoCube FC660	3614	9.9	10.6	-70	660	15.0
Eppendorf / New Brunswick U570	4163	11.4	11.8	-80	570	20.0
Eppendorf / New Brunswick U410	2560	7.0	10.8	-70	410	17.1
Eppendorf / New Brunswick U725	4475	12.3	15.8	-70	725	16.9
Eppendorf / New Brunswick U410	5217	14.3	10.8	-70	410	34.9
Eppendorf / New Brunswick U570	3598[a]	9.9	10.8	-70	570	17.3[a]
	3222[b]	8.8				15.5[b]
Haier DW-86W420	4419	12.1	12.5	-70	420	28.8
Haier DW-86L628E	4062	11.1	18.0	-80	626	17.8
Haier DW-86L628E	4068	11.1	18.0	-70	626	17.8
Haier DW-86L628	5561	15.2	18.0	-70	626	24.3
New Brunswick C66085	4379	12.0	14.3	-70	660	18.2
New Brunswick, U410-86HEF	2181	6.0	10.8	-70	410	14.6
New Brunswick U101 -86	2279	6.2	8.8	-70	101	61.8
New Brunswick, U410 premium	3619	9.9	10.8	-80	410	24.2
New Brunswick, C340 premium	4252	11.6	8.8	-70	340	34.3
New Brunswick C660 HEF	3488	9.6	11.4	-70	660	14.5
New Brunswick C340-86	3393	9.3	10.8	-70	410	22.7
New Brunswick U725-86	3704	10.1	15.8	-70	725	14.0
New Brunswick U410-86	2358	6.5	8.8	-70	340	19.0
New Brunswick Scientific, U570-86 EU	5107	14.0	11.8	-80	570	24.5
New Brunswick High Efficiency C660	3144	8.6	11.4	-70	660	13.1
New Brunswick C660	3999	11.0	14.3	-70	660	16.6
New Brunswick C660	3786	10.4	14.3	-70	660	15.7
New Brunswick C660 Premium	3460	9.5	14.3	-70	660	14.4
New Brunswick C340 Premium	2752	7.5	8.8	-70	340	22.2
New Brunswick Innova C585	3405	9.3	13.6	-70	585	15.9
New Brunswick Innova U535	4521	12.4	13.2	-70	535	23.2
New Brunswick C542-85	4235	11.6	n/a	-80	542	21.4
New Brunswick Model C585	2580	7.1	13.6	-70	585	12.1
New Brunswick C340-86	7743	21.2	8.8	-80	340	62.4
New Brunswick C340-86	7299	20.0	8.8	-80	340	58.8
New Brunswick U410 -86	4599	12.6	10.8	-80	410	30.7
New Brunswick Scientific C542-85	3651	10.0	10.0	-70	542	18.5
Panasonic MDF- DC500VX-PE	4337	11.9	15.5	-70	575	20.7
Panasonic MDF-594-PE	6461	17.7	21.6	-70	487	36.3
Panasonic MDF-DC500VX-PE	4906	13.4	15.5	-70	575	23.4
Sanyo MDF-394	3664	10.0	13.2	-70	309	32.5
Sanyo MDF-594	6804	18.6	21.6	-70	487	38.3
Sanyo MDF-594	7342	20.1	21.6	-80	487	41.3
Sanyo Ultra Low MDF-594	7475	20.5	21.6	-70	594	34.5
Sanyo MDF-U73V	7789	21.3	21.4	-70	728	29.3
Sanyo MDF-U33V	3707	10.2	9,2	-80	333	30.5
Sanyo MDF-U33V	3961	10.9	9,2	-80	333	32.6
Sanyo MDF-794	9778	26.8	21.1	-70	701	38.2
Thermo Scientific Model 5819	3999	11.0	13.0	-70	481	22.8
Thermo Scientific TSX400V	3322	9.1	9.1	-70	548	16.6

2.7.3.3 Information on how to check freezer efficiency

<u>**Recommendations:**</u>

• Prioritize ULT freezers larger than 500 L, which consume 10 kWh per day or less.

Performance
**Bad energy efficiency (> 30 watts / Liter / day)**
**Critical energy efficiency (25 < x < 30 watts / Liter / day)**
**Medium Energy efficiency (20 < x < 25 watts / Liter / day)**
**Good energy efficiency (13.5 < x < 20 watts / Liter / day)**
**Great energy efficiency (< 13.5 watts / Liter / day)**

Increasing the temperature of a –80 °C freezer to **–70 °C** reduces its energy consumption up to 30%.

Example images of well-maintained freezers:

https://www.freezerchallenge.org/uploads/2/1/9/4/21945752/minus-70-is-the-new-minus-80_3.pdf

https://www.nature.com/articles/d42473-021-00361-7

https://www.exeter.ac.uk/about/sustainability/sustainablelabs/energy/ultfreezers/

<u>Further actions:</u>

- Check if people conduct maintenance i.e. cleanup of the freezer and defrosting twice per year.
- Check if door seals are not blocked by excess ice or frost, and close securely.
- Check if they clean filters from dust accumulation every 3 months as it increases energy consumption by 14%-25%. Check if the filters are clean.
- Have proper spacing for the freezer and assess the ambient temperature of the room.
- Open and close the doors quickly (less than 30-45 seconds) to avoid frost buildup and a rise in temperature. Well-maintained freezers have inner doors that are able to be closed / with not too much ice on them.
- Keep an updated inventory list of samples inside the freezer and sort out unneeded samples.

Replacement of inefficient freezers:

As of March 2024, all ULT-freezers at the Faculty of Science and Engineering have been measured to assess energy efficiency. Inefficient models were evaluated against the years for return of investment (ROI). When the ROI was assessed to less than 8 years, the Green Lab team is planning to replace such models in 2024. The collective expected costs, without possible discounts, for 7 models determined is 94 368.69 €. By replacing these the faculty will save up to 35 017 kWh / year. Thus, the ROI for all 7 models together will be to (94 368.69 € / (35 017 kWh * 0.30€ =) 8.98 years.

2.7.3.4 Miscellaneous laboratory devices and equipment

Table S37: Energy consumption of various devices and equipment in laboratory spaces. *The following data has been acquired through T. Freese and J. Y. de Boer and is being published with permission of the Faculty of Science and Engineering, University of Groningen.*

Equipment (Lab 932, Feringa)	Temperature or other specifications	Power Usage (W)	Energy Usage (kWh)	Energy Average per day (kWh)	Energy average per year (kWh)	Annual kWh when idle
Fridge #1	5 degrees (13W)	-	5	0.72	262	-
Fridge #2	7 degrees (14.1W)	-	8.6	0.73	265	-
Balance	3.4W when not in use	-	0.7	0.06	21	29.78
Balance	2.0W when not in use	-	0.5	0.04	15	-
Ultrasonic bath	0.3W when not in use	-	0	0.00	0	-
Vortex	0.0W when not in use	-	0.3	0.02	9	-
pH meter	-	-	-	-	-	0
Oven	-	-	-	-	-	0
TLC lamp	-	-	-	-	-	0
**Lab NB (Feringa)**						
**Stirring plates**						
IKA basic	Metal heating block	-	0.10	1.91	698.40	-
IKA basic standard 1.0	Metal heating block	-	0.07	1.52	556.07	-
IKA basic standard 1.0	Oil bath	-	0.18	1.93	704.69	-
IKA basic standard 2.0	Metal heating block	-	0.16	1.74	634.61	-
**Rotary evaporator bath**						
Buchi R-210 *with balls*	55.7 reduced energy	-	0.18	1.46	531.44	-
Buchi R-210 *without balls*	-	-	0.11	3.28	1198.37	-
Heidolph Hei-VAP	-	-	0.08	1.40	512.62	-
Buchi R-300	-	-	0.08	1.31	478.88	-
IKA RV8	-	-	0.03	1.54	561.85	-
**Oven**						
Thermo Scientific oven	-	-	0.57	4.80	1750.98	-
**Energy Measurements (Lerch)**						
Rotavap (both)	only right thing uses energy	-	0.5	0.06	20	-
Balance	uses energy when off, multiplug (4.2 W)	-	0.4	0.04	16	-
Orbital Mixer	-	-	0	0.00	0	-
Shutter	Dymax, 0.3 W, Multiplug	-	2	0.22	82	-
Oven	-	-	4.6	0.51	188	-
Goedhoudt Airconditioning	-	-	19.1	2.68	980	-
**Life sciences**						
Gallenkamp incubator with fan	50°C	302.20	0.23	2.56	933.95	-
Marius Utrecht incubator 28°C	-	443.80	1.16	0.99	360.42	-
WTC Binder incubator 37°C	60.4 W low; 620 W high	63.50	0.19	1.46	531.25	-
Inventum incubator 50°C	-	385.80	0.96	1.21	440.83	-
Marius Utrecht incubator 28°C	-	213.50	0.20	1.97	718.68	-
New Brunswick shaker	-	33.80	0.16	0.79	288.53	-
New Brunswick shaker with cooling	69.4 W; 49.3 W low; 741.2 W high	69.40	1.04	1.27	462.01	-

Vacuum pumps

The vacuum pumps of the Feringa group can be dived in two categories, oil pumps and membrane pumps. Oil pumps draw significantly more power and are less chemical resistant than membrane pumps. However, the amount of vacuum it can reach is way lower (around 10-3 mbar compared to 10-20 mbar). Thus, still important for our research.

The power draw of vacuum pumps is stable under the same load. However, more power is required to push air though a vacuum pump. The pump was run for 10 min to reach stable state. Afterwards the closed power draw was noted. Then, the vacuum pump was opened to air to measure the open power draw.

Results oil pumps Type	Power usage (open)	Power usage (closed)
**Duo M6 Oilpump**	505 W	315 W
**Atlas copco**	310 W	265 W
**Edwards 3**	280 W	270 W

Results membrane pumps Type	Power usage (open)	Power usage (closed)
**Heidolph valve control**	240 W	199 W
**KNF N820**	98 W	92 W
**KNF**	130 W	37 W
**KNF N840**	180 W	162 W

Stirring and heating plates, metal heating blocks as oil bath replacements

Stirring plates have a peak load in the heating phase, followed by a periodic load to maintain the temperature. Measuring the peak load is not insightful since this depends on the heating element inside of the plate. Thus, only the efficiency of maintaining a certain temperature during the periodic load was measured.

For all measurements the same heating block/ oil bath was used. Energy consumption in kWh was measure over a period of at least one hour. Afterwards the kWh was divided by the number of hours to obtain the average amount of watt.

Results heating block at 100°C and 1000 RPM (* oil bath)

Type	Power usage
**Ika basic**	80 W
**IKA basic standard 1.0**	63 W
**IKA basic standard 1.0***	80 W
**IKA basic standard 2.0**	72 W

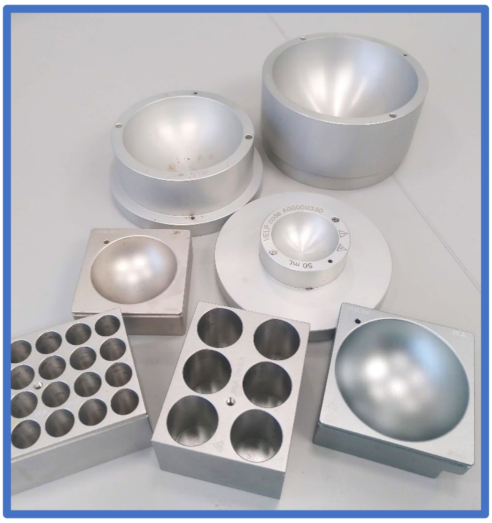

Figure S21: Metal heating block as efficient and safe replacement for oil heating baths. *Image reproduced from previously published guidebook (https://doi.org/10.26434/chemrxiv-2023-q3lmq-v2).*

Rotary evaporators

The power draw of the rotary evaporator depends on three factors. The vacuum pump, water bath and the rotating speed. The power draw of the vacuum pumps was tested before and the power draw of the rotation is not very signification (around 10 Watts max.). Thus, only the water baths were tested. Tests were performed similar to the heating plates.

For all measurements the bath was set to 60°C. The energy consumption was measured in kWh for at least one hour. Afterwards the kWh was divided by the number of hours to obtain the average amount of watt.

Results rotary evaporator baths (*without condensation balls)

Type	Power usage
**Buchi R-210**	61 W
**Buchi R-210***	138 W
**Heidolph hei-vap**	58 W
**Buchi R-300**	55 W
**IKA RV 8**	64 W

Ensure water baths have a cover. For instance, use plastic water bath balls for rotary evaporators to cover the open water (**Figure S22**). They prevent heat loss, evaporation (up to 90%), reduce oxygen input, reduce odors and levels of algae growth in the water.

- Avoid running the rotary evaporator overnight, unless required.

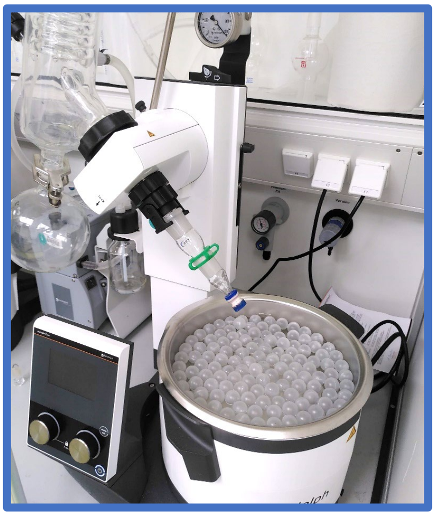

Figure S22: Plastic balls in the water bath of a rotary evaporator to prevent energy loss in form of heat. *Image reproduced from previously published guidebook (https://doi.org/10.26434/chemrxiv-2023-g3lmq-v2).*

Oven

An oven works very similar to the stirring plates as well. Energy consumption was measured overnight at 135 °C.

Type	Power usage
**Thermo scientific**	200 W

Conclusion

The difference in energy consumption within each category is not very significant when adjusting for the specification. For example, the difference within the membrane pumps is rather big 37-199 Watts. However, the more efficient one is not designed for a rotary evaporator.

The latest efforts to buy heating blocks and the condensation balls have a way bigger impact than a more efficient device (*i.e.* pumps or oven) could achieve.

2.7.3.5 Devices and their power distribution in a laboratory

Table S38: Energy consumption measurements of each device present in a research laboratory. Each device was measured for 40 days. As fume hoods were not included in the measurement, ULT freezers are the highest consuming equipment in the lab space measured. *The following data has been acquired through R.J.W. Visser and is being published with permission of the Faculty of Science and Engineering, University of Groningen.*

Device	Type	NEN code	Total acc. power consumption (kWh)	Percentage of total	Annual consumption (kWh)
**NB –77 °C**	–80°C freezer	20156	848.6	24.5	7743
**NB –77 °C**	–80°C freezer	21055	799.9	23.1	7299
**Freezer + cold trap + speedvac.**	Freezer	29508	306.1	8.8	2793
**NB –70°C**	–80°C freezer	24808	282.7	8.2	2579
**Oven Depex 100 °C**	Oven	21014	137.6	4.0	1256
**PCR nanodrop + PC**	PCR	29511	109.0	3.1	995
**Crate freezer**	Freezer	22044	89.3	2.6	815
**Ice machine**	Ice machine	24943	87.2	2.5	795
**Fridge R18**	Fridge	21020	59.1	1.7	539
**Oven Sanyo incubator**	Oven	21120	57.0	1.6	520
**Fridge R18 MC0123**	Fridge	26899	53.7	1.5	490
**Freezer WK6**	Freezer	20948	49.4	1.4	451
**PC001342**	PC (incl. screen)	none	49.1	1.4	448
**Biorad FPLC+PC**	System	004355,015411,26883,26844	43.6	1.3	398
**Fridge R5H**	Fridge	29514	31.4	0.9	287
**Freezer VK5**	Freezer	24970	31.0	0.9	283
**Panasonic 37°C incubator**	Incubator	26077	29.4	0.8	268
**Freezer MI051**	Freezer	27015	29.0	0.8	265
**FreezerWK15**	Freezer	24979	29.0	0.8	265
**Autoclave**	Autoclave	21521	27.6	0.8	252
**Autoclave**	Autoclave	20964	26.4	0.8	241
**Freezer VK20**	Freezer	26898	20.5	0.6	187
**Water Boiler**	Boiler		19.5	0.6	178
**Fridge R1K**	Fridge	26896	18.9	0.5	172
**Fridge KK3**	Fridge	24969	17.7	0.5	162
**Fridge R15**	Fridge	21249	17.2	0.5	157
**MilliQ**	MilliQ	26902	17.2	0.5	157
**Fridge 390 (Below R18)**	Fridge	20955	17.2	0.5	157
**Fridge R17**	Fridge	26919	16.8	0.5	153
**Freezer VK9**	Freezer	24967	16.8	0.5	153
**Eppendorf centrifuge**	Centrifuge	26910	16.4	0.5	150
**IKA Stirrer**	Stirrer	26877	15.5	0.4	141
**M1031**	Fridge	26911	15.1	0.4	138
**Fridge R4**	Fridge	24966	14.5	0.4	132
**Fridge R3**	Fridge	24968	11.6	0.3	106
**PC Screen AOC**	Monitor	41056	10.4	0.3	95
**Sharp amplifier**	Entertainment	21048	9.8	0.3	89
**VWR heatblock**	Heat block	26099	6.5	0.2	60
**Synergy Plate Reader**	Other	27018	6.2	0.2	56
**Weighing scale**	Scale	20978	5.9	0.2	53
**Memmert 25°C incubator**	Incubator	26076	4.8	0.1	44
**Lenovo ThinkCentre**	PC	41045	2.9	0.1	27

**Ker balance**	Scale	26374	2.7	0.1	25
**2x PS Bunsen burner**	Transformer	20916	1.9	0.1	18
**2x PS Bunsen burner**	Transformer	20918	1.9	0.1	18
**Sartorius scale**	Scale	21507	1.2	0.0	11
**Centrifuge**	Centrifuge	26894	0.8	0.0	8
**Licor Odessey**	Other	27017	0.6	0.0	5
**Biorad powerpack 300**	Other	20957	0.5	0.0	5
**Eppendorf centrifuge**	Centrifuge	29515	0.5	0.0	4
**Centrifuge Sorvallegend**	Centrifuge	21018	0.15	0.0	1
**Biorad powerpack 200**	Other	28414	0.1	0.0	1
**IKA magnetic stirrer reo.**	Stirrer	21469	0.0	0.0	0
**Labinco vortex**	Vortex	24973	0.0	0.0	0
**Microman pipette boy**	Small	none	0.0	0.0	0
**Egg centrifuge**	Centrifuge	21304	0.0	0.0	0
**Roth egg centrifuge**	Centrifuge	26075	0.0	0.0	0
**Salm and kipp.**	Shaker	21039	0.0	0.0	0
**VWR vacuum pump**	Pump	26886	0.0	0.0	0
**Fridge MI052**	Fridge	27015	0.0	0.0	0
**LG Shaker**	Shaker	26889	0.0	0.0	0
**Martin Hume Sealer**	Vacuum Sealer	26892	0.0	0.0	0
**Mick1 Stirrer**	Stirrer	20903	0.0	0.0	0
**Pipette boy MI006**	Small	26880	0.0	0.0	0
**Schneiders Vortex**	Vortex	21027	0.0	0.0	0
**Vacuum pump**	Pump	26881	0.0	0.0	0
**Freezer + coldtrap + speedvac.**	Freezer	29516	0.0	0.0	0
**Sum**			**3468 kWh**	**100%**	**31 645 kWh**

2.7.3.6 Computer screens

Table S39: Power consumption measurements on a computer screen with different brightness levels. *The following data has been acquired through R. Pollice and T. Freese and is being published with permission of the Faculty of Science and Engineering, University of Groningen.*

Power (W)	Brightness	Energy per day (kWh)	Price per day (€)	Energy per day for 1000 screens (kWh)	Price per day for 1000 screens (€)	Energy per day for 10 000 screens (kWh)	Price per day for 10 000 screens (€)
13.5	0	0.108	0.03	108	32.4	1080	324
17.3	25	0.138	0.04	138.4	41.52	1384	415.2
21.4	50	0.171	0.05	171.2	51.36	1712	513.6
25.7	75	0.206	0.06	205.6	61.68	2056	616.8
30.4	100	0.243	0.07	243.2	72.96	2432	729.6

As the University of Groningen has 6390 employees, it can be assumed that at least 6390 computer screens exist in the office spaces (probably more due to multiple screens). If all employees would reduce their screen brightness from 100% to 75% the UG would save 23 324 € annually while consuming 86 297 kWh less energy (equivalent to the annual energy consumption of 27-35 Dutch households, where one average household consumes 2500 - 3200 kWh per year: https://www.cbs.nl/en-gb/figures/detail/81528ENG).

Table S40: Power consumption and costs of all (single) computer screens (i.e. monitor) for 6390 employees at the UG. Correlation and costs savings are depicted for various brightness levels.

UG consumption for 6390 screens					
**power [W]**	**brightness**	**energy per day [kWh]**	**energy per year (kWh)**	**Price per day (€)**	**Price per year (€)**
13.5	0	690.12	251 894	191.7	69 971
17.3	25	881.82	321 864	255.6	93 294
21.4	50	1092.69	398 832	319.5	116 618
25.7	75	1316.34	480 464	383.4	139 941
30.4	100	1552.77	566 761	447.3	163 265
**Annual savings for brightness from 100 to 75%.**			**86 297 (kWh)**		**23 324 €**

2.7.4 Winter break closure 2022-2023 at FSE

The following data has been acquired through R.J.W. Visser and is being published with permission of the Faculty of Science and Engineering, University of Groningen.

For financial and environmental reasons, the buildings of FSE were closed for two weeks during the Christmas period 2022-2023. From Friday 23-12-2022 till Monday 9-1-2023 the buildings of the faculty were in 'weekend'- mode and only accessible with permission. Furthermore, all personnel were asked to postpone experiments, switch off equipment, close curtains etc. to reduce energy consumption.

In the following sections the energy consumption during this last Christmas period is compared to the energy consumption over the previous four winter break periods. Our main focus is the difference of the "total" closure compared to an "normal" Christmas holiday period (2018-2021) where a lot of people take two weeks off due to vacation, but when the buildings and research operate more or less normally.

As Christmas is on fixed dates differences due to it being in a weekend or in the middle of the week could be observed. In **Figure S23** is depicted how the two-week periods to compare in relation to the dates of the holidays.

vrijdag 21 december 2018	vrijdag 20 december 2019	vrijdag 18 december 2020	vrijdag 17 december 2021	vrijdag 16 december 2022	vrijdag 15 december 2023
**zaterdag 22 december 2018**	**zaterdag 21 december 2019**	**zaterdag 19 december 2020**	**zaterdag 18 december 2021**	**zaterdag 17 december 2022**	**zaterdag 16 december 2023**
**zondag 23 december 2018**	**zondag 22 december 2019**	**zondag 20 december 2020**	**zondag 19 december 2021**	**zondag 18 december 2022**	**zondag 17 december 2023**
maandag 24 december 2018	maandag 23 december 2019	maandag 21 december 2020	maandag 20 december 2021	maandag 19 december 2022	maandag 18 december 2023
dinsdag 25 december 2018	dinsdag 24 december 2019	dinsdag 22 december 2020	dinsdag 21 december 2021	dinsdag 20 december 2022	dinsdag 19 december 2023
woensdag 26 december 2018	woensdag 25 december 2019	woensdag 23 december 2020	woensdag 22 december 2021	woensdag 21 december 2022	woensdag 20 december 2023
donderdag 27 december 2018	donderdag 26 december 2019	donderdag 24 december 2020	donderdag 23 december 2021	donderdag 22 december 2022	donderdag 21 december 2023
vrijdag 28 december 2018	vrijdag 27 december 2019	vrijdag 25 december 2020	vrijdag 24 december 2021	vrijdag 23 december 2022	vrijdag 22 december 2023
**zaterdag 29 december 2018**	**zaterdag 28 december 2019**	zaterdag 26 december 2020	zaterdag 25 december 2021	**zaterdag 24 december 2022**	**zaterdag 23 december 2023**
**zondag 30 december 2018**	**zondag 29 december 2019**	**zondag 27 december 2020**	zondag 26 december 2021	zondag 25 december 2022	**zondag 24 december 2023**
maandag 31 december 2018	maandag 30 december 2019	maandag 28 december 2020	maandag 27 december 2021	maandag 26 december 2022	maandag 25 december 2023
dinsdag 1 januari 2019	dinsdag 31 december 2019	dinsdag 29 december 2020	dinsdag 28 december 2021	dinsdag 27 december 2022	dinsdag 26 december 2023
woensdag 2 januari 2019	woensdag 1 januari 2020	woensdag 30 december 2020	woensdag 29 december 2021	woensdag 28 december 2022	woensdag 27 december 2023
donderdag 3 januari 2019	donderdag 2 januari 2020	donderdag 31 december 2020	donderdag 30 december 2021	donderdag 29 december 2022	donderdag 28 december 2023
vrijdag 4 januari 2019	vrijdag 3 januari 2020	vrijdag 1 januari 2021	vrijdag 31 december 2021	vrijdag 30 december 2022	vrijdag 29 december 2023
**zaterdag 5 januari 2019**	**zaterdag 4 januari 2020**	**zaterdag 2 januari 2021**	zaterdag 1 januari 2022	**zaterdag 31 december 2022**	**zaterdag 30 december 2023**
**zondag 6 januari 2019**	**zondag 5 januari 2020**	**zondag 3 januari 2021**	**zondag 2 januari 2022**	zondag 1 januari 2023	**zondag 31 december 2023**
maandag 7 januari 2019	maandag 6 januari 2020	maandag 4 januari 2021	maandag 3 januari 2022	maandag 2 januari 2023	maandag 1 januari 2024
dinsdag 8 januari 2019	dinsdag 7 januari 2020	dinsdag 5 januari 2021	dinsdag 4 januari 2022	dinsdag 3 januari 2023	dinsdag 2 januari 2024
woensdag 9 januari 2019	woensdag 8 januari 2020	woensdag 6 januari 2021	woensdag 5 januari 2022	woensdag 4 januari 2023	woensdag 3 januari 2024
donderdag 10 januari 2019	donderdag 9 januari 2020	donderdag 7 januari 2021	donderdag 6 januari 2022	donderdag 5 januari 2023	donderdag 4 januari 2024
vrijdag 11 januari 2019	vrijdag 10 januari 2020	vrijdag 8 januari 2021	vrijdag 7 januari 2022	vrijdag 6 januari 2023	vrijdag 5 januari 2024
**zaterdag 12 januari 2019**	**zaterdag 11 januari 2020**	**zaterdag 9 januari 2021**	**zaterdag 8 januari 2022**	**zaterdag 7 januari 2023**	**zaterdag 6 januari 2024**
**zondag 13 januari 2019**	**zondag 12 januari 2020**	**zondag 10 januari 2021**	**zondag 9 januari 2022**	**zondag 8 januari 2023**	**zondag 7 januari 2024**

Figure S23: Time period calculations of two weeks winter break period.

Below, the summarized data is presented for the four main buildings of FSE, Nijenborg 4 (NB), Energy Academy (EA), Bernoulliborg (BB), and Linneausborg (LB) as for FSE total which also includes some smaller buildings.

Specifications of winter break:

Laboratories:

Temperature within *generally climate-controlled* laboratories will be reduced to a technically minimal and responsible level. Ventilation is not being adjusted.

Ventilation and room temperature of *specifically climate-controlled* areas, such as climate cells, laser laboratories, animal facilities etc. remain unchanged.

Offices:

Ventilation and the room temperature in offices will be reduced to a technically minimal and responsible level.

Both laboratories and offices:

All staff are requested to switch off all equipment, including computers, that can be safely switched off during the winter holiday closure.

Accessibility

If researchers need to access the research spaces, they are advised that accessibility of buildings will be comparable to accessibility during normal weekends.

There will be no company emergency response (BHV) during the holiday closure.

Table S41: Electricity, gas and costs savings through winter breaks and closures.

	**5100 NB4 electricity (kWh)**	**5100 NB4 gas (m³)**	**5158 EA (kWh)**	**5161 BB electricity (kWh)**	**5171 LB electricity (kWh)**	**5171 LB (m³)**	**FSE total electricity (kWh)**	**FSE total gas (m³)**
**Avg 2018-2021**	379 362	149 921	59 768	74 001	52 3682	9 603	1 019 461	160 489
**2022**	304 325	92 051	47 119	29 036	428 504	10 567	798 977	102 333
**Savings in units (kWh or m³)**	75 037	57 870	12 649	44 965	95 178	- 964	220 484	58 156
**Savings financially**<br><br>**gas m³ = €2.23**<br><br>**kWh = € 0.535**	40 145 €	129 050 €	6 767 €	24 056 €	50 920 €	– 2150	**117 959 €**	**129 687 €**

Note: Data presented is not corrected for outside air temperatures.

The large percentile and absolute differences in buildings presented can be explained by their age and isolation, but also by their function, such as offices *vs.* lab facilities or the presence of animal housings which limits the possibility of a 'total' closure.

Arguments for and against a 'total' closure during the Christmas period 2023-2024

Arguments in favor of 'total' closure

Environmental benefits: The equivalent of the annual use of 60 Dutch households is saved in two weeks.

Financial benefits: Compared to 2018-2021, FSE saved in 2022-2023 around 250 000 €.

Awareness: Climate and energy are back on the agenda for staff and students.

Arguments against a 'total' closure

Research: A negative effect on research is to be expected since experiments and research are on hold for two weeks.

Working from home: Some personnel cannot work from home. Our CAO obliges the UG to provide such staff with free leave hours if not provided with a place to work.

Not a universal holiday: Christmas is not a universal holiday, meaning the UG would expect students and staff to adhere to the Dutch holiday calendar.

Tenants: Some buildings have tenants, meaning a 'total' closure has to be negotiated for those buildings.

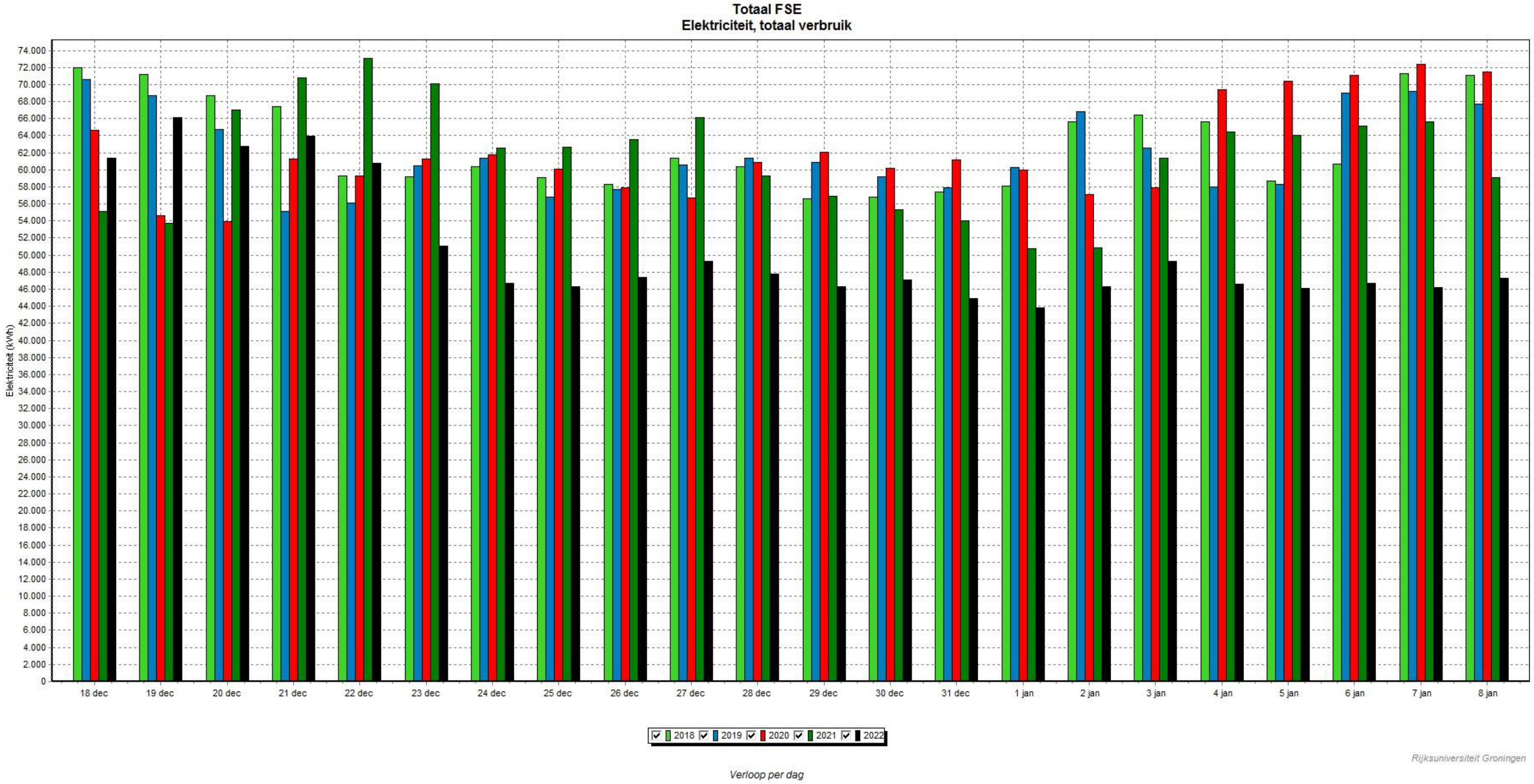

Figure S24: Electricity consumption during the winter breaks at FSE from 2018-2022.

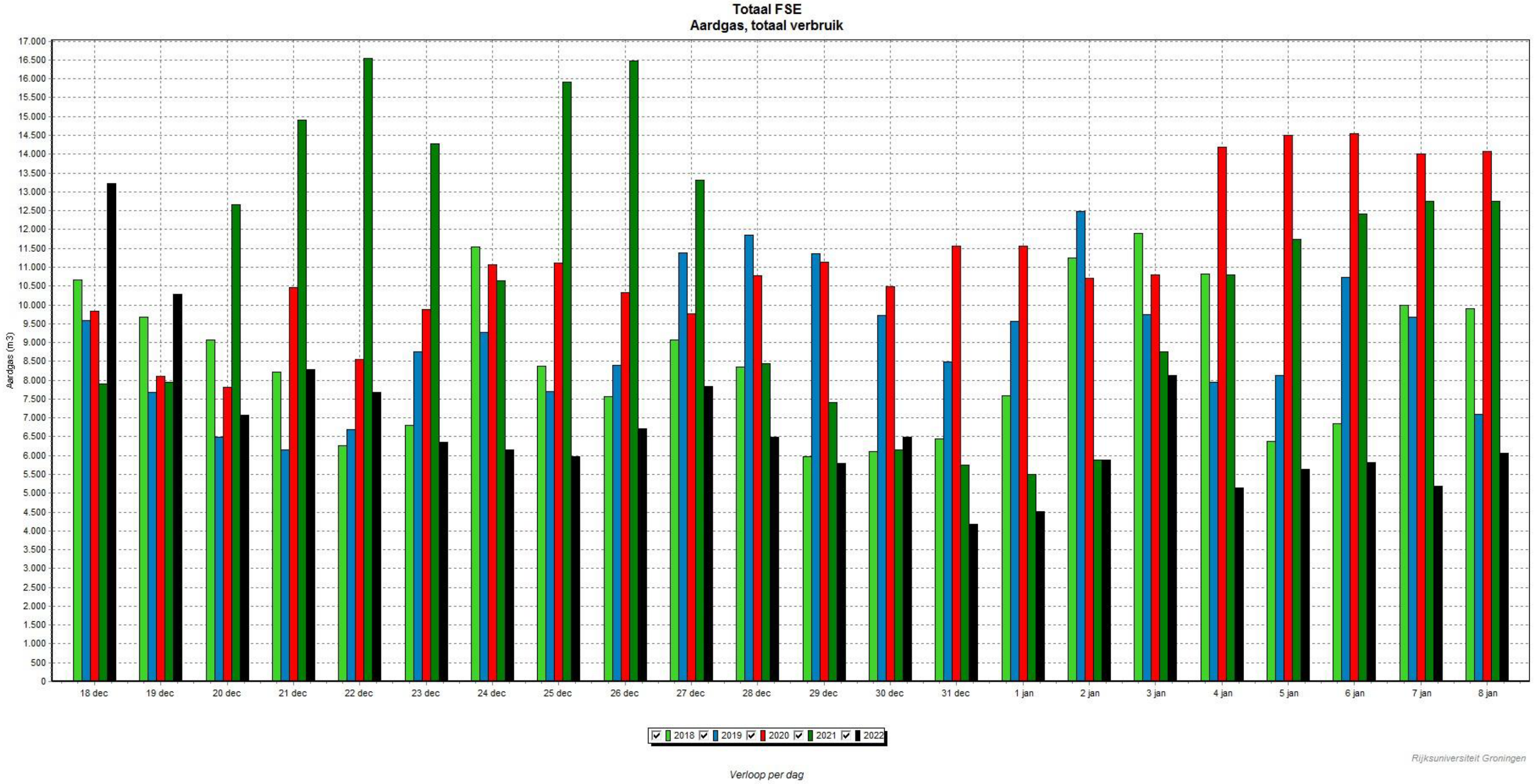

Figure S25: Gas consumption during the winter break at FSE from 2018-2022.

2.7.5 Travel data of the authors

Table S42: Travel data of B. L. Feringa from January 2022 – July 2023.

**Ben L. Feringa**						
**# of months**	**2022**	**from**	**to**	**via**	**remarks**	**CO2e emissions (kg)**
1	jan	-	-	-	-	-
2	feb	AMS	Dubai	-	2 way	3440
3	mrt	AMS	Vienna	-	2 way	632
4	apr	AMS	Vienna	-	2 way	632
5	mei	AMS	Bordeaux	-	2 way	602
6	jun	AMS	Valencia	-	2 way	856
6	jun	AMS	Helsinki	-	2 way	882
6	jun	AMS	Vienna	-	2 way	632
6	jun	AMS	London Heathrow	-	2 way	210
7	jul	AMS	Warsaw	-	2 way	724
8	aug	AMS	Vienna	-	2 way	632
9	sep	AMS	Reno, USA	Salt Lake City	2 way (both ways via SLC)	5423
9	sep	Hamburg	Stockholm	Copenhagen	1 way	323
9	sep	Stockholm	AMS	-	1 way	346
10	okt	AMS	Boston	-	2 way	2708
10	okt	AMS	El Paso	Atlanta	2 way (both ways via ATL)	4928
11	nov	AMS	Geneve	-	2 way	444
12	dec	-	-	-	-	-
	**2023**	**from**	**to**	**via**	**remarks**	
13	jan	AMS	Cardiff	-	2 way	442
14	feb	AMS	Bordeaux	-	2 way	602
15	mrt	AMS	Vienna	-	2 way	632
15	mrt	AMS	Londen Stansted		back to Eindhoven	194
16	apr	AMS	Los Angeles	-	1 way	3482
16	apr	LAX	San Francisco	-	1 way	147
16	apr	SFO	AMS	-	1 way	2344
16	apr	AMS	Washington DC	-	1 way	1805
16	apr	Washington DC	AMS	New York	1 way	1726
17	mei	AMS	Tel Aviv	-	2 way	1802
17	mei	AMS	Milaan Linate	-	1 way	246
17	mei	Geneve	AMS	-	1 way	222
17	mei	AMS	Gothenburg	-	2 way	526
18	jun	AMS	Barcelona	-	1 way	381
18	jun	Valencia	AMS	-	1 way	428
18	jun	AMS	Copenhagen	-	2 way	408
18	jun	AMS	Milaan Linate	-	2 way	492
18	jun	AMS	Stockholm	-	2 way	692
19	jul	AMS	Madrid	-	2 way	796
19	jul	AMS	Rome	-	1 way	373
19	jul	Rome	Londen Heathrow	-	1 way	367
19	jul	Londen Heathrow	AMS	-	1 way	105
**Total emissions (kg)**						**41 626**
**Total # of flights**						**67**
**# flights per month**						**3.53**
**Emissions per month (kg)**						**2191**
**# flights per year**						**42**
**Emissions per year (kg)**						**26290**
**Total CO2e emissions per year (kg)**						**324000**
**Air travel percentage of total carbon footprint**						**8.11%**

Table S43: Travel data of T. Freese, M. Heinemann, and M. M. Lerch from January 2022 – July 2023. N. Elzinga did not take business air travel during this time period.

Michael M. Lerch					
**Jan. 2022 - Jul. 2023**	**from**	**to**	**via**	**remarks**	**CO2e emissions (kg)**
Dec-22	AMS	Geneva	-	2 way	373
**Total emissions (kg)**					**373**
**Total # of flights**					**2**
**# flights per month**					**0.11**
**Emissions per month (kg)**					**20**
**# flights per year**					**1**
**Emissions per year (kg)**					**236**
**Total CO2e emissions per year (kg)**					**24000**
**Air travel percentage of total carbon footprint**					**0.98%**

Matthias Heinemann					
**Jan. 2022 - Jul. 2023**	**from**	**to**	**via**	**remarks**	**CO2e emissions (kg)**
n/a	AMS	Los Angeles	-	2 way	3056
**Total emissions (kg)**					**3056**
**Total # of flights**					**2**
**# flights per month**					**0.11**
**Emissions per month (kg)**					**161**
**# flights per year**					**1**
**Emissions per year (kg)**					**1930**
**Total CO2e emissions per year (kg)**					**38000**
**Air travel percentage of total carbon footprint**					**5.08%**

Thomas Freese					
**Jan. 2022 - Jul. 2023**	**from**	**to**	**via**	**remarks**	**CO2e emissions (kg)**
Jun-23	AMS	Los Angeles	-	1 way	1528
Jul-23	San Francisco	AMS	-	1way	1497
**Total emissions (kg)**					**3025**
**Total # of flights**					**2**
**# flights per month**					**0.11**
**Emissions per month (kg)**					**159**
**# flights per year**					**1**
**Emissions per year (kg)**					**1911**
**Total CO2e emissions per year (kg)**					**21000**
**Air travel percentage of total carbon footprint**					**9.10%**

2.7.6 Hazardous waste production at FSE

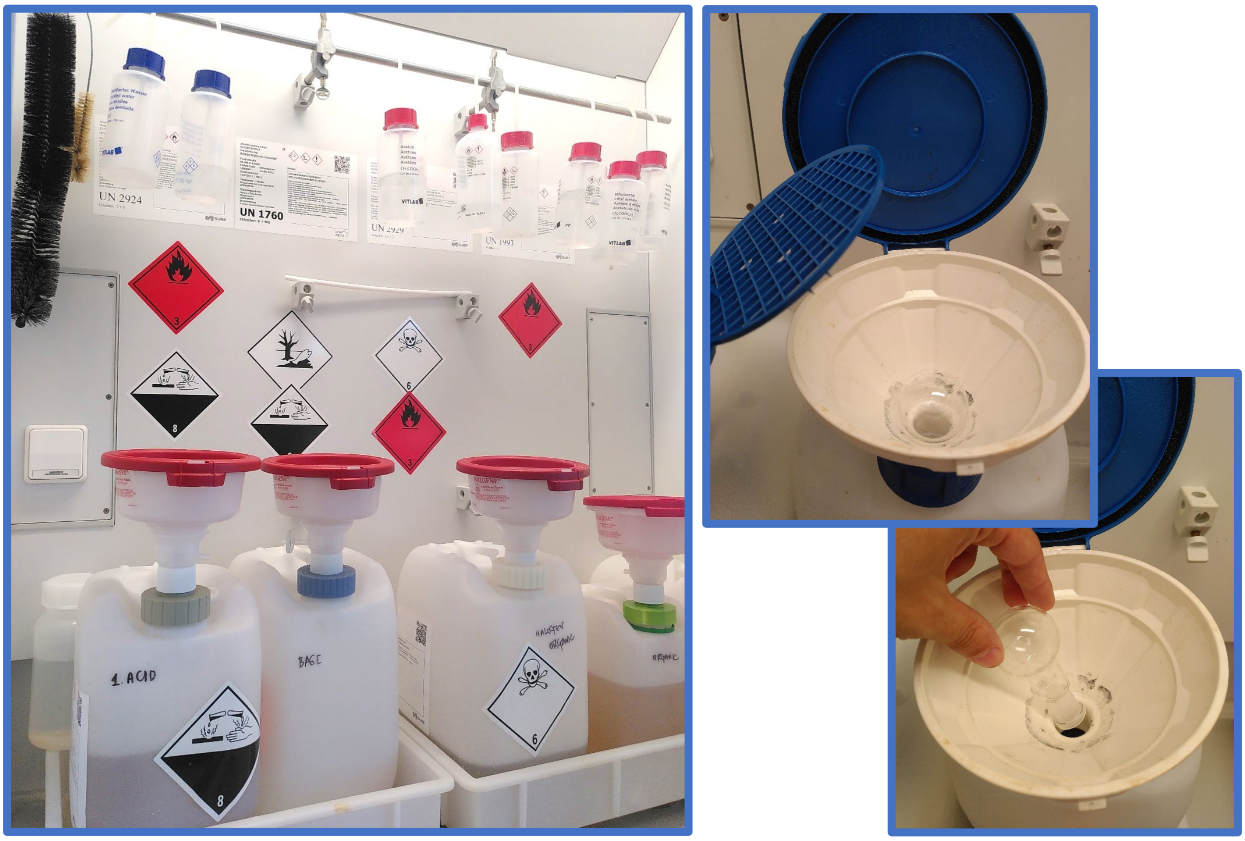

Figure S26: Funnels with lid to prevent solvent evaporation from waste canisters (left). Use of a round bottom flask to improve solvent loss by evaporation, which can serve as alternative to funnels with a lid (right). Another alternative to the round bottom flask is the plastic balls used to cover water baths in rotary evaporators, which can also be placed in a funnel to prohibit solvent loss by evaporation. *Image reproduced from previously published guidebook (https://doi.org/10.26434/chemrxiv-2023-g3lmq-v2).*

2.7.6.1 Education and student practicals

The following data has been acquired through N.N.H.M. Eisink and is being published with permission of the Faculty of Science and Engineering, University of Groningen.

Table S44: Hazardous liquid waste production in 6 months student practical in the academic year September 2021-February 2022, number of Students * days: 6390. *Calculated by N.N.H.M. Eisink, adapted by T. Freese.*

Solvents	Amount (L)	Per student per day (mL)
Acetone	1260	197.2
Ethanol (96%)	385	60.3
Ethanol (absolute)	15	2.3
Diethyl ether	201	31.5
Ethylacetate	82	12.8
Methanol	50	7.8
Pentane	175	27.4
Chloroform (D_1)	3.7	0.6
DMSO (D_6)	0.7	0.1
THF	27.5	4.3
**Total**	**6596 L**	**344 mL**

Table S45: Hazardous solid waste production (e.g. plastic waste production) in 6 months student practical in the academic year September 2021 – February 2022, number of Students * days: 6390. *Calculated by N.N.H.M. Eisink, adapted by T. Freese.*

Disposables	Amount	Unit	Individual items per unit	individual item per student per day	Sum
Aspirine tubes	69	Box	100	1	6900
Cuvets, halfmicro, plastic, 1 mL	59	Box	100	1	5900
Caps for aspirine tubes	92	Bag	100	1	9200
Eppendorf cups, 1.5 mL	30	Bag	500	2	15000
Eppendorf cups, 2 mL	23	Bag	500	2	11500
Greiner tubes 15 mL	10	Bag	50	0.08	500
Greiner tubes 50 mL, skirted	22	Bag	25	0.09	550
Greiner tubes 50 mL, unskirted	5	Bag	20	0.02	100
Hirschmann ring caps	11	4 boxes	1000	2	11000
Inj. Syringe, 2-piece, 1 mL	28	Box	100	0.4	2800
Inj. Syringe, 2-piece, 2 mL	14	Box	100	0.2	1400
Inj. Syringe, 2-piece, 20 mL	4	Box	100	0.1	400
Inj. Syringe, 2-piece, 5 mL	12	Box	100	0.2	1200
Neoprene gloves (L)	52	10 boxes	1000	8	52000
Neoprene gloves (M)	57	11 boxes	1000	9	57000
Neoprene gloves (S)	25	12 boxes	1000	4	25000
Neoprene gloves (XL)	15	13 boxes	1000	2	15000
Pasteur pipet 150 mm	75	Box	250	3	18750
Pasteur pipet 230 mm	39	Box	250	2	9750
Pipet tips blue 1 mL	21	Bag	1000	3	21000
Pipet tips clear 5 mL	6	Bag	1000	1	6000
Pipet tips yellow 250 µL	18	Bag	1000	3	18000
Test tubes 100 x 16 mm	32	box	100	1	3200
Test tubes 160 x 16 mm	75	box	100	1	7500

2.7.6.2 Plastic waste production Heinemann group in 2022

The following data has been acquired through S. R. Bonsing-Vedelaar and is being published with permission of the Faculty of Science and Engineering, University of Groningen.

Table S46: Plastic waste production in a molecular biology research group in 2022. *Calculated by S. R. Bonsing-Vedelaar, adapted by T. Freese.*

Currently used					Orders 2022	Unit	Amount	Total Amount	# per researcher per day	Weight per unit	Total weight	Total weight
Supplier	Cat. No.	Description	Price per unit	LCA score & type						weight (g)		Plastics only
VWR glove tender		Gloves, buy neoprene	€ 0.30 / glove	unknown	43 boxes	1000	43	**43000**	**7**	735	31605	
Sarstedt	95,953	Viltstift, waterproof, blauw	€ 0.73 / marker	unknown								
CBN	26140	neoTape-labelling tape, 19 mm, colored, 55 m long	€ 0.43 / meter	unknown	10					155	1550	
Manutan	9940737	Hydro-alcoholische gel Tork Premium S1	€ 13.92 / tube	unknown								
VWR	115-3473	Tube, 148 ml, Hand Medic®	€ 8.30 / tube	unknown								
VWR	115-9220	Bench Surface Protector	€ 1.00 / piece	unknown								
VWR	115-0031	Cleanroom-schoonmaakdoekje, droog, VWR® Spec-Wipe®, 3, Polyester/cellulose, wit, Cleanroomklasse: 100/ISO 5, l×b: 229×229 mm, 300 doekjes/zak	€ 0.07 / piece	unknown	2					1056.7	2113.4	
Sarstedt	95_64981	Storage box 9x9	€ 1.72 / glove	unknown	20					93.66	21120	
Fisscher scientific	16594994	X50 Disposable Face mask PP blue 3-ply Manufacturer Code : 12888004	€ 0.15 / piece	unknown	5 boxes					209/181.7	1045	
Sarstedt	86_1252001	Pipet, 2 ml individueel verpakt	€ 0.06 / pipet	unknown	1x500	500	2	1000		463.13	2315.65	2315.65
Sarstedt	86_1253001	Pipet, 5 ml individueel verpakt	€ 0.08 / pipet	unknown	2x500	500	2	1000		4200	8400	8400
Sarstedt	86_1254001	Pipet, 10 ml individueel verpakt	€ 0.10 / pipet	unknown	2x500	200	6	1200		5350	10700	10700
Sarstedt	86_1685001	Pipet, 25ml individueel verpakt	€ 0.16 / pipet	unknown	6x200	100	9	900		3360	20160	20160
Sarstedt	86_125600	Pipet, 50 ml individueel verpakt	€ 0.48 / pipet	unknown	9x100	1000	1	1000		1900	17100	17100
**Sum**		**Pipettes**				**Sum Pipettes**		**5100**	**0.8**			
Sarstedt	1562010	Inoculation Loops (10ul, blue, sterile)	€ 0.03 / piece	unknown	1x1000	1000	5	5000		14.73	1473	1473
Sarstedt	1567010	entoog 1ul wit per 10	€ 0.03 / piece	unknown	5x1000	1000	5	5000		14.76	7380	7380
Boomlab	38748545	Spuiten 50 ml, PP, Luer-Lock 3-delig, steriel (38748545)	€ 0.69/ piece	unknown	2x50st	50	2	100		35.09	350.9	350.9
VWR	TERUSS30-L1MP	Injectiespuit, medisch, PP, Steriel, Without needle, Luer lock tip, Inhoud: 30 ml, VPE: 25	€ 0.61/ piece	unknown	2x25	25	2	50		23.22	1161	1161
VWR	613-5402	Syringes, Terumo, Sterile, no needle, excentric LT end, 20 ml	€ 0.12 / piece	unknown	10 syringes?					12.88	128.8	128.8
VWR	613-2317	Spuiten, Zonder naald, excentrische LT punt, 10ml	€ 0.11 / piece	unknown	1x100	100	1	100		7.91	791	791
VWR	613-1599	Terumo Syringe 5ml without needle	€ 0.11 / piece	unknown	1x100	100	1	100		4.74/4.30	474	474
VWR	613-2001	Injectiespuit voor de toediening van tuberculine, voor eenmalig gebruik, met schaalverdeling in ml, en luer-tip, 1 ml	€ 0.15 / piece	unknown	1x100	100	1	100				
Sarstedt	82_1473001	petrischaal 92x16mm nok ster.	€ 0.06 / piece	unknown	6x480	480	6	2880		7150	42900	42900
Isogen/Starlab	S1110-3000-C	TipOne® Pipette Tip, 10/20µl XL, Graduated, Bulk	€ 0.02 / piece	unknown	1x9600	9600	1	9600		110.07	11007	11007
Isogen/Starlab	S1113-1000-C	TipOne® Pipette Tip 200µl UltraPoint, Graduated, Bulk	€ 0.01 / piece	unknown	6x9600	9600	6	57600		543.78	32626.8	32626.8
Isogen/Starlab	S1111-6000-C	TipOne® Pipette Tip 1000µl, Graduated, Bulk	€ 0.01 / piece	unknown	5x9600	9600	5	48000		886.09	44304.5	44304.5
Isogen/Starlab	S1111-3200-C	TipOne® Pipette Tip, 10µl, Graduated, Stack Rack	€ 0.03 / piece	unknown	3x9600	9600	3	28800		399.6	11988	11988
**Sum**		**Pipette tips**				**Sum Pipette tips**		**144000**	**23**			
Isogen/Starlab	I1022-0600	Gel Saver II Tip 1-200µl 204 Rack	€ 0.09 / piece	unknown	3x 96	96	3	288		132	396	396
VWR	231-0106	Microspatula, Single use	€ 0.14 / piece	unknown	2x300	300	2	600		135	270	270
VWR	231-0416	Eco-spatels voor eenmalig gebruik, biologisch afbreekbaar (EcoPure®-additief)	€ 0.17 / piece	unknown	2x300	300	2	600		215	430	430
VWR	231-0105	Spatula big, Single use	€ 0.37 / piece	unknown	2x150	150	2	300		440	880	880
VWR	516-0318	Weigh paper, single use, 100×100 mm	€ 0.02 / piece	unknown	1x500					250	250	
VWR	611-9181	LLG weegbakjes 7 ml, PS zwart 46×46×8 mm, antistatisch	€ 0.06 / piece	unknown	2x500	500	2	1000		345	690	690
VWR	611-9182	LLG weegbakjes 100ml PS zwart 85×85×24 mm, antistatisch	€ 0.11 / piece	unknown	2x500	500	2	1000		1140	2280	2280
VWR	611-9180	LLG weegbakjes 250ml PS zwart 140×140×22 mm, antistatisch	€ 0.29 / piece	unknown	2x500	500	2	1000		3150	6300	6300
Boomlab	19045011	Scalpelmesjes fig.11	€ 0.37 / piece	unknown	2 packages					170	340	
VWR	233-5487	Scalpel blades, Roestvrij staal, Steriel, nr. 24	€ 0.57 / piece	unknown	1 package					1.76	1.76	
VWR	514-0030	Bottle-top filter for 45 mm bottles, 0.2um	€ 8.34 / piece	unknown	25x12	12	25	300		8125	203125	203125
VWR	514-8020	Syringe filters, Puradisc™, Whatman™, PES, 0,2 µm, 25 mm	€ 2.05 / piece	unknown	4x50	50	4	200		210	840	840
VWR	514-0570	Membranefilters Whatman, Nylon, For Biomass	€ 1.40 / piece	unknown	2x100	100	2	200				
Sarstedt	62 554002	buis 15ml 120x17mm rode schrd st	€ 0.10 / piece	unknown	3x500	500	3	1500		3500	10500	10500
Greiner	188261	CELLSTAR®, tube, V, PP, 15ml, label, cap	€ 0.20 / piece	unknown	3x1000	1000	3	3000		6400	19200	19200
Sarstedt	62 548004	Buis 50ml 114x28mm PP rode dop, conisch	€ 0.09 / piece	unknown	2x300	300	2	600		4020	8040	8040
Sarstedt	62 559001	buis 50ml 115x28mm pp ster/sta t/ (self standing)	€ 0.12 / piece	unknown	2x300	300	2	600		4020	8040	8040
Greiner	187262	TUBE, 14ML, PP, ROUND BOTTOM, 18 X 95 MM;	€ 0.13 / piece	unknown	3x500	500	3	1500		6.07	9105	9105
Sarstedt	72 704	Reactievaatje 0,5ml safe-seal met brede rand + aanh. dop	€ 0.02 / piece	unknown	1 bag with 100 tubes	100	1	100		268.4	268.4	268.4
Sarstedt	72 70400X	Reactievaatje 0,5ml safe-seal brede rand, kleur	€ 0.03 / piece	unknown						268.42 ?		
Sarstedt	72 706	Reactievaatje 1,5ml safe-seal 39x10,8mm pp	€ 0.01 / piece	unknown	6x2000	2000	6	12000		2500	15000	15000
Sarstedt	72 70600X	Reactievaatje 1,5ml safe-seal brede rand+nok kleur	€ 0.02 / piece	unknown	4x2000	2000	4	8000		2500	10000	10000
Sarstedt	72 695500	Reactievaatje 2ml pp safe-seal neutraal	€ 0.02 / piece	unknown	1x2000	2000	1	2000		2640	2640	2640
Sarstedt	72 69500X	buisje 2ml pp safe-seal kleur	€ 0.02 / piece	unknown	1x2000	2000	1	2000		291.71	2333.68	2333.68
Sarstedt	72,379	CryoPure buis 2ml ext. schr.dr. wit	€ 0.11 / piece	unknown	1100 tubes	1100	1	1100		104.97	2309.34	2309.34
Sarstedt	72_737002	pcr-cup 0,2ml niet steriel	€ 0.02 / piece	unknown	3x2000	2000	3	6000		98.23	294.69	294.69
Sarstedt	72_991002	PCR-strip v 8, PCR-PT	€ 0.28 / strip	unknown	1x2000	2000	1	2000		188.27	188.27	188.27
Sarstedt	72_1978202	PCR pp plaat 96well, 0,3ml z.rand 25/zak	€ 2.19 / piece	unknown	1x25	25	1	25		1046	1046	1046
Sarstedt	82_1581	Microtestplate 96 well, F	€ 0.25 / piece	unknown	1x100	100	1	100		4170	4170	4170
Sarstedt	82_1972002	Megablock square wells 2.2. ml PP	€ 2.61 / piece	unknown						391.18		
Sarstedt	82 1581120	ELISA plate black Med.Bind., F	€ 1.65 / piece	unknown						1034.68		
Sarstedt	82_1584	Lid for Microtest Plate	€ 0.19 / piece	unknown	1x100	100	1	100		1460	1460	1460
BD	653156	Bacteriostatic Concentrate Solution	€ 5.96 / bottle	unknown	1x10 vials	10	1	10		124.8	124.8	
BD	653157	Cleaning Solution Concentrate	€ 5.96 / bottle	unknown	1 vial	1	1	1		16.02	16.02	
KRSS	VI-04-12-02L	2ml Clear robotic 9mm Screw top vial with label (PK/1000)	€ 0.11 / piece	unknown	2x1000	1000	2	2000				
KRSS	IN-CB-02	250ul Conical glass inserts (6mm OD) (PK/1000)	€ 0.12 / piece	unknown	1x1000	1000	1	1000				
KRSS	CP-B-04-11-04	Blue 9mm screw cap with white silicone/red ptfe septa (PK/1000)	€ 0.13 / piece	unknown	3x1000	1000	3	3000				
Baseclear	KI-007860ACL	ClearSpin filter microtubes, nylon, porosity 0.22 µm, no sterile	€ 0.82 / piece	unknown	15x100	100	15	1500		138.9	2083.5	2083.5
Instech Laboratories	LS20	20 Gauge luer stubs	€ 0.45 / piece	unknown						40g		
mavom bv	1060040	DC Sylgard 184 elastomer	€ 273.00 / piece	unknown								
Metrohm NL	EW-06418-03	Tygon microbore Tubing, 0.030" ID x 0.090" OD, 100'	€ 86.00 / piece	unknown						240.55		
Metrohm NL	06417-11	PTFE #30 AWG Thin Wall Tubing Natural	€ 28.90 / piece	unknown						126.25		
Sigma-Aldrich	85403-1EA	ilicone grease Bayer - high vacuum 35 g (Baysilone paste	€ 21.10 / piece	unknown								
Werken met Merken B.V	ISON KOMBI SNE	N KOMBI SNEL, Glasheldere snelle 2-componenten epox	€ 9.95 / piece	unknown								
Instech Laboratories	SP20/12	Stainless steel catheter plug, 20ga x 12mm, non-sterile	€ 0.55 / piece	unknown								
DiversifiedBiotech	TTLW-1000	Tough-tags for 1.5-2.0ml tubes	€ 23.00 / piece	unknown								
VWR	631-1550	Objectglaasje met gesneden randen en normaal uiteinde	€ 0.04 / piece	unknown								
CBN	1121226245	Erlenmeyer, 100ml, WM	€ 1.45 / piece	unknown	20	20				77.44	1548.8	
CBN	1121216445	Erlenmeyer, 500ml, NM	€ 1.75 / piece	unknown	20	20				178.6	3572	
Stores		Alumium foil			9x	9				210	1890	
Stores		Autoclave tape			13x	13				115	1495	
**Stores**		**Cuvettes**			23boxes	100	23	**2300**	**0.4**	270	6210	6210
Stores		Matches			9x	9				80	720	
Stores		Paper towels			11x6	11	6	66		7000	77000	
Stores		Parafilm			2x	2	1	2		660	1320	1320
Stores		Pasteur pipettes			1x250	250	1	250		752.91	752.91	
					**TOTAL (g)**						**677825**	**532681**
					**TOTAL (kg)**						**678**	**533**
					**TOTAL (kg) per person per month, 17 researchers**						**3.3**	**2.6**
					**TOTAL (kg) per person per year, 17 researchers**						**39.9**	**31.3**

2.7.6.3 Autoclaving experiment of plastic syringes for reuse

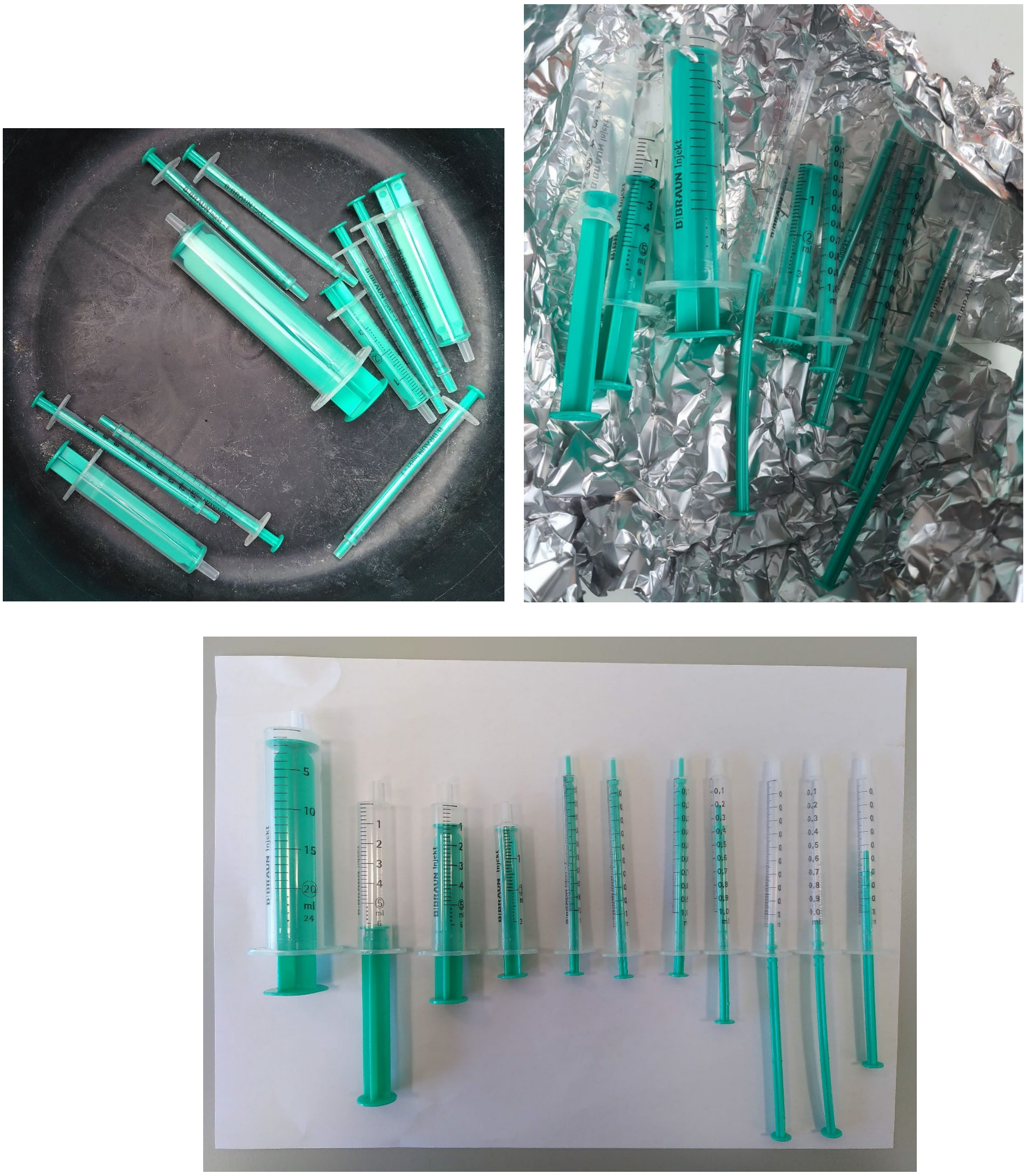

Figure S27: Syringes before (top left) and after (top right and bottom) autoclaving for reuse in a chemistry laboratory. Stains and remaining water droplets led to unfeasibility.

2.7.7 Marketplace for second-hand laboratory equipment

Website
In the menu on the left hand side of this page: https://myuniversity.rug.nl/infonet/medewerkers/fse/fse-research/green-labs/

Old lab equipment is often stored somewhere in the lab for a long time. This equipment is sometimes donated to colleagues, but too often they are only taking up space in the lab to only be thrown out years later. This is a pity because selling this equipment frees up space in your lab and will financially benefit your lab group. To facilitate this, you can use Labmakelaar.com, a Benelux-based second-hand equipment trader.

Three options

Labmakelaar.com provides three ways of selling your equipment.

The first, concerning a direct purchase by Labmakelaar.com, is the easiest. You provide all the information necessary, and Labmakelaar.com gives you their quotation for buying it. This money will be transferred to your project code directly.

The second method is to have Labmakelaar.com sell it for you 'in consignment'. This means that the equipment is still in your possession, but that Labmakelaar will take care of all practicalities. They will make sure the item is correctly calibrated, is functioning properly, and will transport it to the eventual buyer. You are only responsible for sending the piece of equipment to Labmakelaar.com and have to reimburse any reparation costs if it is broken (*i.e.* refurbishing). In the end, you will receive 50% of the eventual amount the object was resold for.

The third method is Labmakelaar facilitating the resell of your piece of equipment, without having it in consignment. This means you are responsible for calibration, repairs, transport, and sending an invoice to Labmakelaar. They will put the item on their website and will process the request by the buyer. Using this method, Labmakelaar is entitled to 21% of the agreed-upon amount but this will result in more work for you.

The procedure

If you want to make use of either the first or second option, please send Labmakelaar.com an e-mail (info@labmakelaar.com) with all the information as it appears in the "product aanmaken" form. In their reply, they will either offer to buy it directly from you or offer to take it into consignment. If you only want Labmakelaar.com to mediate between you and the buyer, you can fill out their form. Please make sure to include your management controller and financial controller in cc, to make sure they know that you intend to sell a certain item. After a certain item is sold and the money received by the university, the sale of that specific piece of equipment will be disinvested. This means that the depreciation will be increased, which will be deducted from the sale price. Hence, the money received by your lab group will in some cases be lower than the amount agreed upon by Labmakelaar.com and you. In all cases, please make sure to read and abide by their terms and conditions.

Legality

Most lab equipment can be sold without any problems. However, it is important to realize that if a certain piece of equipment was bought using specific project funds, it is not eligible for resale. Of course, if a certain subsidy specifically mentions that the bought equipment is not eligible for resale, this also means that the piece of equipment cannot be sold.

2.7.8 Art from laboratory waste

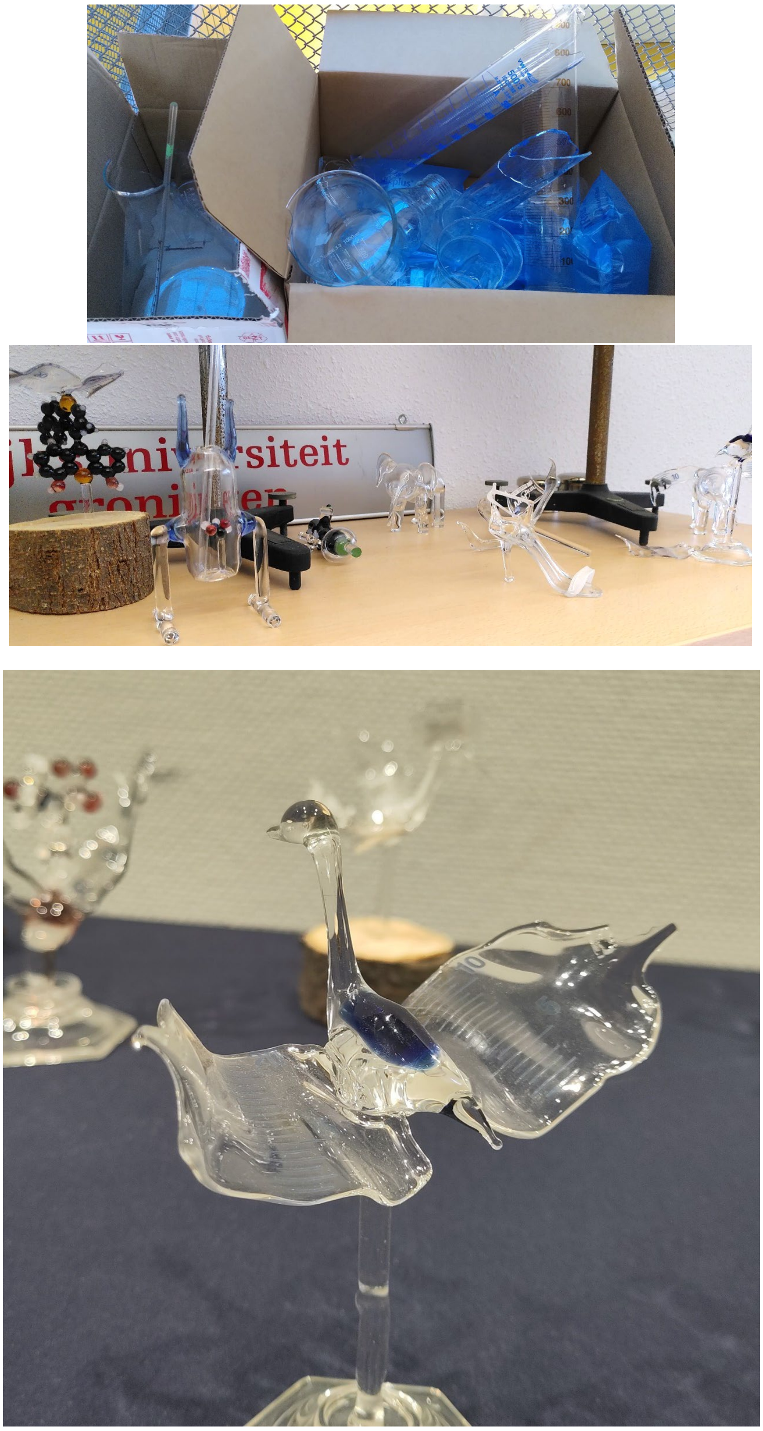

Figure S28: Swan and other glass sculpture made from broken glassware.
Artist credit: M. C. A. Stuart, University of Groningen.

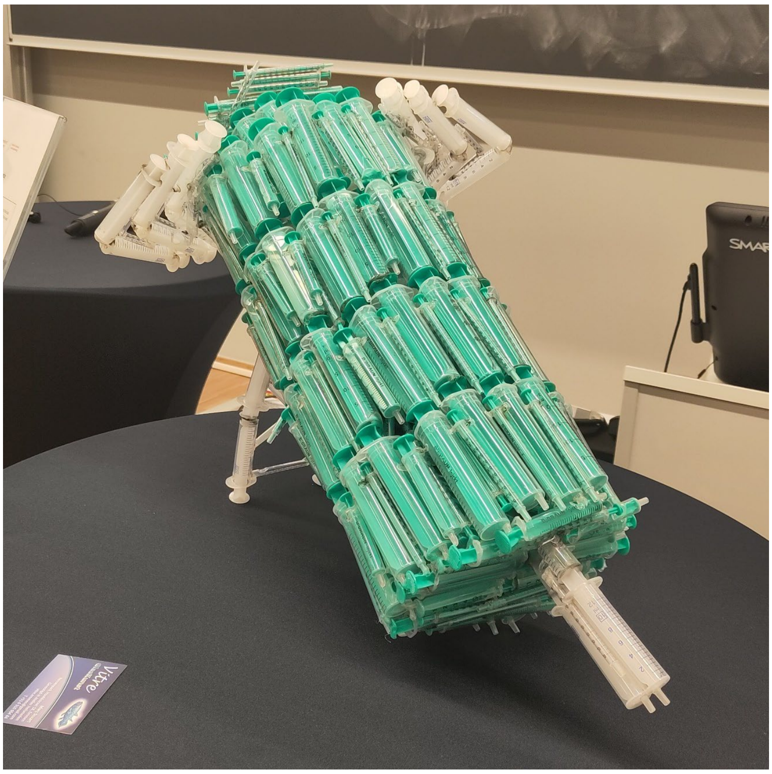

Figure S29: Syringe made from syringes plastic waste collected from the pilot laboratory.
Artist credit: M. Heinhuis, University of Groningen.

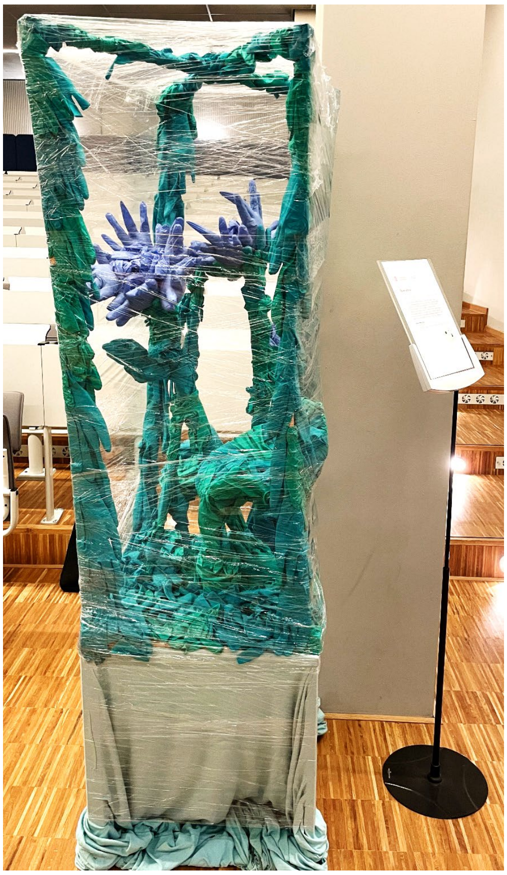

Figure S30: Flower art made from glove waste.
Artist credit: A. Hallik, University of Groningen.

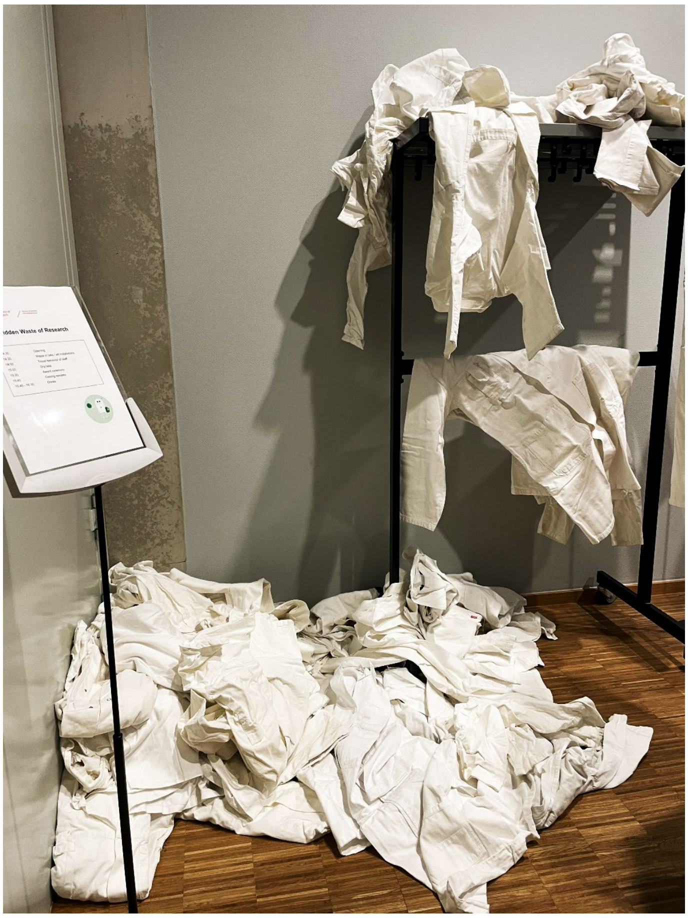

Figure S31: Old and forgotten lab coats piling up. *Artist credit: T. Freese & P. Nauw, University of Groningen.*

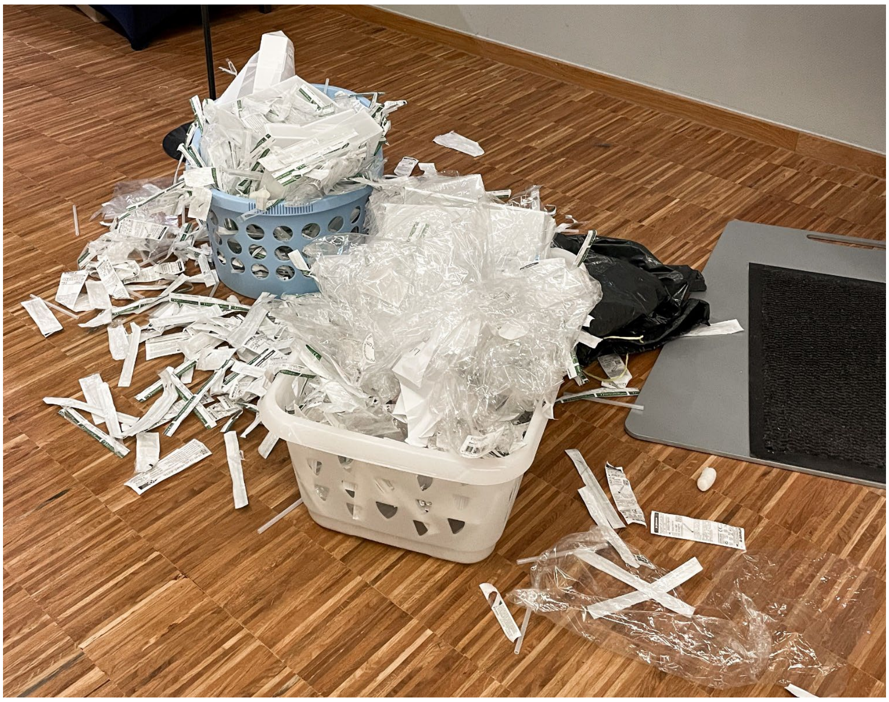

Figure S32: Packaging material waste from laboratory single-use consumables.
Artist credit: T. Freese, University of Groningen.

2.7.9 Scope 3 - Furniture CO_2e calculations at FSE

The following data has been acquired through G. Boesjes and N. Elzinga and is being published with permission of the Faculty of Science and Engineering, University of Groningen.

Method

The carbon footprint of the inventoried furniture at the Faculty of Science and Engineering (FSE) was calculated using the Energy Analysis Program (EAP).[16] This program uses a hybrid method to calculate the environmental impact of a product or service.

This hybrid method consists in part of a Life Cycle Analysis (LCA). In an LCA, a product is described as a system. There raw materials and materials needed for manufacturing of the product, and production methods (preparation, production of materials, transportation, *etc.*) are included in the analysis. Environmental impacts are calculated for each component.

Furthermore, EAP also uses Input-Output Analysis. This looks at the connections between different industries in the economy, including their environmental impacts. It reveals how transactions between certain industries affect each other. This allows you to determine from a financial transaction (the market price of the product) what - on average - the carbon footprint of that product is.

Inventory

For each piece of furniture, the following data must be entered into EAP:

1. General information
    a. Weight (kg).
    b. Transport weight (kg)
    c. Price (€)
    d. VAT rate (%)
2. Production
    a. Type of manufacturer
3. Materials
    a. Basic Goods
    b. Packaging materials
4. Transportation
    a. Distance by transport type (km).
5. Trade
    a. Market type.

The weight and price of each product were looked up on websites of sellers of this furniture. Estimates were made where necessary. A VAT rate of 21% was assumed for each product. EAP offers a wide selection of manufacturer types from which to choose. In this analysis, Manufacturer of furniture (code 31 in EAP) was chosen. For each product, the packaging is assumed to be 85% cardboard, 10% Styrofoam (polystyrene) and 5% plastic (LDPE). It is further assumed that each product must be transported on average 200 km by truck, and that the products are traded through furniture wholesalers (wholesale trade furniture, soft furnishings, floor covering).

Results

The table below depicts the emission factor per furniture item, the total number inventoried, and the total emissions in CO_2 equivalents as calculated in EAP. All the furniture of Nijenborgh 4 correspond to emissions of 1032 tons CO_2e.

Table S47: Furniture inventory and its corresponding carbon impact of NB4 (Stratingh Institute of Chemistry, etc.)

Furniture	Type	Emission factor (kg CO_2e)	Amount	Emissions (tons CO_2e)
Office chair	-	185	1718	319
Desk	-	210	1155	243
Drawer unit	*Hanging*	70	151	11
	Standing	145	1114	161
Cupboard	*Blinds high*	184	345	63
	Blinds middle	163	68	11
	Blinds low	142	46	7
	Archive high	140	111	16
	Archive low	122	60	7
Meeting chair	-	36	1905	69
Meeting table	-	181	690	125
**Total**				**1032**
**Annual emissions *via* years of depreciation (10 years)**				**103.2**

Validation

Environmental impact analyses often involve relatively large margins of uncertainty. In the inventory of materials used and underlying manufacturing processes of the furniture, many assumptions were made that affect the final result. No large amount of literature research has been done on the environmental impacts of office furniture. A small number of results found are nevertheless highlighted below.

A University of Michigan study reports emissions of 114 kg CO_2e for office chairs, and 220 kg CO_2e for office tables.[17] The British agency FIRA reports 72 kg CO_2e for office chairs, 36 kg CO_2e for conference chairs.[18] *Medeiros et al.* calculated from a 2017 LCA that an office cabinet provides 122 kg CO_2e.[19] A publication by *Ahrend* (the manufacturer of the UG's furniture) mentions how many CO_2 emissions were saved by TU Delft, which purchased refurbished furniture from *Ahrend*. There approximately 140 kg CO_2e per piece of furniture was calculated,[20] which agrees reasonably well with the average value calculated in this analysis.

It is worth noting that the results from EAP seem structurally higher. It is recommended to interpret the results of this analysis carefully and to keep at least a 10% margin of error.

Furniture footprint of FSE

For the calculation of the furniture carbon impact at FSE the inventory of NB4 was correlated through its area to the areas of all buildings of FSE. We further calculated the annual CO_2e emissions through correlating the emissions to average years of depreciation of ten years, resulting in 103 tons CO_2e annually. Afterwards the m^2 of office and meeting room space of each building at FSE was used to calculate the furniture footprint per building as well as the total, which corresponded to an annual CO_2e footprint of 304 tons for FSE.

Table S48: Furniture carbon impact (tons CO_2e) of FSE and contribution of each building.

Building	FNO per building (m^2)	Furniture (tons CO_2e)
Nijenborgh 4	32 272	103.2
Linnaeusborg	25 373	81.1
Bernoulliborg	8591	27.5
EAE	6040	19.3
ZL25	7576	24.2
Herdershut	389	1.2
Lutjewad	169	0.5
A. Deusinglaan	10 871	34.8
FEB/kapteyn	3669	11.7
**Total**	**94 950**	**303.6**

2.7.10 Interviews and outreach

#	Publisher	Date	Journalist	Title	Link
**1**	Ukrant	30.06.21	Laura Nederveen	Labs should be greener	https://ukrant.nl/magazine/labs-should-be-greener/?lang=en
**2**	Ukrant	21.11.22	Eoin Gallagher	Chemistry labs are going green	https://ukrant.nl/chemistry-labs-are-going-green-the-drive-is-in-the-young-generations/?lang=en
**3**	ScienceLinx	18.01.23	Myrna Kooij	Laboratories are going green	https://www.rug.nl/sciencelinx/nieuws/2023/01/laboratories-are-going-green
**4**	MyUniversity	14.03.23	*E. W. Zinkstok*	LEAF project launch: six labs receive awards for sustainability	https://myuniversity.rug.nl/infonet/medewerkers/fse/announcements/leaf-project-launch
**5**	Faculty Newsletter	20.03.23	*E. W. Zinkstok*	Seven labs receive LEAF awards for sustainability	https://www.rug.nl/fse/news/seven-labs-receive-leaf-award
**6**	Chemisch 2 Weekblad	13.04.23	Renee Canrinus-Moezelaar	Lab moet zelf ook duurzamer werken	https://www.sciencelink.net/verdieping/lab-moet-zelf-ook-duurzamer-werken/21235.article
**7**	Bionieuws	01.09.23	Stijn van Schie	Lab kan stuk duurzamer	print
**8**	YouTube	16.10. 23	Green Lab RUG FSE	Green Lab Movie	https://www.youtube.com/watch?v=Zk_CEmyHZZg
**9**	MyUniversity	30.10. 23	*E. W. Zinkstok*	Sustainable labs are on the rise: 46 labs now LEAF accredited	https://myuniversity.rug.nl/infonet/medewerkers/fse/news/sustainable-labs-are-on-the-rise
**10**	ChemRxiv	01.12.23	T. Freese *et al.*	A guidebook for sustainability in laboratories	https://doi.org/10.26434/chemrxiv-2023-g3lmq-v2

3. References

1. J. Ciers, A. Mandic, L. D. Toth, and G. Op ’t Veld, “Carbon Footprint of Academic Air Travel: A Case Study in Switzerland,” *Sustain. Sci. Pract. Policy*, vol. 11, no. 1, p. 80, Dec. 2018.
2. S. van Ewijk, S. Chaudhary, and P. Berrill, “Estimating passenger emissions from airfares supports equitable climate action,” *Environ. Res. Lett.*, vol. 18, no. 2, p. 024013, Jan. 2023.
3. “Sustainable business travel policy,” 2023. https://www.rug.nl/about-ug/profile/facts-and-figures/duurzaamheid/projecten/nieuw-zakelijk-reisbeleid?lang=en (accessed Apr. 12, 2023).
4. https://myuniversity.rug.nl/infonet/medewerkers/fse/fse-topics/sustainability/
5. https://www.rug.nl/about-ug/profile/facts-and-figures/duurzaamheid/roadmap-sustainability
6. Van der Tak et al (2021), *The carbon footprint of astronomy research in the Netherlands*, Nature Astronomy, Vol. 5, p. 1195-1198; doi:10.1038/s41550-021-01552-4.
7. https://www.rug.nl/about-ug/profile/facts-and-figures/duurzaamheid/praktischeinformatie/documenten
8. Larsson et al (2019), *International and national climate policies for aviation: a review*, Climate Policy, 19:6, 787-799; DOI: 10.1080/14693062.2018.1562871.
9. Mariette et al (2022), *An open-source tool to assess the carbon footprint of research*, Environ. Res.: Infrastruct. Sustain., 2, 035008; DOI: 10.1088/2634-4505/ac84a4.
10. Steg, L., Dreijerink, L., & Abrahamse, W. (2006). *Why are energy policies acceptable and effective?* Environment and behaviour, 38(1), 92-111.
11. Schuitema, G., & Steg, L. (2008). *The role of revenue use in the acceptability of transport pricing policies.* Transportation Research Part F: Traffic Psychology and Behaviour, 11(3), 221-231.
12. Schuitema, G., & Bergstad, C. J. (2018). *Acceptability of environmental policies.* Environmental psychology: An introduction, 295-306.
13. Schuitema, G., Steg, L., & Forward, S. (2010). *Explaining differences in acceptability before and acceptance after the implementation of a congestion charge in Stockholm.* Transportation Research Part A: Policy and Practice, 44(2), 99-109.
14. Israel, G.D. (1992) *Determining Sample Size.* University of Florida Cooperative Extension Service, Institute of Food and Agriculture Sciences, EDIS, Florida
15. Taherdoost, H. (2017), *Determining Sample Size; How to Calculate Survey Sample Size.* International Journal of Economics and Management Systems, Vol. 2.; https://ssrn.com/abstract=3224205
16. R. M. J. Benders, W. Louwes, and H. C. Moll, *EAP: Energy Analysis Program, Manual Version 3.0, IVEM Research Report no. 95*. s.n., 1998.
17. David V. Spitzley Bernhard A. Dietz Gregory A. Keoleian, “Life-Cycle Assessment of Office Furniture Products Final report on the study of three Steelcase office furniture products: Airtouch table, Garland desk, and Siento chair,” Center for Sustainable Systems , University of Michigan, CSS06-11, 2006.
18. Furniture Industry Research Association, “A study into the feasibility of Benchmarking carbon footprints of furniture products.” 2011.
19. D. L. Medeiros, A. O. do C. Tavares, Á. L. Q. R. e. S. Rapôso, and A. Kiperstok, “Life cycle assessment in the furniture industry: the case study of an office cabinet,” *Int. J. Life Cycle Assess.*, vol. 22, no. 11, pp. 1823–1836, 2017.
20. Ahrend, “TU Delft bespaart tonnen CO_2 met gerefurbisht meubilair.” https://www.ahrend.com/nl/over-ahrend/stories/tu-delft-bespaart-tonnen-co2-met-gerefurbisht-meubilair/ (accessed 28 Nov., 2023).
21. Roggema, R. From nature-based to nature-driven: Landscape first for the design of moeder zernike in groningen. *Sustainability* **13**, 1–21 (2021).
